# A revision of Chilicola (Heteroediscelis), a subgenus of xeromelissine bees (Hymenoptera, Colletidae) endemic to Chile: taxonomy, phylogeny, and biogeography, with descriptions of eight new species

**DOI:** 10.3897/zookeys.591.7731

**Published:** 2016-05-19

**Authors:** Spencer K. Monckton

**Affiliations:** 1 Department of Biology, York University, Toronto, Ontario, Canada

**Keywords:** Apoidea, Xeromelissinae, integrative taxonomy, systematics, maximum parsimony, morphology, continuous characters, DNA barcoding, spatial analysis of vicariance, historical biogeography

## Abstract

The bee subgenus Chilicola (Heteroediscelis) Toro & Moldenke, 1979 (Hymenoptera, Colletidae, Xeromelissinae) is revised. The subgenus is considered endemic to Chile and occurs across a broad range of habitats. Eight new species are described: Chilicola (Heteroediscelis) charizard Monckton, **sp. n.**, Chilicola (Heteroediscelis) curvapeligrosa Monckton, **sp. n.**, Chilicola (Heteroediscelis) guanicoe Monckton, **sp. n.**, Chilicola (Heteroediscelis) katherinae Monckton, **sp. n.**, Chilicola (Heteroediscelis) lickana Monckton, **sp. n.**, Chilicola (Heteroediscelis) mayu Monckton, **sp. n.**, Chilicola (Heteroediscelis) packeri Monckton, **sp. n.**, and Chilicola (Heteroediscelis) randolphi Monckton, **sp. n.** One of the existing species, Chilicola (Heteroediscelis) valparaiso Toro & Moldenke, 1979, **syn. n.**, is treated as a junior synonym of Chilicola (Heteroediscelis) mantagua Toro & Moldenke, 1979, and the nine remaining valid species are redescribed. Thoroughly illustrated keys to species for males and females are provided, along with habitus images, images of male terminalia, distribution maps for each species, and a map of relevant Chilean biogeographic regions. Results of phylogenetic analyses are presented, based upon 74 morphological characters and on CO1 barcode sequences, analyzed both separately and as a combined dataset. Monophyly of the subgenus is supported, and groupings within the subgenus are discussed in light of a biogeographic analysis of their species distributions (spatial analysis of vicariance), whereby divergence between taxa is found to occur primarily via north-south disjunctions.

## Introduction

*Chilicola* Spinola, 1851 is a diverse neotropical genus of stem-nesting bees, comprising 98 named species (Ascher and Pickering 2015) in 15 subgenera (Packer 2008). The subgenus Chilicola (Heteroediscelis) Toro & Moldenke, 1979 is notable in that its 10 named species are endemic to Chile (Packer 2008), where they occupy a broad range of habitats, from the hyper-arid Atacama Desert in the north, to moist temperate forests in the south, occurring at elevations from sea level to over 3200 m a.s.l.. Bees of the subgenus *Heteroediscelis* are small and slender, 4–7 mm long, and hylaeiform in appearance. They are mostly dark brown or black, with females having variable yellow markings on the legs and ventral surface of the antennae, and males having more extensive yellow colouration, particularly on the face. In males, the distinctly expanded hind legs and the robust, truncate process extending ventrally from the first sternum (S1 process) together readily distinguish this subgenus from all other *Chilicola*.

The subgenus *Heteroediscelis* was first described with 13 species (Toro and Moldenke 1979). Three of these species were later moved to Chilicola (Anoediscelis) Toro & Moldenke by Michener (1995) due to obvious differences: their males lack the combination of expanded hind legs and truncate S1 process characteristic of the other ten species. Toro and Moldenke (1979) recognized these differences but did not classify the three species in a different subgenus, citing the lack of corresponding differences in females as justification. Michener (1995) moved the other ten species to Chilicola (Oediscelis) Philippi. Packer (2008) performed a phylogenetic analysis of *Chilicola* with exemplars from all known subgenera plus additional outgroup taxa, and found very strong support for the monophyly of *Heteroediscelis*, along with moderate support for its placement as sister to a well-supported clade formed by Chilicola (Chilioediscelis) Toro & Moldenke, 1979, and *Chilicola* *s. str.* Spinola, 1851; consequently, he resurrected the subgenus.

A number of new species belonging to *Heteroediscelis* have been recognized in collections for some time, several of them from geographic regions novel to the subgenus. However, no species-level taxonomic work has been done on the group since its description, and its phylogeny is unknown. Here, I synonymise one of the original species, redescribe the remaining nine and describe eight new species, bringing the total within the subgenus to seventeen. I also present a thorough phylogenetic analysis for the subgenus, as well as a discussion of the historical biogeography of these interesting little bees.

## Methods

### Taxonomy

For the revision, 1316 specimens of *Heteroediscelis* were examined. Of these, 344 were collected during field excursions over the course of this study, and the remaining 972 were obtained from five collections: the Packer Collection at York University, Toronto, Canada (PCYU), the American Museum of Natural History, New York, U.S.A. (AMNH), the collection at the Pontificia Universidad Católica de Valparaíso, Chile (PUCV), the Bee Biology and Systematics Laboratory, Logan, UT, U.S.A. (BBSL), and the collection at the Universidade Federal do Rio de Janeiro, Brazil (UFRJ). In addition to these collections, a number of specimens from PCYU will also be deposited at the Central Texas Melittological Institute (CTMI) upon completion of this work. As all specimens were collected in Chile, all locality information is written with country excluded.

Specimens were identified using the existing key (Toro and Moldenke 1979) and those not matching the descriptions of the documented species were sorted into putative OTUs (operational taxonomic units) based on morphological characters. Sorted specimens were initially compared with paratype material of existing species where possible (later to holotypes for all species). Subsets of specimens from these sorted groups were selected for DNA barcoding (Hebert et al. 2003), particularly where either barcode sequences for that species were apparently not already recorded, or where specific identity was uncertain and comparison could be made to previously databased sequences.

For barcoding, a midleg from each specimen was removed and placed into a single well of a 96-well plate along with three drops of 95% EtOH. Extraction, PCR, and sequencing of the cytochrome *c* oxidase subunit I (CO1) were all done at the Canadian Centre for DNA Barcoding (CCDB) in Guelph, Canada, using standardized protocols (see Hebert et al. 2003, Ivanova et al. 2006). Successful output sequences were uploaded to the Barcode of Life Data Systems database (BOLD) (Ratnasingham and Hebert 2007). In order to check putative identifications against CO1 sequence data, barcode sequences were investigated in two ways: first, BOLD’s built-in analysis platform was used to generate neighbour-joining trees from DNA barcodes based on the Kimura two-parameter (K80) base substitution model (Kimura 1980); second, all barcode sequences over 300bp were assigned Barcode Index Numbers (BINs) representing groups of similar sequences automatically generated by BOLD using a clustering algorithm (Ratnasingham and Hebert 2013). Sequences belonging to different BINs or to different groups on the neighbour-joining tree can subsequently be checked for morphological differences.

Accordingly, barcode sequences were used in species delimitation employing an integrative approach (e.g. Gibbs 2009a, 2009b, DeSalle et al. 2005, Will et al. 2005, Meier et al. 2006). Identified BINs were investigated at the specimen level for any morphological differences that might support further separation into distinct groups, and additional specimens were selected for barcoding as necessary for a representative range of morphological variation and geographic coverage. Throughout, morphological characters were given precedence over DNA barcodes, as clear morphological differences were in some cases obvious among members of the same BIN (an outcome also noted by Gibbs 2009b). Groupings consistently supported by morphological characters were considered to be distinct species (i.e. using the phylogenetic species concept; Nixon and Wheeler 1990). Names assigned to groupings representing existing species were confirmed following examination of holotype material at AMNH.

Body measurements were taken using a calibrated ocular micrometer mounted on a Nikon SMZ1500 stereomicroscope. Measurements of body length, head width, thorax width, and forewing length were recorded in millimeters (mm). Due to distortions of the mesothorax from pinning these small bees, the standard measure of intertegular width could not be accurately assessed, and thorax width was instead measured across the pronotal lobes. Because the tagmata were not always in the same plane, body length was measured in sections (head, meso- and metasoma) and summed. To facilitate comparisons, measurements of pubescence are given relative to the diameter of the median ocellus (OD). Surface sculpture is described with puncture density stated in terms of puncture diameter (d) and interspace (i). All other measurements are expressed as ratios, with values representing ocular units at a common magnification of 90, where one ocular unit equals approximately 0.0137mm; these values have an approximate measurement error of +/- 0.5 units. For measurements of the pedicel and antennal flagellomeres, greater precision was required, and these measurements were verified at 112.5 magnification (one ocular unit equals approximately 0.0110mm); their measurement error is estimated as +/- 0.25 units.

Specimen records not originally having GPS coordinates are supplemented with them where possible, as determined via discussions with the collectors and using Google Earth (2013). Supplemented GPS coordinates are rounded to three decimal places and italicized in the descriptions, and include elevations recorded from the nearest road or path according to Google Earth. Specimens with barcode reference numbers or index/database numbers have these codes listed where applicable; multiple codes for a single specimen are listed separated by a double forward slash (i.e. //), and when multiple specimens are listed for a single locality, these codes are given in respective order, separated by commas. Each specimen was given a unique ID number (e.g. C.kate.001) in order to allow explicit reference to individual bees; these number codes are listed along with all specimen records in Suppl. material 2.

The following acronyms are used: OD: diameter of the median ocellus; MOC: distance from lower tangent of median ocellus to apex of clypeus; OOC: ocellocular distance, shortest distance between lateral ocellus and compound eye; IOC: interocellar distance, shortest distance between lateral ocelli; UOD & LOD: upper & lower interocular distance, shortest distance between inner orbits of compound eyes, above & below level of emargination, respectively; IOD: maximum interocular distance, or greatest distance between inner orbits of compound eyes at level of emargination; LOT: lower ocular tangent, an imaginary line crossing lower extremities of compound eyes in frontal view, perpendicular to main axis of face; IAD: interalveolar distance, shortest distance between antennal sockets; AOD: minimum antennocular distance, shortest distance between antennal socket and compound eye, typically at an angle of approximately 30° below horizontal laterad from antennal socket; F: antennal flagellomere; T: tergum; S: sternum.

Descriptions use terminology from Harris (1979) for surface sculpture and standard melittological terminology from Michener (2007) for structure, with the exception of the following non-standard terms: *stria* refers to a raised, rather than impressed, lineation; *vertexal area* refers to the dorsalmost surface of the head, between the compound eyes, frontal area, and occiput; *clypeal lateral* refers to the lateral, ventrally-bent portion of the clypeal apex; *genal beard* refers to the long erect hairs of the genal area; *stigmal perpendicular* refers to an imaginary line through the apex of the stigma and perpendicular to the costal margin of the forewing (Packer 2008); *ventral metatibial convexity* refers to the apicoventral expansion of the male metatibia, which has a readily defined *summit*; *posterior metatibial concavity* refers to the wide, deep longitudinal groove which extends the length of the male metatibia on the posterior surface; *ventral/posterior metatibial carina* refers to the *carinae* associated with the posterior concavity of the male metatibia; *apical metatibial lamina* refers to the structure extending mediad from the base of the male metatibial spurs; *metapostnotum* refers to the dorsal surface of the propodeum (*sensu* Brothers, 1976). The following assumptions are made: when describing colouration, orange in place of normal yellow colouration is assumed to indicate discolouration due to exposure to cyanide, and is described as yellow for discoloured specimens; when describing legs, they are assumed to be fully extended and at right angles to the longitudinal axis of the body. Finally, *frontal view* is used to refer specifically to the angle of observation at which the lower tangent of the median ocellus and the apex of the clypeus are in a common plane.

Distributions were plotted using ArcGIS 10.3.1 (ESRI 2015). Speculative ranges are minimum-bounding convex hulls (IUCN 1994), smoothed with Bezier interpolation and enlarged in Adobe Illustrator CS5 to surround all occurrence markers (typically 115%).

All specimen images were taken using a Visionary Digital lift-operated imaging system and a Canon 5D Mark II digital SLR camera. Composite images were assembled using Helicon Focus stacking software. Images were edited, cropped, and scale bars added using Adobe Photoshop CS6 Extended ver. 13.0. Photographs marked in the caption with a “†” have been flipped horizontally to ease comparison. All images of new species are of the holotype (male) or allotype (female) except where otherwise indicated in the captions.

### Phylogenetic analysis

Outgroups were selected based on the findings of Packer (2008); Chilicola (Oediscelis) vernalis (Philipi, 1866), Chilicola (Chilicola) rubriventris Spinola, 1851, and Chilicola (Chilioediscelis) penai Willis & Packer, 2008 were chosen. *Chilicola penai* and the type species of the genus *Chilicola rubriventris* were chosen as representative members of the sister clade to *Heteroediscelis*, while *Chilicola vernalis* was chosen to represent *Oediscelis*, which is considered sister to the clade comprising *Heteroediscelis*, *Chilicola* s. str. and *Chilioediscelis*.

Specimens were examined for morphological characters that were potentially phylogenetically informative, and additional characters were adapted from the key and diagnoses of Toro and Moldenke (1979), and from the works of Gibbs and Packer (2006), Willis and Packer (2007), and Packer (2008). A total of 74 morphological characters were coded for each species, comprising 54 discrete and 20 continuous characters, the latter representing ratios between measured structures. Care was taken not to select inter-dependent characters; for example, characters were only included for both sexes when they did not vary in a consistent manner between males and females, thereby providing different phylogenetic information.

Leg colour characters in both sexes were considered to be potentially confounded, based on the observation that some combinations of colouration are never observed. In other words, certain changes in colour of different leg regions may occur together in single evolutionary steps. Non-redundant linear coding (NRLC; O’Grady and Deets 1987) of leg colour characters was therefore used to reduce fourteen potentially confounded characters to three pairs of multi-state characters representing the full range of leg colour variation (see Appendix 3 for explanation).

To ensure independence between continuous morphological characters, regression analyses were performed both on pairs of characters suspected to covary, as well as on male and female characters representing the same structure. Using this approach, adapted from Koch et al. (2015), characters were treated as dependent when they were found to have a significant linear regression without any outliers, indicating that they provide equivalent phylogenetic information – i.e. the variation in one character reliably predicts that of the other for all species. Conversely, the presence of an outlier for even one species is evidence that the two characters offer different phylogenetic information and are independent. Therefore, the magnitude of the difference between observed and expected values was calculated for each data point, and a threshold of 15% was used to identify outliers (arrived at through qualitative comparison of residuals between probable pairs of dependent and independent characters). For pairs of characters found to be dependent, the less variable character was discarded in order to capture maximal variation among species. For traits coded as discrete in one sex and continuous in the alternate sex, independence was assessed using continuous measurements taken from both sexes; if a significant linear regression was found with no outliers differing more than 10% from expected values, the continuous character in the alternate sex was discarded in favour of the discrete character. Finally, continuous characters were re-scaled to unity to avoid biases from magnitude (e.g. Koch et al. 2015).

Representative CO1 sequences were aligned in BOLD with default settings and using ClustalW. The sequence trace files were inspected in BOLD and sequences were edited if applicable – occasionally, BOLD truncates one or both ends of a sequence if it encounters a poor-quality read for a single base pair separating the tail from the rest of the sequence, even when the base pair reads beyond it are high-quality themselves. In such cases, excluded nucleotides were edited back into the full sequence for analysis. In all, this resulted in three datasets for phylogenetic analyses in TNT (Goloboff et al. 2008): morphological data only, molecular data only, and a combined dataset of both.

Maximum parsimony analysis was performed using extended implied weighting (Goloboff 1993, Golobof et al. 2008, Goloboff 2014) whereby discrete, continuous, and molecular data were treated as separate partitions in each analysis; the concavity value K was calculated independently for each partition using the “setk.run” script (as in Santos et al. 2014). The following values were used: discrete morphological, K = 5.78125 (N = 15.03); continuous morphological, K = 0.800781 (N = 15.05); molecular, K = 3.046875 (N = 14.86). Continuous characters are treated as additive by TNT (Goloboff et al. 2006) and characters derived from NRLC were also coded as additive (see Appendix 3). For all three analyses, a tree search was run for 100 replicates, using a driven search with default settings for ratchet, drift, and tree-fusing. The output was visualized using WinClada (Nixon 2002) and characters were mapped using unambiguous optimization, except where noted (see Suppl. material 1 for character-annotated tree, Fig. S1). Tree support is reported by fit, consistency index (CI), and retention index (RI). Support for nodes of the most parsimonious tree was assessed in TNT using absolute frequencies for bootstrap (BS) and GC values from symmetric resampling, both with 1000 replications (Goloboff et al. 2003). GC values represent the percentage of times that a given grouping was found at a node minus the number of times that the next most frequent arrangement was found without that grouping.

### Biogeography

Biogeographic areas referenced throughout follow Morrone’s formal regionalisation (2015) and are reproduced in Fig. 52. For this reproduction, published maps were georeferenced in ArcGIS and used to trace biogeographic provinces (Morrone 2015) and districts (Peña 1966, O’Brien 1971, Artigas 1975). Three district-level areas in Chile’s segment of the Puna province are herein given placeholder names – Altiplano, Puna, and Northern Volcanic Cordillera – as they have not been formally described. The first two are as identified and named by Artigas (1975); the third corresponds to Artigas’ Northern Andean zone (1975), a portion of which extends into the Atacaman Province and is formally described as an Atacaman district (Morrone 2015). The Punan portion of that area contains many more active volcanoes and coincides with the Andean volcanic arc (Stern 2004); hence, “Northern Volcanic Cordillera,” to distinguish it.

Distributions of *Heteroediscelis* were compared using VIP: Vicariance Inference Program (Arias 2001), which infers vicariance events (or more generally, distributional disjunctions or barriers) by identifying allopatric sister pairs of taxa directly from distributional data (Arias et al. 2011, Hovenkamp 1997, 2001). Analysis in VIP was performed using a grid of 0.3 × 0.3 degrees without fill. Cost of distribution removal was set to 5, and a maximum overlap of 25% was allowed. The search was conducted with 1000 iterations using otherwise default settings.

## Results

### Taxonomy

#### Chilicola (Heteroediscelis)

Taxon classificationAnimaliaHymenopteraColletidae

Toro & Moldenke, 1979

Chilicola (Heteroediscelis) Toro & Moldenke, 1979: 98, 112. Michener 1992: 89. Michener 1995: 335, 337 (*partim*, as Chilicola (Oediscelis)). Michener 2002: 11. Michener 2007: 182, 184 (*partim*, as Chilicola (Oediscelis)). Packer and Genaro 2007: 41, 50. Packer 2008: 72–74, 77, 96. Almeida et al. 2008: 80–81. Moure et al. 2012. Monckton 2014: 234. Packer 2014: 254, 262. Ascher and Pickering 2015.

##### Type species.

*Chilicola mantagua* Toro & Moldenke, 1979, by original designation.

##### Diagnosis.

Males are readily diagnosable by S1 with a robust, elongate, ventrally directed process, with truncate apex having a well-defined (sometimes oblique) apical surface (other *Chilicola* that have such a process have it either spatulate as in *Chilicola rubriventris* or narrowing to a pointed apex as in some species of *Oediscelis*). In females, the combination of protibia pale at least in basal half of dorsal surface, stigmal perpendicular basal to or interstitial with first recurrent vein (extending into basal quarter of second submarginal cell in some), and thorax ≤ 1.5mm wide separates this subgenus from all but three. From these, *Heteroediscelis* females are distinguished as follows: (1) from *Unicarinicola* by at least T1–T2 with apicolateral patches of white hair (lacking on all terga in *Unicarinicola*); (2) from *Pseudiscelis* by the pronotum not elongate, with medial length of collar at most one-quarter of anterior width of collar (at least one-third in *Pseudiscelis*); (3) from *Prosopoides* by the absence of distinct and depressed facial foveae, although these are sometimes represented by a poorly delimited, not-depressed shiny area of reduced puncture density. The more mesal longitudinal depressions of the paraocular area in *Chilicola neffi* and *Chilicola packeri* partially accommodate the scape and are not homologous with facial foveae (for example, species of the subgenus *Hylaeosoma* possess both).

##### Redescription.

Small bees, length 4.2–6.8mm.

*Colouration*: Both sexes black to black-brown, margin of pronotal lobe yellow, tegula translucent yellow, wing veins brown to translucent yellow basally, tarsal segments II–IV of all legs variable from dark with apices yellow to entirely yellow, metasomal terga and sterna (T1–T6 & S1–S6 in males, T1–T5 & S1–S5 in females) with translucent yellow or amber marginal zones, wider on recurved lateral portion of terga, widened medially on sterna. Male antenna with ventral surface yellow to yellow-brown, and following parts yellow: labrum and most of mandible, markings on clypeus and lower paraocular area, and on all legs at least on femoral apex and tibial base. Female labrum variable but usually black-brown, mandible variable at least partially dark, face entirely dark (rarely with a yellow-brown or yellow spot on lower paraocular area below anterior tentorial pit, and a single specimen with a brown median spot on clypeus just above upper margin of anterior tentorial pits), ventral surface of antenna yellow or yellow-brown, and yellow on legs at least on tibial base of fore- and midleg, and following parts invariable: yellow apical ring on profemur, broader on anterior surface; yellow apicodorsal rim on mesofemur; yellow-brown apicoventral rim on coxa and trochanter of all legs.

*Pubescence*: White hairs generally short (0.5OD) and sparse, not especially plumose. Longer and denser on lower paraocular area and lateral margin of mesosomal dorsum. Mesal and lateral margins of scape with single row of long hairs forming a fringe. Genal beard longer and denser in males. Margin of pronotal lobe with short dense tomentum. Mesepisternum with long posteriorly curved hairs. Metasoma with apicolateral patches of white tomentum on at least T1–T2, apical margins of all terga otherwise with short and sparse appressed hairs and very sparse tiny setae medially. Male S1 with a few long, erect hairs basad of process, and S2 with long erect hairs dense, becoming shorter posteriorly. Female with scopae on metafemur and metatibia, corbiculate scopa on S2.

*Surface sculpture*: Microsculpture imbricate, integument generally dull; punctures generally small and deep. Mesosomal dorsum except metapostnotum densely punctate (i=0.5–1d), metapostnotum usually coarsely rugose. Legs variably puncticulate. Malar space microsculpture completely smooth, shiny, and moderately densely punctate (i=1–2d) along anterior ocular margin, becoming puncticulate-aciculate along posterior ocular margin, with dense minute punctures extending to middle of mandibular base in longer-faced bees. Male lateral surface of pronotum moderately densely punctate posteriorly (i=1–2d) densely punctate anteriorly (i≤d); in females moderately densely punctate throughout. Male metasomal T2–T5 basal depressed area coarsely microsculptured and punctures obscure; in females not coarsely microsculptured. Male metasomal sterna shallowly and sparsely punctate (i≥2d) punctures deeper and denser apicolaterally (i=1–2d); in females sparsely and minutely punctate throughout.

*Structure*: Anterior tentorial pits elongate, comma-shaped. Inner orbits of compound eye slightly convergent below. Length of pronotal collar medially ~1OD. Pronotum not elongate, at most one-quarter as long as anterior width. Probasitarsus elongate, ≥2.66 as long as maximum depth (usually ≥3). Metatibial spurs not unusually strongly sclerotized or curved. Metabasitarsus elongate, at least three times longer than maximum depth (usually ≥3.5). Stigmal perpendicular at first recurrent vein or in basal quarter of second submarginal cell. Male metafemur strongly swollen, weakly concave ventrally. Male metatibia robust and highly modified: shape approximately triangular in anterior view, with a ventral convexity leading to a rounded summit ventrally; deep longitudinal concavity on posterior surface; posterior surface with two longitudinal carinae: a ventral carina along inside of posterior concavity, and a posterior carina along lateral edge of posterior concavity (see Fig. 6B & D); apical lamina extending mediad from base of spurs, variable in shape and size, in part forming apical rim to posterior concavity. Metapostnotum well-defined, slightly depressed medially, longer than metanotum. Male S1 with a robust, elongate, ventrally directed process, with truncate apex with a well-defined and sometimes oblique apical surface.

##### Distribution.

Known from Chile, as far north as San Pedro de Atacama (Region II) and south to Cuesta Las Raíces (Region IX). Distributed throughout the Central Chilean sub-region, and found also in the Puna province (South American transition zone) and Maule province (Subantarctic sub-region); 0–3210m a.s.l.

##### Ecology.

Habitats range from *Araucaria araucana* Juss. forest to extreme desert. Recorded late August to late June, most abundant September to December.

##### Keys to the species of *Heteroediscelis*

Note: High magnification is required for some identifications; an ocular micrometer capable of distinguishing differences of 0.01mm is recommended. In frontal view, the perimeter of the median ocellus and the apex of the clypeus must be in the same plane of focus.

**Males**

**Table:** 

1	Tibiae and tarsi mostly or entirely yellow-orange (Fig. 1A)	**2**
–	Tibiae and tarsi mostly black-brown (Fig. 1B)	**3**
2(1)	Apical surface of S1 process longitudinally concave throughout (Fig. 2A); MOC equal to or greater than width of head (Fig. 2B)	***Chilicola travesia* Toro & Moldenke, 1979**
–	Apical surface of S1 process concave posteriorly, convex anteromedially (Fig. 2C); MOC less than (≤0.95×) width of head (Fig. 2D)	***Chilicola erithropoda* Toro & Moldenke, 1979**
3(1)	Frontal area with longitudinal depression above each antennal socket which partially accommodates the scape (Fig. 3A)	**4**
–	Frontal area without such depressions (Fig. 3B)	**5**
4(3)	Metabasitarsus ≤3.8× as long as maximum depth (Fig. 4A); S8 lateral process with anteromedial sclerotized margin swollen forming a convex incursion into membranous interior (Fig. 4B)	***Chilicola neffi* Toro & Moldenke, 1979**
–	Metabasitarsus ≥4× as long as maximum depth (Fig. 4C); S8 lateral process with anteromedial sclerotized margin wide but subparallel to margin throughout, not swollen (Fig. 4D)	***Chilicola packeri* Monckton, sp. n.**
5(3)	Apical surface of S1 process with distinct longitudinal median ridge, surface otherwise approximately flat (Fig. 5A)	**6**
–	Apical surface of S1 process either longitudinally concave (Fig. 5B), weakly convex (Fig. 5C), or with a median anterior convexity (Fig. 5D) but never with a longitudinal median ridge	**11**
6(5)	Malar space ≥1.4× as long as clypeal lateral (Fig. 6A); posterior metatibial carina distinctly toothed (Fig. 6B)	**7**
–	Malar space ≤1.1× as long as clypeal lateral (Fig. 6C); posterior metatibial carina lacking tooth, at most with a rounded inflection at its midlength (Fig. 6D)	**8**
7(6)	Clypeus with a distinct median longitudinal depression (Fig. 7A); malar space 2.2× as long as clypeal lateral; lower paraocular maculation not reaching lower tangent of antennal socket (Fig. 7A)	***Chilicola lickana* Monckton, sp. n.**
–	Clypeus with median longitudinal depression weak (Fig. 7B); malar space 1.4–2× as long as clypeal lateral; lower paraocular maculation just reaching lower tangent of antennal socket (Fig. 7B)	***Chilicola guanicoe* Monckton, sp. n.**
8(6)	Malar space ~0.5× as long as clypeal lateral (Fig. 8A)	***Chilicola vicugna* Toro & Moldenke, 1979**
–	Malar space ≥0.8× as long as clypeal lateral (Fig. 8B)	**9**
9(8)	Malar space longer than (1.05–1.1×) clypeal lateral (Fig. 9A); in lateral view, S1 process with anterior angle of apex acute (Fig. 9B)	***Chilicola mavida* Toro & Moldenke, 1979**
–	Malar space subequal to or shorter than (0.8–1×) clypeal lateral (Fig. 9C); in lateral view, apex of S1 process appearing broadly rounded (Fig. 9D)	**10**
10(8)	First flagellomere subequal in length and width or shorter (<1.05×) than wide (Fig. 10A); clypeus extending for half its length or more below lower ocular tangent (Fig. 10A); malar space subequal to or slightly shorter than clypeal lateral (Fig. 10B); clypeal apex yellow along entire width (Fig. 10B)	***Chilicola curvapeligrosa* Monckton, sp. n.**
–	First flagellomere ≥1.1× as long as wide (Fig. 10C); clypeus extending for less than half its length below lower ocular tangent (Fig. 10C); malar space about 0.8× as long as clypeal lateral (Fig. 10D); clypeal apex brown laterad (Fig. 10D)	***Chilicola mayu* Monckton, sp. n.**
11(5)	Malar space longer than (>1.05×) clypeal lateral (Fig. 11A); in lateral view anterior margin of S1 process strongly concave (Fig. 11B); metatibia with wide yellow basal ring (up to one fifth of tibial length) and apex yellow only in ventral half (Fig. 11C), sometimes very narrowly yellow in dorsal half	**12**
–	Malar space shorter than (<0.95×) clypeal lateral (Fig. 11D); in lateral view anterior margin of S1 process sublinear apically (Fig. 11E); metatibia more extensively pale, with wide (up to one third of tibial length) yellow basal and apical rings (Fig. 11F), sometimes yellow along entire dorsal and/or ventral surface	**15**
12(11)	Malar space very long, ≥2.7× as long as clypeal lateral (Fig. 12A); clypeus lacking median longitudinal depression (Fig. 12B); apical surface of S1 process weakly concave except for weak anterior convexity (Fig. 12C)	***Chilicola charizard* Monckton, sp. n.**
–	Malar space not as long, ≤1.6× as long as clypeal lateral (Fig. 12D); clypeus with distinct median longitudinal depression extending nearly to apex (Fig. 12E); apical surface of S1 process longitudinally concave throughout (Fig. 12F)	**13**
13(12)	Metatibia broadly expanded (≥0.5× as deep as long) and with summit of ventral convexity near to tibial apex, at approximately 0.85× tibial length (Fig. 13A); ventral metatibial carina toothed near apex and posterior carina present but weakly defined basally (Fig. 13B); malar space ≥1.2× as long as clypeal lateral (Fig. 13C)	**14**
–	Metatibia less expanded (>2× as long as maximum depth) and with summit of ventral convexity situated basad of tibial apex at approximately three-quarters of tibial length (Fig. 13D); ventral metatibial carina not toothed near apex, but with a rounded inflection near its midpoint, and posterior carina absent basally, arising at midpoint of ventral carina (Fig. 13E); malar space ≤1.1× as long as clypeal lateral (Fig. 13F)	***Chilicola katherinae* Monckton, sp. n.**
14(13)	Malar space 1.5–1.6× as long as clypeal lateral (Fig. 14A); in frontal view, lower ocular tangent passing above or just crossing upper margin of anterior tentorial pits (Fig. 14B)	*Chilicola diaguita* Toro & Moldenke, 1979
–	Malar space 1.2–1.4× as long as clypeal lateral (Fig. 14C); in frontal view, lower ocular tangent passing through anterior tentorial pits (Fig. 14D)	***Chilicola vina* Toro & Moldenke, 1979**
15(11)	In lateral view, truncate apex of S1 process perpendicular to axis of process, anterior and posterior margins parallel apically (Fig. 15A); metatibia broadly expanded (≥0.5× as deep as long) and with summit of ventral convexity near to tibial apex, at approximately 0.85× tibial length (Fig. 15B); ventral metatibial carina toothed near apex and posterior carina weakly defined basally (Fig. 15C)	***Chilicola randolphi* Monckton, sp. n.**
–	In lateral view, truncate apex of S1 process slightly oblique, anterior and posterior margins convergent apically (Fig. 15D); metatibia less expanded (≥2.1× as long as maximum depth) and with summit of ventral convexity situated basad of tibial apex at approximately two-thirds of tibial length (Fig. 15E); ventral metatibial carina not toothed near apex, but with a rounded inflection near its midpoint, and posterior carina absent basally, arising at or beyond midpoint of ventral carina (Fig. 15F)	**16**
16(15)	Mesobasitarsus mostly yellow (Fig. 16A); second flagellomere >1.1 l×onger than first (Fig. 16B); malar space <0.5× as long as clypeal lateral (Fig. 16C)	***Chilicola deserticola* Toro & Moldenke, 1979**
–	Mesobasitarsus mostly dark (Fig. 16D); second flagellomere subequal to first in length (Fig. 16E); malar space >0.5× as long as clypeal lateral (Fig. 16F)	***Chilicola mantagua* Toro & Moldenke, 1979**

**Females**

**Table:** 

1	Protibia with yellow on dorsal surface present in basal half only, apical half dark (Fig. 17A)	**2**
–	Protibia with yellow along entire dorsal surface (Fig. 17B; slightly darkened to yellow-brown apically in *Chilicola mantagua*, Fig. 17C)	**5**
2(1)	Malar space at most 0.8× as long as clypeal lateral (Fig. 18A); pedicel and first flagellomere yellow-brown apicoventrally (Fig. 18B); metasoma mostly orange-brown (Fig. 18C); T3 with apicolateral patches of tomentum as on T1–T2 (Fig. 18C)	***Chilicola randolphi* Monckton, sp. n.**
–	Malar space at least as long as clypeal lateral (Fig. 18D); pedicel and first flagellomere dark, at most with apicoventral margins brown (Fig. 18E); metasoma black-brown (Fig. 18F); T3 with apicolateral hair band sparse, not tomentose as on T1–T2 (Fig. 18F)	**3**
3(2)	In frontal view, lower ocular tangent passing just tangent to or above upper margin of anterior tentorial pits (Fig. 19A)	***Chilicola diaguita* Toro & Moldenke, 1979**
–	In frontal view, lower ocular tangent passing through anterior tentorial pits (Fig. 19B)	**4**
4(3)	Malar space ≤1.1× as long as clypeal lateral (Fig. 20A)	***Chilicola katherinae* Monckton, sp. n.**
–	Malar space ≥1.2× as long as clypeal lateral (Fig. 20B)	***Chilicola vina* Toro & Moldenke, 1979**
5(1)	Malar space clearly longer than (>1.1×) clypeal lateral (Fig. 21A)	**6**
–	Malar space at most subequal in length to clypeal lateral (Fig. 21B)	**8**
6(5)	Malar space less than <1.7× as long as clypeal lateral (Fig. 21A)	***Chilicola guanicoe* Monckton, sp. n.**
–	Malar space at least twice as long as clypeal lateral (Fig. 22B, D)	**7**
7(6)	Metatibia with wide yellow basal and apical rings (Fig. 22A); malar space approximately 2× as long as clypeal lateral (Fig. 22B), but shorter than width of genal area in lateral view.	***Chilicola lickana* Monckton, sp. n.**
–	Metatibia entirely dark, or with a wide basal band of brown that is clearly darker than the pale colouration on other legs (Fig. 22C); malar space >2× as long as clypeal lateral (Fig. 22D), and longer than width of genal area in lateral view	***Chilicola charizard* Monckton, sp. n.**
8(5)	Malar space approximately 0.9× as long as, or subequal in length to, clypeal lateral (Fig. 23A)	**9**
–	Malar space at most two-thirds as long as clypeal lateral (Fig. 23B)	**10**
9(8)	Apex of metafemur and base of metatibia yellow-brown to brown, distinctly darker than same parts of midleg (Fig. 24A); subantennal sutures ≥1.1× longer than the shortest distance between them (Fig. 24B)	***Chilicola curvapeligrosa* Monckton, sp. n.**
–	Apex of metafemur and base of metatibia yellow, as on same parts of midleg (Fig. 24C); subantennal sutures at most as long as the shortest distance between them (Fig. 24D)	***Chilicola mavida* Toro & Moldenke, 1979**
10(8)	Frontal area with longitudinal depressions which partially accommodate the scape (Fig. 25A)	**11**
–	Frontal area without such depressions (Fig. 25B)	**12**
11(10)	Metasoma with at least T1–T3 orange-brown, except for dark premarginal line and/or a pair of lateral dark spots (Fig. 26A); metabasitarsus narrower, ≥4× as long as maximum depth (Fig. 26B)	***Chilicola packeri* Monckton, sp. n.**
–	Metasoma darker, at most T1–T3 orange-brown with apical half of disc dark (Fig. 26C); metabasitarsus broader, ≤4× as long as maximum depth (Fig. 26D)	***Chilicola neffi* Toro & Moldenke, 1979**
12(10)	T3 with apicolateral hair band sparse, not tomentose as on T1–T2 (Fig. 27A); clypeus more sparsely punctate than supraclypeal area (Fig. 27B)	**13**
–	T3 with apicolateral patches of tomentum as on T1–T2 (Fig. 27C); clypeus at least as densely punctate as supraclypeal area (Fig. 27D)	**14**
13(12)	Clypeus strongly convex in dorsal half, bulging ~2OD above compound eye in lateral view (Fig. 28A); apex of metafemur and base of metatibia brown, distinctly darker than same parts of midleg (Fig. 28B)	***Chilicola mayu* Monckton, sp. n.**
–	Clypeus not as above, dorsal half bulging ≤OD above compound eye in lateral view (Fig. 28C); apex of metafemur and base of metatibia yellow, as on same parts of midleg (Fig. 28D)	***Chilicola vicugna* Toro & Moldenke, 1979**
14(12)	Metatibia somewhat expanded, >0.25× as deep as long (Fig. 29A); legs with following parts yellow as on protibia: mesotibia with at least a wide apical ring, apicodorsal rim on metafemur, metatibia with wide basal ring and narrow to wide apical ring, apical half of metadistitarsus (Fig. 29B)	**15**
–	Metatibia not expanded, and >4.1× as long as maximum depth (Fig. 29C); legs less extensively yellow: apex of mesotibia at most narrowly yellow, metafemur with at most a narrow yellow-brown apical ring, metatibia with at most a wide yellow basal ring and posterior apical rim yellow-brown (Fig. 29D)	**16**
15(14)	Metatibia mostly yellow, at least in basal one-third and apical quarter, often entirely so (Fig. 30A); probasitarsus entirely yellow, at most suffused with yellow-brown at midlength of posterior surface (Fig. 30B)	***Chilicola travesia* Toro & Moldenke, 1979**
–	Metatibia mostly dark, at most basal one-third and a narrow yellow apical ring (Fig. 30C); probasitarsus darker, only basal and apical quarters as pale as yellow on protibia (Fig. 30D)	***Chilicola erithropoda* Toro & Moldenke, 1979**
16(14)	Metapostnotum with sparse, irregular longitudinal carinae (Fig. 31A); protibia with dorsal surface darkened to yellow-brown apically (Fig. 31B); meso- and metabasitarsi almost entirely dark; mesodistitarsus brown in apical one-third; apex of metatibia black-brown (Fig. 31C)	***Chilicola mantagua* Toro & Moldenke, 1979**
–	Metapostnotum rugose, often more weakly so posteriorly (Fig. 31D); protibia with dorsal surface yellow for entire length (Fig. 31E); meso- and metabasitarsi yellow or yellow-brown ventrally; mesodistitarsus yellow or yellow-brown in apical half; posterior apical rim of metatibia brown or yellow-brown (Fig. 31F)	***Chilicola deserticola* Toro & Moldenke, 1979**

**Figure 1.**
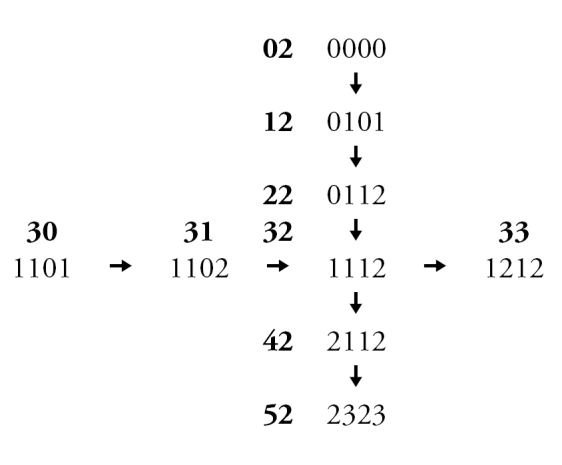
Male, leg colouration: **A** *Chilicola travesia*, tibiae and tarsi entirely yellow-orange **B** *Chilicola mantagua*, tibiae and tarsi mostly black-brown; scale bars 0.75 mm.

**Figure 2.**
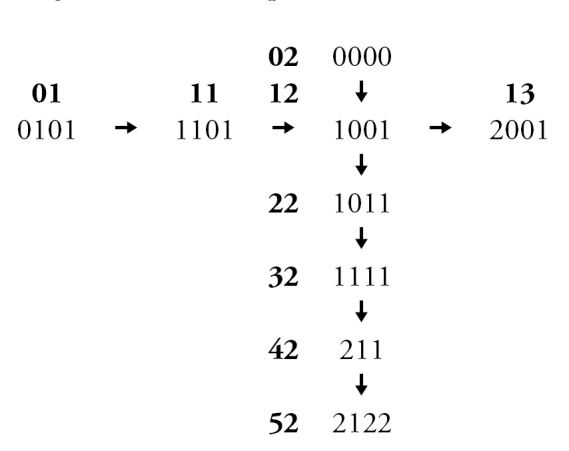
Male: **A** *Chilicola travesia*, apical surface of S1 process longitudinally concave throughout, scale bar 0.15 mm **B** *Chilicola travesia*, frontal view, MOC equal to or greater than width of head, scale bar 0.5 mm **C** *Chilicola erithropoda*, apical surface of S1 process with anteromedial convexity, scale bar 0.15 mm **D** *Chilicola erithropoda*, frontal view, MOC less than width of head, scale bar 0.5 mm.

**Figure 3.**
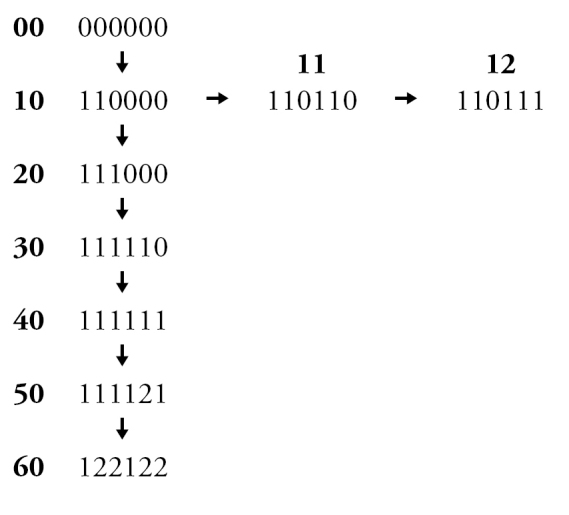
Male, frontal view: **A** *Chilicola neffi*, arrow indicates longitudinal depression of frontal area **B** *Chilicola vicugna*, longitudinal depressions absent; scale bars 0.25 mm.

**Figure 4.**
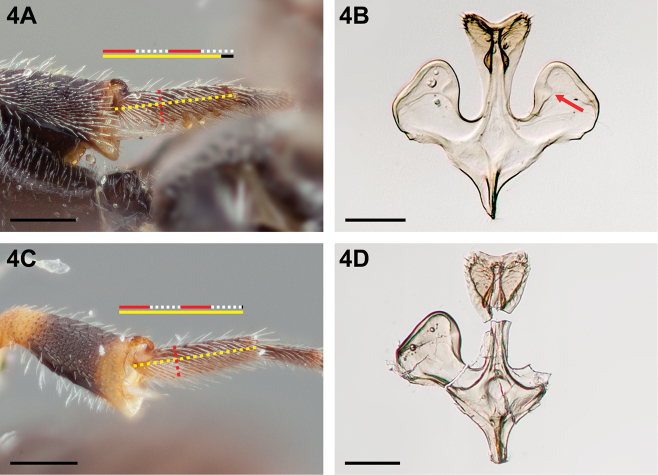
Male: **A** *Chilicola neffi*, metabasitarsus^†^ ≤3.8× as long as maximum depth, scale bar 0.25 mm **B** *Chilicola neffi*, S8, arrow indicates convex incursion of anteromedial sclerotized margin into lateral process, scale bar 0.15 mm **C** *Chilicola packeri*, metabasitarsus ≥4× as long as maximum depth, scale bar 0.25 mm **D** *Chilicola packeri*, S8, lateral process with anteromedial sclerotized margin wide but subparallel to margin throughout scale bar 0.15 mm.

**Figure 5.**
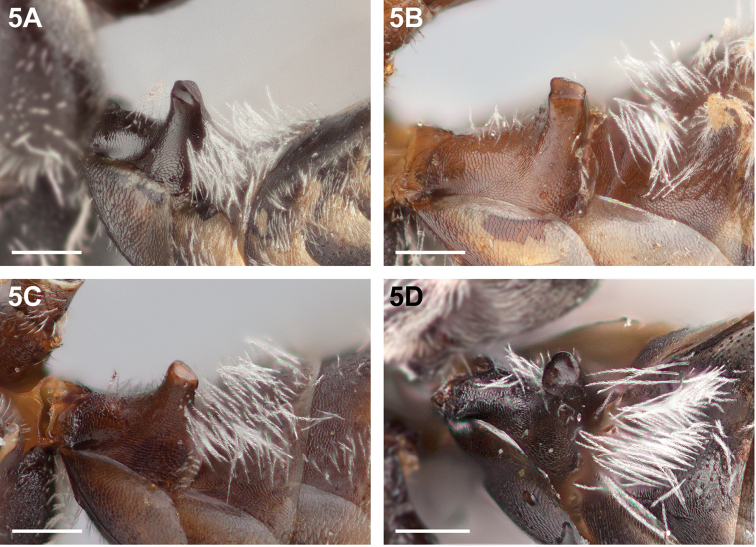
Male S1, apical surface: **A** *Chilicola mavida*, with longitudinal median ridge **B** *Chilicola randolphi*, longitudinally concave **C** *Chilicola neffi*, weakly convex **D** *Chilicola charizard*, with median anterior convexity; scale bars 0.25 mm.

**Figure 6.**
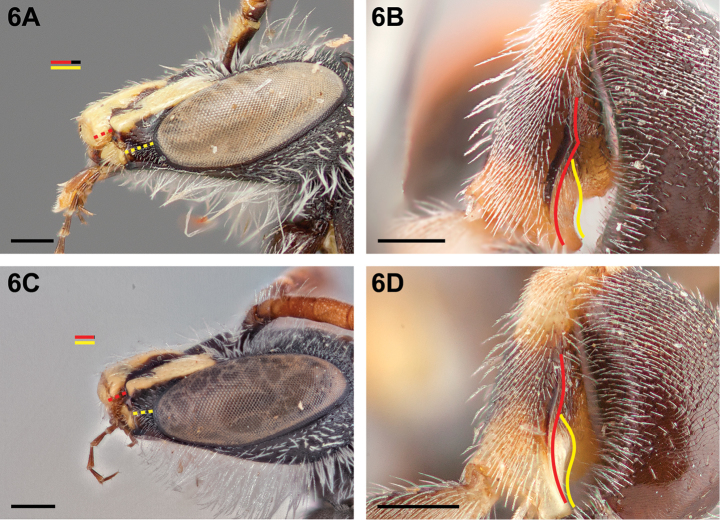
Male: **A** *Chilicola guanicoe*, malar space ≥1.4× as long as clypeal lateral **B** *Chilicola lickana*, metatibia, posterior view, toothed posterior carina indicated by red line, ventral carina indicated by yellow line **C** *Chilicola mavida*, malar space ≤1.1× as long as clypeal lateral **C** *Chilicola vicugna*, metatibia, posterior view, untoothed posterior carina indicated by red line, ventral carina indicated by yellow line; scale bars 0.25 mm.

**Figure 7.**
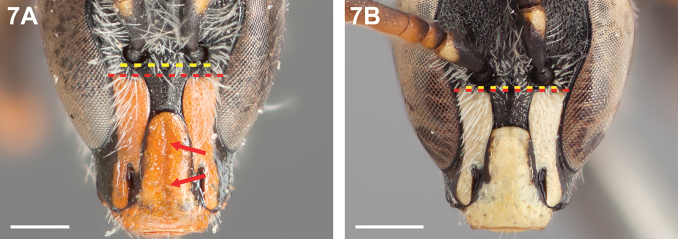
Male, frontal view: **A** *Chilicola lickana*, arrows indicate median longitudinal depression of clypeus, lower paraocular maculation does not reach lower tangent of antennal socket **B** *Chilicola guanicoe*, clypeus without median longitudinal depression, lower paraocular maculation just reaches lower tangent of antennal socket; scale bars 0.5 mm.

**Figure 8.**
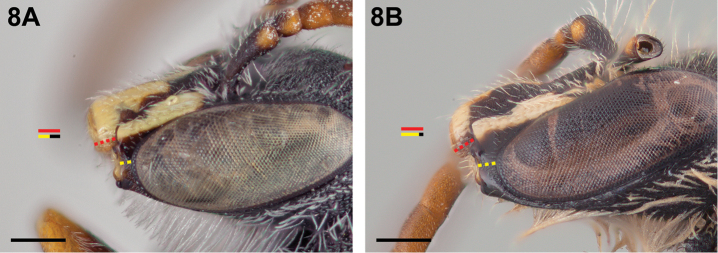
Male: **A** *Chilicola vicugna*, malar space ~0.5× as long as clypeal lateral **B** *Chilicola mayu*, malar space ≥0.8× as long as clypeal lateral; scale bars 0.25 mm

**Figure 9.**
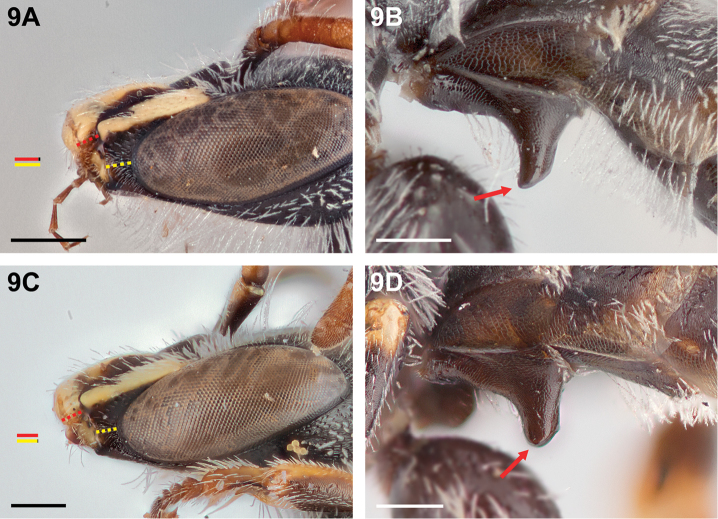
Male: **A** *Chilicola mavida*, malar space longer than clypeal lateral **B** *Chilicola mavida*, S1 process^†^, lateral view with arrow indicating acute anterior apical angle **C** *Chilicola curvapeligrosa*, malar space subequal to clypeal lateral **D** *Chilicola curvapeligrosa*, S1 process^†^, lateral view with arrow indicating rounded apex; scale bars 0.25 mm.

**Figure 10.**
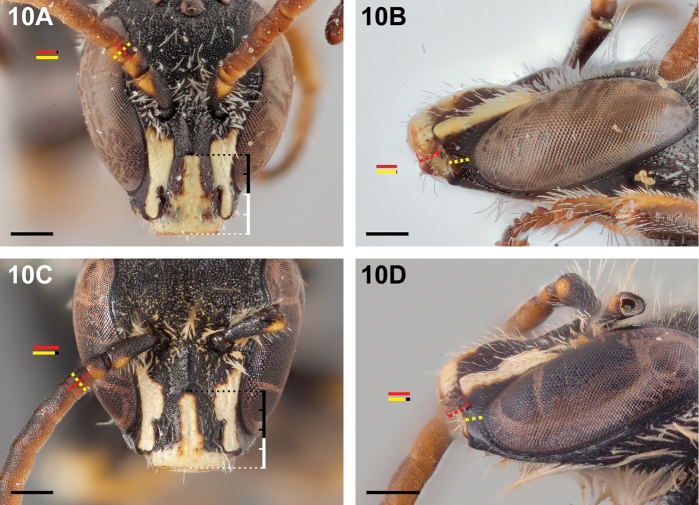
Male: **A** *Chilicola curvapeligrosa*, frontal view, F1 shorter than wide, clypeus extending for more than half its length below LOT (white portion of bracket) **B** *Chilicola curvapeligrosa*, malar space subequal to clypeal lateral **C** *Chilicola mayu*, frontal view, F1 ~1.1× as long as wide, clypeus extending for less than half its length below LOT (white portion of bracket) **D** *Chilicola mayu*, malar space ~0.8× as long as clypeal lateral; scale bars 0.25 mm.

**Figure 11.**
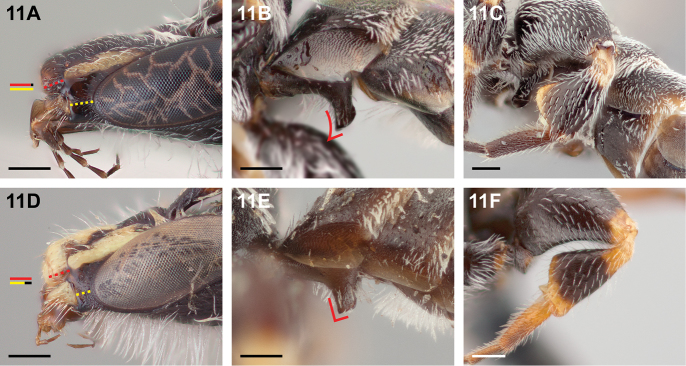
Male: **A** *Chilicola katherinae*, malar space longer than clypeal lateral **B** *Chilicola charizard*, S1 process, lateral view showing strongly concave anterior margin **C** *Chilicola charizard*, metatibia with wide yellow basal ring and apex yellow only basally (C.chzd.005) **D** *Chilicola randolphi*, malar space shorter than clypeal lateral **E** *Chilicola deserticola*, S1 process^†^, lateral view showing sublinear anterior margin **F** *Chilicola deserticola*, metatibia with basal third yellow and wide yellow apical ring; scale bars 0.25 mm.

**Figure 12.**
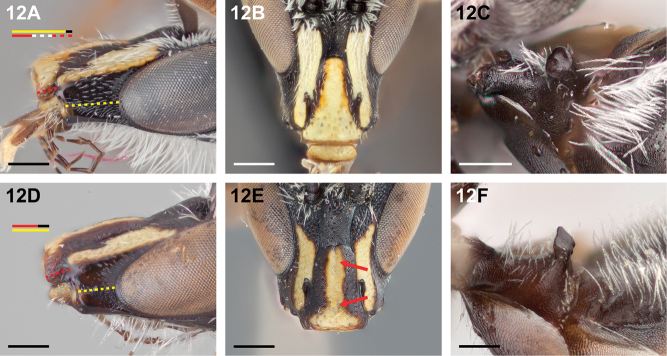
Male: **A** *Chilicola charizard*, malar space ~2.7× as long as clypeal lateral **B** *Chilicola charizard*, clypeus without median longitudinal depression **C** *Chilicola charizard*, S1 apical surface with median anterior convexity (C.chzd.005) **D** *Chilicola diaguita*, malar space ~1.5× as long as clypeal lateral **E** *Chilicola diaguita*, arrows indicate median longitudinal depression **F** *Chilicola diaguita*, S1 apical surface longitudinally concave; scale bars 0.25 mm.

**Figure 13.**
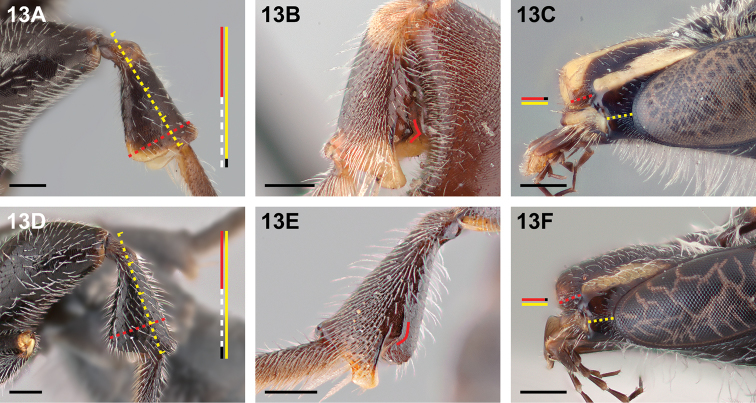
Male: **A** *Chilicola diaguita*, metatibia ≥0.5× as deep as long **B** *Chilicola vina*, ventral metatibial carina toothed near apex, posterior carina present but weakly defined basally **C** *Chilicola vina*, malar space ~1.2× as long as clypeal lateral **D** *Chilicola katherinae*, metatibia >2× as long as maximum depth **E** *Chilicola katherinae*, ventral metatibial carina with rounded inflection near apex, posterior carina absent basally **F** *Chilicola katherinae*, malar space ~1.1× as long as clypeal lateral; scale bars 0.25 mm.

**Figure 14.**
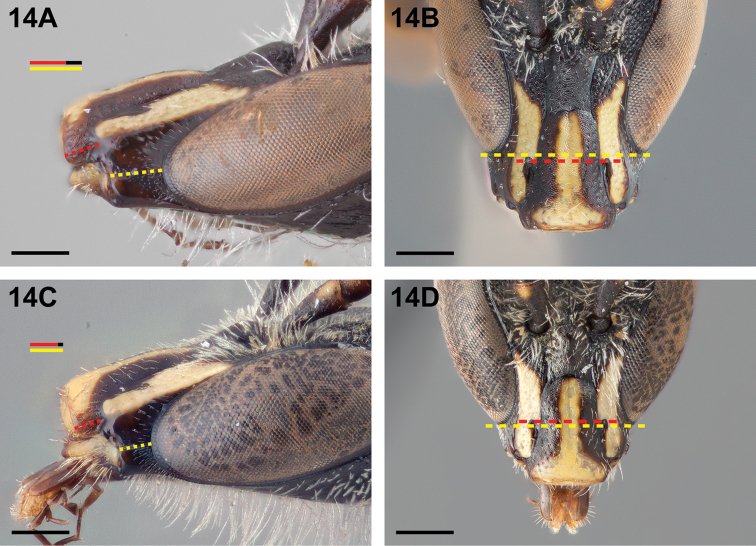
Male: **A** *Chilicola diaguita*, malar space ~1.5× as long as clypeal lateral **B** *Chilicola diaguita*, frontal view, LOT above anterior tentorial pits **C** *Chilicola vina*, malar space ~1.2× as long as clypeal lateral **D** *Chilicola vina*, frontal view, LOT passing through anterior tentorial pits; scale bars 0.25 mm.

**Figure 15.**
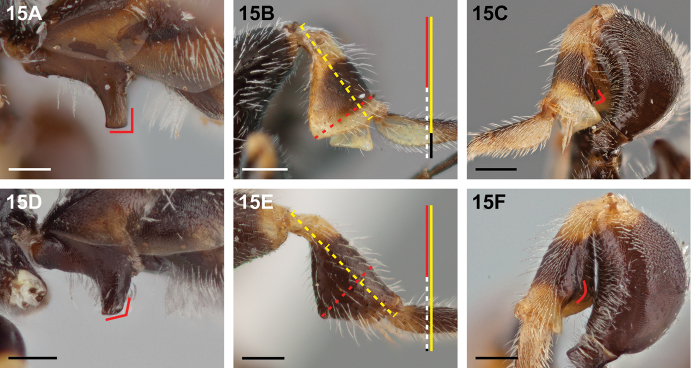
Male: **A** *Chilicola randolphi*, S1 process^†^, lateral view showing perpendicular truncate apex and parallel margins **B** *Chilicola randolphi*, metatibia >0.5× as deep as long, summit of ventral convexity at ~0.85× tibial length **C** *Chilicola randolphi*, ventral metatibial carina toothed near apex, posterior carina present but weakly defined basally **D** *Chilicola mantagua*, S1 process^†^, lateral view showing oblique apex and apically convergent margins **E** *Chilicola mantagua*, metatibia ~2.1× as long as maximum depth, summit of ventral convexity at two-thirds tibial length **F** *Chilicola deserticola*, ventral metatibial carina with rounded inflection near apex, posterior carina absent basally; scale bars 0.25 mm.

**Figure 16.**
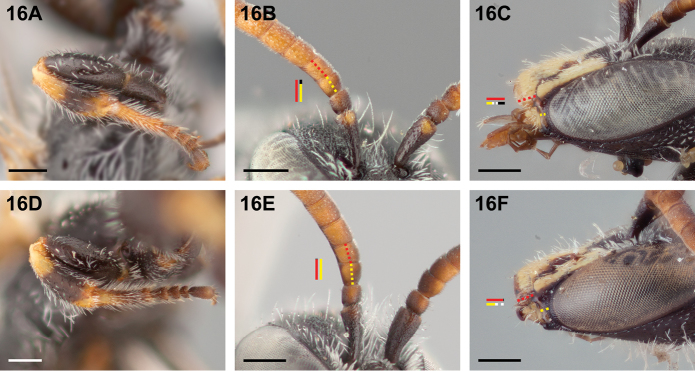
Male: **A** *Chilicola deserticola*, mesobasitarsus mostly yellow **B** *Chilicola deserticola*, F2 longer than F1 **C** *Chilicola deserticola*, malar space ~0.33× as long as clypeal lateral **D** *Chilicola mantagua*, mesobasitarsus mostly dark **E** *Chilicola mantagua*, F2 subequal in length to F1 **F** *Chilicola mantagua*, malar space >0.5× as long as clypeal lateral; scale bars 0.25 mm.

**Figure 17.**
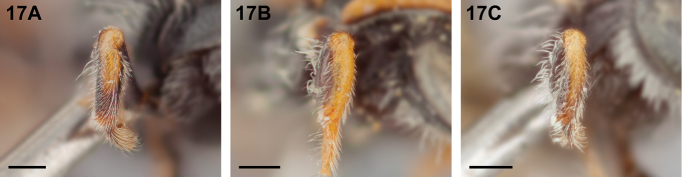
Female, dorsal surface of protibia: **A** *Chilicola randolphi*, yellow in basal half only **B** *Chilicola deserticola*, yellow along entire length **C** *Chilicola mantagua*, yellow along entire length except darkened to yellow-brown apically; scale bars 0.25 mm.

**Figure 18.**
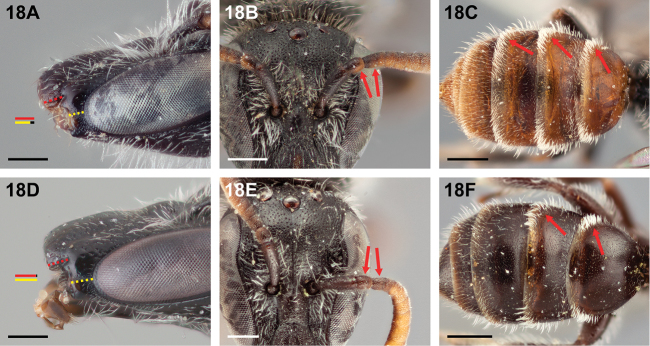
Female: **A** *Chilicola randolphi*, malar space ~0.8× as long as clypeal lateral, scale bar 0.25 mm **B** *Chilicola randolphi*, frontal view, pedicel and F1 yellow-brown apicoventrally as indicated by arrows, scale bar 0.25 mm **C** *Chilicola randolphi*, metasoma orange-brown, T1-T3 with apicolateral patches of tomentum, scale bar 0.5 mm **D** *Chilicola katherinae*, malar space ~1.1× as long as clypeal lateral, scale bar 0.25 mm **E** *Chilicola vina*, frontal view, pedicel and F1 dark throughout, as indicated by arrows, scale bar 0.25 mm **F** *Chilicola vina*, metasoma black-brown, T3 apicolateral hair band sparse, scale bar 0.5 mm.

**Figure 19.**
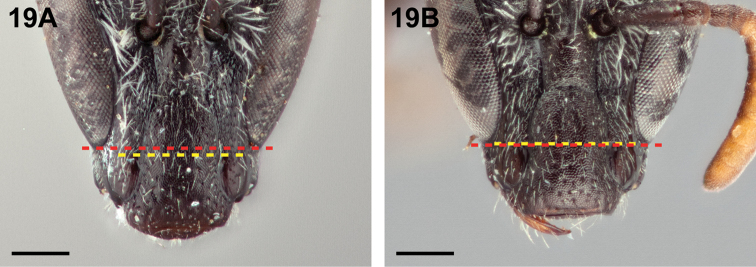
Female, frontal view: **A** *Chilicola diaguita*, LOT above anterior tentorial pits **B** *Chilicola vina*, LOT passing through anterior tentorial pits; scale bars 0.25 mm.

**Figure 20.**
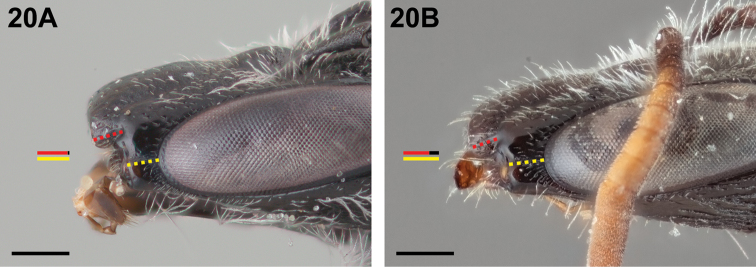
Female: **A** *Chilicola katherinae*, malar space ~1.1× as long as clypeal lateral **B** *Chilicola vina*, malar space ~1.3× as long as clypeal lateral; scale bars 0.25 mm.

**Figure 21.**
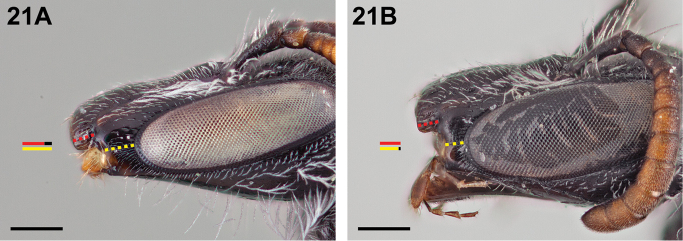
Female: **A** *Chilicola guanicoe*, malar space ~1.33× as long as clypeal lateral **B** *Chilicola mavida*, malar space ~0.9× as long as clypeal lateral; scale bars 0.25 mm.

**Figure 22.**
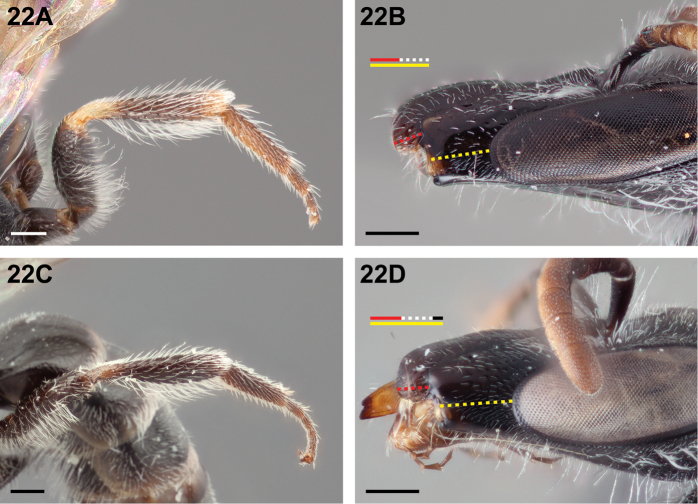
Female: **A** *Chilicola lickana*, metatibia with wide yellow basal and apical rings **B** *Chilicola lickana*, malar space ~2× as long as clypeal lateral **C** *Chilicola charizard*, metatibia with wide brown basal ring, otherwise black-brown **D** *Chilicola charizard*, malar space ~2.33× as long as clypeal lateral; scale bars 0.25 mm.

**Figure 23.**
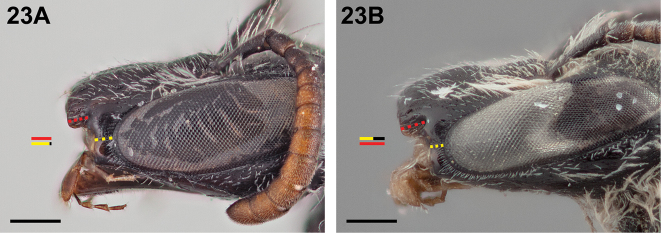
Female: **A** *Chilicola mavida*, malar space ~0.9× as long as clypeal lateral **B** *Chilicola mayu*, malar space ~0.5× as long as clypeal lateral; scale bars 0.25 mm.

**Figure 24.**
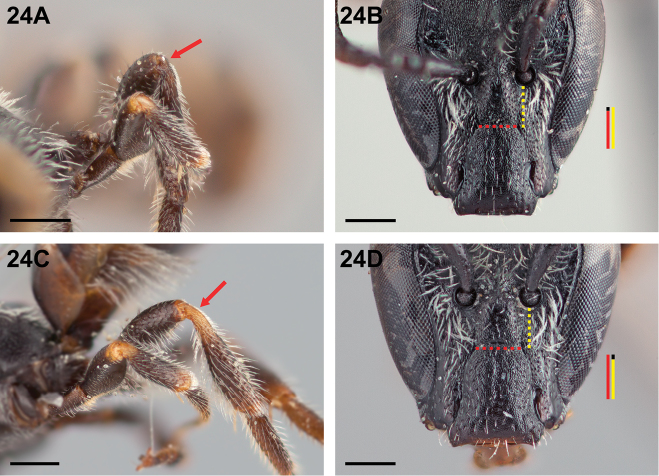
Female: **A** *Chilicola curvapeligrosa*, apex of metafemur and base of metatibia darker than same parts on midleg, as indicated by arrow, scale bar 0.5 mm **B** *Chilicola curvapeligrosa*, frontal view, subantennal sutures ~1.1× as long as shortest distance between them, scale bar 0.25 mm **C** *Chilicola mavida*, apex of metafemur and base of metatibia yellow as on same parts of midleg, as indicated by arrow, scale bar 0.5 mm **D** *Chilicola mavida*, frontal view, subantennal sutures ~0.9× as long as shortest distance between them, scale bar 0.25 mm.

**Figure 25.**
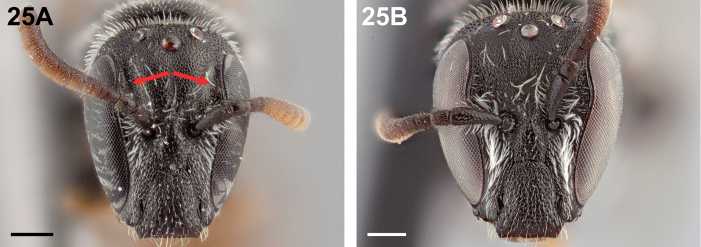
Female, frontal view: **A** *Chilicola neffi*, arrows indicate longitudinal depressions of frontal area **B** *Chilicola mantagua*, longitudinal depressions absent; scale bars 0.25 mm.

**Figure 26.**
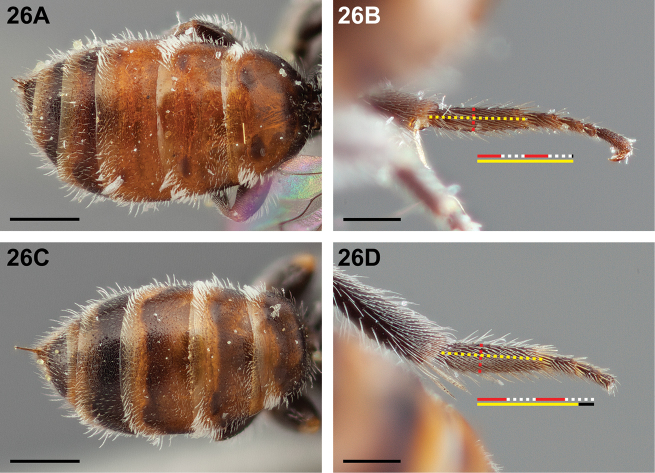
Female: **A** *Chilicola packeri*, metasoma, T1-T3 orange-brown except for paired lateral dark spots, scale bar 0.5 mm **B** *Chilicola packeri*, metabasitarsus >4× as long as maximum depth, scale bar 0.25 mm **C** *Chilicola neffi*, metasoma, T1-T3 partly orange-brown with apical half of disc dark, scale bar 0.5 mm **D** *Chilicola neffi*, metabasitarsus ~3.3× as long as maximum depth, scale bar 0.25 mm.

**Figure 27.**
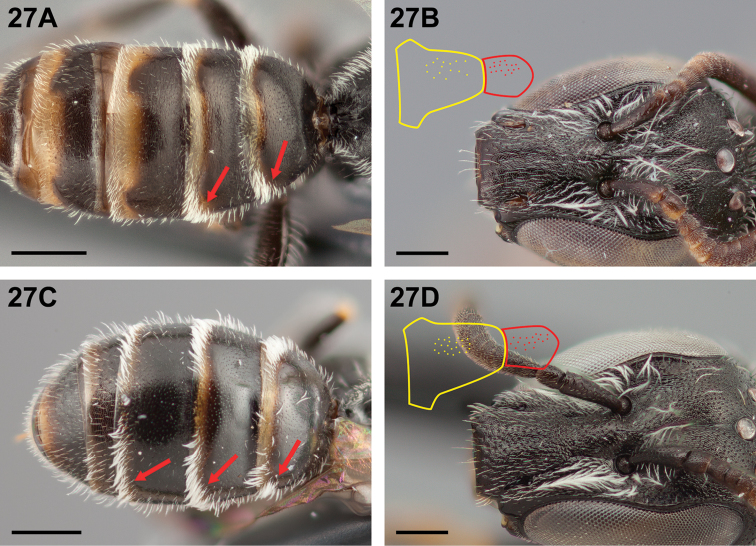
Female: **A** *Chilicola vicugna*, metasoma, T3 apicolateral hair band sparse, scale bar 0.5 mm **B** *Chilicola vicugna*, frontal view^†^, clypeus more sparsely punctate than sypraclypeal area, as indicated by select areas of punctation reproduced in yellow and red respectively, scale bar 0.25 mm **C** *Chilicola mantagua*, metasoma, T1-T3 with apicolateral patches of tomentum, scale bar 0.5 mm **D** *Chilicola mantagua*, frontal view, clypeus as densely punctate as supraclypeal area, as indicated by select areas of punctation reproduced in yellow and red respectively, scale bar 0.25 mm.

**Figure 28.**
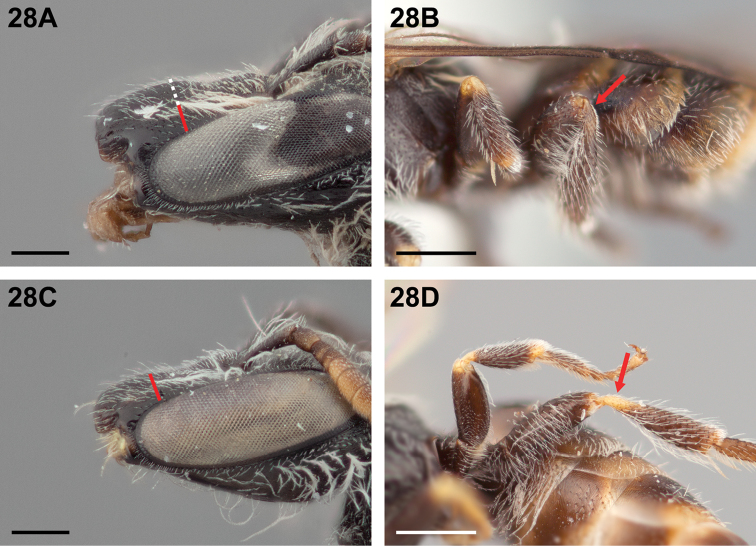
Female: **A** *Chilicola mayu*, clypeus bulging ~2OD above compound eye in lateral view, scale bar 0.25 mm **B** *Chilicola mayu*, apex of metafemur and base of metatibia darker than same parts on midleg, scale bar 0.5 mm (C.mayu.005) **C** *Chilicola vicugna*, clypeus bulging ~OD above compound eye in lateral view, scale bar 0.25 mm **D** *Chilicola vicugna*, apex of metafemur and base of metatibia yellow as on same parts of midleg, scale bar 0.5 mm.

**Figure 29.**
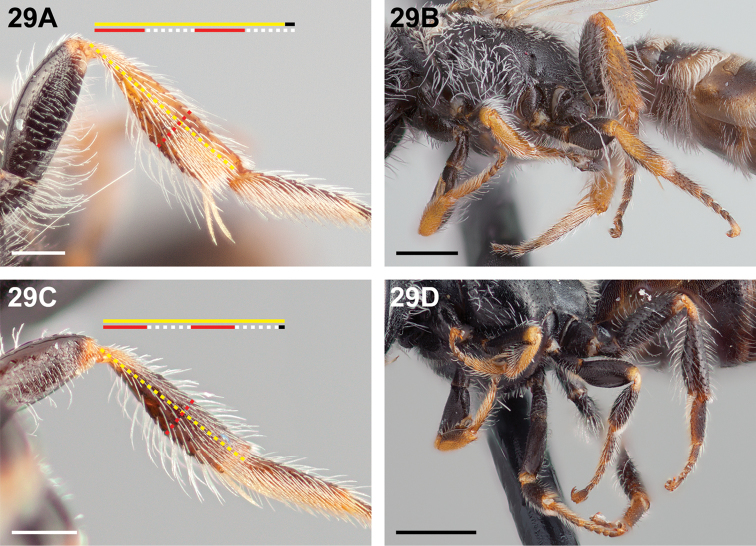
Female: **A** *Chilicola travesia*, metatibia >0.25× as deep as long, scale bar 0.25 mm **B** *Chilicola travesia*, legs with extensive yellow colouration, scale bar 0.5 mm **C** *Chilicola deserticola*, metatibia ~4.1× as long as maximum depth, scale bar 0.25 mm **D** *Chilicola deserticola*, legs less extensively yellow, scale bar 0.5 mm.

**Figure 30.**
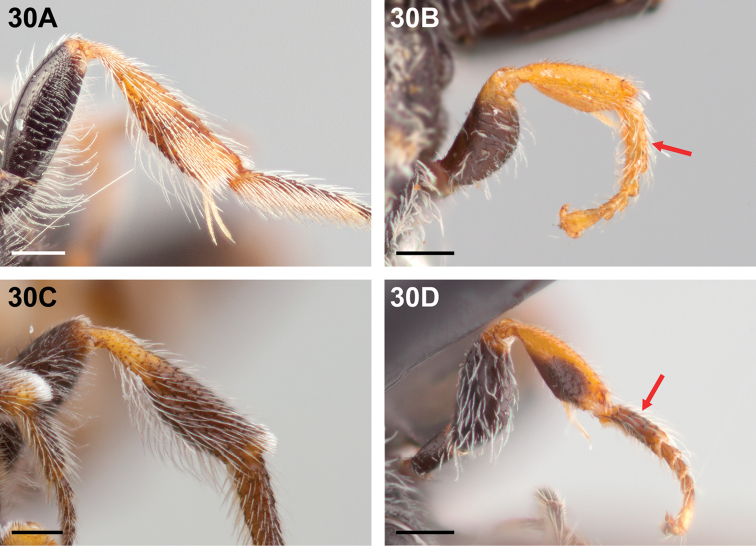
Female: **A** *Chilicola travesia*, metatibia yellow in basal half and apical third **B** *Chilicola travesia*, probasitarsus entirely yellow as indicated by arrow **C** *Chilicola erithropoda*, metatibia yellow in basal third and with narrow yellow apical ring **D** *Chilicola erithropoda*, probasitarsus with only basal and apical quarters yellow, midlength brown as indicated by arrow; scale bars 0.25 mm.

**Figure 31.**
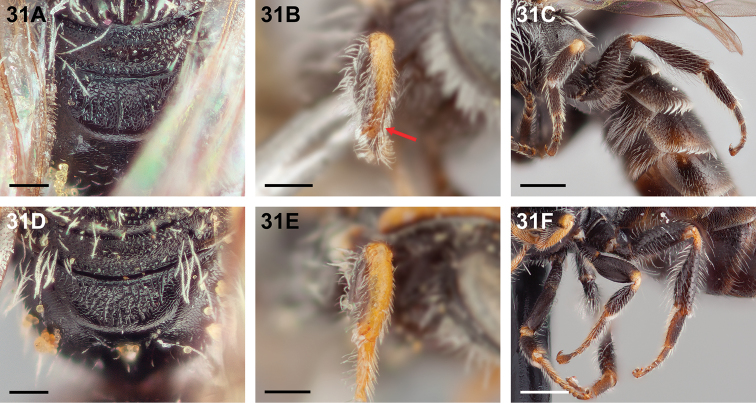
Female: **A** *Chilicola mantagua*, metapostnotum with sparse, irregular longitudinal carinae, scale bar 0.15 mm **B** *Chilicola mantagua*, protibia dorsal surface darkened to yellow-brown apically, scale bar 0.25 mm **C** *Chilicola mantagua*, meso- and metabasitarsi almost entirely dark, mesodistitarsus brown apically, apex of metatibia black-brown, scale bar 0.5 mm **D** *Chilicola deserticola*, metapostnotum rugose, scale bar 0.15 mm **E** *Chilicola deserticola*, protibia dorsal surface yellow along entire length, scale bar 0.25 mm **F** *Chilicola deserticola*, meso- and metabasitarsi yellow-brown ventrally, mesodistitarsus yellow-brown apically, metatibia with posterior apical rim yellow-brown, scale bar 0.5 mm.

#### Species descriptions

##### Chilicola (Heteroediscelis) mantagua

Taxon classificationAnimaliaHymenopteraColletidae

Toro & Moldenke, 1979

Figs 1B ; 15D–E ; 16D–F ; 17C ; 25B ; 27C–D ; 31A–C ; 32A–J ; 49

Chilicola mantagua Toro & Moldenke, 1979: 114–116 (male holotype, female allotype, AMNH [examined]). Aravena and Toro 1985: 177, 179–183; figs 6, 13, 29 (illustration, morphology). Michener 1995: 337. Michener 2007: 184. Almeida et al. 2008: 76 (phylogeny). Packer 2008: 74, 77–78 (phylogeny). Montalva and Ruz 2010: 28 (checklist). Moure et al. 2012 (catalogue). Ascher and Pickering 2015 (checklist).
Chilicola valparaiso Toro & Moldenke, 1979: 117–118 (male holotype, AMNH [examined]). Packer 2008: 77. Montalva and Ruz 2010: 29 (checklist). Moure et al. 2012 (catalogue). Ascher and Pickering 2015 (checklist). **Syn. n.**

###### Diagnosis.

Males are diagnosable by the combination of malar space short (~0.6× clypeal lateral), mesobasitarsus mostly dark, and metatibia with the combination of ventral carina extending full length of longitudinal concavity and smooth, posterior carina absent basally and originating in apical half of ventral carina, and apical lamina reduced (~0.5OD in length). Females are diagnosable by the combination of the malar space short (~0.5× clypeal lateral), metapostnotum with sparse, irregular longitudinal carinae, and protibia yellow along entire dorsal surface but darkened somewhat to yellow-brown apically. Among consubgeners *Chilicola deserticola* is most similar, but can be differentiated in both sexes by the shorter malar space (<0.5× clypeal lateral) and by the characters given in the key.

###### Description.

**Male.** Length 4.8–5.5mm, forewing length 3.0–3.3mm, head width 1.1–1.3mm, thorax width 1.1–1.4mm, median ocellar diameter (OD) 0.11–0.12mm.

*Colouration*: Black-brown except as follows: antenna variable (scape with yellow apicoventral spot to entirely dark; apicoventral rim of pedicel brown to entirely dark; ventral surface of flagellum yellow to yellow-brown, narrowing basally on F1, remaining flagellomeres suffused with brown basally). Following parts yellow: labrum; clypeus except narrowly to broadly dark along epistomal suture and apex usually brown laterad; lower paraocular area extending ~1OD above transverse portion of epistomal suture and not reaching lower tangent of antennal socket; protrochanter apical rim posteriorly; apex of profemur broadly on anterior surface, narrowly on posterior surface; protibia except dark medioventrally; probasitarsus, at least on dorsal half; prodistitarsus, at least on apical two-thirds; narrow apical ring on mesofemur; wide basal ring and narrow apical ring on mesotibia, the latter wider anteriorly; apical ring on metafemur, broad dorsally; broad basal and apical rings on metatibia, excluding apical margin of ventral convexity; metabasitarsus in basal quarter dorsally, extending to apex ventrally; anterior spot on tegula. Apicoventral rim of metacoxa yellow-brown. Ventral metatibial carina black. Metasoma dark brown, T1–T6 marginal zones translucent yellow.

*Pubescence*: White, hairs generally short (0.5OD) and sparse, not especially plumose; on lower paraocular area and around antennal base moderately long and moderately dense (1OD); genal beard long and dense (0.5–2OD) longest at midlength; discs of mesoscutum, scutellum, and metanotum mostly bare, few short hairs (0.5OD), denser and as long or longer toward lateral margin as follows: mesoscutum (0.5OD), scutellum (1–2OD) also on posterior margin, longest posteromedially, metanotum (≤2OD); propodeal hairs moderately long and dense dorsolaterally (0.5–1.5OD); T1–T3 apicolateral patches of tomentum (0.5–1OD); S2 hairs long and dense (1.5OD basolaterad, 0.5OD distilaterad, shorter mesad).

*Surface sculpture*: Microsculpture imbricate, integument generally dull; punctures generally small and deep. Clypeus and supraclypeal, lower paraocular, and vertexal areas moderately densely punctate (i=1–2d) except supraclypeal area more densely punctate toward lateral margin (i≤d); frontal area punctate (i≤d); ocellocular space moderately densely punctate adjacent to compound eye (i=1–2d) becoming impunctate adjacent to lateral ocellus; scape shallowly and moderately densely punctate (i=1–2d) densely punctate apicoventrally (i≤d); genal area coarsely imbricate to weakly microstriate and moderately densely punctate (i=1–2d); hypostomal area weakly imbricate and densely punctate (i=d); pronotum densely punctate (i=0.5–1d); mesoscutum and scutellum moderately densely punctate (i=1–2d) punctures crowded around median (i≤d); mesepisternum irregularly punctate, sparse below scrobe and anterior of episternal groove (i=1–3d), impunctate just dorsad of scrobe, otherwise dense on hypoepimeral area (i≤d); metanotum moderately densely punctate (i=1–2d) punctures crowded around median and anteriorly (i≤0.5d); metepisternum densely punctate (i≤d) distinctly longitudinally striate anterodorsally; metapostnotum rugose, fewer transverse lineations and more weakly microsculptured posteriorly; propodeum coarsely imbricate, moderately densely punctate (i=1–2d); T1 moderately densely punctate (i=1–2d); T2–T5 moderately densely punctate-puncticulate (i=1–3d); T6–T7 moderately sparsely punctate (i≥2d); marginal zones of terga weakly imbricate and shiny, shallowly punctate.

*Structure*: Labrum 2.4× wider than long (24:10); malar space ~0.6× as long as clypeal lateral (5:8); LOT below anterior tentorial pits; clypeus ~1.2× as long as maximum width in frontal view (35:29) extending for less than half its length beyond LOT; clypeus lacking median longitudinal groove; subantennal sutures ~1.15× as long as shortest distance between them (14:12); IAD 1.5× AOD (12:8); scape ~2.5× as long as maximum width (23:9); pedicel slightly shorter than wide (7.5:8); F1 slightly shorter than wide (8:8.5); F2 shorter than F1 and ~0.75× as long as F3 (F2:F3 - 7:9); UOD ~1.4× LOD, IOD ~1.1× UOD (UOD:IOD:LOD - 47:51:34); frontal line carinate in lower half, flat above; MOC subequal to width of head (84:83); OOC ~0.7× IOC (12:17); genal area ~0.6× as wide as compound eye in lateral view (18:31); ratio of lengths of mesoscutum: scutellum: metanotum: metapostnotum - 55:22:10:12; probasitarsus ~3.33× as long as maximum depth (20:6); prodistitarsus ~0.9× as long as preceding three tarsomeres combined (from II to V - 6:5:4:13); metafemur nearly twice as long as maximum depth (61:31); summit of metatibial ventral convexity at approximately two-thirds tibial length; metatibia ~2.2× as long as maximum depth (55:25); in apical view apical lamina of metatibia reduced, short and thick, length ~0.5× OD (5:9); ventral metatibial carina angled ventrad, somewhat laminate, rounded inflection near midpoint, posterior carina absent basally and originating in apical half of ventral carina; metabasitarsus 3.4× as long as maximum depth (34:10); S1 process apical surface longitudinally concave, in lateral view anterior and posterior margins convergent apically. S7 apodemal arm sclerotized margin entire, fully enclosing membranous portion of arm; ventral lobe ~0.5× as broad as basal attached length; dorsal lobe strap-like, subparallel-sided and somewhat anteriorly curved, with basal tuft of long setae and a short apical row, midlength bare. S8 lateral process ~1.1× wider than long, anteromedial sclerotized margin wide and subparallel to margin throughout. Gonobase apicoventral truncate process biconvex and distinctly notched by a width less than one convexity.

**Female.** Length 4.5–5.1mm, forewing length 2.8–3.0mm, head width 1.0–1.1mm, thorax width 1.1–1.2mm, median ocellar diameter (OD) 0.10mm.

*Colouration*: Black to black-brown except as follows: mandible with yellow basal spot, translucent yellow in apical half except apex translucent brown; antenna variable (apicoventral margin of F3 yellow-brown to brown; ventral surface of flagellum from F4 to terminal flagellomere yellow-brown to yellow, flagellomeres suffused with brown basally). Following parts yellow: dorsal surface of protibia, darkened to yellow-brown apicodorsally; apical half of prodistitarsus; narrow basal ring on mesotibia, wider dorsally. Anterior spot on tegula absent or faintly yellow. Following parts brown: apicodorsal spot on mesotibia; apical one-third of mesodistitarsus; narrow apical ring on metafemur (yellow-brown on some specimens). Metatibia with wide basidorsal band yellow-brown. Metasoma black, T1–T5 marginal zones narrowly translucent yellow.

*Pubescence*: As in male except as follows: clypeal hairs short and sparse (0.5OD); genal beard sparse (0.5–1.5OD); discs of mesoscutum, scutellum, metanotum with sparse short hairs (≤0.5OD), longer and denser toward lateral margin of mesoscutum (0.5OD), and toward lateral and posterior margins of scutellum (1–1.5OD), longest posteromedially; scopae on metafemur and metatibia (1–1.5OD); T1–T3 apicolateral patches of tomentum (0.5–1OD); S1 hairs long and moderately dense (≤2OD); S2 scopal hairs (1–2OD).

*Surface sculpture*: As in male except as follows: lower paraocular area densely punctate (i=d); scape and frontal, vertexal, and genal areas moderately densely punctate (i=1–2d), scape shallowly punctate, genal area weakly imbricate; hypostomal area weakly imbricate to glossy apically, shallowly and moderately sparsely punctate (i≥2d); mesoscutum and scutellum densely punctate (i=0.5–1d); mesepisternum irregularly punctate, sparse below scrobe and anterior of episternal groove (i=1–3d), irregularly spaced on hypoepimeral area (i≤2d); metanotum moderately densely punctate (i=0.5–2d); metapostnotum sparsely longitudinally carinate, carinae irregular; propodeum moderately densely punctate (i=1–2d); T1–T5 punctures small and sparse (i=1–3d on T1–T3; i≥2d on T4–T5); T6 moderately densely punctate (i=1–2d).

*Structure*: Labrum ~2.5× wider than long (21:8); malar space 0.5× as long as clypeal lateral (4:8); LOT below anterior tentorial pits; clypeus subequal in length to maximum width in frontal view (30:28) extending for less than half its length beyond LOT; median longitudinal groove weak on clypeus; subantennal suture length subequal to shortest distance between them (11:12); IAD ~1.5× AOD (12.5:8); scape ~3.2× as long as maximum width (21:6.5); pedicel longer than wide (7.5:6.5); F1 subequal in length and width (6:6); F2 ~0.67× as long as F1 and same length as F3 (F2:F3 - 4:4); UOD ~1.3× LOD, IOD ~1.05× as long as UOD (UOD:IOD:LOD - 44:47:34); frontal line carinate in lower half, flat above; MOC subequal to or slightly longer than width of head (78:74); OOC ~0.75× IOC (11.5:16); genal area ~0.6× as wide as eye in lateral view (16:26); ratio of lengths of mesoscutum: scutellum: metanotum: metapostnotum – 51:20:10:11.5; probasitarsus ~3× as long as maximum depth (20:6.5); length of prodistitarsus subequal to that of preceding three tarsomeres combined (from II to V - 5.5:5:4:14); metafemur ~2.6× as long as maximum depth (42:16); metatibia ~4.2× as long as maximum depth (55:13); metabasitarsus ~3.67× as long as maximum depth (33:9).

**Figure 32.**
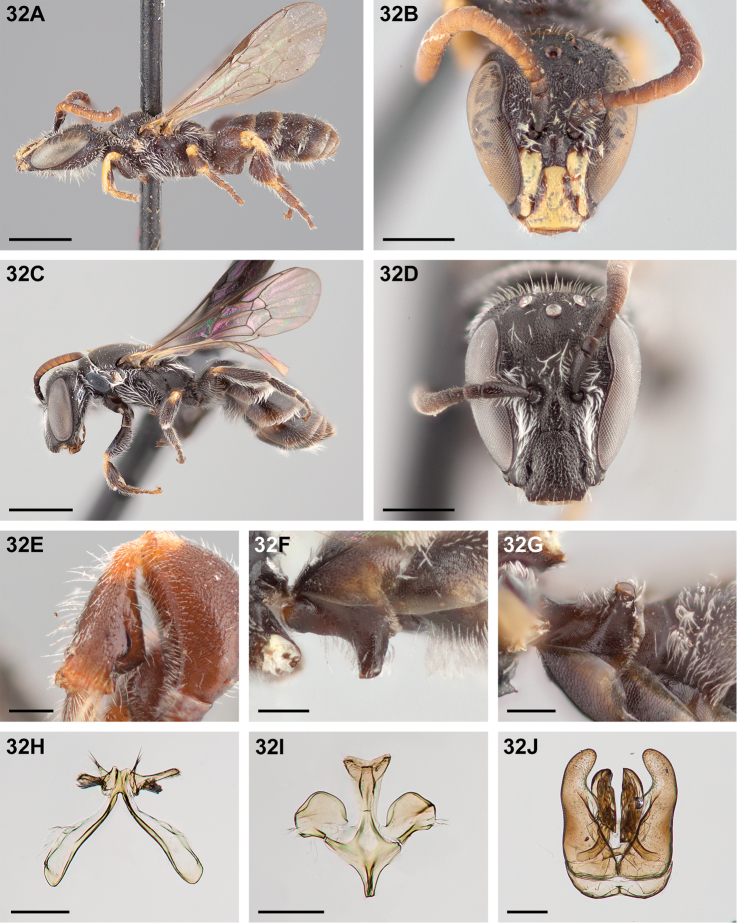
*Chilicola mantagua*: **A** male habitus, lateral view, scale bar 1 mm **B** male head, frontal view, scale bar 0.5 mm **C** female habitus, lateral view, scale bar 1 mm **D** female head, frontal view, scale bar 0.5 mm **E** male metatibia, posterior view^†^, scale bar 0.25 mm **F** male S1 process, lateral view^†^, scale bar 0.25 mm **G** male S1 process, apical view, scale bar 0.25 mm **H** male S7, scale bar 0.25 mm **I** male S8, scale bar 0.25 mm **J** male genital capsule, scale bar 0.25 mm.

###### Material studied

(38 males & 20 females): **Holotype** (male): Region V, Lilenes, *S 32.928°, W 71.546°, 19m*, 13.i.1967, Toro (AMNH); **Allotype** (female): same locality and date as holotype, De la Hoz (AMNH); ***Chilicola valparaiso* holotype** (male): Region V, Peñuelas, *S 33.149°, W 71.559°, 362m*, i.1975, Cerda (AMNH) **Region V**: one paratype female, same locality as holotype, 5.x.1975, L. Ruz (PUCV); two paratype females, same data, PUCV-ENTO 21480, PUCV-ENTO 21481 (PUCV); one paratype male, same locality as holotype, 23.x.1966, De la Hoz (AMNH); one paratype male, same locality as holotype, 25.x.1966, De la Hoz (AMNH); one paratype male, same locality as holotype, 23.x.1966, H. Toro G. (AMNH); two paratype males, same locality as holotype, 5.x.1975, H. Toro, PUCV-ENTO 21467, PUCV-ENTO 21474 (PUCV); one paratype male, Mantagua, *S 32.866°, W 71.491°, 15m*, 13.i.1967, H. Toro G. (AMNH); three paratype males, same locality and collector, xii.1971 (AMNH); two paratype males, Reñaca, *S 32.972°, W 71.544°, 14m*, 20.xi.1966, De la Hoz (AMNH); one paratype female, Pr. Aconcagua, 5.xi.1971, A.R. Moldenke, on *Chaetanthera linearis*, INT BIOL PROGRAM 45330 (AMNH); one male, same locality as holotype, 26.x.1979, L. Ruz (PUCV); one female, Lilenes Alto, *S 32.934°, W 71.544°, 74m*, i.2000, F. Vivallo (UFRJ); one male, Dunas de Concón, *S 32.941°, W 71.552°, 41m*, 12.i.1976, G. Cerda (AMNH); one male, Reñaca, *S 32.972°, W 71.544°, 14m*, 22.x.1994, J.G. Rozen (AMNH); one male, same locality, 17.i.1973, G. Cerda (AMNH); one female, Curauma, *S 33.015°, W 71.541°, 61m*, 5.i.2000, F. Vivallo (UFRJ); seven males, Viña del Mar, *S 33.025°, W 71.552°, 18m*, xi.1988, Fritz (AMNH); one female, Peñuelas, *S 33.149°, W 71.559°, 362m*, 22.i.1975, Cerda (AMNH); two males and two females, Concón, *S 32.917°, W 71.5°, 60m*, 21.x.2008, K.S. Ramos, CCDB-06736 F02 // PCYU-KCC-10040, CCDB-06736 F03 // PCYU-KCC-10041, CCDB-06736 F01 // PCYU-KCC-10039, CCDB-06736 E12 // PCYU-KCC-10038 (PCYU); one female, Mantagua, *S 32.866°, W 71.491°, 15m*, 26.xi.1976, H. Toro Jr (AMNH); one female, same locality, 2.ii.1967, H. Toro, PUCV-ENTO 21990 (PUCV); one male, Mantagua Dunas, *S 32.865°, W 71.494°, 13m*, 12.i.1976, G. Cerda B. (PUCV); six males and five females, Playa Ritoque, N of Concón, *S 32.849°, W 71.515°, 13m*, 5.x.2000, L. Packer (PCYU); one male and one female, same data (CTMI); one male, same data, CCDB-03755 A04 (PCYU); two males, Aconcagua Prov. Papudo/Zapallar, 5.xi.1971, A.R. Moldenke, on *Chaetanthera linearis*, INT BIOL PROGRAM 45330, INT BIOL PROGRAM 45387 (AMNH); one male, Aconcagua Prov. Papudo/Zapallar, date unknown, H. Toro (AMNH); one female, Aconcagua Prov., 12.i.1973, A.R. Moldenke, on *Carpobrotus chilensis*, INT BIOL PROGRAM 45397 (AMNH); **Region IV**: one female, Choapa, Pichidangui, S 32.1383°, W 71.5067°, 15m, 10.xi.2003, F.D. Parker, pan trap, CHL-14510-85 // Native Bee Survey BBSL695300 (BBSL).

###### Distribution.

Coastal, southern Coquimban Desert, from Peñuelas (Region V) north to Pichidangui (Region IV); 13–362m a.s.l.

###### Ecology.

Collected on *Carpobrotus chilensis* (Molina) N. E. Br. (1 record), *Cheatanthera linearis* Poepp. ex Less. (2 records), and *Chorizanthe vaginata* Benth. (1 record). Recorded October to February.

##### Chilicola (Heteroediscelis) charizard

Taxon classificationAnimaliaHymenopteraColletidae

Monckton sp. n.

http://zoobank.org/F896D9B4-8723-4F95-9F30-C9221F3F6EC2

**Figure 33.**
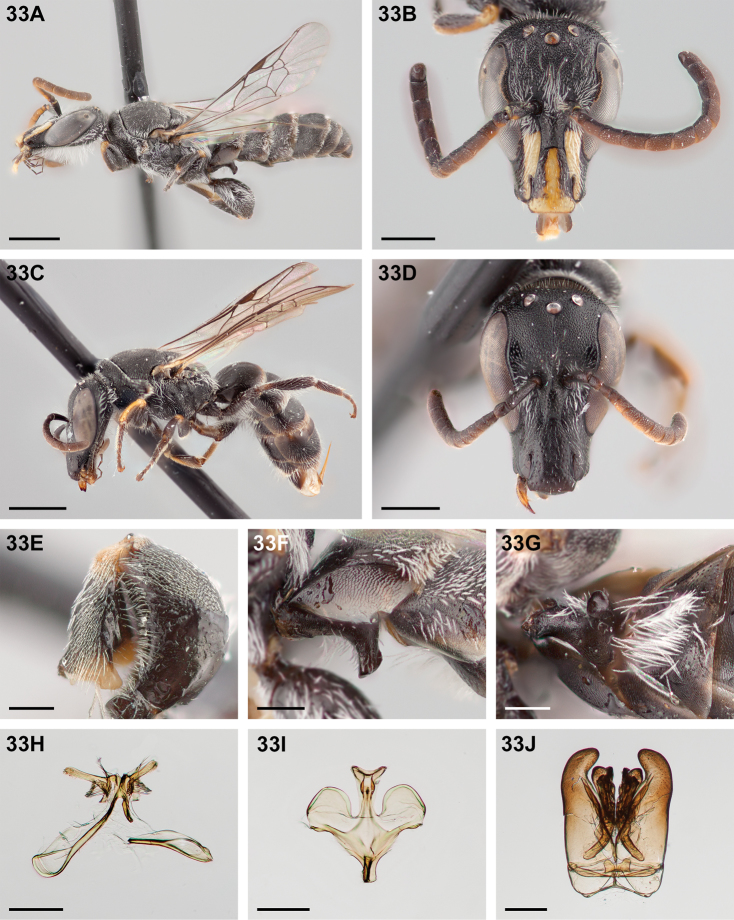
*Chilicola charizard*: **A** male habitus, lateral view, scale bar 1 mm **B** male head, frontal view, scale bar 0.5 mm **C** female habitus, lateral view, scale bar 1 mm **D** female head, frontal view, scale bar 0.5 mm **E** male metatibia, posterior view^†^, scale bar 0.25 mm **F** male S1 process, lateral view, scale bar 0.25 mm **G** male S1 process, apical view, scale bar 0.25 mm **H** male S7 (C.chzd.003), scale bar 0.25 mm **I** male S8 (C.chzd.003), scale bar 0.25 mm **J** male genital capsule (C.chzd.003), scale bar 0.25 mm.

Figs 5D ; 11B–C ; 12A–C ; 22C–D ; 33A–J ; 49

###### Diagnosis.

This species is diagnosable by the malar space, which is the longest in the subgenus: in males it is ~2.7× as long as the clypeal lateral, in females it is ~2.33× as long as the clypeal lateral.

###### Description.

**Male (Holotype).** Length 6.1mm, forewing length 3.6mm, head width 1.2mm, thorax width 1.3mm, median ocellar diameter (OD) 0.13mm.

*Colouration*: Black-brown, following parts yellow: labrum; clypeus except broadly dark along epistomal suture and apex brown laterad; lower paraocular area, extending further medially than laterally, more than 1OD above transverse portion of epistomal suture, not reaching lower tangent of antennal socket; apicoventral spot on scape; apicoventral surface of pedicel; ventral surface of F1–F5, F5 suffused with brown; apical rim of protrochanter posteriorly; apex of profemur broadly on anterior surface, narrowly on posterior surface; dorsal surface of protibia, including dorsal half of anterior surface; narrow apical ring on mesofemur; narrow basal ring and apicodorsal rim of mesotibia; basal quarter of metatibia, as well as apical quarter in ventral half only; basal quarter of metabasitarsus ventrally; anterior spot on tegula. Ventral surface of F6–F10 yellow-brown. Ventral surface of F11 brown. Apicoventral rim of metacoxa brown. Metasoma black, T1–T6 marginal zones amber to translucent yellow at margins.

*Pubescence*: White, hairs generally short (0.5OD) and sparse, not especially plumose; on lower paraocular area and around antennal base moderately long and moderately dense (1OD); genal beard long and dense (0.5–3OD) longest at midlength; discs of mesoscutum, scutellum, and metanotum mostly bare, few short hairs (0.5OD), longer and denser toward lateral margin as follows: mesoscutum (0.5–1OD), scutellum (0.5–1OD) also on posterior margin, longest posteromedially, metanotum (≤2.5OD); propodeal hairs moderately long and moderately dense dorsolaterally (0.5–1.5OD); T1–T3 apicolateral patches of tomentum (0.5–1OD); S2 hairs long and dense (1.5OD basolaterad, 1OD distilaterad, shorter mesad).

*Surface sculpture*: Microsculpture imbricate, integument generally dull; punctures generally small and deep. Clypeus and lower paraocular area moderately densely punctate (i=1–2d) except clypeus sparsely punctate medially (i=2–3d); supraclypeal area irregularly punctate (i=1–3d) except densely punctate toward lateral margin (i≤d); frontal area very densely punctate (i≤0.5d), densely punctate around ocelli (i≤d); ocellocular space moderately densely punctate adjacent to compound eye (i=1–2d) becoming impunctate adjacent to lateral ocellus; vertexal area densely punctate (i≤d); scape shallowly and densely punctate (i=d) more densely punctate apicoventrally (i≤d); genal area microstriate and moderately densely punctate (i≤2d); hypostomal area glossy apically and densely punctate (i=d); pronotum, mesoscutum, and scutellum densely punctate (i=0.5–1d), scutellar punctures crowded around median (i≤d); lateral surface of pronotum coarsely imbricate; mesepisternum irregularly punctate, moderately dense below scrobe (i=1–2d), sparser anterior of episternal groove (i=1–3d), impunctate just dorsad of scrobe, otherwise moderately dense on hypoepimeral area (i≤2d); metanotum moderately densely punctate (i=1–2d) punctures crowded around median and anteriorly (i≤0.5d); metepisternum densely punctate (i≤d) distinctly longitudinally striate anterodorsally; metapostnotum rugose, minutely so posteriorly; propodeum coarsely imbricate, rugulose posteriorly, moderately densely punctate throughout (i=1–2d); T1–T5 coarsely imbricate and moderately densely punctate (i≤2d); T6 coarsely imbricate and moderately densely punctate (i=1–2d); T7 irregularly punctate (i=1–3d); marginal zones of terga weakly imbricate and shiny, minutely punctate.

*Structure*: Labrum ~2.7× wider than long (23:8.5); malar space 2.75× as long as clypeal lateral (22:8); LOT above anterior tentorial pits; clypeus ~1.4× as long as maximum width in frontal view (40:28) extending for approximately three quarters of its length beyond LOT; clypeus lacking median longitudinal groove; subantennal sutures ~1.6× as long as shortest distance between them (22:14); IAD 2× AOD (12:6); scape ~2.6× as long as maximum width (25:9.5); pedicel shorter than wide (8:9); F1 slightly shorter than wide (9:9.5); F2 ~1.3× as long as F1 and shorter than F3 (F2:F3 - 12:14); UOD 1.5× LOD, IOD slightly wider than UOD (UOD:IOD:LOD - 51:53:34); frontal line carinate in lower half, flat above; MOC longer than width of head (101:87); OOC 0.6× IOC (12:20); genal area less than half as wide as compound eye in lateral view (15:34); ratio of lengths of mesoscutum: scutellum: metanotum: metapostnotum - 65:24:12:16; probasitarsus ~4.1× as long as maximum depth (29:7); prodistitarsus ~0.7× as long as preceding three tarsomeres combined (from II to V - 7:6:5:13); metafemur nearly twice as long as maximum depth (63:32); summit of metatibial ventral convexity at approximately two-thirds tibial length; metatibia ~2.1× as long as maximum depth (57:27); in apical view apical lamina of metatibia broad and thick, length ~0.8× OD (7:9); ventral metatibial carina angled ventrad, rounded inflection near midpoint, posterior carina absent basally and originating at midpoint of ventral carina; metabasitarsus 4.2× as long as maximum depth (42:10); S1 process apical surface weakly concave except slightly convex anteromedially, in lateral view anterior and posterior margins divergent apically. S7 apodemal arm sclerotized margin recurved mesad, dividing membranous portion of arm; ventral lobe narrow, almost linear; dorsal lobe strap-like, sides subparallel and somewhat anteriorly curved, with basal tuft of long setae and short apical row, midlength bare. S8 lateral process subequal in width and length, anteromedial sclerotized margin wide and following marginal contour. Gonobase apicoventral truncate process biconvex and distinctly notched by a width less than one convexity.

**Female (Allotype).** Length 5.8mm, forewing length 3.3mm, head width 1.1mm, thorax width 1.3mm, median ocellar diameter (OD) 0.12mm.

*Colouration*: Black to black-brown except as follows: mandible with yellow basal spot, translucent yellow in apical half except apex translucent brown. Following parts brown: pedicel, F1 and F2, on apicoventral margins (the latter two additionally with small yellow-brown apicoventral spot); apicodorsal rim of metafemur; basidorsal spot on metatibia. Following parts yellow: ventral surface of antenna from F3 to terminal flagellomere; dorsal surface of protibia; apical half of prodistitarsus; basidorsal and apicodorsal spots on mesotibia; anterior spot on tegula. Metasoma black, T1–T5 marginal zones narrowly translucent yellow.

*Pubescence*: As in male except as follows: clypeal hairs short and sparse (0.5OD); genal beard sparse (0.5–1.5OD); discs of mesoscutum, scutellum, and metanotum with sparse short hairs (≤0.5OD), longer and denser toward lateral margin as follows: mesoscutum (0.5–1OD), scutellum (1–1.5OD) also on posterior margin, longest posteromedially, metanotum (≤2OD); propodeal hairs moderately long and dense dorsolaterally (0.5–1OD); scopae on metafemur and metatibia (1–1.5OD); T1–T3 apicolateral patches of tomentum (0.5–1OD); S1 hairs long and moderately dense (≤2OD); S2 scopal hairs (1–2.5OD).

*Surface sculpture*: As in male except as follows: supraclypeal area irregularly punctate (i=1–2d) densely punctate toward lateral margin (i≤d); lower paraocular area densely punctate (i=d); frontal and vertexal areas densely punctate (i≤d); scape shallowly and moderately densely punctate (i=1–2d); genal area weakly imbricate and densely punctate (i=d); hypostomal area weakly imbricate to glossy apically, shallowly and moderately densely punctate (i=1–2d); mesoscutum moderately densely punctate (i=0.5–2d) punctures crowded around median (i≤d); scutellum densely punctate (i=0.5–1d) punctures crowded around median (i≤d); mesepisternum irregularly punctate, moderately dense below scrobe (i=1–2d), sparser anterior of episternal groove (i=2–3d), impunctate just dorsad of scrobe, otherwise dense on hypoepimeral area (i≤d); metanotum densely punctate (i=0.5–1d) punctures crowded around median and anteriorly (i≤0.5d); metapostnotum rugose, more weakly microsculptured posteriorly; propodeum coarsely imbricate, moderately densely punctate (i=1–2d); T1–T5 sparsely punctate (i≥2d on T1; i=1–3d on T2–T5); T6 moderately densely punctate (i=1–2d); marginal zones weakly imbricate and shiny, mostly impunctate.

*Structure*: Labrum 2.25× wider than long (22.5:10); malar space ~2.33× as long as clypeal lateral (21:9); LOT above anterior tentorial pits; clypeus ~1.5× as long as maximum width in frontal view (40:27) extending for more than three-quarters its length beyond LOT; median longitudinal groove weak on clypeus; subantennal sutures 1.85× as long as the shortest distance between them (18.5:10); IAD ~1.5× AOD (11.5:7.5); scape ~3× as long as maximum width (25:8); pedicel longer than wide (8.5:7); F1 slightly longer than wide (7.5:7); F2 0.6× as long as F1 and ~0.65× as long as F3 (F2:F3 – 4.5:7); UOD ~1.4× LOD, IOD ~1.05× as long as UOD (UOD:IOD:LOD - 48:51:34); frontal line carinate in lower half, flat above; MOC longer than width of head (98:81); OOC ~0.5× IOC (11:20); genal area ~0.6× as wide as eye in lateral view (16:28); ratio of lengths of mesoscutum: scutellum: metanotum: metapostnotum – 61:22:10:15; probasitarsus ~4.6× as long as maximum depth (30:6.5); prodistitarsus ~0.75× as long as preceding three tarsomeres combined (depth (20:6.5); length of prodistitarsus subequal to that of preceding three tarsomeres combined (from II to V - 8:6.5:5:14); metafemur ~3.1× as long as maximum depth (50:16); metatibia ~4.1× as long as maximum depth (62:15); metabasitarsus 4× as long as maximum depth (40:10).

###### Type material

(6 males & 11 females): **Holotype** (male): Region III, Emb. Sta Juana, C-479 km 21.5, S 28.6747°, W 70.6432°, 642m, 15.x.2013, S. Monckton, CCDB-19989 A10 // PCYU 0021672 (PCYU); **Allotype** (female): Region III, Chuingo, S 28.8251°, W 70.3560°, 963m, 15.ix.2010, L. Packer, B09857-D12 Bees of Chile332 (PCYU); **Paratypes** (3 males & 8 females): **Region III**: one female, same data as holotype, B09857-E05 Bees of Chile337 (PCYU); one male, C-453 km 22, S 28.1816°, W 69.8261°, 1625m, 30.ix.2013, S. Monckton, CCDB-19989 A11 // PCYU 0021673 (PCYU); one female, Fundo La Semilla, 125 km SE Copiapó, S 28.2507°, W 69.741°, 2358m, 17.x.2004, F.D. Parker, M.E. Irwin, pan trap, CHL-14509-57 // FDP52565 (BBSL); one female, Emb. Sta Juana, C-479 km 21.5, S 28.6747°, W 70.6432°, 645m, 15.x.2013, S. Monckton, PCYU 0020910 (PCYU); one female, Alto del Carmen, *S 28.759°, W 70.486°, 2272m*, 2.x.1982, F. Rodriguez (BBSL); **Region IV**: two males, Incahuasi, *S 29.228°, W 71.014°, 779m*, 10.x.1981, H. Toro (PUCV); one female, same data, H. Doñoso (PUCV); one female, same data, H. Burgos (PUCV); one female, same data, B. Dyer (PUCV); one female, same locality, 1.x.1982, O. Martinez (PUCV); one female, Elqui Prov., 6 km S of Vicuña, *S 30.085°, W 70.725°, 889m*, 16.xi.1991, J.G. Rozen, on *Pleurophora pungens* (AMNH); one male, E of Vicuña, km 114, S 29.9123°, W 70.3013°, 1429m, ix.2010, L.Packer, white pan (PCYU); one female, Puente Las Terneras, S 29.9089°, W 70.2522°, 1644m, 4.x.2010, L. Packer, PCYU 0021698 (PCYU); one male, E of Vicuña, 127.8 km, S 29.9777°, W 70.2293°, 1711m, 12–19.2010, L. Packer (PCYU).

###### Variation.

Some females have the apicoventral surface of pedicel, F1 and F2 yellow-brown, and/or a brown or yellow-brown spot on the lower paraocular below the anterior tentorial pit.

###### Distribution.

Intermediate Desert & Central Andean Cordillera, from Fundo La Semilla (Region III) south to Elqui Prov., 6 km S of Vicuña (Region IV) and west to Incahuasi & Embalse Sta. Juana (Region III); 645–2358m a.s.l.

###### Ecology.

Collected on *Pleurophora pungens* D. Don (1 record). Recorded September to November.

###### Etymology.

The specific epithet is in homage to the fictional monster which this species resembles. It is treated as a noun in apposition.

##### Chilicola (Heteroediscelis) curvapeligrosa

Taxon classificationAnimaliaHymenopteraColletidae

Monckton sp. n.

http://zoobank.org/C9805F8F-5F8B-43D1-8C5C-948E5637CD64

**Figure 34.**
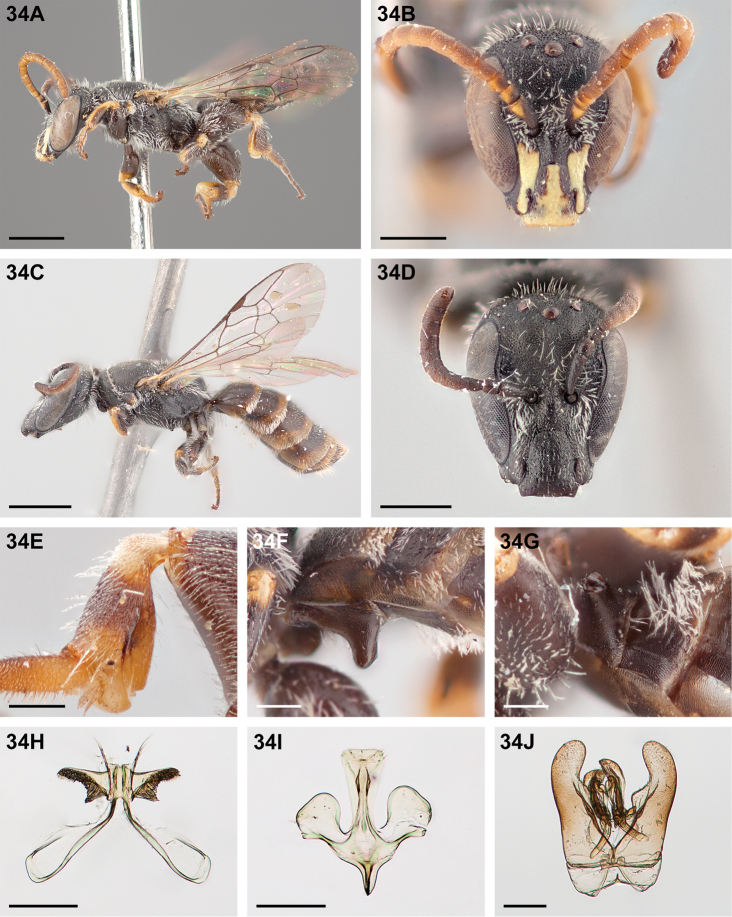
*Chilicola curvapeligrosa*: **A** male habitus, lateral view, scale bar 1 mm **B** male head, frontal view, scale bar 0.5 mm **C** female habitus, lateral view, scale bar 1 mm^†^ **D** female head, frontal view, scale bar 0.5 mm **E** male metatibia, posterior view, scale bar 0.25 mm **F** male S1 process, lateral view^†^, scale bar 0.25 mm **G** male S1 process, apical view, scale bar 0.25 mm **H** male S7 (C.cplg.006), scale bar 0.25 mm **I** male S8 (C.cplg.006), scale bar 0.25 mm **J** male genital capsule (C.cplg.006), scale bar 0.25 mm.

Figs 9C–D ; 10A–B ; 24A–B ; 34A–J ; 49

###### Diagnosis.

Males are diagnosable by the combination of S1 process in lateral view having a rounded anterior apical angle, malar space subequal to or slightly shorter than the clyperal lateral, and first flagellomere no longer than wide. Females are diagnosable by the combination of malar space subequal in length to the clypeal lateral (0.9–1×)and leg colouration with apex of metatibia black-brown and following parts brown: apicodorsal spot on mesotibia, apicodorsal rim of metafemur, basidorsal spot on metatibia. Females of *Chilicola mavida* are most similar, but have lighter leg colouration (apicodorsal spot on mesotibia yellow-brown, apicodorsal rim of metafemur yellow, wide basal ring of metatibia yellow, posterior apical rim of metatibia yellow-brown).

###### Description.

**Male (Holotype).** Length 5.7mm, forewing length 3.5mm, head width 1.2mm, thorax width 1.2mm, median ocellar diameter (OD) 0.13mm.

*Colouration*: Black-brown, following parts yellow: labrum; clypeus except broadly dark brown along epistomal suture and apex yellow along entire width; lower paraocular area, extending ~1OD above transverse portion of epistomal suture medially, further laterally but not reaching lower tangent of antennal socket; apicoventral surface of scape; protrochanter apical rim posteriorly; apex of profemur broadly on anterior surface, narrowly on posterior surface; dorsal surface of protibia, including dorsal half of anterior surface; narrow apical ring on mesofemur; basal and apical ring of mesotibia, the latter extending to cover apical half of anterodorsal surface; apicodorsal rim of metafemur; ventral surface of metatibia, as well as dorsal surface in basal quarter, narrowly yellow apically except broad around apical lamina and posterior concavity; basal half of metabasitarsus ventrally; anterior spot on tegula; apex of T7. Following parts orange-brown: ventral surface of antenna from apical half of pedicel to terminal flagellomere, narrowing basad on pedicel and F1; ventral surface of mesobasitarsus. Apicoventral rim of metacoxa and metatrochanters translucent yellow. Apical half of prodistitarsus brown. Metasoma black, T1-T6 amber marginal zones to translucent at margins.

*Pubescence*: White, hairs generally short (0.5OD) and sparse, not especially plumose; on lower paraocular area and around antennal base moderately long and moderately dense (1OD); genal beard long and dense (0.5–3OD) longest at midlength; discs of mesoscutum, scutellum, and metanotum mostly bare, few short hairs (0.5OD), longer and denser toward lateral margin as follows: mesoscutum (0.5–1OD), scutellum (1–2OD) also on posterior margin, longest posteromedially, metanotum (≤2OD); propodeal hairs moderately long and dense dorsolaterally (0.5–1.5OD); T1-T2 apicolateral patches of tomentum (0.5–1OD), T3 apicolateral hair band sparse, not tomentose; S2 hairs long and dense (1.5OD basolaterad, 1OD distilaterad, shorter mesad).

*Surface sculpture*: Microsculpture imbricate, integument generally dull; punctures generally small and deep. Clypeus and supraclypeal and lower paraocular areas moderately densely punctate (i=1-2d) except clypeus sparsely punctate medially (i=2-3d); frontal area very densely punctate (i≤0.5d), densely punctate around ocelli (i≤d); ocellocular space moderately densely punctate adjacent to compound eye (i=1-2d) becoming impunctate adjacent to lateral ocellus; vertexal area densely punctate (i≤d); scape shallowly and moderately densely punctate (i=1-2d) densely punctate apicoventrally (i≤d); genal area microstriate and moderately densely punctate (i≤2d); hypostomal area rough, weakly imbricate and densely punctate (i=d); pronotum moderately densely punctate (i=1-2d), lateral surface coarsely imbricate; mesoscutum densely punctate (i=0.5-1d) sparser laterally (i=1-2d) crowded around median (i≤d); scutellum moderately densely punctate (i=1-2d) crowded around median (i≤d); mesepisternum irregularly punctate, sparse below scrobe (i=2-3d), sparser anterior of episternal groove (i≥3d), impunctate just dorsad of scrobe, otherwise irregularly spaced on hypoepimeral area (i=1-3d); metanotum moderately densely punctate (i=1-2d) punctures crowded around median and anteriorly (i≤0.5d); metepisternum densely punctate (i≤d) distinctly longitudinally striate anterodorsally; metapostnotum rugose; propodeum coarsely imbricate, moderately densely punctate (i=1-2d); T1-T5 coarsely imbricate and moderately densely punctate (i≤2d); T6 coarsely imbricate posteriorly and moderately densely punctate (i=1-2d); T7 moderately densely punctate (i=1-3d); marginal zones of terga weakly imbricate and shiny, minutely punctate.

*Structure*: Labrum ~2.4× wider than long (22:9); length of malar space subequal to that of clypeal lateral (7:7.5); LOT at level of anterior tentorial pits; clypeus ~1.1× as long as maximum width in frontal view (31:28) extending for half its length beyond LOT and convex above anterior tentorial pit, in lateral view bulging ~1OD above compound eye; median longitudinal groove on clypeus present in dorsal half, absent ventrad; subantennal sutures ~1.1× as long as the shortest distance between them (15:13.5); IAD nearly double AOD (12.5: 6.5); scape ~2.5× as long as maximum width (23:9); pedicel as long as wide (8:8); F1 as long as wide (8.5:8.5); F2 ~1.4× as long as F1 and slightly shorter than F3 (F2:F3 - 12:12.5); UOD ~1.4× LOD, IOD ~1.1× UOD (UOD:IOD:LOD - 48:52:34); frontal line carinate between antennal bases for a length less than 0.5OD, flat just below median ocellus for a length of ~1OD, otherwise undefined; MOC subequal to width of head (84:85); OOC two-thirds IOC (12:18); genal area approximately half as wide as eye in lateral view (16:30); ratio of lengths of mesoscutum: scutellum: metanotum: metapostnotum – 58:21:11:17; probasitarsus ~3.4× as long as maximum depth (22:6.5); prodistitarsus ~0.9× as long as preceding three tarsomeres combined (from II to V - 6:5:4.5:14); metafemur ~1.7× as long as maximum depth (65:38); summit of metatibial ventral convexity near tibial apex; metatibia less than twice as long as maximum depth (51:28); in apical view apical lamina of metatibia broad and thick, length ~1.1× OD (10:9); ventral metatibial carina bowed ventrad and absent basally, originating at midpoint of weakly sigmoid posterior carina; metabasitarsus 3.7× as long as maximum depth (37:10); S1 process apical surface with a longitudinal median ridge, in lateral view anterior and posterior margins subparallel apically and median ridge projecting posteriorly from apex. S7 apodemal arm sclerotized margin terminating laterally at arm midlength; ventral lobe narrow, almost linear; dorsal lobe roughly triangular, wide at base and narrow apically, with row of setae long basally and shorter apically. S8 lateral process ~0.9× as wide as long, anteromedial sclerotized margin wide and following marginal contour. Gonobase apicoventral truncate process biconvex and distinctly notched by a width less than one convexity.

**Female (Allotype).** Length 5.0mm, forewing length 3.0mm, head width 1.0mm, thorax width 1.1mm, median ocellar diameter (OD) 0.10mm.

*Colouration*: Black to black-brown except as follows: mandible with yellow basal spot, translucent yellow in apical half except apex translucent brown. Following parts brown: apicoventral margin of pedicel; apical two-thirds of prodistitarsus; apicodorsal spot on mesotibia; apicodorsal rim of metafemur; basidorsal spot on metatibia. Following parts yellow-brown: ventral surface of antenna from F3 to terminal flagellomere, flagellomeres suffused with darker brown basally; posterior surface of mesobasitarsus. Following parts yellow: dorsal surface of protibia; narrow basal ring on mesotibia; anterior spot on tegula. Metasoma brown, T1-T5 marginal zones amber to translucent yellow at margins.

*Pubescence*: As in male except as follows: clypeal hairs short and sparse (0.5OD); genal beard sparse (0.5–2OD); discs of mesoscutum, scutellum, and metanotum with sparse short hairs (≤0.5OD), longer and denser toward lateral margin of mesoscutum (0.5OD), and toward lateral and posterior margins of scutellum (1–1.5OD) longest posteromedially; propodeal hairs moderately long and dense dorsolaterally (0.5–1OD); scopae on metafemur and metatibia (1–1.5OD); T1-T2 apicolateral patches of tomentum (0.5–1OD), T3 apicolateral hair band sparse, not tomentose; S1 hairs long and moderately dense (≤2.5OD); S2 scopal hairs (1–2.5OD).

*Surface sculpture*: As in male except as follows: lower paraocular area densely punctate (i=d); frontal area densely punctate (i≤d); vertexal area moderately densely punctate (i=1-2d); scape shallowly and moderately densely punctate (i=1-2d); genal area very weakly imbricate and moderately densely punctate (i=1-2d); hypostomal area glossy apically and moderately densely punctate (i=1-2d); mesoscutum and scutellum densely punctate (i=0.5-1d); mesepisternum irregularly punctate, sparse below scrobe and anterior of episternal groove (i≥3d), impunctate just dorsad of scrobe, otherwise irregularly spaced on hypoepimeral area (i=1-3d); metanotum moderately densely punctate (i=0.5-2d); metapostnotum rugose; T1-T5 punctures small and sparse (i≥2d); T6 moderately densely punctate (i=1-2d).

*Structure*: Labrum ~2.3× wider than long (21.5:9.5); malar space ~0.9× as long as clypeal lateral (6.5:7.5); LOT at level of anterior tentorial pits; clypeus slightly longer than maximum width in frontal view (29:27) extending for half its length beyond LOT and convex above anterior tentorial pit as in male; median longitudinal groove weak on clypeus; subantennal sutures ~1.1× as long as the shortest distance between them (13.5:12); IAD 1.5× AOD (12:8); scape ~3.2× as long as maximum width (21:6.5); pedicel ~1.33× as long as wide (8:6); F1 slightly longer than wide (6.5:6); F2 ~0.75× as long as F1 (5) and ~0.8× as long as F3 (6); UOD ~1.3× LOD, IOD ~1.1× UOD (UOD:IOD:LOD - 45:49:34); frontal line carinate in lower half, flat above; MOC longer than width of head (80:74); OOC ~0.7× IOC (12:17.5); genal area about two thirds as wide as eye in lateral view (17:25); ratio of lengths of mesoscutum: scutellum: metanotum: metapostnotum – 53:20:9:14; probasitarsus ~3.1× as long as maximum depth (20:6.5); prodistitarsus ~1.2× as long as preceding three tarsomeres combined (from II to V - 5:4:3:14); metafemur 2.75× as long as maximum depth (44:16); metatibia ~4.2× as long as maximum depth (55:13); metabasitarsus 4× as long as maximum depth (34:8.5).

###### Type material

(27 males & 49 females). **Holotype** (male): RM Santiago, Farellones, Camino a Valle Nevado, S 33.3700°, W 70.2786°, 2393m, 31.xii.2008, L. Packer, on *Phacelia*, CHI09-2-3-005 (PCYU); **Allotype** (female): RM Santiago, Farellones, Curva 31, S 33.3563°, W 70.3248°, 2179m, 31.xii.2008, L. Packer, on *Echium vulgare*, CHI09-2-2-015 (PCYU); **Paratypes: RM Santiago**: three males and three females, same data as holotype, CHI09-2-3-003, CHI09-2-3-006, CHI09-2-3-008, B09997-B10 Bees of Chile591 // CHI09-2-3-004, CHI09-2-3-001, CHI09-2-3-002 (PCYU); one male, same data as allotype (PCYU); one female, same data as allotype, CHI09-2-2-017 (PCYU); one female, Farellones, Curva 33, S 33.3543°, W 70.3195°, 2295m, 30.xii.2012, L. Packer & R. Smith (PCYU); two males, Farellones, *S 33.354°, W 70.317°, 2385m*, 1.i.1973, A. Moldenke (AMNH); two males, Farellones, S 33.3535°, W 70.3168°, 2385m, 9.i.2009, L. Packer, CHI09-12-3-003, CHI09-12-3-004 (PCYU); one female, Farellones, S 33.2208°, W 70.1633°, 2464m, 31.xii.2008, L. Packer (PCYU); five males and one female, Farellones, Valle Nevado, *S 33.354°, W 70.249°, 3026m*, 9.xii.2006, L. Packer (PCYU); one female, same locality, 1.i.1973, A. Moldenke (AMNH); two males and two females, Valle Nevado, S 33.3411°, W 70.2950°, 2596m, 9.i.2009, L. Packer, CHI09-10-2-008, CHI09-10-2-009, CHI09-10-2-010, CHI09-10-2-011 (PCYU); one male and two females, Valle Nevado, S 33.856°, W 69.9804°, 3000m, 8.i.2009, L. Packer, CCDB-03755 G03 // CHI09-10-2-004, CCDB-03755 H02 // CHI09-10-2-003, CHI09-10-2-002 (PCYU); three males and three females, Valle Nevado, S 33.3622°, W 70.2576°, 2677m, 31.xii.2008, L. Packer, on *Phacelia* (PCYU); one male, same data, CHI09-2-5-011 (PCYU); two females, E of El Volcán, S 33.8181°, W 70.021°, 2228m, 15.ii–22.iii.2013, S. Monckton & J. Postlethwaite, pan traps (PCYU); one male and one female, Río Yeso, Hwy G-455 km 18.1, S 33.6942°, W 70.1120°, 2337m, 1–8.2013, L. Packer & R. Smith (PCYU); two males and one female, same locality, 27.ii–22.iii.2013, S. Monckton & J. Postlethwaite, pan traps (PCYU); three females, Valle El Yeso, Hwy G-457, km 16, S 33.6247°, W 69.9584°, 2705m, 7.i-26.ii.2013, S. Monckton & J. Postlethwaite, pan traps (PCYU); one female, same locality and collectors, 26.ii-23.iii.2013 (PCYU); nine females, Termas del Plomo, S 33.6209°, W 69.9196°, 2938m, 31.xii.2012, L. Packer & R. Smith (PCYU); two females, same data (BBSL); two females, same data (CTMI); one female, same locality 31.xii.2012–7.i.2013, L. Packer & R. Smith, pan trap, CCDB-19991 C04 (PCYU); four females, same locality, 26.ii-21.iv.2013, S. Monckton & J. Postlewaite, pan traps (PCYU); **Region VI**: two females, Colchagua, Vegas del Flaco, *S 34.961°, W 70.432°, 1735m*, 28-30.vi.1957, L. Peña (AMNH); one male, Termas del Flaco, *S 34.961°, W 70.432°, 1734m*, ii.1967, M. Guzman (PUCV); three females, same locality, 7.ii.1967, H. Toro (PUCV); two females, same data (AMNH); **Region VII**: one male and one female, Río Teno, Paso Vergara control post, S 35.1381°, W 70.4783°, 1789m, 28.ii-21.iii.2013, S. Monckton & J. Postlethwaite, pan traps (PCYU). **Non-type material** (4 males & 2 females): **RM Santiago**: one male, Cerro Roble, nr. Til-til, *S 32.976°, W 71.014°, 2200m*, 17.xi.2001, L. Packer (PCYU); one male, same date and collector, 18.xi.2001 (PCYU); one male, same data, CCDB-03755 F09 (PCYU); one female, same date and collector, 19.xi.2001 (PCYU); one female, same locality, 20.i.2004, collector unknown (PCYU); one male, Espinalillo, S 33.0050°, W 70.9406°, 877m, 5.x.2013, L.Packer (PCYU).

###### Variation.

Some males have the clypeal apex brown laterad. Some females have the apicoventral margin of F1 and F2 brown or yellow-brown, and/or a yellow-brown or brown spot on the lower paraocular area below the anterior tentorial pit. Many females have an orange-brown metasoma with the gradulus and usually disc of T1-T6 brown, or at minimum darker than the pregradular and marginal zones. Males from Cerro El Roble and Espinalillo have the F1 longer than wide, as in *Chilicola mavida*, but resemble *Chilicola curvapeligrosa* in both colouration and malar space length; females from these localities are consistent with the diagnosis of *Chilicola curvapeligrosa*.

###### Distribution.

Southern Andean from Farellones (RM Santiago) south to Paso Vergara (Region VII), also known from Cerro El Roble and Espinalillo (RM Santiago) in the Central Coastal Cordillera; 877–3026m a.s.l.

###### Ecology.

Collected on *Echium vulgare* L. (3 records) and *Phacelia* Juss. (14 records). Recorded October to April and June.

###### Etymology.

The specific epithet is a nounphrase inspired by the signs encountered on the road leading to the type locality; it refers to the hairpin turns used to achieve high elevation. It is to be treated as a noun in apposition.

###### Comments.

Specimens from Cerro El Roble and Espinalillo may belong to a divergent population, distinct from those found at higher altitudes in the Andes, and are thus excluded from the type series (see variation, above).

##### Chilicola (Heteroediscelis) deserticola

Taxon classificationAnimaliaHymenopteraColletidae

Toro & Moldenke, 1979

Figs 11E–F ; 15F ; 16A–C ; 17B ; 29C–D ; 31D–F ; 35A–J ; 49

Chilicola deserticola Toro & Moldenke, 1979: 121–122 (male holotype, AMNH [examined]). Packer 2008: 77. Montalva and Ruz 2010: 28 (checklist). Moure et al. 2012 (catalogue). Ascher and Pickering 2015 (checklist).

###### Diagnosis.

Males are diagnosable by the combination of malar space short (<0.5× clypeal lateral), mesobasitarsus mostly yellow, and metatibia with the combination of ventral carina extending full length of longitudinal concavity and toothed, posterior carina absent basally and originating in apical half of ventral carina, and apical lamina reduced (~0.5OD in length). Females are diagnosable by the combination of the malar space short (~0.4× clypeal lateral), frontal area without longitudinal depressions which partially accommodate the scape, and clypeus evenly and moderately densely punctate throughout (i=1-2d). Among consubgeners, *Chilicola mantagua* is most similar, but can be differentiated in both sexes by the longer malar space (≥0.5× clypeal lateral) and by the characters given in the key.

###### Description.

**Male.** Length 4.3–5.5mm, forewing length 2.7–3.3mm, head width 1.0–1.2mm, thorax width 1.0–1.3mm, median ocellar diameter (OD) 0.10–0.12mm.

*Colouration*: Black-brown, following parts yellow: labrum; clypeus except narrowly dark along epistomal suture near anterior tentorial pit and apex usually brown laterad; lower paraocular area extending ~OD above transverse portion of epistomal suture medially, further laterally and nearly reaching lower tangent of antennal socket; apicoventral surface of scape; apical one-third of profemur on anterior surface, apical quarter on posterior surface; protibia except dark medioventrally; protarsus; broad apical ring on mesofemur, wider anteriorly; broad basal and apical rings on mesotibia, expanded to basal and apical one-third or continuous on dorsal margin; mesobasitarsus ventrally to entirely; wide apical ring on metafemur; basal quarter and apical quarter of metatibia, apical colouration extending to summit of ventral convexity; metabasitarsus ventrally and at least basal one-third dorsally; anterior spot on tegula. Following parts yellow-brown: apicoventral surface of pedicel; ventral surface of antenna from F1 to terminal flagellomere, narrowing basally on F1; protrochanter apical rim posteriorly; apical one-third of mesodistitarsus. Apicoventral rim of metacoxa and metatrochanter brown. Ventral metatibial carina black. Metasoma dark brown, T1-T7 marginal zones yellow-brown to translucent yellow at margins.

*Pubescence*: White, hairs generally short (0.5OD) and sparse, not especially plumose; on lower paraocular area and around antennal base moderately long and moderately dense (1OD); genal beard long and dense (0.5–2OD) longest at midlength; discs of mesoscutum, scutellum, and metanotum mostly bare, few short hairs (0.5OD), longer and denser toward lateral margin as follows: mesoscutum (0.5–1OD), scutellum (1–1.5OD) also on posterior margin, longest posteromedially, metanotum (≤2OD); propodeal hairs moderately long and dense dorsolaterally (0.5–1.5OD); T1-T3 apicolateral patches of tomentum (0.5–1OD); S2 hairs long and dense (1.5OD basolaterad, 0.5OD distilaterad, shorter mesad).

*Surface sculpture*: Microsculpture imbricate, integument generally dull; punctures generally small and deep. Clypeus and supraclypeal area moderately densely punctate (i=1-2d) except supraclypeal area more densely punctate toward lateral margin (i≤d); lower paraocular area densely punctate (i=d); frontal area very densely punctate (i≤0.5d), densely punctate around ocelli (i≤d); ocellocular space moderately densely punctate adjacent to compound eye (i=1-2d) becoming impunctate adjacent to lateral ocellus; vertexal area densely punctate (i≤d); scape shallowly and moderately densely punctate (i=1-2d) densely punctate apicoventrally (i≤d); genal area microstriate and moderately densely punctate (i=1-2d); hypostomal area weakly imbricate and densely punctate (i=d); pronotum densely punctate (i=0.5-1d) lateral surface coarsely imbricate; mesoscutum and scutellum moderately densely punctate (i=1-2d) punctures crowded around median (i≤d); mesepisternum irregularly punctate, sparse below scrobe and anterior of episternal groove (i=1-3d), irregularly spaced in hypoepimeral area (i≤3d); metanotum densely punctate (i=0.5-1d) punctures crowded around median and anteriorly (i≤0.5d); metepisternum densely punctate (i≤d) distinctly longitudinally striate anterodorsally; metapostnotum rugose, minutely so and weakly microsculptured posteriorly; propodeum coarsely imbricate, moderately densely punctate (i=1-2d); T1-T5 coarsely imbricate; T1-T3 moderately densely punctate (i≤2d); T4 moderately densely punctate (i=1-3d); T5 sparsely punctate (i≥2d); T6 sparsely punctate (i≥2d), coarsely imbricate posteriorly; T7 moderately sparsely punctate (i≥2d); marginal zones of terga weakly imbricate and shiny, minutely punctate.

*Structure*: Labrum 3× wider than long (21:7); malar space ~0.3× as long as clypeal lateral (2:7); LOT below anterior tentorial pits; length of clypeus subequal to maximum width in frontal view (29:28) extending for approximately one third of its length beyond LOT; clypeus lacking median longitudinal groove; subantennal sutures ~1.15× as long as the shortest distance between them (14:12); IAD 1.6× AOD (12:7.5); scape ~2.5× as long as maximum width (22:9); pedicel shorter than wide (7:8); F1 shorter than wide (6.5:8.5); F2 ~1.4× as long as F1 and subequal in length to F3 (F2:F3 - 9:9.5); UOD ~1.5× LOD, IOD ~1.1× UOD (UOD:IOD:LOD - 48:52:33); frontal line carinate in lower half, flat above; MOC shorter than width of head (79:84); OOC ~0.67× IOC (12:18); genal area ~0.67× as wide as compound eye in lateral view (18:28); ratio of lengths of mesoscutum: scutellum: metanotum: metapostnotum - 56:20:11:14; probasitarsus 4× as long as maximum depth (20:5); prodistitarsus 0.8× as long as preceding three tarsomeres combined (from II to V - 6:5:4:12); metafemur ~1.75× as long as maximum depth (60:34); summit of metatibial ventral convexity at approximately two-thirds of its length; metatibia ~2.2× as long as maximum depth (54:25); in apical view apical lamina of metatibia reduced, length ~0.5× OD (4:9), short and thick; ventral metatibial carina angled ventrad, somewhat laminate, rounded inflection near midpoint, posterior carina absent basally and originating in apical half of ventral carina; metabasitarsus 3.4× as long as maximum depth (34:10); S1 process apical surface longitudinally concave, in lateral view anterior and posterior margins convergent apically. S7 apodemal arm sclerotized margin terminating laterally at arm midlength; ventral lobe narrow, almost linear; dorsal lobe strap-like, subparallel-sided and somewhat anteriorly curved, with basal tuft of long setae and short apical row, midlength bare. S8 lateral process ~0.9× as wide as long, anteromedial sclerotized margin following marginal contour. Gonobase apicoventral truncate process biconvex and distinctly notched by ~2× width of one convexity.

**Female.** Length 4.5–5.0mm, forewing length 2.4–3.0mm, head width 0.9–1.0mm, thorax width 1.0–1.2mm, median ocellar diameter (OD) 0.09–0.11mm.

*Colouration*: Black to black-brown except as follows: mandible with yellow basal spot, otherwise translucent yellow to yellow except apex translucent brown. Following parts brown: apicoventral margin of pedicel and F1; posterior apical rim of metatibia (yellow-brown on some specimens). Following parts yellow: apicoventral surface of F2; ventral surface of antenna from F3 to terminal flagellomere, suffused with yellow-brown basally; dorsal surface of protibia, narrow basal and apical rings; probasitarsus; apical two-thirds of prodistitarsus; wide basal ring and narrow apical ring on mesotibia; mesobasitarsus ventrally; ventral half of metabasitarsus; apical half of metadistitarsus brown; anterior spot on tegula. Following parts yellow-brown: narrow apical ring on metafemur; wide basal ring on metatibia (yellow on some specimens). Metasoma black, T1-T5 marginal zones yellow-brown to translucent yellow at margins.

*Pubescence*: As in male except as follows: clypeal hairs short and sparse (0.5OD); genal beard sparse (0.5–2OD); discs of mesoscutum, scutellum, and metanotum with sparse short hairs (≤0.5OD), longer and denser toward lateral margin of mesoscutum (0.5OD), and toward lateral and posterior margins of scutellum (1–1.5OD) longest posteromedially; scopae on metafemur and metatibia (1–1.5OD); T1-T3 apicolateral patches of tomentum (0.5–1OD); S1 hairs long and moderately dense (≤2.5OD); S2 scopal hairs (1–3OD).

*Surface sculpture*: As in male except as follows: lower paraocular area moderately densely punctate (i=1-2d); frontal area densely punctate (i≤d), moderately densely punctate around ocelli (i=1-2d); vertexal area moderately densely punctate (i=1-2d); scape shallowly and moderately densely punctate (i=1-2d); genal area weakly imbricate and moderately densely punctate (i=1-2d); hypostomal area weakly imbricate to glossy apically, shallowly and sparsely punctate (i≥2d); mesoscutum and scutellum densely punctate (i=0.5-1d); mesepisternum irregularly punctate, sparse below scrobe (i=2-3d), sparser anterior of episternal groove (i≥3d), irregularly spaced on hypoepimeral area (i≤2d); metanotum moderately densely punctate (i=0.5-2d); metapostnotum rugose or striate-rugose anteriorly, weakly microsculptured posteriorly; propodeum moderately densely punctate (i=1-2d); T1-T3 moderately puncticulate; T4-T5 punctures small and sparse (i≥2d); T6 moderately densely punctate (i=1-2d).

*Structure*: Labrum ~2.5× wider than long (19.5:7.5); malar space ~0.4× as long as clypeal lateral (3:7); LOT below anterior tentorial pits; clypeus subequal in length to maximum width in frontal view (24:26) extending for less than half of its length beyond LOT; median longitudinal groove weak on clypeus; subantennal sutures subequal in length to the shortest distance between them (11.5:11); IAD ~1.6× AOD (11.5:7); scape ~3.33× as long as maximum width (20:6); pedicel longer than wide (8:6.5); F1 subequal in length and width (5.5:5); F2 ~0.67× as long as F1 (3.5) and shorter than F3 (4.5); UOD 1.4× LOD, IOD ~1.05× as long as UOD (UOD:IOD:LOD - 42:45:30); frontal line carinate in lower half, flat above; MOC subequal to width of head (72:69); OOC 0.8× IOC (10:12.5); genal area ~0.6× as wide as eye in lateral view (14:23); ratio of lengths of mesoscutum: scutellum: metanotum: metapostnotum – 48:18:9:12; probasitarsus ~3.5× as long as maximum depth (19:5.5); length of prodistitarsus ~0.9× that of preceding three tarsomeres combined (from II to V - 5:4.5:4:11.5); metafemur ~2.8× as long as maximum depth (39:14); metatibia ~4.5× as long as maximum depth (52:11.5); metabasitarsus ~4.4× as long as maximum depth (31:7).

**Figure 35.**
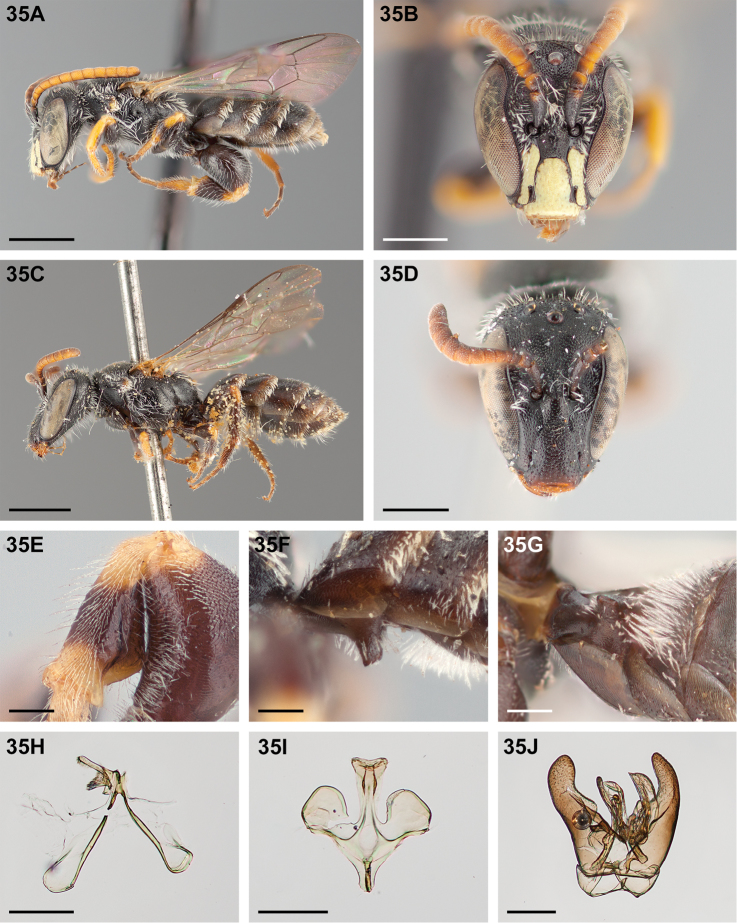
*Chilicola deserticola*: **A** male habitus, lateral view, scale bar 1 mm **B** male head, frontal view, scale bar 0.5 mm **C** female habitus, lateral view, scale bar 1 mm **D** female head, frontal view, scale bar 0.5 mm **E** male metatibia, posterior view, scale bar 0.25 mm **F** male S1 process, lateral view, scale bar 0.25 mm **G** male S1 process, apical view, scale bar 0.25 mm **H** male S7, scale bar 0.25 mm **I** male S8, scale bar 0.25 mm **J** male genital capsule, scale bar 0.25 mm.

###### Material studied

(58 males & 110 females). **Holotype** (male). Region IV, Pueblo Hundido, *S 30.063°, W 70.491°, 1031m*, x.1972, L. Ruz, on *Heliotropium stenophyllum* (AMNH) - see comments; **Region IV**: one paratype male, same data as holotype (AMNH); one female, same locality and date as holotype, V. Cabezas (AMNH); one female, Hwy 41, 2–4 km S of Vicuña, *S 30.067°, W 70.726°, 755m*, 9.x.2001, L. Packer (PCYU); one female, Elqui Prov., 6 km S of Vicuña, *S 30.085°, W 70.725°, 889m*, 16.x.1991, J.G. Rozen & L. Peña (AMNH); six females, Elqui Prov., 7 km S Pisco Elqui, *S 30.177°, W 70.486°, 1449m*, 8.x.1994, Rozen, Quinter, Ascher (AMNH); one male and one female, Elqui Prov., 26 km S of Vicuña, *S 30.19°, W 70.661°, 1691m*, 5.x.1994, Rozen, Quinter, Ascher (AMNH); one female, Elqui, 4–5 km south Pisco, *S 30.383°, W 70.347°, 1340m*, 27.x.1992, A. Sharkov (AMNH); one male, Pr. Coquimbo, Carretera Pan-Am al norte de La Serena, 1970-72, A.R. Moldenke, on *Calandrinia grandiflora*, INT BIOL PROGRAM (AMNH); one male, Pr. Coquimbo, Carretera Pan-Am al norte de La Serena, 21.xi.1970, A.R. Moldenke, INT BIOL PROGRAM #47128 (AMNH); five females, Choros Bajos, *S 29.291°, W 71.309°, 57m*, 12.x.1977, H. Flores (AMNH); one female, same data, PUCV-ENTO 19930 (PUCV); one male, same data, PUCV-ENTO 19926 (PUCV); one female, same locality, 16.x.2000, L. Packer (PCYU); one female, Los Choros, *S 29.291°, W 71.309°, 57m*, 25.x.2002, J. Grixti, A. Zayed, white pan (PCYU); one male and one female, Dunes, Los Choros, S 29.3130°, W 71.2937°, 145m, 13.ix.2010, L. Packer, B09857-A09 Bees of Chile293, B09857-B01 (PCYU); one male, Q. Los Choros, *S 29.343°, W 71.155°, 232m*, 11.x.1977, V. Cabezas (AMNH); one female, same locality and collector, 12.x.1977 (AMNH); one male, Prov. Coquimbo, Río Los Chorros, *S 29.343°, W 71.155°, 232m*, 6.xi.1956, Wagenknecht (PUCV); one female, Carretera Pan-Am al norte de La Serena, *S 29.805°, W 71.287°, 30m*, 15.x.1971, A.R. Moldenke, on *Calandrinia*, INT BIOL PROGRAM 41979 (AMNH); one male and one female, Elqui Prov., 8 km N of La Serena, *S 29.837°, W 71.261°, 16m*, 7.xi.2000, J.G. Rozen (AMNH); one male, La Serena, *S 29.906°, W 71.25°, 20m*, 28.xii.1988, L. Ruz E. (PUCV); one female, Quebrada, El Arrayan, 15 km S La Villa, S 30.071°, W 71.0007°, 400m, 20.xi.2003, F.D. Parker & M.E. Irwin, malaise trap, CH-56 FDP 109843 (BBSL); one male, Elqui Prov., 17 km S Coquimbo, *S 30.088°, W 71.362°, 35m*, 4.x.1994, Rozen, Quinter, Ascher (AMNH); one male, Guanaqueros, *S 30.198°, W 71.422°, 22m*, 7.x.1982, H. Toro (PUCV); one female, same locality and date, E. Chiappa (PUCV); one male, W of Barrancas, *S 30.215°, W 71.329°, 337m*, 10.x.2000, L. Packer (PCYU); one female, Tongoy, *S 30.252°, W 71.5°, 54m*, 5.x.2002, J. Grixti & A. Zayed (PCYU); one female and one male, same locality and collectors, 14.xi.2002, pan traps (PCYU); one male, Tongoy Avda Costanera, *S 30.253°, W 71.494°, 9m*, 25.x.2001, L. Packer & G.S. Fraser (PCYU); one female, same locality and collectors, 26.x.2001 (PCYU); four males and one female, Bahía Tongoy, S of Tongoy, *S 30.272°, W 71.483°, 25m*, 8.x.2001, L. Packer & G.S. Fraser (PCYU); one male, same data, CCDB-22789 D05 (PCYU); four males and six females, Puerto Aldea nr. Tongoy, *S 30.301°, W 71.606°, 9m*, 26.x.2001, Packer & Fraser (PCYU); one male, same locality, 8.xi.2001, L. Packer (PCYU); one wo females, Quebrada Los Litres, SW of Tongoy, inland rd., *S 30.323°, W 71.526°, 16m*, 26.x.2001, L. Packer & G.S. Fraser (PCYU); one male, Las Breas, *S 30.369°, W 70.613°, 1622m*, 11–19.ix.2010, L. Packer (PCYU); one male, P.N. Fray Jorge Entrance, *S 30.627°, W 71.663°, 282m*, 7.x.2001, L. Packer (PCYU); one male, Coquimbo Prov., 4km S of Socos, *S 30.745°, W 71.528°, 201m*, 22.ix.1966, M.E. Irwin (BBSL); one male, Limarí Prov., Talinay Parq Nal., *S 30.867°, W 71.601°, 282m*, 10.x.2001, J.G. Rozen & A. Ugarte, on *Nolana* (AMNH); one male and one female, Choapa: Los Vilos, *S 31.912°, W 71.511°, 54m*, 18.xi.1991, Rozen, Pena, Ugarte, on *Cristaria* (AMNH); one male, Los Vilos, Caleta Los Lobos, *S 31.949°, W 71.526°, 11m*, 26.x.2001, Packer & Fraser (PCYU); one female, Los Vilos, S 31.9312°, W 71.5133°, 12m, 13.xii.2006, L. Packer (PCYU); one female, Bahía Tongoy, S of Tongoy, *S 30.272°, W 71.483°, 25m*, 8.x.2001, Packer & Fraser (PCYU); **Region III**: one male and eleven females, Huasco Prov., Chañar de Aceituno, S 28.9534°, W 71.3472°, 279m, 18.ix.2003, A. Ugarte (PCYU); one female, same data, B10037-H11 Bees of Chile759 (PCYU); one female, 15km W of Domeyko (C-500), S 28.9668°, W 71.0365°, 587m, 15.x.2014, J. Postlethwaite (PCYU); one male and one female, E of Carrizalillo, C-500, km 53.3, S 28.9982°, W 71.3787°, 208m, 8.xi-13.xii.2013, S. Monckton & J. Postlethwaite (PCYU); eight males and seventeen females, Huasco Prov., Chañar de Aceituno, S 29.0279°, W 71.4036°, 279m, 20.ix.2003, A. Ugarte (PCYU); one female, Caleta Chañaral de Aceituna, S 29.09°, W 71.467°, 8m, 18.ix.2003, L. Packer (PCYU); one female, Caleta Chañaral de Aceituna, S 29.0804°, W 71.4672°, 10m, 5.x.2010, L. Packer, G.S. Fraser (PCYU); one male and eleven females, same data, PCYU 0004655, PCYU 0004646, PCYU 0004643, PCYU 0004649, PCYU 0004664, PCYU 0004663, PCYU 0004661, PCYU 0004660, PCYU 0004657, PCYU 0004654, PCYU 0004652, PCYU 0004672 (PCYU); one male and five females, Huasco Prov., Carrizalillo, S 29.0991°, W 71.3912°, 179m, 20.ix.2003, A. Ugarte (PCYU); one male, same data, B10037-H8 Bees of Chile756 (PCYU); five females, Caleta Carrizalillo, S 29.1098°, W 71.4613°, 11m, 8.xi.2013, S. Monckton (PCYU); one female, same data, CCDB-19989 C05 // PCYU 0021691 (PCYU); one female, same locality, 13.x-08.xi.2013, J. Postlethwaite & S. Monckton (PCYU); one male and two females, Caleta Apolillada, S 29.1826°, W 71.4882°, 18m, 8.xi.2013, S. Monckton (PCYU); one female, same locality, 8.xi-13.xii.2013, S. Monckton & J. Postlethwaite (PCYU); four females, 11 km WNW Choros Bajos, *S 29.265°, W 71.411°, 10m*, 14.x.2001, L. Packer (PCYU); four males and two females, Los Choros, *S 29.291°, W 71.309°, 57m*, 25.x.2002, J. Grixti, A. Zayed, yellow pan (PCYU); seven males and two females, SE of Los Choros, near dunes, S 29.3052°, W 71.2836°, 280m, 13.ix.2010, L. Packer (PCYU); one male and one female, same data (CTMI); **Region V**: one female, Aconcagua Prov. Papudo/Zapallar, 5.xi.1971, A.R. Moldenke, on *Chorizanthe vaginata*, INT BIOL PROGRAM 45388 (AMNH).

###### Distribution.

Coquimban & Intermediate Desert, distributed coastally from Caleta Chañaral de Aceituna (Region III) south to Los Vilos (Region IV) and inland along the Elqui Valley to Pueblo Hundido & Guanta (Region IV); 8–1691m a.s.l.

###### Ecology.

Collected on *Calandrinia* Kunth (1 record), *Calandrinia grandiflora* Lindl. (1 record), *Chorizanthe vaginata* (1 record), *Cristaria* Cav. (2 records), *Heliotropium stenophyllum* Hook. & Arn. (3 records) and *Nolana* L. f. (1 record). Recorded September to December.

###### Comments.

The type series (male holotype & one male paratype) is labeled Atacama, Pueblo Hundido, x.1972, L. Ruz, on *Heliotropium stenophyllum*. Atacama is the name of Region III, thus the locality is interpreted as Diego de Almagro (formerly called Pueblo Hundido), and was indicated as such on a map of type localities by Toro and Moldenke (1979). However, besides the type series, *Chilicola deserticola* has not been collected from further north than Carrizal Bajo, Region III, and *Heliotropium stenophyllum* does not occur north of Totoral, Region III (Luebert 2013), while Diego de Almagro is located 1.1° latitude further north than Totoral and in a different biogeographic region (Peña 1966, O’Brien 1971, Artigas 1975). In contrast, Pueblo Hundido is also the name of a village in Region IV (Coquimbo) to the southeast of Vicuña (COPEC 2012, Sernatur 2012); a road sign marks its location at approximately S 30.0629° W 70.4911° (Google Earth 2013). This village is within the range of *Heliotropium stenophyllum* (Luebert 2013) and is the apparent provenance of a specimen collected on that host plant species, labeled Coquimbo, Pueblo Hundido, x.1972, V. Cabezas. Specimen records show that Ruz, Toro, Neff, and Cabezas traveled together in September/October 1972 (see Supplementary File S2); to collect near Diego de Almagro in October of that year would have been inconsistent with their pace and pattern of travel. Moreover, regional mislabeling for locality names that occur in multiple regions is known for the Toro and PUCV collections (L. Ruz, personal communication) where the type material was originally held. Following these considerations, I believe the type locality to be misattributed, and have adjusted it to the village in Region IV accordingly.

##### Chilicola (Heteroediscelis) diaguita

Taxon classificationAnimaliaHymenopteraColletidae

Toro & Moldenke, 1979

**Figure 36.**
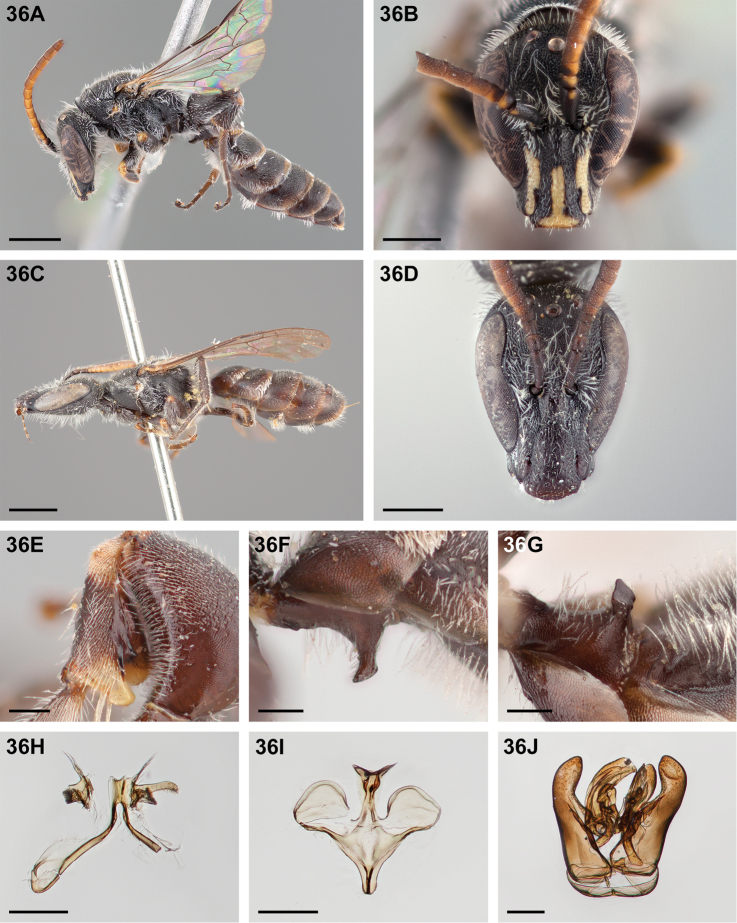
*Chilicola diaguita*: **A** male habitus, lateral view, scale bar 1 mm **B** male head, frontal view, scale bar 0.5 mm **C** female habitus, lateral view, scale bar 1 mm **D** female head, frontal view, scale bar 0.5 mm **E** male metatibia, posterior view, scale bar 0.25 mm **F** male S1 process, lateral view, scale bar 0.25 mm **G** male S1 process, apical view, scale bar 0.25 mm **H** male S7, scale bar 0.25 mm **I** male S8, scale bar 0.25 mm **J** male genital capsule, scale bar 0.25 mm.

Figs 12D–F ; 13A ; 14A–B ; 19A ; 36A–J ; 49

Chilicola diaguita Toro & Moldenke, 1979: 116-117 (male holotype, female allotype, AMNH [examined]). Packer 2008: 77. Montalva and Ruz 2010: 28 (checklist). Moure et al. 2012 (catalogue). Ascher and Pickering 2015 (checklist).

###### Diagnosis.

Males are diagnosable by the combination of malar space 1.5-1.6× as long as the clypeal lateral, apical surface of the S1 process weakly longitudinally concave, and LOT above or just tangent to upper margin of anterior tentorial pits. Females are diagnosable by the combination of protibia with yellow colouration on dorsal surface only in basal half, malar space 1.4–1.6× as long as the clypeal lateral, and LOT above or just tangent to upper margin of anterior tentorial pits. Shorter-faced individuals of both sexes may be confused with *Chilicola vina* and are best distinguished by the position of the LOT, which in *Chilicola vina* passes through the upper margin of the anterior tentorial pits. Additionally, males of *Chilicola diaguita* have the S8 anteromedial sclerotized margin wide and subparallel to margin throughout, not swollen as in *Chilicola vina*.

###### Description.

**Male.** Length 6.0–6.7mm, forewing length 3.5–4.2mm, head width 1.2–1.4mm, thorax width 1.3–1.5mm, median ocellar diameter (OD) 0.13–0.15mm.

*Colouration*: Black-brown, following parts yellow: labrum except apical margin; inverted T shape on clypeus and apex dark laterad; lower paraocular area, extending <1OD above transverse portion of epistomal suture medially, further laterally but not reaching lower tangent of antennal socket; apicoventral spot on scape; apicoventral surface of pedicel; protrochanter apical rim posteriorly; apex of profemur broadly on anterior surface, narrowly on posterior surface; dorsal surface of protibia, including dorsal half of anterior surface; narrow apical ring on mesofemur; narrow basal ring and apicodorsal spot on mesotibia; wide basal ring on metatibia, as well as apical quarter in ventral half only; basal one-third of metabasitarsus ventrally; faint anterior spot on tegula. Ventral surface of antennal flagellum yellow-brown, narrowing basally on F1, and F11 brown. Apical one-third of prodistitarsus brown. Metasoma black, T1-T7 marginal zones translucent yellow.

*Pubescence*: White, hairs generally short (0.5OD) and sparse, not especially plumose; on lower paraocular area and around antennal base long and moderately dense (1.5–2OD); genal beard long and moderately dense (0.5–2OD) longest at midlength; mesoscutal hairs moderately long and sparse, denser toward lateral margin (0.5–1OD); discs of scutellum and metanotum mostly bare, few short hairs (0.5OD), longer and denser toward lateral and posterior margin of scutellum (1–2OD), and toward lateral margin of metanotum (≤2OD); propodeal hairs moderately long and dense dorsolaterally (0.5–2OD); T1-T3 apicolateral patches of tomentum (0.5–1OD); S2 hairs long and dense (1.5OD basolaterad, 1OD distilaterad, shorter mesad).

*Surface sculpture*: Microsculpture imbricate, integument generally dull; punctures generally small and deep. Clypeus and lower paraocular area moderately densely punctate (i=1-2d) except clypeus sparsely punctate medially (i=2-3d); supraclypeal area irregularly punctate (i=1-3d) densely punctate toward lateral margin (i≤d); frontal and vertexal areas very densely punctate (i≤0.5d); ocellocular space densely punctate adjacent to compound eye (i≤d) becoming impunctate adjacent to lateral ocellus; vertexal area rugose; scape shallowly and moderately densely punctate (i=1-2d) densely punctate apicoventrally (i≤d); genal area microstriate and moderately densely punctate (i=1-2d); hypostomal area glossy apically and sparsely punctate (i=1-3d); pronotum densely punctate (i=0.5-1d), lateral surface coarsely imbricate; mesoscutum, scutellum, and metanotum densely punctate (i≤d) except mesoscutal punctures crowded around median (i≤0.5d); mesepisternum irregularly punctate, moderately dense to sparse below scrobe (i=1-2d), sparser anterior of episternal groove (i=2-3d), dense on hypoepimeral area (i≤d); metepisternum densely punctate (i≤d); metapostnotum rugose, more weakly microsculptured posteriorly; propodeum coarsely imbricate, rugulose posteriorly, moderately densely punctate throughout (i=1-2d); T1-T6 coarsely imbricate; T1 sparsely punctate basally (i≥2d) dense apically (i≤d); T2-T5 very densely punctate (i≤d) basal depressed area more coarsely microsculptured but punctures distinct; T6-T7 moderately sparsely punctate (i=1-3d); marginal zones of terga weakly imbricate and shiny, minutely punctate.

*Structure*: Labrum ~2.6× wider than long (28:11); malar space 1.5-1.6× as long as clypeal lateral (15-16:10); LOT above anterior tentorial pits; clypeus ~1.25× as long as maximum width in frontal view (43:35) extending for more than half of its length beyond LOT; median longitudinal groove present on clypeus but weak apically; subantennal sutures ~1.5× as long as the shortest distance between them (23:15); IAD ~1.6× AOD (14:9); scape ~2.4× as long as maximum width (29:12); pedicel shorter than wide (10:11); F1 shorter than wide (10:11); F2 1.4× as long as F1 and shorter than F3 (F2:F3 - 14:15.5); UOD ~1.4× LOD, IOD ~1.1× UOD (UOD:IOD:LOD - 57:63:40); frontal line carinate between antennal bases for a length of ~0.5OD, flat just below median ocellus for a length of ~1OD, otherwise undefined; MOC ~1.1× as long as width of head (111:101); OOC ~0.6× IOC (13:21); genal area ~0.6× as wide as compound eye in lateral view (20:35); ratio of lengths of mesoscutum: scutellum: metanotum: metapostnotum - 75:26:14.5:19; probasitarsus ~3.9× as long as maximum depth (31:8); length of prodistitarsus ~0.8× that of preceding three tarsomeres combined (from II to V - 8:6:5:16); metafemur ~1.85× as long as maximum depth (84:45); summit of metatibial ventral convexity near tibial apex; metatibia ~1.75× as long as maximum depth (61:35); in apical view apical lamina of metatibia broad and thick, length ~0.9× OD (10:11); ventral metatibial carina toothed near apex, posterior carina sublinear and weakly defined basally; metabasitarsus 4.25× as long as maximum depth (51:12); S1 process apical surface longitudinally concave at least posteriorly, in lateral view anterior and posterior margins divergent apically, apex appearing anteriorly bent. S7 apodemal arm sclerotized margin terminating laterally at arm midlength; ventral lobe narrow, almost linear; dorsal lobe strap-like, subparallel-sided and somewhat anteriorly curved, with basal tuft of long setae and a short apical row, midlength bare; apex of disc sinuate. S8 lateral process ~1.2× wider than long, anteromedial sclerotized margin following marginal contour. Gonobase apicoventral truncate process biconvex and distinctly notched by a width less than one convexity.

**Female.** Length 5.3–6.6mm, forewing length 3.3–3.5mm, head width 1.1–1.2mm, thorax width 1.2–1.4mm, median ocellar diameter (OD) 0.12mm.

*Colouration*: Black to black-brown except as follows: mandible with yellow basal spot, translucent yellow in apical half except apex translucent brown. Following parts brown: pedicel, F1 and F2, on apicoventral margins. Following parts variably yellow to yellow-brown: ventral surface of F3-F10, each suffused with brown basally; dorsal surface of protibia in basal half; apical half of prodistitarsus; basidorsal spot on mesotibia; faint anterior spot on tegula. Metasoma black, T1-T5 marginal zones translucent yellow.

*Pubescence*: As in male except as follows: clypeal hairs short and sparse (0.5OD); on lower paraocular area and around antennal base moderately long and moderately dense (1OD); genal beard sparse (0.5–2OD); discs of mesoscutum, scutellum, and metanotum with sparse short hairs (≤0.5OD), longer and denser toward lateral margin as follows: mesoscutum (0.5OD), scutellum (1–1.5OD) also on posterior margin, longest posteromedially, metanotum (≤2OD); propodeal hairs moderately long and dense dorsolaterally (0.5–1.5OD); scopae on metafemur and metatibia (1–1.5OD); T1-T2 apicolateral patches of tomentum (0.5–1OD), T3 apicolateral hair band sparse, not tomentose; S1 hairs long and moderately dense (≤2.5OD); S2 scopal hairs (1–3OD).

*Surface sculpture*: As in male except as follows: frontal and vertexal areas densely punctate (i≤d); scape shallowly and moderately densely punctate (i=1-2d); genal area weakly microstriate and moderately densely punctate (i=1-2d); hypostomal area weakly imbricate to glossy apically, shallowly and moderately densely punctate (i=1-2d); mesoscutum densely punctate (i=0.5-1d) crowded around median (i≤d); scutellum densely punctate (i≤d); mesepisternum irregularly punctate, sparse below scrobe (i=1-3d), sparser anterior of episternal groove (i=2-3d), denser on hypoepimeral area (i≤2d); metanotum densely punctate (i≤d); metapostnotum rugose, more weakly microsculptured posteriorly; propodeum moderately densely punctate (i=1-2d); T1 punctures small and sparse (i≥2d basally; i=1-3d apically); T2-T5 punctures small and sparse (i=1-3d on T2-T3; i≥2d on T4-T5); T6 moderately densely punctate (i=1-2d).

*Structure*: Labrum 2.6× wider than long (26:10); malar space ~1.4-1.6× as long as clypeal lateral (12-13.5:8.5); LOT crossing above anterior tentorial pits; clypeus ~1.3× as long as maximum width in frontal view (40:31) extending for more than half of its length beyond LOT; clypeus median longitudinal groove weakly present at least in dorsal half; subantennal sutures ~1.2× as long as the shortest distance between them (17:14); IAD ~1.4× AOD (12:8.5); scape ~3.1× as long as maximum width (25:8); pedicel longer than wide (9:8); F1 longer than wide (8:7); F2 shorter than F1 and shorter than F3 (F2:F3 - 5:6); UOD ~1.33× LOD, IOD 1.1× as long as UOD (UOD:IOD:LOD - 50:55:37); frontal line carinate in lower third, flat above; MOC ~1.2× as long as width of head (99:84); OOC ~0.6× IOC (11:19); genal area ~0.65× as wide as eye in lateral view (18:28); ratio of lengths of mesoscutum: scutellum: metanotum: metapostnotum – 62:23:11:15; probasitarsus ~3.7× as long as maximum depth (26:7); length of prodistitarsus ~0.9× that of preceding three tarsomeres combined (from II to V - 6.5:6:4.5:15); metafemur 3× as long as maximum depth (54:18); metatibia ~4.25× as long as maximum depth (64:15); metabasitarsus ~4.3× as long as maximum depth (41:9.5).

###### Material studied

(8 males & 22 females): **Holotype** (male): Region IV, La Serena, *S 29.906°, W 71.250°, 20m*, 28.ii.1956, Moure (AMNH); **Allotype** (female): same locality as holotype, 20.iii.1952, Wagenknecht (AMNH); **Region IV**: one male, Choros Bajos, *S 29.291°, W 71.309°, 57m*, x.1977, De La Hoz (PUCV); one male, same locality, 12.x.1977, H. Flores (AMNH); one female, same locality and date, Magunacelaya (PUCV); four females, Q. Los Choros, *S 29.343°, W 71.155°, 232m*, 12.x.1977, V. Cabezas (PUCV); three females, Cuesta Buenos Aires, S 29.5624°, W 71.2530°, 518m, 8.xi.2013, S. Monckton (PCYU); two females, same data (CTMI); one male, same data, CCDB-19989 B09 // PCYU 0021683 (PCYU); one male and one female, same locality, Cuesta Buenos Aires, 13.x-8.xi.2013, S. Monckton, pan traps, CCDB-22789 D07, CCDB-22789 D10 (PCYU); two females, 1.1 km S on Rd to Tololo Observatory, S 30.0358°, W 70.8158°, 543m, 22.x.2009, J.Gibbs (PCYU); two males and four females, Vicuña, Agric. Exp. Station, S 30.036°, W 70.6913°, 650m, 5.xi.2003, F.D. Parker & M.E. Irwin, pan traps, CH-56 FDP 110911, CH-56 FDP 111827, CH-56 FDP 110929, CH-56 FDP 111935, CH-56 FDP 111836, CH-56 FDP 111822 (BBSL); one male, Quebrada, El Arrayan, 33 km S La Villa, S 30.1952°, W 70.9338°, 653m, 1-8.xi.2003, F.D. Parker & M.E Irwin, malaise trap, CH-56 FDP 109942 (BBSL); one female, Quebrada, El Arrayan, 15 km S La Villa, S 30.071°, W 71.0007°, 400m, 20.xi.2003, F.D. Parker & M.E. Irwin, malaise trap, CH-56 FDP 109833 (BBSL); one female, Quebrada, El Arrayan, 15 km S La Villa, S 30.1045°, W 70.9902°, 472m, 1.xi.2003, F.D. Parker & M.E. Irwin, pan trap, CH-11 FDP 111580 (BBSL); one female, Elqui Prov., Pangue, S 30.1539°, W 70.6639°, 1686m, 11-30.ix.2004, A. Ugarte (PCYU); one female, 1.1 km S on road to Tololo Obs., S 30.3058°, W 70.8151°, 1069m, 11-22.x.2009, J. Gibbs, CHL-09:134 (PCYU).

###### Distribution.

Coquimban–Intermediate Desert transition zone, from Choros Bajos southeast to Tololo Observatory (Region IV); 20–1686m a.s.l.

###### Ecology.

Recorded September to November and February to March.

##### Chilicola (Heteroediscelis) erithropoda

Taxon classificationAnimaliaHymenopteraColletidae

Toro & Moldenke, 1979

**Figure 37.**
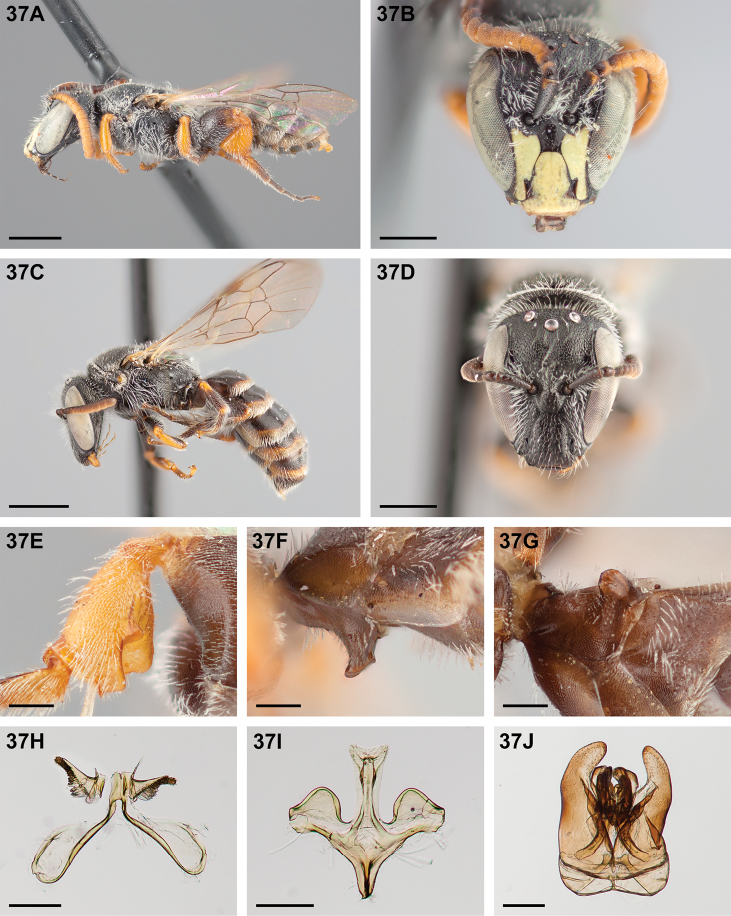
*Chilicola erithropoda*: **A** male habitus, lateral view, scale bar 1 mm **B** male head, frontal view, scale bar 0.5 mm **C** female habitus, lateral view, scale bar 1 mm **D** female head, frontal view, scale bar 0.5 mm **E** male metatibia, posterior view, scale bar 0.25 mm **F** male S1 process, lateral view, scale bar 0.25 mm **G** male S1 process, apical view, scale bar 0.25 mm **H** male S7, scale bar 0.25 mm **I** male S8, scale bar 0.25 mm **J** male genital capsule, scale bar 0.25 mm.

Figs 2C–D ; 30C–D ; 37A–J ; 49

Chilicola erithropoda Toro & Moldenke, 1979: 127 (male holotype, AMNH [examined]). Packer 2008: 77. Montalva and Ruz 2010: 28 (checklist). Moure et al. 2012 (catalogue). Ascher and Pickering 2015 (checklist).

###### Diagnosis.

Males are separated - along with *Chilicola travesia* - by the tibiae and tarsi mostly or entirely yellow-orange, and differentiated from the latter by MOC less than 0.95× width of the head, and apical surface of S1 process longitudinally concave except for a median anterior convexity. Females are separated from all other species of the subgenus except *Chilicola travesia* by the combination of malar space less than 2/3× as long as the clypeal lateral, metatibia expanded (>0.25× as deep as long), and T3 with apicolateral patches of tomentum as on T1-T2. Females of *Chilicola erithropoda* are differentiated from those of *Chilicola travesia* by the hind tibia mostly dark (at most basal one-third and narrow apical band yellow) and the probasitarsus mostly yellow-brown to dark brown (at most basal and apical quarter yellow) – both are mostly or entirely yellow in the latter species.

###### Description.

**Male.** Length 5.5–6.8mm, forewing length 3.4–3.9mm, head width 1.3–1.5mm, thorax width 1.3–1.5mm, median ocellar diameter (OD) 0.13–0.15mm.

*Colouration*: Black-brown except as follows: antenna variable (scape with yellow apicoventral spot to entirely dark; apicoventral surface of pedicel yellow to brown; ventral surface of flagellum yellow to yellow-brown, narrowing basally on F1). Following parts yellow: labrum; clypeus except narrowly dark at mid-length along epistomal suture and apex often translucent brown laterad; lower paraocular area, extending more than 1OD above transverse portion of epistomal suture medially and reaching lower tangent of antennal socket laterally; protrochanter apical rim posteriorly; apical one-third to half of profemur on anterior surface, narrowly on posterior surface; protibia; protarsus; apex of mesofemur broadly on anterior surface, narrowly on posterior surface; mesotibia; mesobasitarsus variable from brown to yellow; apical half of mesodistitarsus; broad apical ring on metafemur; metatibia except ventral and posterior carinae brown; metabasitarsus ventrally, sometimes dorsally in basal half; anterior spot on tegula; apex of T7. Apicoventral rim of metacoxa and metatrochanter yellow-brown. Metasoma dark brown, T1-T6 marginal zones yellow-orange to translucent yellow at margins.

*Pubescence*: White, hairs generally short (0.5OD) and sparse, not especially plumose; on lower paraocular area and around antennal base moderately long and moderately dense (1OD); genal beard long and dense (0.5–2.5OD) longest at midlength; mesoscutal hairs moderately long and sparse (1–1.5OD) shorter and denser toward lateral margin (0.5–1OD); discs of scutellum and metanotum mostly bare, few short hairs (0.5OD), longer and denser toward lateral and posterior margins of scutellum (1–2OD) longest posteromedially, and toward lateral margin of metanotum (≤2.5OD); propodeal hairs moderately long and dense dorsolaterally (0.5–2OD); T1-T3 apicolateral patches of tomentum (0.5–1OD); S2 hairs moderately long and moderately sparse (1OD basolaterad, 0.5OD distilaterad, bare mesad).

*Surface sculpture*: Microsculpture imbricate, integument generally dull; punctures generally small and deep. Clypeus and supraclypeal and vertexal areas moderately densely punctate (i=1-2d) except clypeus irregularly punctate in upper half (i=1-3d), supraclypeal area densely punctate toward lateral margin (i≤d); lower paraocular area irregularly punctate (i=1-3d); frontal area very densely punctate (i≤0.5d), densely punctate around ocelli (i≤d); ocellocular space moderately densely punctate (i=1-2d); scape shallowly and moderately densely punctate (i=1-2d) densely punctate apicoventrally (i≤d); genal area microstriate and moderately densely punctate (i≤d); hypostomal area weakly imbricate and densely punctate (i≤d); pronotum densely punctate (i=0.5-1d), lateral surface coarsely imbricate; mesoscutum densely punctate (i=0.5-1d) punctures crowded around median (i≥0.5d); scutellum moderately densely punctate (i=0.5-2d); mesepisternum irregularly punctate, sparse below scrobe and anterior of episternal groove (i=1-2d), impunctate just dorsad of scrobe, otherwise irregularly spaced on hypoepimeral area (i≤2d); metanotum densely punctate (i=0.5-2d) punctures crowded around median and anteriorly (i≤0.5d); metepisternum densely punctate (i≤d); metapostnotum rugose, minutely so posteriorly; propodeum coarsely imbricate, moderately densely punctate (i=1-2d); T1-T5 coarsely imbricate; T1-T3 moderately densely punctate (i≤2d); T4 moderately punctate-puncticulate (i=1-2d); T5 moderately punctate-puncticulate (i=1-3d); T6 sparsely punctate-puncticulate (i≥2d) and coarsely imbricate posteriorly; T7 sparsely punctate-puncticulate (i≥2d); marginal zones of terga weakly imbricate and shiny, minutely punctate.

*Structure*: Labrum ~2.5× wider than long (28:11); malar space 0.4× as long as clypeal lateral (4:10); LOT below anterior tentorial pits; length of clypeus subequal to maximum width in frontal view (37:37) extending for less than half of its length beyond LOT; clypeus lacking median longitudinal groove; subantennal sutures ~1.4× as long as the shortest distance between them (18:13); IAD ~1.5× AOD (11:16); scape ~2.67× as long as maximum width (29:11); pedicel as long as wide (9:9); F1 shorter than wide (8.5:9.5); F2 slightly shorter than F1 and shorter than F3 (F2:F3 - 8:10); UOD ~1.3× LOD, IOD ~1.1× UOD (UOD:IOD:LOD - 59:64:46); frontal line carinate in lower half, flat above; MOC shorter than width of head (96:105); OOC ~0.5× IOC (12:23); genal area ~0.6× as wide as compound eye in lateral view (21:36); ratio of lengths of mesoscutum: scutellum: metanotum: metapostnotum - 68:26:12:17; probasitarsus ~3.9× as long as maximum depth (27:7); length of prodistitarsus ~0.8× that of preceding three tarsomeres combined (from II to V - 8:6:5:15); metafemur ~1.5× as long as maximum depth (69:45); summit of metatibial ventral convexity situated at approximately two-thirds of its length; metatibia ~1.67× as long as maximum depth (69:42); in apical view apical lamina of metatibia short and thick, length ~1.1× OD (12:11); ventral metatibial carina bluntly toothed near midpoint and deeply concave apically, posterior carina absent basally and originating at level of tooth on ventral carina; metabasitarsus ~3× as long as maximum depth (46:15.5); S1 process apical surface concave posteriorly and convex anteromedially, in lateral view anterior and posterior margins divergent apically, apex appearing strongly anteriorly bent. S7 apodemal arm sclerotized margin entire, enclosing membranous portion of arm; ventral lobe ~0.25× as broad as basal attached length; dorsal lobe strap-like, subparallel-sided and somewhat anteriorly curved, with row of setae long basally and shorter apically; apex of disc disinctly emarginate. S8 lateral process ~1.1× wider than long, apicolateral margin distinctly concave, anteromedial sclerotized margin following marginal contour. Gonobase apicoventral truncate process biconvex and distinctly notched by ~2× width of one convexity.

**Female.** Length 5.2–6.6mm, forewing length 3.2–3.7mm, head width 1.2–1.4mm, thorax width 1.3–1.5mm, median ocellar diameter (OD) 0.12–0.13mm.

*Colouration*: Black to black-brown except as follows: labrum entirely black or with yellow-brown apex; mandible with yellow basal spot, otherwise translucent yellow with apex translucent brown; antenna variable (scape dark or with brown apicoventral spot; apicoventral rim of pedicel yellow-brown to brown; apicoventral spot on F1 and F2 yellow-brown to brown; ventral surface of flagellum from F3 to terminal flagellomere yellow to yellow-brown). Following parts yellow: protibia except dark medioventrally; probasitarsus at most in basal and apical quarter; prodistitarsus; wide basal and apical rings on mesotibia; mesobasitarsus variable from brown to yellow ventrally; apical half of mesodistitarsus; narrow apicodorsal rim on metafemur; metatibia at most in basal one-third and narrow apical band; metabasitarsus ventrally; apical half of metadistitarsus yellow to yellow-brown; anterior spot on tegula. Metasoma black, T1-T5 marginal zones yellow to translucent yellow at margins.

*Pubescence*: As in male except as follows: clypeal hairs short and sparse (0.5OD); genal beard sparse (0.5–2.5OD); discs of mesoscutum, scutellum, and metanotum with sparse short hairs (≤0.5OD) interspersed with sparse moderately long hairs (1OD), denser and as long or longer toward lateral margin as follows: mesoscutum (0.5–1OD); scutellum (1–1.5OD) also on posterior margin, longest posteromedially, metanotum (≤2OD); propodeal hairs moderately long and dense dorsolaterally (0.5–1.5OD); scopae on metafemur and metatibia (1–1.5OD); T1-T3 apicolateral patches of tomentum (0.5–1OD); S1 hairs long and moderately dense (≤2.5OD); S2 scopal hairs (1–2.5OD).

*Surface sculpture*: As in male except as follows: clypeus and lower paraocular area moderately densely punctate throughout (i=2d); supraclypeal area irregularly punctate (i=1-3d) densely punctate toward lateral margin (i≤d); frontal and vertexal areas moderately densely punctate (i=1-2d); scape shallowly and densely punctate (i=d); genal area microstriate and densely punctate (i=d); hypostomal area weakly imbricate, shallowly and moderately densely punctate (i=1-2d); mesoscutum, scutellum, and metanotum densely punctate (i=0.5-1d) except scutellar punctures crowded around median (i≤d), metanotal punctures crowded around median and anteriorly (i≤d); mesepisternum irregularly punctate, moderately dense below scrobe and anterior of episternal groove (i=1-2d), dense on hypoepimeral area (i≤d); metapostnotum rugose, more weakly microsculptured posteriorly; propodeum coarsely imbricate, rugulose posteriorly, moderately densely punctate throughout (i=1-2d); T1 impunctate basally, punctures small and sparse apically (i=1-3d); T2-T5 punctures small and sparse (i=1-3d); T6 moderately densely punctate (i=1-2d).

*Structure*: Labrum ~3× wider than long (28:9.5); malar space ~0.6× as long as clypeal lateral (4.5:8); LOT below anterior tentorial pits; clypeus ~1.1× as long as maximum width in frontal view (38:34) extending for approximately one third of its length beyond LOT; median longitudinal groove weak on clypeus; subantennal sutures ~1.1× as long as the shortest distance between them (14:13); IAD 1.5× AOD (15:10); scape ~3.1× as long as maximum width (28:6); pedicel longer than wide (9:8); F1 as long as wide (7.5:7.5); F2 shorter than F1 and slightly shorter than F3 (F2:F3 - 4.5:5); UOD ~1.3× LOD, IOD ~1.1× as long as UOD (UOD:IOD:LOD - 54:60:42); frontal line carinate in lower half, flat above; MOC shorter than width of head (91.5:96.5); OOC ~0.5× IOC (10.5:20); genal area ~0.67× as wide as eye in lateral view (20:31); ratio of lengths of mesoscutum: scutellum: metanotum: metapostnotum – 62:26:11:13; probasitarsus ~3.1× as long as maximum depth (22:7); length of prodistitarsus ~0.9× that of preceding three tarsomeres combined (from II to V - 7:6:4.5:15); metafemur ~2.6× as long as maximum depth (53:20.5); metatibia ~3.8× as long as maximum depth (66:17.5); metabasitarsus ~3.9× as long as maximum depth (41:10.5).

###### Material studied

(19 males & 22 females): **Holotype** (male): Region IV, Pueblo Hundido, *S 30.063°, W 70.491°, 1031m*, x.1972, L. Ruz, on *Heliotropium stenophyllum* (AMNH) - see comments; **Region IV**: one male and three females, 11 km WNW of Choros Bajos, *S 29.265°, W 71.411°, 10m*, 14.x.2001, L. Packer (PCYU); one male, Los Choros, *S 29.291°, W 71.309°, 57m*, 25.x.2002, J. Grixti & A. Zayed (PCYU); three females, same data, yellow pan (PCYU); one female, same data, bright yellow pan (PCYU); one female, Dunes, Los Choros, S 29.3130°, W 71.2937°, 145m, 13.ix.2010, L. Packer, B09857-A06 Bees of Chile290 (PCYU); **Region III**: one male and one female, Huasco Prov., Chañar de Aceituno, S 28.9534°, W 71.3472°, 279m, 18.ix.2003, A. Ugarte (PCYU); one male, same data (AMNH); one male and one female, same data (BBSL); one male and one female, Huasco Prov., Chañar de Aceituno, S 29.0279°, W 71.4036°, 279m, 20.ix.2003, A. Ugarte (CTMI); one male, same data (AMNH); one male, Caleta Chañaral de Aceituna, S 29.09°, W 71.467°, 8m, 18.ix.2003, L. Packer (PCYU); four males and four females, Caleta Chañaral de Aceituna, S 29.0904°, W 71.4672°, 10m, 5.x.2010, L. Packer & G.S. Fraser, PCYU 0004665, PCYU 0004656, PCYU 0004666, PCYU 0004659, PCYU 0004648, PCYU 0004662, PCYU 0004658, PCYU 0004650 (PUCV); one male and two females, Huasco prov., Carrizalillo, *S 29.099°, W 71.391°, 179m*, 20.ix.2003, A. Ugarte, B10037-H10 Bees of Chile758, B10037-H9 Bees of Chile757, B10037-H07 Bees of Chile755 (PCYU); three females, Caleta Carrizalillo, S 29.1098°, W 71.4613°, 11m, 8.xi.2013, S. Monckton (PCYU); one female, same data, pan traps, CCDB-19989 C06 // PCYU 0021692 (PCYU); four males, 11km WNW Choros Bajos, *S 29.265°, W 71.411°, 10m*, 14.x.2001, L. Packer (PCYU); one male, same data, CCDB-03755 D02 (PCYU); one female, same locality and collector, 16.x.2000 (PCYU).

###### Distribution.

Coastal, southern Intermediate Desert, from Chañaral de Aceituna (Region III) south to Los Choros (Region IV), with type locality disjunct from otherwise observed distribution; 8–1031m a.s.l.

###### Ecology.

Collected on *Heliotropium stenophyllum* (1 record). Recorded September to November.

###### Comments.

The holotype is labeled Atacama, Pueblo Hundido, x.1972, L. Ruz, on *Heliotropium stenophyllum*. This refers to the renamed village of Diego de Almagro (Region III), but is inconsistent with the otherwise-documented distribution of *Chilicola erithropoda*, as well as the known range of *Heliotropium stenophyllum* (Luebert 2013). Following evidence presented in the comments on *Chilicola deserticola* above, I believe the type locality to be misattributed, and have adjusted it to the village of Pueblo Hundido in Region IV accordingly.

##### Chilicola (Heteroediscelis) guanicoe

Taxon classificationAnimaliaHymenopteraColletidae

Monckton sp. n.

http://zoobank.org/D6EA94C8-BED2-4A7F-8019-7F9A70BA2996

**Figure 38.**
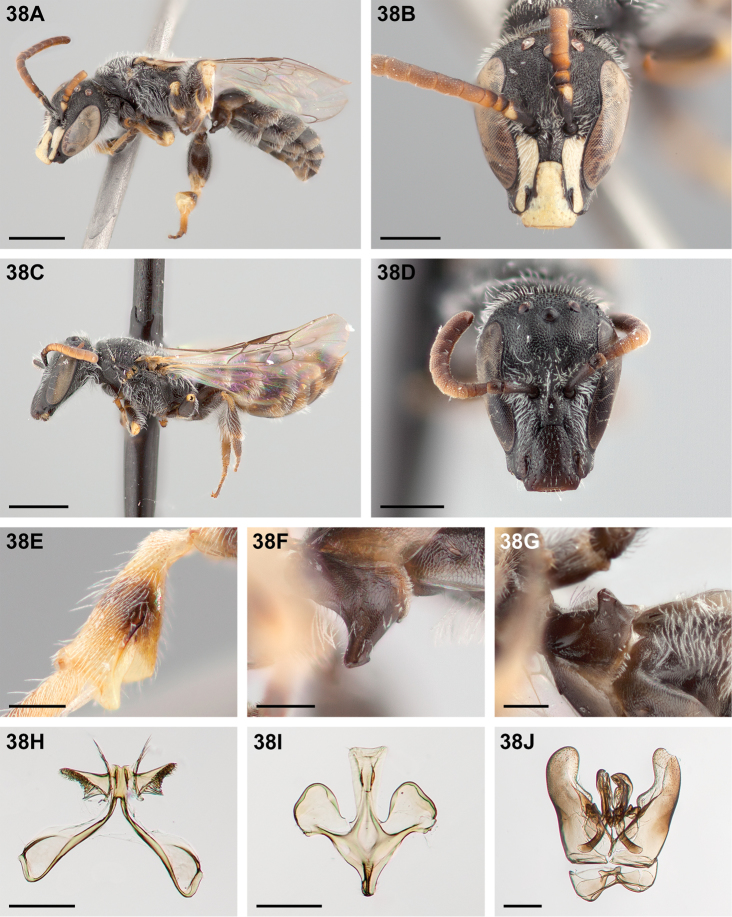
*Chilicola guanicoe*: **A** male habitus, lateral view, scale bar 1 mm **B** male head, frontal view, scale bar 0.5 mm **C** female habitus, lateral view, scale bar 1 mm **D** female head, frontal view, scale bar 0.5 mm **E** male metatibia, posterior view^†^, scale bar 0.25 mm **F** male S1 process, lateral view, scale bar 0.25 mm **G** male S1 process, apical view^†^, scale bar 0.25 mm **H** male S7 (C.gnco.007), scale bar 0.25 mm **I** male S8 (C.gnco.007), scale bar 0.25 mm **J** male genital capsule (C.gnco.007), scale bar 0.25 mm.

Figs 6A ; 7B ; 21A ; 38A–J ; 49

###### Diagnosis.

Males are diagnosable by the combination of malar space 1.4–2× as long as the clypeal lateral, and apical surface of S1 process with a longitudinal median ridge. Males share many characters with *Chilicola vicugna* and *Chilicola mavida*, but are differentiable by the longer face and weakly toothed posterior carina on the metatibia. Females are diagnosable by the combination of malar space 1.2–1.7× as long as the clypeal lateral, and dorsal surface of the protibia yellow along its entire length.

###### Description.

**Male (Holotype).** Length 5.5mm, forewing length 3.3mm, head width 1.1mm, thorax width 1.2mm, median ocellar diameter (OD) 0.14mm.

*Colouration*: Black-brown, following parts yellow: labrum; clypeus except narrowly dark brown along epistomal suture and apex yellow along entire width; lower paraocular area extending more than 1OD above transverse epistomal suture, nearly reaching lower tangent of antennal socket; apicoventral surface of scape; apex of profemur broadly on anterior surface, narrowly on posterior surface; dorsal surface of protibia, including anterior surface in dorsal half, with ventral half translucent yellow; probasitarsus; apicodorsal rim of mesofemur, wider anteriorly; wide basal and apical rings on mesotibia, as well as apical half of anterior surface; mesobasitarsus, suffused with brown distad; apicoventral rim of metacoxa; wide apical ring on metafemur; metatibia except black posterior carina, medial dark patch on posterior surface, dark medial quadrangle on anterior surface covering approximately half of total length; basal half of metabasitarsus and ventrally in apical half; anterior spot on tegula; apex of T7. Following parts yellow-brown: apicoventral surface of pedicel; ventral surface of F1, narrowing basally; ventral surface of antenna from F2 to terminal flagellomere, suffused with darker brown; protrochanter apical rim posteriorly; apical half of prodistitarsus; apicoventral rim of mesocoxa; apical half of mesodistitarsus; apicoventral rim of metatrochanter. Metasoma black, T1-T6 marginal zones amber to translucent yellow at margins.

*Pubescence*: White, hairs generally short (0.5OD) and sparse, not especially plumose; on lower paraocular area and around antennal base moderately long and moderately dense (1OD); genal beard long and dense (0.5–2OD) longest at midlength; mesoscutal hairs moderately long and sparse, denser toward lateral margin (0.5–1OD); discs of scutellum and metanotum mostly bare, few short hairs (0.5OD), longer and denser toward lateral and posterior margins of scutellum (1–1.5OD) longest posteromedially, and toward lateral margin of metanotum (≤2OD); propodeal hairs moderately long and dense dorsolaterally (0.5–1.5OD); T1-T3 apicolateral patches of tomentum (0.5–1OD); S2 hairs long and dense (1.5OD basolaterad, 0.5OD distilaterad, shorter mesad).

*Surface sculpture*: Microsculpture imbricate, integument generally dull; punctures generally small and deep. Clypeus and lower paraocular area moderately densely punctate (i=1-2d) except clypeus sparsely punctate medially (i≥2d); supraclypeal area irregularly punctate (i=1-3d) densely punctate toward lateral margin (i≤d); frontal area very densely punctate (i≤0.5d); ocellocular space densely punctate adjacent to compound eye (i≤d) becoming impunctate adjacent to lateral ocellus; vertexal area densely punctate (i≤d); scape shallowly and moderately densely punctate (i=1-2d); genal area microstriate and moderately densely punctate (i=1-2d); hypostomal area rough, weakly imbricate and moderately densely punctate (i=1-2d); pronotum moderately densely punctate (i=1-2d), lateral surface coarsely imbricate; mesoscutum densely punctate (i=0.5-1d) punctures crowded around median (i≤d); scutellum moderately densely punctate (i=0.5-2d) punctures crowded around median (i≤d); mesepisternum irregularly punctate, moderately dense to sparse below scrobe (i=1-2d), sparser anterior of episternal groove (i=1-3d), impunctate just dorsad of scrobe, otherwise moderately dense on hypoepimeral area (i≤2d); metanotum moderately densely punctate (i=1-2d) punctures crowded around median and anteriorly (i≤d); metepisternum densely punctate (i≤d); metapostnotum rugose, fewer transverse lineations and more weakly microsculptured posteriorly; propodeum rugulose posteriorly, moderately densely punctate throughout (i=1-2d); T1-T5 coarsely imbricate and moderately densely punctate (i=1-2d on T1-T4; i=1-3d on T5); T2-T5 basal depressed area more coarsely microsculptured but punctures distinct; T6 moderately densely punctate (i=1-3d) and coarsely imbricate posteriorly; T7 moderately densely punctate (i=1-3d); marginal zones of terga weakly imbricate and shiny, minutely punctate.

*Structure*: Labrum ~2.4× wider than long (22:9); malar space ~1.4× as long as clypeal lateral (11:8); LOT just above anterior tentorial pits; clypeus ~1.1× as long as maximum width in frontal view (34:30) extending for more than half of its length beyond LOT; median longitudinal groove on clypeus weakly present in dorsal half, absent ventrad; subantennal sutures ~1.4× as long as the shortest distance between them (16:11.5); IAD 2× AOD (12:6); scape 2.4× as long as maximum width (24:10); pedicel shorter than wide (7.5:8.5); F1 slightly shorter than wide (8.5:9); F2 ~1.5× as long as F1 and shorter than F3 (F2:F3 - 12.5:14); UOD ~1.5× LOD, IOD ~1.1× UOD (UOD:IOD:LOD - 48:52:33); frontal line carinate between antennal bases for a length less than 0.5OD, flat just below median ocellus for less than 1OD, otherwise undefined; MOC longer than width of head (88:83); OOC ~0.67× IOC (12:18); genal area ~0.6× as wide as compound eye in lateral view (18:29); ratio of lengths of mesoscutum: scutellum: metanotum: metapostnotum - 60:21:9:14; probasitarsus ~3.8× as long as maximum depth (25:6.5); length of prodistitarsus subequal to that of preceding three tarsomeres combined (from II to V - 6:5:4:14); metafemur almost twice as long as maximum depth (59:30); summit of metatibial ventral convexity at approximately two-thirds tibial length and rounded; metatibia ~2.1× as long as maximum depth (53:25); in apical view apical lamina of metatibia short and thick, length 0.7× OD (7:10); ventral metatibial carina bowed ventrad and absent basally, originating at midpoint of posterior carina, the latter sharply inflected near base and somewhat concave apicad of inflection; metabasitarsus ~4.2× as long as maximum depth (38:9); S1 process apical surface with a longitudinal median ridge, in lateral view anterior and posterior margins convergent apically and median ridge projecting posteriorly from apex. S7 apodemal arm sclerotized margin recurved mesad, dividing membranous portion of arm; ventral lobe narrow, almost linear; dorsal lobe roughly triangular, wide at base and narrow apically, with row of setae long basally and shorter apically; apex of disc disinctly emarginate. S8 lateral process ~0.9× as wide as long, anteromedial sclerotized margin wide and weakly convex. Gonobase apicoventral truncate process biconvex and distinctly notched by ~1.5× width of one convexity.

**Female (Allotype).** Length 5.0mm, forewing length 3.1mm, head width 1.0mm, thorax width 1.1mm, median ocellar diameter (OD) 0.12mm.

*Colouration*: Black to black-brown except as follows: labrum pale to translucent yellow along basal margin; mandible with yellow basal spot and pale to translucent yellow except apex translucent brown. Following parts yellow-brown: apicoventral surface of pedicel and F1; ventral surface of antenna from F2 to terminal flagellomere, narrowing basally on F2; mesobasitarsus ventrally; apical half of mesodistitarsus; wide apical ring on metatibia; posterior surface of metabasitarsus. Following parts yellow: dorsal surface and narrow apical ring on protibia; anterior surface of probasitarsus and second to fourth tarsomeres; apical half of prodistitarsus; wide basal and narrow apical ring on mesotibia; narrow apical ring on metafemur; wide basal ring on metatibia; anterior spot on tegula. Apical one-third of metadistitarsus brown. Metasoma brown, T1-T5 marginal zones amber to translucent yellow at margins.

*Pubescence*: As in male except as follows: clypeal hairs short and sparse (0.5OD); genal beard sparse (0.5–2OD); discs of mesoscutum, scutellum, and metanotum with sparse short hairs (≤0.5OD), longer and denser toward lateral margin as follows: mesoscutum (0.5–1.0OD), scutellum (1–1.5OD) also on posterior margin, longest posteromedially, metanotum (≤2OD); propodeal hairs moderately long and dense dorsolaterally (0.5–1.5OD); scopae on metafemur and metatibia (1–1.5OD); T1-T3 apicolateral patches of tomentum (0.5–1OD); S1 hairs long and moderately dense (≤2OD); S2 scopal hairs (1–2.5OD).

*Surface sculpture*: As in male except as follows: clypeus moderately densely punctate throughout (i=1-2d); supraclypeal area irregularly punctate (i=1-2d) densely punctate toward lateral margin (i≤d); frontal area densely punctate (i≤d); ocellocular space moderately densely punctate adjacent to compound eye (i=1-2d) becoming impunctate adjacent to lateral ocellus; vertexal and genal areas moderately densely punctate (i=1-2d); hypostomal area weakly imbricate to glossy apically and moderately densely punctate (i=1-3d); mesoscutum and scutellum densely punctate (i=0.5-1d); metepisternum irregularly punctate, sparse below scrobe (i=1-3d), sparser anterior of episternal groove (i≥3d), impunctate just dorsad of scrobe, otherwise irregularly spaced on hypoepimeral area (i≤2d); metanotum moderately densely punctate (i=0.5-2d); metapostnotum rugose to longitudinally striate posteriorly; propodeum coarsely imbricate, moderately densely punctate (i=1-2d); T1-T5 moderately puncticulate; T6 moderately densely punctate (i=1-2d).

*Structure*: Labrum ~2.5× wider than long (23.5:9.5); malar space ~1.25× as long as clypeal lateral (9.5:7.5); LOT just above anterior tentorial pits; clypeus ~1.1× as long as maximum width in frontal view (32:28) extending for more than half of its length beyond LOT; median longitudinal groove weak on clypeus; subantennal sutures ~1.4 longer than shortest distance between them (15:11); IAD ~1.4× AOD (11.5:8); scape ~3.3× as long as maximum width (23:7); pedicel longer than wide (8:7); F1 as long as wide (6.5:6.5); F2 ~0.7× as long as F1 (4.5) and 0.75× as long as F3 (6); UOD ~1.3× LOD, IOD ~1.1× as long as UOD (UOD:IOD:LOD - 45:48.5:34.5); frontal line carinate in lower third, flat just below median ocellus for a length of ~1OD, otherwise roughly defined; MOC ~1.2× as long as width of head (86:73); OOC ~0.6× IOC (11:19); genal area ~0.75× as wide as eye in lateral view (17:23); ratio of lengths of mesoscutum: scutellum: metanotum: metapostnotum – 57:20:11:14; probasitarsus ~3.4× as long as maximum depth (24:7); length of prodistitarsus ~0.8× that of preceding three tarsomeres combined (from II to V - 6:5:4.5:13); metafemur 3× as long as maximum depth (45:15); metatibia ~4.5× as long as maximum depth (58:13); metabasitarsus ~4.2× as long as maximum depth (38:9).

###### Type material

(9 males & 26 females): **Holotype** (male): Region III, C-453 km 22, S 28.1816° W 69.8261°, 1625m, 6.xi.2013, fl. yellow pan trap, S. Monckton, CCDB-19989 B08 // PCYU 0021682 (PCYU); **Allotype** (female): Region III, C-35 km 51.5, S 27.8237°, W 70.1227°, 875m, 29.ix.2013, S. Monckton, CCDB-19989 A12 // PCYU 0021674 (PCYU); **Paratypes: Region III**: one male, Las Juntas, *S 28.003°, W 69.976°, 1160m*, 5.x.1982, C. Tobar (PUCV); one female, same locality and date, R. Aldunate (PUCV); one male and three females, Rd to Pastos Largos, km 4, S 28.1643°, W 69.7913°, 2127m, 11.xi.2013, S. Monckton & J. Postlethwaite (PCYU); one male and one female, same data (AMNH); one female, same data (BBSL); one female, same data (CTMI); one female, Rd to Pastos Largos, km 0.5, S 28.1802°, W 69.8047°, 1863m, 6.xi.2013, S. Monckton (PCYU); one female, same locality, 6.xi-11.xii.2013, J. Postlethwaite & S. Monckton, pan trap (PCYU); four females, Fundo La Semilla, 125 km SE Copiapó, S 28.2507°, W 69.741°, 2358m, 17.x.2004, F.D. Parker, M.E. Irwin, pan traps, CHL-14509-54 // FDP52511, CHL-14509-55 // FDP52599, CHL-14509-56 // FDP52403, CHL-14509-03 // FDP4749 // USDA Native Bee Survey BBSL699121 (BBSL); two females, E of El Maray, S27.63363°, W70.007844°, 1316m, 3.xi.2015–4.ii.2016, L. Packer, pan traps (PCYU); one male and four females, Cuesta Castaño, S27.69314°, W69.75387°, 2975m, 3.xi.2015–4.ii.2016, L. Packer, pan traps (PCYU); one male and three females, Cuesta Castaño, S27.68156°, W69.75493°, 2672m, 3.xi.2015-4.ii.2016, L. Packer, pan traps (PCYU); one female, Finca las Herrera, S27.66756°, W69.79012°, 2315m, 3.xi.2015-4.ii.2016, L. Packer, pan trap (PCYU); one male and one female, E of Potrerillos, C-177, km 24, S 26.4585°, W 69.3272°, 2839m, 28.ix-16.xi.2013, J. Postlethwaite, pan traps (PCYU); one male, same locality, 16.xi.2013, J. Postlethwaite, blue pan, CCDB-22789 F02 (PCYU); one male, Hwy C-269, km 0.5, E of Potrerillos, S 26.4763°, W 69.3214°, 3000m, 28.iii–24.iv.2013, J. Postlethwaite & S. Monckton, yellow cup (PCYU); **Region IV**: one female, Limarí Prov., Chañar, S 30.2804°, W 70.6402°, 1564m, 4.ix.2004, L. Packer (PCYU).

###### Variation.

Some females have a yellow-brown or yellow spot on the lower paraocular area below the anterior tentorial pit.

###### Distribution.

Upper Intermediate Desert and northern Central Andean Cordillera, from Fundo La Semilla west to Last Juntas and north to E of Potrerillos (Region III); 875–3000m a.s.l.

###### Ecology.

Recorded September to December and March to April.

###### Etymology.

This species is named after the guanaco - *Lama guanicoe* (Müller, 1776) - a camellid native to South America and found throughout Chile. The specific epithet is identical to that of the guanaco and is treated as a noun in apposition.

##### Chilicola (Heteroediscelis) katherinae

Taxon classificationAnimaliaHymenopteraColletidae

Monckton sp. n.

http://zoobank.org/7AD41B89-6EE8-4536-99F3-2D6948D71390

**Figure 39.**
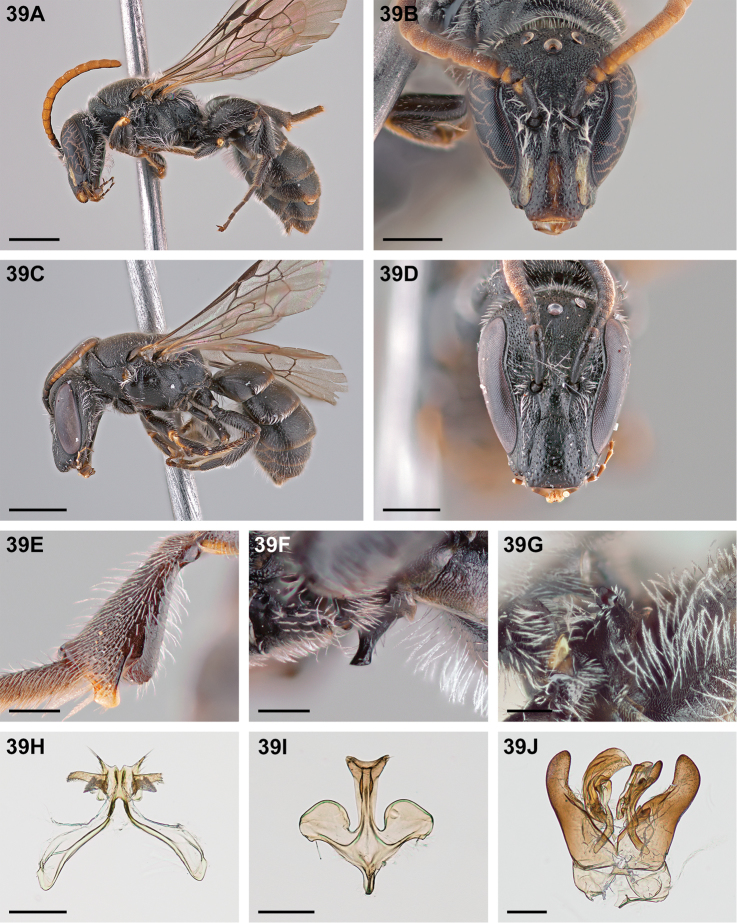
*Chilicola katherinae*: **A** male habitus, lateral view, scale bar 1 mm **B** male head, frontal view, scale bar 0.5 mm **C** female habitus, lateral view, scale bar 1 mm **D** female head, frontal view, scale bar 0.5 mm **E** male metatibia, posterior view, scale bar 0.25 mm **F** male S1 process, lateral view, scale bar 0.25 mm **G** male S1 process, apical view, scale bar 0.25 mm **H** male S7, scale bar 0.25 mm **I** male S8, scale bar 0.25 mm **J** male genital capsule, scale bar 0.25 mm.

Figs 11A ; 13D–F ; 18D ; 20A ; 39A–J ; 49

###### Diagnosis.

Males are diagnosable by the combination of malar space subequal to or longer than clypeal lateral (1–1.1×), the S1 proces apical surface weakly longitudinally concave and in lateral view anterior and posterior margins divergent apically, and the metatibia >2× as long as maximum depth with summit of ventral convexity situated at approximately three-quarters tibial length. Females are diagnosable by the combination of protibia yellow on basal half of dorsal surface only and malar space subequal in length to the clypeal lateral (0.95–1.05×). Females may be confused with *Chilicola randolphi* or *Chilicola vina*, but can be distinguished from the former by the dark brown metasoma and from the latter by the shorter malar space (≥1.2× as long as clypeal lateral in *Chilicola vina*).

###### Description.

**Male (Holotype).** Length 6.4mm, forewing length 3.9mm, head width 1.3mm, thorax width 1.4mm, median ocellar diameter (OD) 0.15mm.

*Colouration*: Black-brown, following parts yellow: labrum except apical margin yellow-brown; lower paraocular area to below transverse portion of epistomal suture medially, extending ~0.5OD further laterally; apicoventral spot on scape; apicoventral surface of pedicel; protrochanter apical rim posteriorly; narrow apical ring on profemur; dorsal surface of protibia; apical half of prodistitarsus; apicodorsal rim of mesofemur; basidorsal spot on mesotibia; apical lamina of metatibia; anterior spot on tegula. Following parts yellow-brown: median line and medial third of apical margin on clypeus, apex dark laterad; ventral surface of antennal flagellomeres F1-F10, narrowing basally on F1; mesobasitarsus ventrally; narrow apical ring on metafemur, expect brown dorsally. Following parts brown: ventral surface of antennal flagellomere F11; small apicodorsal spot on mesotibia; apical one-third of mesodistitarsus. Metasoma black, T1-T7 marginal zones translucent yellow.

*Pubescence*: White hairs generally short (0.5OD) and sparse, not especially plumose; on lower paraocular area and around antennal base moderately long and moderately dense (1OD); genal beard long and moderately dense (0.5–2OD) longest at midlength; discs of mesoscutum, scutellum, and metanotum mostly bare, few short hairs (0.5OD), longer and denser toward lateral margin as follows: mesoscutum (0.5–1OD), scutellum (1–1.5OD) also on posterior margin, longest posteromedially, metanotum (≤2OD); propodeal hairs moderately long and dense dorsolaterally (0.5–1.5OD); T1-T2 apicolateral patches of tomentum (0.5–1OD), T3 apicolateral hair band sparse, not tomentose; S2 hairs long and dense (2OD basolaterad, 1.5OD distilaterad, shorter mesad).

*Surface sculpture*: Microsculpture imbricate, integument generally dull; punctures generally small and deep. Clypeus moderately densely punctate (i=1-2d); supraclypeal area moderately densely and irregularly punctate (i=1-3d) densely punctate toward lateral margin (i≤d); lower paraocular area densely punctate (i=d); frontal area very densely punctate (i≤0.5d), densely punctate around ocelli (i≤d); ocellocular space moderately densely punctate (i=1-2d); vertexal area rugose and densely punctate (i≤d); scape shallowly and moderately densely punctate (i=1-2d) densely punctate apicoventrally (i≤d); genal area weakly imbricate and moderately sparsely punctate (i=1-2d); hypostomal area weakly imbricate to glossy apically, shallowly and moderately densely punctate (i≥2d); pronotum densely punctate (i=0.5-1d), lateral surface coarsely imbricate; mesoscutum densely punctate (i≤d); scutellum moderately densely punctate (i=0.5-2d) punctures crowded around median (i≥2d); mesepisternum irregularly punctate, sparse below scrobe (i=1-3d), sparser anterior of episternal groove (i≥2d), impunctate just dorsad of scrobe, otherwise moderately dense on hypoepimeral area (i=1-2d); metanotum moderately densely punctate (i≤2d) punctures crowded around median and anteriorly (i≤d); metepisternum densely punctate (i≤d) distinctly longitudinally striate anterodorsally; metapostnotum rugose; propodeum coarsely imbricate, rugulose posteriorly, moderately densely punctate throughout (i=1-2d); T1 moderately densely punctate; T2-T6 coarsely imbricate; T2-T5 moderately densely punctate, basal depressed area more coarsely microsculptured; T6-T7 moderately sparsely punctate (i=1-3d); marginal zones of terga weakly imbricate and shiny, minutely punctate.

*Structure*: Labrum 2.5× wider than long (25:10); malar space ~1.1× as long as clypeal lateral (9:8); LOT at level of anterior tentorial pits; clypeus ~1.2× as long as maximum width in frontal view (37:32) extending for approximately half its length beyond LOT; median longitudinal groove present on clypeus; subantennal sutures ~1.2× as long as the shortest distance between them (16:13); IAD ~1.5× AOD (12.5:8.5); scape 2.5× as long as maximum width (25:10); pedicel as long as wide (9:9); F1 slightly shorter than wide (8.5:9); F2 ~1.5× as long as F1 and shorter than F3 (F2:F3 – 12.5:15); UOD ~1.4× LOD, IOD ~1.1× UOD (UOD:IOD:LOD - 53:58:39); frontal line carinate in lower two-fifths, flat just below median ocellus for a length of ~0.5OD, otherwise roughly defined; MOC subequal to width of head (93:94); OOC ~0.67× IOC (13:19); genal area ~0.6× as wide as compound eye in lateral view (18:29); ratio of lengths of mesoscutum: scutellum: metanotum: metapostnotum - 66:23:13:17; probasitarsus ~4.2× as long as maximum depth (27:6.5); length of prodistitarsus subequal to that of preceding three tarsomeres combined (from II to V - 9:7:6:21); metafemur ~2× as long as maximum depth (71:36); summit of metatibial ventral convexity at approximately two-thirds tibial length; metatibia ~2.1× as long as maximum depth (60:28); in apical view apical lamina of metatibia short and thick, length ~0.6× OD (6.5:11); ventral metatibial carina angled ventrad, rounded inflection near midpoint, posterior carina absent basally and originating at midpoint of ventral carina; metabasitarsus ~5.1× as long as maximum depth (46:9); S1 process apical surface longitudinally concave, in lateral view anterior and posterior margins divergent apically, apex appearing anteriorly bent. S7 apodemal arm sclerotized margin recurved mesad, dividing membranous portion of arm; ventral lobe ~0.25× as broad as basal attached length; dorsal lobe strap-like, subparallel-sided and somewhat anteriorly curved, with basal tuft of long setae and a short apical row, midlength bare. S8 lateral process ~1.2× wider than long, anteromedial sclerotized margin irregular with sclerotized spot disjunct from margin (but see variation below). Gonobase apicoventral truncate process biconvex and distinctly notched by ~1.5× width of one convexity.

**Female (Allotype).** Length 5.5mm, forewing length 3.4mm, head width 1.2mm, thorax width 1.4mm, median ocellar diameter (OD) 0.12mm.

*Colouration*: Black to black-brown except as follows: mandible with brown basal spot, apical half yellow-orange except apex black. Following parts brown: pedicel, F1 and F2, on apicoventral margins; apicodorsal rim of metafemur; protrochanter apical rim posteriorly; apical one-third of mesodistitarsus; narrow apical ring on metafemur; large basidorsal spot on metatibia. Following parts yellow: dorsal surface of protibia in basal half; apical half of prodistitarsus; basidorsal spot on mesotibia; faint anterior spot on tegula. Ventral surface of F3-F10 yellow-brown, narrowing basally on F3. Metasoma black, T1-T5 marginal zone translucent yellow.

*Pubescence*: As in male except as follows: genal beard sparse (0.5–2OD); mesoscutal hairs short and sparse (≤0.5OD) denser toward lateral margin; discs of scutellum and metanotum mostly bare, few short hairs (0.5OD), longer and denser toward lateral and posterior margins of scutellum (0.5–1.5OD) longest posteromedially, and toward lateral margin of metanotum (≤2OD); propodeal hairs moderately long and dense dorsolaterally (0.5–1.5OD); scopae on metafemur and metatibia (1–1.5OD); T1-T2 apicolateral patches of tomentum (0.5–1OD), T3 apicolateral hair band sparse, not tomentose; S1 hairs long and moderately dense (≤2.5OD); S2 scopal hairs (1–2.5OD).

*Surface sculpture*: As in male except as follows: clypeus moderately densely punctate (i=1-2d) except sparsely punctate medially (i=2-3d); supraclypeal area moderately densely punctate (i=1-2d) more densely punctate laterally (i≤d); frontal area moderately densely punctate (i≤2d), moderately densely punctate around ocelli (i=1-2d); ocellocular space moderately densely punctate adjacent to compound eye (i=1-2d) becoming impunctate adjacent to lateral ocellus; vertexal area densely punctate (i≤d); scape shallowly and moderately densely punctate (i=1-2d); genal area imbricate and moderately densely punctate; hypostomal area imbricate and moderately sparsely punctate (i≥2d); mesoscutum and scutellum densely punctate (i≤d), except mesoscutum moderately densely punctate laterally (i=1-2d) and scutellar punctures crowded on median (i=0.5d); mesepisternum irregularly punctate, sparse below scrobe (i=1-3d), sparser anterior of episternal groove (i≥3d), impunctate just dorsad of scrobe, otherwise moderately sparse on hypoepimeral area (i=0.5-3d); metanotum moderately densely punctate (i=0.5-2d); metepisternum densely punctate (i≤d) weakly striate anterodorsally; metapostnotum rugose, fewer transverse lineations posteriorly; propodeum coarsely imbricate and moderately densely punctate (i=1-2d); T1-T5 punctures small; T1 & T6 moderately sparsely punctate (i=1-3d); T2-T3 moderately densely punctate (i≤2d); T4-T5 sparsely punctate (i≥3d).

*Structure*: Labrum ~2.6× wider than long (28:11); malar space slightly longer than clypeal lateral (9.5:9); LOT at level of anterior tentorial pits; clypeus 1.25× as long as maximum width in frontal view (40:32) extending for approximately half its length beyond LOT; clypeus without median longitudinal groove; subantennal sutures ~1.1× as long as the shortest distance between them (16:15); IAD ~1.33× AOD (13.5:10); scape ~3.2× as long as maximum width (27:8.5); pedicel longer than wide (9:8); F1 as long as wide (7.5:7.5); F2 shorter than F1 and shorter than F3 (F2:F3 - 5:6); UOD ~1.3× LOD, IOD ~1.05× UOD (UOD:IOD:LOD - 54:56:41); frontal line carinate between antennal bases for a length of ~1.5OD, flat just below median ocellus for a length of ~1OD, otherwise roughly defined; MOC ~1.1× as long as width of head (98:88); OOC ~0.6× IOC (13:21); genal area ~0.55× as wide as eye in lateral view (16:29); ratio of lengths of mesoscutum: scutellum: metanotum: metapostnotum – 66:24:13:16; probasitarsus ~3.1× as long as maximum depth (25:8); length of prodistitarsus subequal to that of preceding three tarsomeres combined (from II to V - 6:6:5:16); metafemur ~3× as long as maximum depth (53:18); metatibia ~4.2× as long as maximum depth (67:16); metabasitarsus 4.1× as long as maximum depth (41:10).

###### Type material

(5 males & 17 females): **Holotype** (male): Region VII, Río Teno nr. Estero Pichuante, S 35.0863° W 70.4971°, 1457m, 19.ii.2015, S. Monckton, CCDB-22014 E01, parasitized (but see comments) (PCYU); **Allotype** (female): same data as holotype (PCYU); **Paratypes: Region VII**: one female, same locality as holotype, 28.ii-21.iii.2013, S. Monckton & J. Postlethwaite, pan trap, parasitized (PCYU); one female, same locality as holotype, 2.iii.2015, S.Monckton (PCYU); one female, same locality as holotype, 2.iii.2015, S. Monckton (CTMI); one female, Río Teno, S35.04417°, W70.59093°, 1241m, 22.ii.2016, L. Packer (PCYU); two females, E of Los Queñes, *S 35.028°, W 70.713°, 817m*, 1.i.2013, L. Packer & R. Smith, CCDB-19991 C07, CCDB-19991 C08 (PCYU); four males, Los Queñes, *S 35.001°, W 70.816°, 707m*, 31.x.1983, J. Magunacelaya (PUCV); one female, same locality and date, C. Tobar (PUCV); four females, same locality and date, F. Rodriguez (BBSL); **Region VIII**: two females, Ñuble: Shangrila (near Las Trancas), *S 36.876°, W 71.468°, 1549m*, 13–15.xii.1983, Luis E. Peña (AMNH); one female, Ñuble: Shangri-la, Las Trancas, SE of Recinto, *S 36.876°, W 71.468°, 1549m*, 13–17.xii., Luis E. Peña (AMNH); one female, Las Trancas, *S 36.911°, W 71.48°, 1250m*, 10.xi.1976, H. Toro (AMNH); one female, Las Cabras, mts. in Chillán area, S Chillán Volcano, Ñuble Prov., *S 36.918°, W 71.429°, 1500m*, 31.vi.1963, Luis E. Peña (AMNH).

###### Variation.

Paratype males differ in colour from the holotype as follows: inverted T shape on clypeus yellow; lower paraocular maculation extending above transverse portion of epistomal suture; wide apical ring on profemur yellow; apical half of prodistitarsus yellow-brown; narrow basal ring and large apicodorsal spot on mesotibia yellow; wide basal and apical rings on metatibia yellow, expect dark apicodorsally; basal half of metabasitarsus yellow ventrally. Additionally, some females have a yellow or yellow-brown basal spot on the mandible, the apicoventral margin of F1 and F2 brown or yellow-brown, and/or a yellow-brown or brown spot on the lower paraocular area below the anterior tentorial pit. The male S8 anteromedial sclerotized margin varies in form among paratypes: C.kate.003 has a sclerotized spot disjunct from the margin as in the holotype (easily distinguished only on the righthand lateral process), C.kate.004 has a distinct convex incursion which appears swollen, and C.kate.005 has the margin only slightly widened and weakly convex; all three were collected from Los Queñes on the same date.

###### Distribution.

Southern Andean Cordillera, from Río Teno near Estero Pichuante west to Los Queñes (Region VII) and south to Valle Las Trancas (Region VIII); 707–1549m a.s.l.

###### Ecology.

Recorded October to March and June.

###### Etymology.

The specific epithet honours Katie D’Angelo, whose support, love, and encouragement were vital to the completion of this work. It is formed in the genitive singular case.

###### Comments.

The holotype is parasitized, with a visible female strepsipteran protruding from beneath T4. In order to verify that its morphology was not altered by parasitism, the holotype was thoroughly compared to the male paratypes for differences in colouration, pubescence, surface sculpture and structure; only colouration differed appreciably from the paratypes and these differences are noted in the variation section above. One female paratype (C.kate.007) from the same locality as the holotype is also parasitized, with two female strepsipterans protruding from beneath T4.

##### Chilicola (Heteroediscelis) lickana

Taxon classificationAnimaliaHymenopteraColletidae

Monckton sp. n.

http://zoobank.org/EDE5C3E0-7E79-404A-BD42-CB7F741963CD

**Figure 40.**
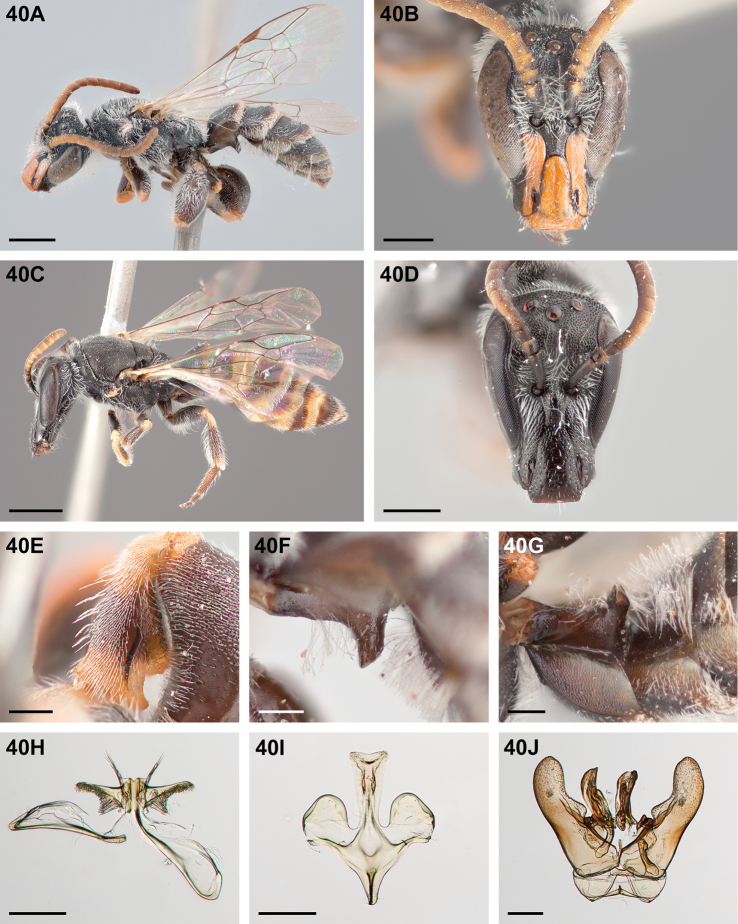
*Chilicola lickana*: **A** male habitus, lateral view, scale bar 1 mm^†^ **B** male head, frontal view, scale bar 0.5 mm **C** female habitus, lateral view, scale bar 1 mm **D** female head, frontal view, scale bar 0.5 mm **E** male metatibia, posterior view, scale bar 0.25 mm **F** male S1 process, lateral view^†^, scale bar 0.25 mm **G** male S1 process, apical view, scale bar 0.25 mm **H** male S7 (C.lkna.005), scale bar 0.25 mm **I** male S8 (C.lkna.005), scale bar 0.25 mm **J** male genital capsule (C.lkna.005), scale bar 0.25 mm.

Figs 6B ; 7A ; 22A–B ; 40A–J ; 49

###### Diagnosis.

Males are diagnosable by the combination of malar space ~2.2× as long as clypeal lateral, and apical surface of S1 process with a longitudinal median ridge. Females are diagnosable by the combination of malar space ~2× as long as clypeal lateral, and the presence of wide yellow basal and apical rings on the metatibia.

###### Description.

**Male (Holotype).** Length 6.8mm, forewing length 4.4mm, head width 1.4mm, thorax width 1.4mm, median ocellar diameter (OD) 0.15mm.

*Colouration*: Black-brown, following parts yellow: labrum; clypeus except broadly dark brown along epistomal suture and apex yellow along entire width; lower paraocular area extending more than 1OD above transverse epistomal suture, not reaching lower tangent of antennal socket; apex of profemur broadly on anterior surface, narrowly on posterior surface; dorsal surface of protibia, including dorsal half of anterior surface; apicodorsal rim of mesofemur, wider anteriorly; apicodorsal rim of metatibia; wide basal and apical rings on mesotibia, as well as apical quarter of anterior surface; basal half of mesobasitarsus dorsally; apicodorsal rim of metafemur; anterior surface of metatibia in basal quarter and apical band narrow dorsally to wide on summit of ventral convexity, posterior surface in basal one-third and apical one-third; ventral metatibial carina; basal half of metabasitarsus ventrally; apex of T7. Following parts yellow-brown: apicoventral surface of scape and pedicel, ventral surface of antenna from F1 to terminal flagellomere, narrowing basally on F1, and suffused with darker brown on remaining flagellomeres; apical half of prodistitarsus. Protrochanter apical rim posteriorly yellow-brown. Faint yellow anterior spot on tegula. Apicoventral rim of metacoxa and metatrochanter translucent brown. Posterior metatibial carina black. Metasoma black, T1-T6 marginal zones amber to translucent yellow at margins.

*Pubescence*: White, hairs generally short (0.5OD) and sparse, not especially plumose; on lower paraocular area and around antennal base long and moderately dense (1.5–2OD); genal beard long and dense (0.5–3OD) longest at midlength; mesoscutal hairs moderately long and sparse (1OD), longer and denser toward lateral margin (1–1.5OD); discs of scutellum and metanotum mostly bare, few short hairs (0.5OD), longer and denser toward lateral and posterior margins of scutellum (1–2OD) longest posteromedially, and toward lateral margin of mmetanotum (≤2OD); propodeal hairs moderately long and dense dorsolaterally (0.5–2OD); T1-T3 apicolateral patches of tomentum (0.5–1OD); S2 hairs long and dense (2OD basolaterad, 0.5OD distilaterad, shorter mesad).

*Surface sculpture*: Microsculpture imbricate, integument generally dull; punctures generally small and deep. Clypeus moderately densely punctate (i=1-2d) irregularly and sparsely punctate medially (i≥2d); supraclypeal area irregularly punctate (i=1-4d) densely punctate toward lateral margin (i≤d); lower paraocular area densely punctate (i=d); frontal area very densely punctate (i≤0.5d), densely punctate around ocelli (i≤d); vertexal area moderately densely punctate (i=1-2d); scape shallowly and moderately densely punctate (i=1-2d) densely punctate apicoventrally (i≤d); genal area microstriate and densely punctate (i≤d); hypostomal area densely punctate (i=d); pronotum densely punctate (i=0.5-1d), lateral surface coarsely imbricate; mesoscutum densely punctate (i=0.5-1d) moderately densely punctate laterally (i=1-2d) and crowded around median (i≤d); scutellum moderately densely punctate (i=0.5-2d) punctures crowded around median (i≤d); mesepisternum irregularly punctate, moderately dense below scrobe and anterior of episternal groove (i=1-2d), impunctate just dorsad of scrobe, otherwise irregularly spaced on hypoepimeral area (i≤3d); metanotum moderately densely punctate (i=1-2d) puntures crowded around median and anteriorly (i≤0.5d); metepisternum densely punctate (i≤d) distinctly longitudinally striate anterodorsally; metapostnotum rugose; propodeum rugulose posteriorly, moderately densely punctate throughout (i=1-2d); metasomal terga coarsely imbricate, T1-T3 very densely punctate (i≤d), T4-T5 moderately densely punctate (i≤2d), T6 moderately densely punctate (i=1-2d), T7 sparsely punctate (i≥2d); marginal zones of terga weakly imbricate and shiny, minutely punctate.

*Structure*: Labrum ~2.6× wider than long (30:11.5); malar space ~2.2× as long as clypeal lateral (20:9); LOT above anterior tentorial pits; clypeus ~1.3× as long as maximum width in frontal view (46:35) extending for approximately two thirds of its length beyond LOT; median longitudinal groove present on clypeus; subantennal sutures ~1.8× as long as the shortest distance between them (22:12); IAD ~1.7× AOD (13.5:8); scape ~2.4× as long as maximum width (29:12); pedicel slightly shorter than wide (9:11); F1 shorter than wide (10:11); F2 1.4× as long as F1 and shorter than F3 (F2:F3 - 14:16); UOD ~1.3× LOD, IOD ~1.1× UOD (UOD:IOD:LOD - 55:61:42); frontal line carinate between antennal bases for a length less than 1OD, flat just below median ocellus for less than 0.5OD, otherwise undefined; MOC ~1.1× width of head (111:100); OOC ~0.67× IOC (14:21); genal area ~0.6× as wide as compound eye in lateral view (22:36); ratio of lengths of mesoscutum: scutellum: metanotum: metapostnotum - 73:27:13:20; probasitarsus ~3.8× as long as maximum depth (34:9); length of prodistitarsus subequal to that of preceding three tarsomeres combined (from II to V - 8:6:4:17); metafemur ~1.8× as long as maximum depth (80:45); summit of metatibial ventral convexity at approximately two-thirds tibial length; metatibia 2× as long as maximum depth (66:33); in apical view apical lamina of metatibia short and thick, length equal to OD (11:11); ventral metatibial carina bowed ventrad and absent basally, originating at midpoint of posterior carina, the latter sharply inflected near base; metabasitarsus ~4.2× as long as maximum depth (50:12); S1 process apical surface with longitudinal median ridge, in lateral view anterior and posterior margins subparallel apically and median ridge projecting posteriorly from apex. S7 apodemal arm sclerotized margin terminating laterally at arm midlength; ventral lobe narrow, almost linear; dorsal lobe roughly triangular, wide at base and narrow apically, with row of setae long basally and shorter apically; apex of disc disinctly emarginate. S8 lateral process ~0.9× as wide as long, anteromedial sclerotized margin swollen forming convex incursion into membranous interior. Gonobase apicoventral process weakly emarginate.

**Female (Allotype).** Length 6.4mm, forewing length 3.5mm, head width 1.1mm, thorax width 1.4mm, median ocellar diameter (OD) 0.12mm.

*Colouration*: Black to black-brown except as follows: labrum translucent brown with apex yellow; mandible with yellow basal spot, translucent yellow with apex translucent brown; pale spot on lower paraocular area below anterior tentorial pit. Following parts yellow: dorsal surface and narrow apical ring on protibia; probasitarsus; apical two-thirds of prodistitarsus; wide basal and apical rings on mesotibia; apical half of mesodistitarsus; apical ring on metafemur, wider ventrally; wide basal and apical ring on metatibia; anterior spot on tegula. Following parts yellow-brown: small spot on apicoventral rim of scape; apicoventral surface of pedicel, F1, and F2; ventral surface of antenna from F3 to terminal flagellomere, with pale apical bands; ventral half of metabasitarsus. Metasoma black-brown, T1-T5 marginal zones narrowly yellow to translucent yellow at margins.

*Pubescence*: As in male except as follows: clypeal hairs short and sparse (0.5OD); lower paraocular hairs moderately long and moderately dense (1OD); genal beard sparse (0.5–2OD); mesoscutal hairs short and sparse (≤0.5OD) denser toward lateral margin (0.5OD); disc of scutellum mostly bare, few short hairs (0.5OD), longer and denser toward lateral and posterior margins (1–1.5OD) longest posteromedially; propodeal hairs moderately long and dense dorsolaterally (0.5–1.5OD); scopae on metafemur and metatibia (1–1.5OD); T1-T2 apicolateral patches of tomentum (0.5–1OD), T3 apicolateral hair band sparse, not tomentose; S1 hairs long and moderately dense (≤2OD); S2 scopal hairs (1–2OD).

*Surface sculpture*: As in male except as follows: frontal area densely punctate (i≤d); ocellocular space moderately densely punctate (i=1-2d); vertexal area moderately densely punctate (i=1-2d); scape shallowly and moderately densely punctate (i=1-2d); genal area coarsely imbricate to weakly imbricate anteriorly and densely punctate (i=d); hypostomal area imbricate to glossy apically, shallowly and irregularly punctate (i=1-3d); pronotum densely punctate (i=d); mesoscutum and scutellum densely punctate (i=0.5-1d) punctures crowded around median (i≤0.5d); mesepisternum irregularly punctate, moderately dense below scrobe (i=1-2d), sparser anterior of episternal groove (i=1-3d), impunctate just dorsad of scrobe, otherwise moderately dense on hypoepimeral area (i≤2d); metanotum moderately densely punctate (i=0.5-2d); metapostnotum rugose, fewer transverse lineations and more weakly microsculptured posteriorly; propodeum coarsely imbricate and moderately densely punctate (i=1-2d); T1-T3 & T6 moderately densely punctate (i=1-3d on T1-T2; i=1-2d on T3 & T6); T4-T5 punctures small and sparse (i≥2d).

*Structure*: Labrum ~2.25× wider than long (26:11.5); malar space twice as long as clypeal lateral (18:9); LOT above anterior tentorial pits; clypeus ~1.4× as long as maximum width in frontal view (45:32) extending for approximately two thirds of its length beyond LOT; median longitudinal groove weak on clypeus; subantennal sutures ~1.75 longer than shortest distance between them (19:11); IAD ~1.33× AOD (12:9); scape 3.6× as long as maximum width (27:7.5); pedicel slightly longer than wide (8:7.5); F1 longer than wide (8:7); F2 more than half as long as F1 (5) and ~0.7× as long as F3 (7); UOD ~1.3× LOD, IOD ~1.1× UOD (UOD:IOD:LOD - 48.5:53:38); frontal line carinate in lower third, flat above; MOC ~1.25× as long as width of head (103:81); OOC ~0.5× IOC (11:20.5); genal area ~0.67× as wide as eye in lateral view (17:26); ratio of lengths of mesoscutum: scutellum: metanotum: metapostnotum – 63:24:11:15; probasitarsus 4× as long as maximum depth (28:7); length of prodistitarsus equal to that of preceding three tarsomeres combined (from II to V - 5.5:5:4.5:15); metafemur ~2.8× as long as maximum depth (53:19); metatibia ~4.9× as long as maximum depth (68:14); metabasitarsus ~4.3× as long as maximum depth (41:9.5).

###### Type material

(3 males & 3 females). **Holotype** (male): Region II, 5.1km NNE. of San Pedro, *S 22.872°, W 68.177°, 2531m*, 14.x.2001, L. Packer (PCYU); **Allotype** (female): Region II, B-245 km 12, N of SP de Atacama, S 22.8272°, W 68.1289°, 2801m, 6.xii.2013, J. Postlethwaite, CCDB-19989 D06 (PCYU); **Paratypes: Region II**: one male and one female, Aguas Blancas SE of San Pedro de Atacama, *S 23.268°, W 67.995°, 2474m*, 12.x.2001, Packer & Fraser, CCDB-22789 D12, CCDB-03755 E11 (PCYU); one sample, mixed male and female cleared parts, same data (PCYU).

###### Distribution.

Northern Volcanic Cordillera, from Aguas Blancas north to 12 km N of San Pedro de Atacama (Region II); 2474–2801m a.s.l.

###### Ecology.

Recorded October and December.

###### Etymology.

The specific epithet is taken from Kunza, an extinct language of the Atacama people. *Lickana* is the word for the Atacama region, encompassing the type locality and nearby San Pedro de Atacama (Vaïsse and Hoyos 1896). It is to be treated as a noun in apposition.

##### Chilicola (Heteroediscelis) mavida

Taxon classificationAnimaliaHymenopteraColletidae

Toro & Moldenke, 1979

Figs 5A ; 6C ; 9A–B ; 21B ; 23A ; 24C–D ; 41A–J ; 49

Chilicola mavida Toro & Moldenke, 1979: 122–124 (male holotype, female allotype, AMNH [examined]). Michener 2002: 5–7 (phylogeny). Packer 2008: 77. Montalva and Ruz 2010: 28 (checklist). Moure et al. 2012 (catalogue). Monckton 2014: 234. Ascher and Pickering 2015 (checklist).

###### Diagnosis.

Males are diagnosable by the combination of first flagellomere longer than wide (≥1.1×), malar space longer than clypeal lateral (1.05–1.1×), and S1 process with anterior apical angle acute in lateral view. Females are diagnosable by the combination of malar space subequal in length to the clypeal lateral (0.9–1×) and leg colouration as follows: apicodorsal spot on mesotibia yellow-brown, apicodorsal rim of metafemur yellow, wide basal ring of metatibia yellow, posterior apical rim of metatibia yellow-brown. Females of *Chilicola curvapeligrosa* are most similar, but have darker leg colouration (following parts brown: apicodorsal spot on mesotibia, apicodorsal rim of metafemur, basidorsal spot on metatibia; apex of metatibia black-brown).

###### Description.

**Male.** Length 5.3–5.8mm, forewing length 3.4–3.5mm, head width 1.1–1.2mm, thorax width 1.1–1.2mm, median ocellar diameter (OD) 0.13mm.

*Colouration*: Black-brown, following parts yellow: labrum; clypeus except broadly dark along epistomal suture; lower paraocular area, extending ~1OD above transverse portion of epistomal suture and not reaching lower tangent of antennal socket; apicoventral spot on scape; apicoventral surface of pedicel; protrochanter apical rim posteriorly; apex of profemur broadly on anterior surface, narrowly on posterior surface; protibia except dark medioventrally; probasitarsus dorsally, at least in basal half; apex of mesofemur broadly on anterior surface, narrowly on posterior surface; broad basal and apical rings on mesotibia, the latter widened to apical half anteriorly; at least basoventral spot on mesobasitarsus; apicoventral rim of metacoxa and metatrochanter, the latter sometimes yellow-brown; narrow apical ring on metafemur; metatibia in basal one-third, in apical quarter of anterior surface, apical one-third of posterior surface and along ventral varina, yellow colouration continuous along ventral surface, sometimes continuous or nearly so along dorsal surface; metabasitarsus in basal one-third dorsally, basal two-thirds ventrally; anterior spot on tegula; apex of T7. Ventral surface of flagellum yellow-brown, flagellomeres suffused with brown basally. Apical half of prodistitarsus and apical one-third of mesodistitarsus brown. Posterior metatibial carina black. Metasoma black, T1-T6 marginal zones translucent yellow.

*Pubescence*: White, hairs generally short (0.5OD) and sparse, not especially plumose; on lower paraocular area and around antennal base moderately long and moderately dense (1OD); genal beard long and dense (0.5–3OD) longest at midlength; discs of mesoscutum, scutellum, and metanotum mostly bare, few short hairs (0.5OD), longer and denser toward lateral margin as follows: mesoscutum (0.5–1OD), scutellum (1–2OD) also on posterior margin, longest posteromedially, metanotum (≤2OD); propodeal hairs moderately long and dense dorsolaterally (0.5–1.5OD); T1-T3 apicolateral patches of tomentum (0.5–1OD); S2 hairs long and dense (1.5OD basolaterad, 0.5OD distilaterad, shorter mesad).

*Surface sculpture*: Microsculpture imbricate, integument generally dull; punctures generally small and deep. Clypeus and lower paraocular area moderately densely punctate (i=1-2d) except clypeus irregularly punctate in upper half (i=1-3d); supraclypeal area irregularly punctate (i=1-4d) densely punctate toward lateral margin (i≤d); frontal area very densely punctate (i≤0.5d), densely punctate around ocelli (i≤d); ocellocular space densely punctate adjacent to compound eye (i≤d) becoming impunctate adjacent to lateral ocellus; vertexal area rugose and very densely punctate (i≤0.5d); scape shallowly and moderately densely punctate (i=1-2d) densely punctate apicoventrally (i≤d); genal area microstriate and moderately densely punctate (i=1-2d); hypostomal area weakly imbricate to glossy apically and densely punctate (i=d); pronotum densely punctate (i=0.5-1d), lateral surface coarsely imbricate; mesoscutum densely punctate (i=0.5-1d) except moderately densely punctate laterally (i=1-2d); scutellum moderately densely punctate (i=1-2d) punctures crowded around median (i≤d); mesepisternum irregularly punctate, sparse below scrobe (i=2-3d), sparser anterior of episternal groove (i≥3d), impunctate just dorsad of scrobe, otherwise moderately dense on hypoepimeral area (i=1-2d); metanotum moderately densely punctate (i=1-2d) punctures crowded around median and anteriorly (i≤0.5d); metepisternum densely punctate (i≤d) distinctly longitudinally striate anterodorsally; metapostnotum rugose, fewer transverse lineations posteriorly; propodeum coarsely imbricate, rugulose posteriorly, moderately densely punctate throughout (i=1-2d); T1-T5 coarsely imbricate; T1 sparsely punctate basally (i≥2d) moderately densely punctate apically (i=1-2d); T2-T5 moderately densely punctate (i=1-2d); T6 moderately densely punctate (i=1-2d) and coarsely imbricate posteriorly; T7 sparsely puncticulate; marginal zones of terga weakly imbricate and shiny, punctate to impunctate at margins.

*Structure*: Labrum 2.3× wider than long (23:10); malar space subequal in length to clypeal lateral (9:8.5); LOT at level of anterior tentorial pits; clypeus subequal in length to maximum width in frontal view (31:30) extending for more than half of its length beyond LOT; median longitudinal groove present on clypeus, weak apically; subantennal sutures ~1.4× as long as the shortest distance between them (17:12); IAD ~2.2× AOD (13:6); scape 2.4× as long as maximum width (24:10); pedicel shorter than wide (8:9); F1 longer than wide (10:8.5); F2 1.3× as long as F1 and shorter than F3 (F2:F3 - 13:14); UOD ~1.4× LOD, IOD ~1.1× UOD (UOD:IOD:LOD - 50:54:35); frontal line carinate in lower two-fifths, flat just below median ocellus for a length of ~1OD, otherwise roughly defined; MOC subequal to width of head (89:90); OOC ~0.6× IOC (11:18); genal area ~0.5× as wide as compound eye in lateral view (17:33); ratio of lengths of mesoscutum: scutellum: metanotum: metapostnotum - 63:23:9:15; probasitarsus 3.25× as long as maximum depth (26:8); length of prodistitarsus subequal to that of preceding three tarsomeres combined (from II to V - 7:5:5:16); metafemur ~1.9× as long as maximum depth (70:37); summit of metatibial ventral convexity rounded and near tibial apex; metatibia ~1.8× as long as maximum depth (56:31); in apical view apical lamina of metatibia broad and thick, length 0.8× OD (8:10); ventral metatibial carina bowed ventrad and absent basally, originating at midpoint of weakly sigmoid posterior carina; metabasitarsus ~3.6× as long as maximum depth (43:12); S1 process apical surface with longitudinal median ridge, in lateral view anterior and posterior margins convergent apically and median ridge projecting posteriorly from apex. S7 apodemal arm sclerotized margin terminating laterally at arm midlength; ventral lobe narrow, almost linear; dorsal lobe roughly triangular, wide at base and narrow apically, with row of setae long basally and shorter apically; apex of disc disinctly emarginate. S8 lateral process ~0.9× as wide as long, anteromedial sclerotized margin following marginal contour. Gonobase apicoventral truncate process biconvex and distinctly notched by ~1.5× width of one convexity.

**Female.** Length 4.2–4.8mm, forewing length 2.6–3.0mm, head width 1.0mm, thorax width 1.0–1.1mm, median ocellar diameter (OD) 0.10mm.

*Colouration*: Black to black-brown except as follows: mandible with yellow basal spot, translucent yellow in apical half except apex translucent brown; antenna variable (scape entirely dark; pedicel to F2 dark, or apicoventral rim brown; ventral surface of F3 to penultimate flagellomere yellow-brown, narrowing basally on F3, flagellomeres suffused with brown basally; terminal flagellomere yellow-brown in basal half, brown in apical half). Following parts yellow: dorsal surface of protibia; apical two-thirds of prodistitarsus, sometimes yellow-brown; narrow basal ring on mesotibia; apicodorsal rim on metafemur; wide basal ring on metatibia, narrow ventrally; anterior spot on tegula. Following parts yellow-brown: apicodorsal spot on mesotibia; posterior apical rim on metatibia. Apical half of mesodistitarsus brown. Metasoma black, T1-T5 yellow-brown beyond premarginal line to translucent yellow at margins.

*Pubescence*: As in male except as follows: clypeal hairs short and sparse (0.5OD); genal beard sparse (0.5–2.5OD); discs of mesoscutum and scutellum with sparse short hairs (≤0.5OD), longer and denser toward lateral margin of mesoscutum (0.5–1OD), and toward lateral and posterior margins of scutellum (1–1.5OD) longest posteromedially; scopae on metafemur and metatibia (1–1.5OD); T1-T3 apicolateral patches of tomentum (0.5–1OD); S1 hairs long and moderately dense (≤2OD); S2 scopal hairs (1–2OD).

*Surface sculpture*: As in male except as follows: clypeus sparsely punctate (i=2-3d) moderately densely punctate toward lateral margin (i=1-2d); supraclypeal area irregularly punctate (i=1-2d) densely punctate toward lateral margin (i≤d); lower paraocular area densely punctate (i=d); frontal area densely punctate (i≤d), moderately densely punctate around ocelli (i=1-2d); vertexal area moderately densely punctate (i=1-2d); scape shallowly and moderately densely punctate (i=1-2d); genal area weakly imbricate and moderately densely punctate (i=1-2d); hypostomal area weakly imbricate to glossy apically and moderately densely punctate (i=1-2d); mesoscutum and scutellum densely punctate; metanotum moderately densely punctate (i=0.5-2d); metepisternum densely punctate (i≤d) distinctly longitudinally striate anterodorsally (weak in some specimens); metapostnotum rugose, fewer transverse lineations posteriorly (posterior half to two-thirds longitudinally carinate in some specimens); propodeum moderately densely punctate (i=1-2d); T1-T5 moderately puncticulate; T6 moderately densely punctate (i=1-2d).

*Structure*: Labrum 2.5× wider than long (20:8); malar space subequal in length to clypeal lateral (6:6.5); LOT at level of anterior tentorial pits; clypeus ~1.1× as long as maximum width in frontal view (29:26) extending for approximately half of its length beyond LOT; median longitudinal groove weak on clypeus; subantennal sutures subequal in length to the shortest distance between them (11:11); IAD ~1.4× AOD (11.5:8); scape ~3.4× as long as maximum width (22:6.5); pedicel longer than wide (8:6.5); F1 longer than wide (6.5:5.5); F2 shorter than F1 and shorter than F3 (F2:F3 - 4:5); UOD ~1.3× LOD, IOD ~1.1× as long as UOD (UOD:IOD:LOD - 44:47:34); frontal line carinate in lower third, flat above; MOC ~1.1× as long as width of head (77:71); OOC ~0.7× IOC (11:16); genal area ~0.65× as wide as eye in lateral view (15:23); ratio of lengths of mesoscutum: scutellum: metanotum: metapostnotum – 51:20:9:12.5; probasitarsus ~3.2× as long as maximum depth (21:6.5); length of prodistitarsus ~1.3× that of preceding three tarsomeres combined (from II to V - 4:4:3:14); metafemur ~2.9× as long as maximum depth (43:15); metatibia ~4.6× as long as maximum depth (53:11.5); metabasitarsus 4.25× as long as maximum depth (34:8).

**Figure 41.**
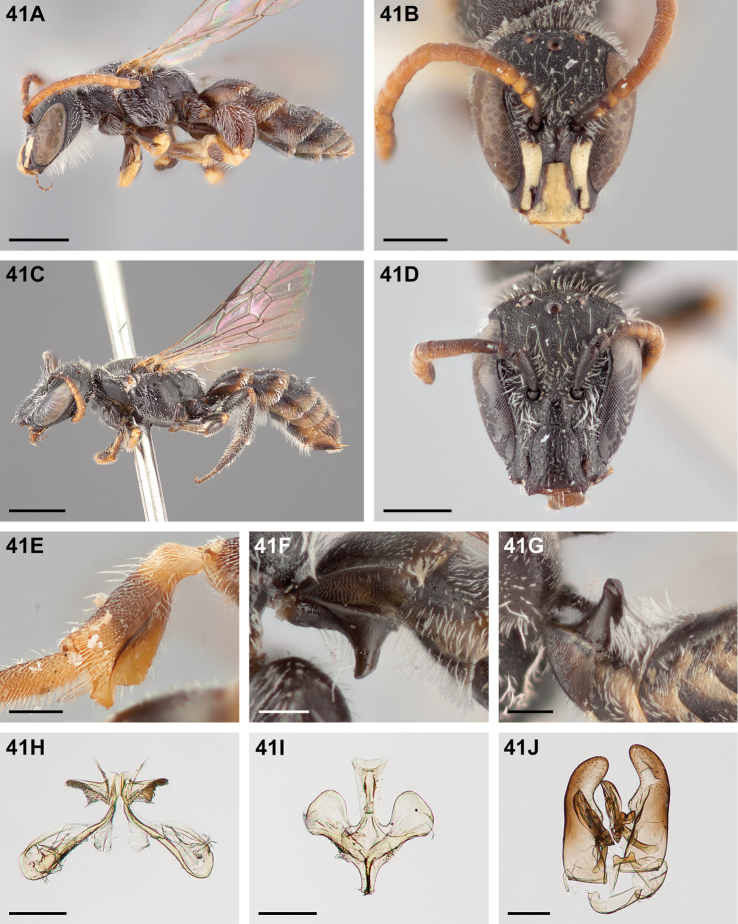
*Chilicola mavida*: **A** male habitus, lateral view, scale bar 1 mm **B** male head, frontal view, scale bar 0.5 mm **C** female habitus, lateral view, scale bar 1 mm **D** female head, frontal view, scale bar 0.5 mm **E** male metatibia, posterior view, scale bar 0.25 mm **F** male S1 process, lateral view^†^, scale bar 0.25 mm **G** male S1 process, apical view, scale bar 0.25 mm **H** male S7, scale bar 0.25 mm **I** male S8, scale bar 0.25 mm **J** male genital capsule, scale bar 0.25 mm.

###### Material studied

(21 males & 19 females): **Holotype** (male): Region IV, Baños del Toro, *S 29.838°, W 70.026 °, 3202m*, i.1970, Toro (AMNH); **Allotype** (female): same data as holotype (AMNH); **Region IV**: one paratype male, same data as holotype (AMNH); five paratype males, Laguna Dam, *S 30.206°, W 70.042°, 3170m*, 5.xii.1950, Ross & Michelbacher (AMNH); one paratype male, Río Laguna, *S 30.191°, W 70.047°, 3000m*, i.1970, Dazarola (AMNH); one female, same locality as holotype, 1970, collector unknown (AMNH); one male, Río Laguna, *S 30.191°, W 70.047°, 3000m*, 1970, H. Toro (AMNH); one male, same locality, 8.i.1970, H. Toro (PUCV); one male, CH-41 km 165, S of La Laguna, S 30.1479°, W 70.0518°, 2767m, 15.xii.2013, L. Packer (PCYU); one male, 41-CH km km 171, N of Emb La Laguna, S 30.1787°, W 70.0473°, 2942m, 15.xii.2013, J. Postlethwaite, CCDB-19989 D05 (PCYU); two females, W of Junta Toro, 41-CH km 146, S 29.9675°, W 70.1285°, 1977m, 8.xi.2013, S. Monckton, pan traps, CCDB-19989 D01 // PCYU 0021694, CCDB-19989 D02 // PCYU 0021695 (PCYU); one male, 41-CH km, 137.8, S 29.9670°, W 70.1936, 800m, 8.xi.2013, S. Monckton, pan trap, CCDB-19989 B10 // PCYU 0021684 (PCYU); one male, E of Vicuña, 127.8 km, S 29.9777°, W 70.2293°, 1711m, 12-19.ix.2010, L. Packer (PCYU); one female, Elqui Prov., Pangue, S 30.1539°, W 70.6639°, 1686m, 11-30.ix.2004, A. Ugarte (PCYU); two males, Elqui: El Pangue, 24 km S of Vicuña, *S 30.168°, W 70.663°, 1711m*, 31.x.1992, Snyder & Sharkov (AMNH); one male and one female, Elqui Prov., 26 km S of Vicuña, *S 30.19°, W 70.661°, 1691m*, 29.x.1992, Rozen, Sharkov, Snyder (AMNH); three females, Limarí Prov., Las Mollacas, E Monte Patria, *S 30.753°, W 70.657°, 1142m*, 13.x.1994, Rozen, Quinter, Ascher, on *Oxalis* (AMNH); **Region V**: one female, Portillo Aduanas, S 32.8304°, W 70.0905°, 3210m, 10.i.2009, L. Packer, on *Phacelia*, CHI09-13-2-011 (PCYU); two males and six females, Portillo, S 32.8372°, W 70.1296°, 2870m, 11.i.2009, L. Packer, on *Phacelia*, CHI09-13-1-003, CHI09-13-1-005, CHI09-13-1-004, CHI09-13-1-009 (PCYU); two females, same data (BBSL); two females, same data (CTMI); one female, same locality, 30.xii.2008, L. Packer, CHI09-2-1-018 (PCYU); one female, Portillo, S 32.8507°, W 70.1339°, 2612m, 30.xii.2009, L. Packer, CHI09-2-2-031 (PCYU); one male and one female, same data, on *Stachys*, CHI09-2-2-039, CHI09-2-2-0398 (PCYU); one male, Guardia Vieja mountain, Aconcagua Prov., *S 32.904°, W 70.272°, 1600m*, 17.ix.1964, L. E. Peña (AMNH).

###### Variation.

Some females have a yellow-brown or yellow spot on the lower paraocular area below the anterior tentorial pit.

###### Distribution.

Central Andean Cordillera and upper Coquimban Desert, just extending into Southern Andean Cordillera, from Baños del Toro & Embalse La Laguna west to El Pangue (Region IV) and south to Portillo (Region V); 1142–3210m a.s.l.

###### Ecology.

Collected on *Oxalis* L. (3 records), *Phacelia* (9 records), and *Stachys* L. (2 records). Recorded September to January.

##### Chilicola (Heteroediscelis) mayu

Taxon classificationAnimaliaHymenopteraColletidae

Monckton sp. n.

http://zoobank.org/F43A1A23-AE85-4D3B-B04E-14AC79A4D39A

**Figure 42.**
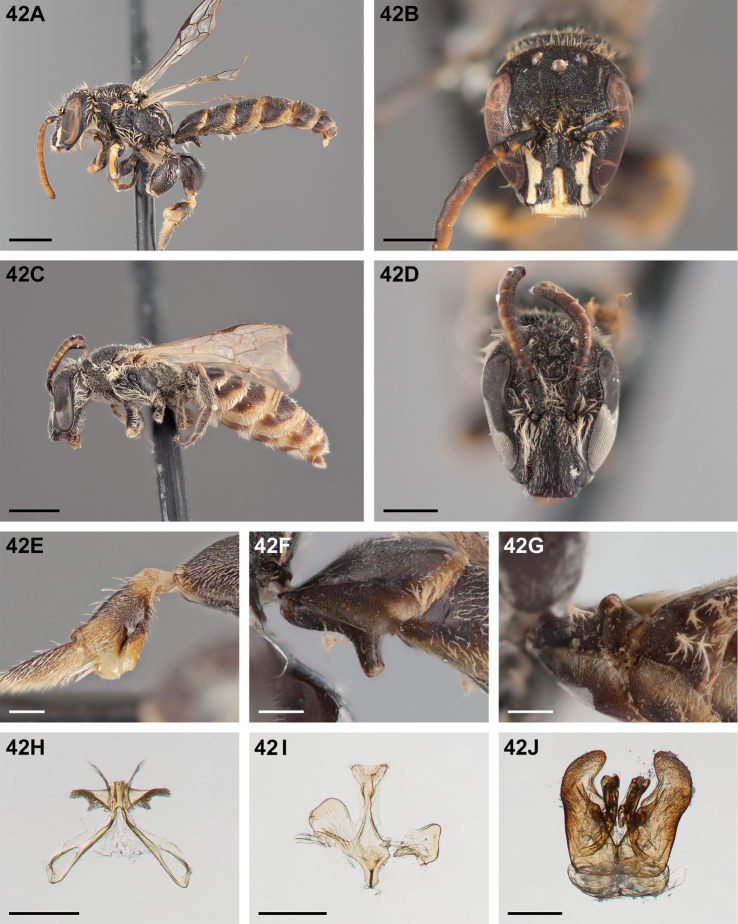
*Chilicola mayu*: **A** male habitus, lateral view, scale bar 1 mm **B** male head, frontal view, scale bar 0.5 mm **C** female habitus, lateral view, scale bar 1 mm^†^ **D** female head, frontal view, scale bar 0.5 mm **E** male metatibia, posterior view^†^, scale bar 0.25 mm **F** male S1 process, lateral view, scale bar 0.25 mm **G** male S1 process, apical view, scale bar 0.25 mm **H** male S7, scale bar 0.25 mm **I** male S8, scale bar 0.25 mm **J** male genital capsule, scale bar 0.25 mm.

Figs 8B ; 10C–D ; 23B ; 28A–B ; 42A–J ; 49

###### Diagnosis.

Males are diagnosable by the combination of first flagellomere longer than wide (≥1.1×), malar space shorter than clypeal lateral (<0.95×), and S1 process with anterior apical angle broadly rounded in lateral view. Females are diagnosable by the combination of clypeus strongly convex and in lateral view bulging ~1.5OD above the compound eye, metatibia expanded (≥0.25× as deep as long), and T3 apicolateral hair band sparse, not tomentose apicolaterally as on T1-T2.

###### Description.

**Male (Holotype).** Length 6.5mm, forewing length 3.3mm, head width 1.3mm, thorax width 1.4mm, median ocellar diameter (OD) 0.12mm.

*Colouration*: Black-brown, following parts yellow: labrum; inverted T on clypeus, with apex dark laterad; lower paraocular area extending to level of transverse epistomal suture medially, further laterally but not reaching lower tangent of antennal socket; apicoventral spot on scape and pedicel; apex of profemur broadly on anterior surface, narrowly on posterior surface; dorsal surface of protibia, including dorsal half of anterior surface; apicodorsal rim of mesofemur, wider anteriorly; wide basal and narrow apical ring on mesotibia, as well as apical half of anterior surface; apicodorsal rim of metafemur; ventral surface of metatibia, as well as dorsal surface in basal quarter, narrowly yellow apically except broad around apical lamina and posterior concavity; basal half of metabasitarsus ventrally; anterior spot on tegula; apex of T7. Following parts yellow-brown: ventral surface of antenna from F1 to terminal flagellomere, suffused with darker brown basally; apical half of prodistitarsus; posterior surface of mesobasitarsus ventrally. Posterior metatibial carina black. Metasoma black, T1-T6 marginal zones amber to translucent yellow at margins.

*Pubescence*: White, hairs generally short (0.5OD) and sparse, not especially plumose; on lower paraocular area and around antennal base moderately long and moderately dense (1OD); genal beard long and dense (0.5–3OD) longest at midlength; propodeal hairs moderately long and dense dorsolaterally (0.5–2OD); T1-T3 apicolateral patches of tomentum (0.5–1OD).

*Surface sculpture*: Microsculpture imbricate, integument generally dull; punctures generally small and deep. Clypeus and lower paraocular and supraclypeal areas moderately densely punctate (i=1-2d) except clypeus sparsely punctate in lower half (i=2-3d), supraclypeal area densely punctate toward lateral margin (i≤d); frontal area very densely punctate (i≤0.5d), moderately densely punctate around ocelli (i=1-2d); ocellocular space densely punctate (i≤d); vertexal area rugose and densely punctate (i≤d); scape shallowly and moderately densely punctate (i=1-2d) densely punctate apicoventrally (i≤d); genal area microstriate and densely punctate (i≤d); hypostomal area coarsely imbricate and densely punctate (i≤d); pronotum moderately densely punctate (i=1-2d), lateral surface coarsely imbricate; mesoscutum densely punctate (i=0.5-1d) sparsely punctate toward lateral margin (i=1-2d) punctures crowded around median (i≤d); scutellum irregularly punctate (i=1-3d) punctures crowded around median (i≤d); mesepisternum irregularly punctate, sparse below scrobe and anterior of episternal groove (i≥2d), impunctate just dorsad of scrobe, otherwise dense on hypoepimeral area (i≤d); metanotum irregularly punctate (i=1-3d) punctures crowded around median and anteriorly (i≤d); metepisternum moderately densely punctate (i=1-2d) distinctly longitudinally striate anterodorsally; metapostnotum rugose; propodeum coarsely imbricate, rugulose posteriorly, moderately densely punctate throughout (i=1-2d); T1-T5 punctures small and sparse (i=1-3d); T6 punctures small and sparse (i≥2d), coarsely imbricate posteriorly; T7 punctures small and sparse (i≥2d); marginal zones of terga weakly imbricate and shiny, minutely punctate.

*Structure*: Labrum ~2.67× wider than long (24:9); malar space ~0.85× as long as clypeal lateral (6:7); LOT below anterior tentorial pits; length of clypeus equal to maximum width in frontal view (30:30) extending for less than half of its length beyond LOT and strongly convex above anterior tentorial pit, in lateral view bulging ~1.5-2OD above compound eye; median longitudinal groove present on clypeus except on apex; subantennal sutures 1.1× as long as the shortest distance between them (16:14.5); IAD ~1.7× AOD (14.5:8.5); scape ~2.6× as long as maximum width (26:10); pedicel slightly longer than wide (9.5:9); F1 longer than wide (10:9); F2 1.3× as long as F1 and shorter than F3 (F2:F3 - 13:15); UOD ~1.4× LOD, IOD ~1.1× UOD (UOD:IOD:LOD - 55:60:39); frontal line carinate only between antennal bases for a length less than OD, undefined above; MOC subequal to width of head (87:89); OOC 0.75× IOC (15:20); genal area ~0.6× as wide as compound eye in lateral view (17:29); ratio of lengths of mesoscutum: scutellum: metanotum: metapostnotum - 61:23:12:17; probasitarsus ~2.9× as long as maximum depth (25:8.5); prodistitarsus 0.9× as long as preceding three tarsomeres combined (from II to V - 6.5:5:4:14); metafemur nearly twice as long as maximum depth (67:40); summit of metatibial ventral convexity rounded and near tibial apex; metatibia ~1.67× as long as maximum depth (52:31); in apical view apical lamina of metatibia broad and thick, length ~1.2× OD (11:9); ventral metatibial carina sublinear and absent basally, originating at midpoint of weakly sigmoid posterior carina; metabasitarsus ~3.5× as long as maximum depth (46:13); S1 process apical surface with longitudinal median ridge, in lateral view anterior and posterior margins subparallel apically and median ridge projecting posteriorly from apex. S7 apodemal arm sclerotized margin recurved mesad, dividing membranous portion of arm; ventral lobe narrow, almost linear; dorsal lobe roughly triangular, wide at base and narrow apically, with row of setae long basally and shorter apically; apex of disc nearly linear. S8 lateral process ~1.1× wider than long, anteromedial sclerotized margin following marginal contour. Gonobase apicoventral process weakly emarginate.

**Female (Allotype).** Length 6.1mm, forewing length 3.1mm, head width 1.1mm, thorax width 1.3mm, median ocellar diameter (OD) 0.10mm.

*Colouration*: Black to black-brown except as follows: labrum black to yellow-brown on apex; mandible with yellow basal spot, translucent yellow with apex black. Following parts yellow-brown: apicoventral spot on F1 and F2; ventral surface of antenna from F3 to terminal flagellomere, with pale apical bands; apicodorsal rim of metafemur; basidorsal spot on metatibia. Following parts yellow: dorsal surface of protibia; apical two-thirds of prodistitarsus; basidorsal and apicodorsal spots on mesotibia. Metasoma black, T1-T5 marginal zones yellow to translucent yellow at margins.

*Pubescence*: As in male except as follows: clypeal hairs short and sparse (0.5OD); genal beard moderately dense (0.5–2.5OD); discs of mesoscutum, scutellum, and metanotum with sparse short hairs (≤0.5OD), longer and denser toward lateral margin as follows: mesoscutum (0.5OD), scutellum (1–2OD) also on posterior margin, longest posteromedially, metanotum (≤2OD); propodeal hairs moderately long and dense dorsolaterally (0.5–1.5OD); scopae on metafemur and metatibia (1–1.5OD); T1-T2 apicolateral patches of tomentum (0.5–1OD), T3 apicolateral hair band sparse, not tomentose; S1 hairs long and moderately dense (≤2OD); S2 scopal hairs (1–2OD).

*Surface sculpture*: As in male except as follows: lower paraocular area densely punctate (i=d); frontal area moderately densely punctate (i=1-2d); vertexal area densely punctate (i≤d); scape shallowly and moderately densely punctate (i=1-2d); genal area weakly microstriate and moderately densely punctate (i=1-2d); hypostomal area imbricate and moderately densely punctate (i=1-2d); pronotum densely punctate (i=0.5-1d); mesoscutum densely punctate (i=d) moderately densely punctate toward lateral margin (i=1-2d) punctures crowded around median (i≤d); scutellum moderately densely punctate (i=0.5-2d) punctures crowded around median (i≤d); mesepisternum irregularly punctate, sparse below scrobe (i=1-3d), sparser anterior of episternal groove (i≥3d), impunctate just dorsad of scrobe, otherwise dense on hypoepimeral area (i≤d); metanotum moderately densely punctate (i=0.5-2d) punctures crowded around median and anteriorly (i≤d); metapostnotum rugose; propodeum coarsely imbricate and moderately densely punctate (i=1-2d); T1-T5 punctures small and sparse (i=1-3d on T1-T3; i≥2d on T4-T5); T6 moderately densely punctate (i=1-2d).

*Structure*: Labrum 2.3× wider than long (23:10); malar space ~0.6× as long as clypeal lateral (5:8.5); LOT below anterior tentorial pits; length of clypeus subequal to maximum width in frontal view (33.5:32) extending for less than half of its length beyond LOT and convex above anterior tentorial pit as in male; median longitudinal groove weak on clypeus; length of subantennal sutures subequal to shortest distance between them (13:13.5); IAD ~1.4× AOD (15:10.5); scape 3.25× as long as maximum width (26:8); pedicel ~1.4× as long as wide (9:6.5); F1 longer than wide (8:7); F2 more than half as long as F1 (4.5) and 0.75× as long as F3 (6); UOD ~1.3× LOD, IOD ~1.1× UOD (UOD:IOD:LOD - 51:58:40); frontal line carinate in lower third, flat above; MOC subequal to width of head (84:83); OOC ~0.75× IOC (14:19); genal area ~0.67× as wide as eye in lateral view (17:25); ratio of lengths of mesoscutum: scutellum: metanotum: metapostnotum – 58:22:12:15; probasitarsus ~2.9× as long as maximum depth (20:7); prodistitarsus ~0.8× as long as preceding three tarsomeres combined (from II to V - 7:6:5:15); metafemur ~2.9× as long as maximum depth (49:17); metatibia ~3.8× as long as maximum depth (61:16); metabasitarsus ~3.7× as long as maximum depth (39:10.5).

###### Type material

(5 males & 7 females). **Holotype** (male): Region IX, Cuesta Las Raíces, S 38.4299°, W 71.4489°, 1583m, 12.ii.2013, S. Monckton & R. Smith, CCDB-19989 G08 (PCYU); **Allotype** (female): same data as holotype, CCDB-19989 G09 (PCYU); **Paratypes: Region VIII**: one female, Termas de Chillán, *S 36.906°, W 71.41°, 1691m*, 13.ii.1976, H. Toro (AMNH); one male, Near Termas de Chillán, *S 36.906°, W 71.41°, 1691m*, 27.xii.2006, L. Packer (PCYU); one female, Ñuble, Las Trancas, *S 36.911°, W 71.48°, 1250m*, xii.1993, L. Peña (AMNH); one female, Las Trancas, *S 36.911°, W 71.48°, 1250m*, 11.xii.2006, L. Packer (PCYU); one male and one female, Ñuble, Las Trancas, SE Recinto in Chillán area, *S 36.911°, W 71.48°, 1250m*, ii.1987, Luis E. Peña (AMNH); one female, Chillán, Las Trancas, *S 36.911°, W 71.48°, 1250m*, 11.ii.1976, H. Toro Jr (AMNH); one female, Las Trancas, Los Nirres, S 36.9121°, W 71.5026°, 1221m, 12.xii.2006, L. Packer (PCYU); **Region VII**: one male, Río Maule, 122 km marker, S 35.9016°, W 70.6426°, 1359m, 6.i.2013, L. Packer & R. Smith (PCYU); one male, Laguna del Maule, S 36.0027°, W 70.4555°, 2227m, 9.i.2009, L. Packer, on *Phacelia*, CCDB-22789 D04 // CHI09-7-2-005 (PCYU).

###### Distribution.

Pehuén and Southern Andean Cordillera, from Cuesta Las Raíces (Region IX) north to Laguna del Maule (Region VII) and west to Valle Las Trancas (Region VIII); 1221–2227m a.s.l.

###### Ecology.

Collected on *Phacelia* (1 record). Recorded December to February.

###### Etymology.

The specific epithet was recommended by R.A. Lühr and is derived from the type locality near Lonquimay, a place name formed in part from the Quechua *mayu* meaning “river”. It is to be treated as a noun in apposition.

##### Chilicola (Heteroediscelis) neffi

Taxon classificationAnimaliaHymenopteraColletidae

Toro & Moldenke, 1979

**Figure 43.**
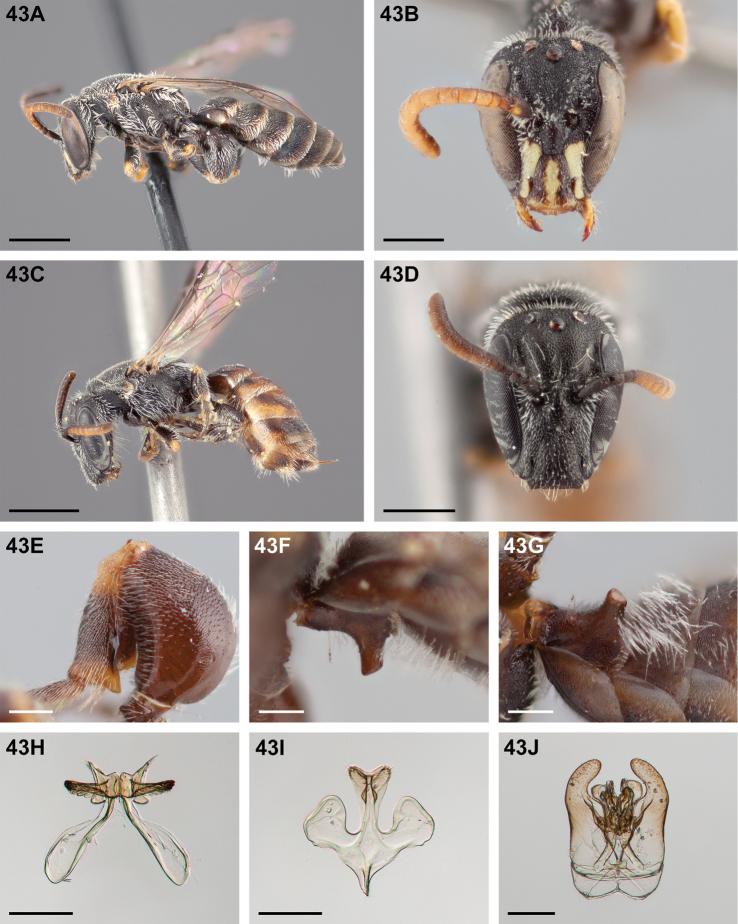
*Chilicola neffi*: **A** male habitus, lateral view, scale bar 1 mm **B** male head, frontal view, scale bar 0.5 mm **C** female habitus, lateral view, scale bar 1 mm **D** female head, frontal view, scale bar 0.5 mm **E** male metatibia, posterior view, scale bar 0.25 mm **F** male S1 process, lateral view^†^, scale bar 0.25 mm **G** male S1 process, apical view, scale bar 0.25 mm **H** male S7, scale bar 0.25 mm **I** male S8, scale bar 0.25 mm **J** male genital capsule, scale bar 0.25 mm.

Figs 3A ; 4A–B ; 5C ; 25A ; 26C–D ; 43A–J ; 49

Chilicola neffi Toro & Moldenke, 1979: 118–119 (male holotype, female allotype, AMNH [examined]). Packer 2008: 74, 77–78 (phylogeny). Montalva and Ruz 2010: 29 (checklist). Moure et al. 2012 (catalogue). Ascher and Pickering 2015 (checklist).

###### Diagnosis.

Males and females of *Chilicola neffi* are differentiated from all other species of the subgenus except for *Chilicola packeri* by the longitudinal depressions on the paraocular area. Males are distinguished from the latter by the broader metabasitarsus, ≤3.9× as long as maximum depth (≥4× in *Chilicola packeri*). Females are distinguished by the darker metasoma, with T1-T3 mostly dark brown; lighter-coloured females have orange-brown colouration restricted to the marginal zone and pregradular area of T1-T3, with disc always dark brown in apical half (in *Chilicola packeri* T1-T3 are almost entirely orange-brown, with at most premarginal line dark brown).

###### Description.

**Male.** Length 5.0–5.7mm, forewing length 3.1–3.2mm, head width 1.0–1.1mm, thorax width 1.1–1.2mm, median ocellar diameter (OD) 0.10–0.12mm.

*Colouration*: Black-brown, following parts yellow: labrum; variable on clypeus from inverted T to median line only, apex dark laterad; lower paraocular area extending <1OD above transverse epistomal suture, not reaching ventral tangent of antennal socket; apicoventral spot on scape; apex of profemur broadly on anterior surface, narrowly on posterior surface; dorsal surface of protibia, including dorsal half of anterior surface; apicodorsal rim of mesofemur, wider anteriorly; narrow basal ring and apicodorsal spot on mesotibia; apicodorsal rim of metafemur; basal quarter of metatibia, as well as apical ring narrow on anterior surface, extending to summit of ventral convexity and widened to apical quarter on posterior surface; anterior spot on tegula. Following yellow-brown: apicoventral surface of pedicel, sometimes brown; ventral surface of flagellum, narrowing basally on F1, remaining flagellomeres variably suffused with brown basally and terminal flagellomere sometimes brown; protrochanter apical rim posteriorly; anterior surface of probasitarsus; apical half of prodistitarsus. Apical one-third of mesodistitarsus brown. Posterior metatibial carina black. Metasoma dark brown, T1-T6 marginal zones yellow-brown to translucent yellow at margins. Apex of T7 yellow to translucent yellow at margin.

*Pubescence*: White, hairs generally short (0.5OD) and sparse, not especially plumose; on lower paraocular area and around antennal base moderately long and moderately dense (1OD); genal beard long and moderately dense (0.5–3OD) longest at midlength; discs of mesoscutum, scutellum, and metanotum mostly bare, few short hairs (0.5OD), denser and as long or longer toward lateral margin as follows: mesoscutum (0.5OD), scutellum (0.5–1OD) also on posterior margin, longest posteromedially, metanotum (≤2OD); propodeal hairs moderately long and dense dorsolaterally (0.5–1.5OD); T1-T3 apicolateral patches of tomentum (0.5–1OD); S2 hairs long and dense (1.5OD basolaterad, 1OD distilaterad, shorter mesad).

*Surface sculpture*: Microsculpture imbricate, integument generally dull; punctures generally small and deep. Clypeus and supraclypeal and lower paraocular areas moderately densely punctate (i=1-2d) except clypeus sparsely punctate in apical half (i=2-3d), supraclypeal area densely punctate toward lateral margin (i≤d); frontal area very densely punctate (i≤0.5d); ocellocular space moderately densely punctate (i=1-2d); vertexal area very densely punctate (i≤0.5d); scape shallowly and sparsely punctate (i=2d); genal area microstriate and densely punctate (i≤d); hypostomal area weakly imbricate and moderately densely punctate (i=1-2d); pronotum densely punctate (i=0.5-1d), lateral surface coarsely imbricate; mesoscutum densely punctate (i=0.5-1d); scutellum moderately densely punctate (i=0.5-2d) punctures crowded around median (i≤0.5d); mesepisternum irregularly punctate, moderately dense below scrobe (i=0.5-2d), sparser anterior of episternal groove (i=1-2d), irregularly spaced on hypoepimeral area (i≤2d); metanotum densely punctate (i=0.5-1d) punctures crowded around median and anteriorly (i≤0.5d); metepisternum densely punctate (i≤d) distinctly longitudinally striate anterodorsally; metapostnotum rugose; propodeum coarsely imbricate, rugulose posteriorly, moderately densely punctate (i=1-2d); T1-T5 coarsely imbricate; T1-T2 moderately densely punctate (i≤2d); T3-T5 densely punctate throughout (i≤d); T6 moderately densely punctate (i=1-2d) and coarsely imbricate posteriorly; T7 moderately densely punctate (i=1-2d); marginal zones of terga weakly imbricate and shiny, minutely punctate.

*Structure*: Labrum ~2.6× wider than long (21:8); malar space ~0.4× as long as clypeal lateral (3:7); LOT below anterior tentorial pits; length of clypeus subequal to maximum width in frontal view (28:28) extending for less than half of its length beyond LOT; median longitudinal groove on clypeus weakly present in dorsal half, absent ventrad; subantennal sutures 1.5× as long as the shortest distance between them (15:10); IAD ~1.6× AOD (11:7); scape ~2.67× as long as maximum width (24:9); pedicel as long as wide (8:8); F1 shorter than wide (6:8.5); F2 ~1.33× as long as F1 and same length as F3 (F2:F3 - 8:8); UOD ~1.4× LOD, IOD ~1.1× UOD (UOD:IOD:LOD - 43:47:31); frontal line carinate between antennal bases for a length less than 1OD, flat just below median ocellus for a length of ~1OD, otherwise undefined; MOC longer than width of head (78:74); OOC ~0.7× IOC (11:16); genal area ~0.67× as wide as compound eye in lateral view (17:27); ratio of lengths of mesoscutum: scutellum: metanotum: metapostnotum - 53:20:9:14; probasitarsus 3.5× as long as maximum depth (21:6); length of prodistitarsus subequal to that of preceding three tarsomeres combined (from II to V - 6:5:4:14); metafemur ~1.75× as long as maximum depth (59:34); summit of metatibial ventral convexity at approximately two-thirds tibial length; metatibia ~2.1× as long as maximum depth (49:23); in apical view apical lamina of metatibia short and thick, length ~0.67× OD (6:9); ventral metatibial carina greatly reduced and originating in apical half of posterior carina, the latter sharply inflected near base; metabasitarsus ~3.75× as long as maximum depth (32:8.5); S1 process apical surface weakly convex, in lateral view anterior and posterior margins subparallel apically. S7 apodemal arm sclerotized margin entire, enclosing membranous portion of arm; ventral lobe ~0.5× as broad as basal attached length; dorsal lobe somewhat anteriorly curved and digitiform, apex widened relative to midlength, with row of broad setae mesobasally to apex; apex of disc disinctly emarginate. S8 lateral process ~0.8× as wide as long, lateral process anteromedial sclerotized margin having distinct convex incursion appearing swollen. Gonobase apicoventral truncate process biconvex and distinctly notched by ~1.5× width of one convexity.

**Female.** Length 4.4–5.6mm, forewing length 2.6–2.8mm, head width 0.9–1.0mm, thorax width 1.0–1.1mm, median ocellar diameter (OD) 0.10mm.

*Colouration*: Black to black-brown except as follows: mandible with yellow basal spot, translucent yellow in apical half except apex translucent brown. Following parts yellow-brown: ventral surface of antenna from F3 to terminal flagellomere, flagellomeres suffused with brown basally, terminal flagellomere brown. Following parts yellow: dorsal surface of protibia; apical half of prodistitarsus; wide basal and narrow apical ring on mesotibia; anterior spot on tegula, sometimes faint. Following parts brown: apical one-third of mesodistitarsus; apicodorsal rim of metafemur; wide basal ring on metatibia. Metasoma variable from mostly black to mostly orange-brown, at least marginal zones of T1-T5 narrowly translucent yellow; individuals with lighter metasomae have T1-T5 marginal zones more widely yellow to translucent yellow at margins, and T1-T3 with pregradular area yellow to yellow-brown, but disc always mostly brown.

*Pubescence*: As in male except as follows: clypeal hairs short and sparse (0.5OD); genal beard sparse (0.5–2OD); discs of mesoscutum, scutellum, and metanotum with sparse short hairs (≤0.5OD), longer and denser toward lateral margin as follows: mesoscutum (0.5OD), scutellum (1–1.5OD) also on posterior margin, longest posteromedially, metanotum (≤1.5OD); scopae on metafemur and metatibia (1–1.5OD); T1-T2 apicolateral patches of tomentum (0.5–1OD), T3 apicolateral hair band sparse, not tomentose; S1 hairs long and moderately dense (≤2.5OD); S2 scopal hairs (1–3OD).

*Surface sculpture*: As in male except as follows: clypeus moderately densely punctate throughout (i=1-2d); supraclypeal area irregularly punctate (i=1-2d) densely punctate toward lateral margin (i≤d); lower paraocular area densely punctate (i=d); frontal area densely punctate (i≤d); ocellocular space moderately densely punctate adjacent to compound eye (i=1-2d) becoming impunctate adjacent to lateral ocellus; vertexal area densely punctate (i≤d); scape shallowly and moderately densely punctate (i=1-2d); genal area densely punctate (i=d); hypostomal area weakly imbricate and sparsely punctate (i≥2d); lateral surface of pronotum coarsely imbricate; mesoscutum and scutellum densely punctate (i=0.5-1d) punctures crowded around median (i≤0.5d); mesepisternum irregularly punctate, moderately dense below scrobe (i=1-3d), sparser anterior of episternal groove (i=1-4d), irregularly spaced on hypoepimeral area (i≤2d); metanotum densely punctate (i=0.5-1d) punctures crowded around median and anteriorly (i≤0.5d); metapostnotum rugose, more weakly microsculptured posteriorly; propodeum moderately densely punctate (i=1-2d); T1-T5 punctures small and sparse (i=1-3d); T6 moderately densely punctate (i=1-2d).

*Structure*: Labrum 2.3× wider than long (23:10); malar space ~0.33× as long as clypeal lateral (2.5:7.5); LOT below anterior tentorial pits; clypeus subequal in length to maximum width in frontal view (28.5:27) extending for less than half of its length beyond LOT; median longitudinal groove weak on clypeus; subantennal sutures 1.25× as long as the shortest distance between them (12.5:10); IAD ~1.8× AOD (11.5:6.5); scape ~3.33× as long as maximum width (20:6); pedicel longer than wide (8:6); F1 slightly shorter than wide (5:5.5); F2 shorter than F1 and shorter than F3 (F2:F3 - 3:4); UOD ~1.4× LOD, IOD ~1.05× as long as UOD (UOD:IOD:LOD - 40:42.5:29); frontal line carinate in lower half, flat above; MOC longer than width of head (75:66.5); OOC ~0.67× IOC (10:15); genal area ~0.7× as wide as eye in lateral view (16:23); ratio of lengths of mesoscutum: scutellum: metanotum: metapostnotum – 49:18:8:11; probasitarsus 3.5× as long as maximum depth (21:6); length of prodistitarsus subequal to that of preceding three tarsomeres combined (from II to V - 5:4:3:13); metafemur ~3× as long as maximum depth (41:14); metatibia ~4.2× as long as maximum depth (52:12.5); metabasitarsus 3.75× as long as maximum depth (30:8).

###### Material studied

(47 males & 91 females). **Holotype** (male): Region II, Taltal, *S 25.406°, W 70.481°, 39m*, 30.ix.1972, J. Neff (AMNH); **Allotype** (female): same data as holotype (AMNH); **Region II**: one paratype female, same data as holotype, on *Oxalis* (AMNH); one paratype female, same locality and collector as holotype, 1.x.1972, on *Cristaria* (AMNH); one paratype female, same locality and collector as holotype, 1.x.1972, on *Balbisia peduncularis* (AMNH); two males and five females, S of Taltal, Ruta 1 km 9.6, S 25.5059°, W 70.4167°, 608m, 1.xii.2014, L. Packer (PCYU); two males and one female, SE of Taltal, B-902 km 1, S 25.5023°, W 70.4122°, 564m, 2.xii.2014, L. Packer (PCYU); two males and one female, SE of Taltal, Ruta 1, km 11, S 25.4936°, W 70.4213°, 499m, 27.ix-17.xi.2013, J. Postlethwaite & S. Monckton, blue cup (PCYU); one male and one female, same data, yellow cup (PCYU); one male, same locality, 17.xi.2013, J. Postlethwaite, white pan, CCDB-22789 D08 (PCYU); one female, Ruta 5, km 1006, S 26.2495°, W 70.4805°, 606m, 19.xi.2002, J. Grixti & A. Zayed, on *Nolana* (PCYU); one male and two females, N of Taltal, Ruta 1 km 32.1, S 25.3426°, W 70.4444°, 17m, 2.xii.2014, L. Packer (PCYU); one male and one female, Beach 16 km N of Taltal, *S 25.284°, W 70.45°, 21m*, 20.xi.2002, J. Grixti & A. Zayed, yellow pan (CTMI); two females, same data (PCYU); one male and two females, N of Taltal, Ruta 1, km 40, S 25.2692°, W 70.4378°, 37m, 5.xi.2013, S. Monckton (PCYU); eight males and twenty-three females, N of Taltal, Ruta 1 km 43.6, S 25.2463°, W 70.4321°, 29m, 2.xii.2014, L. Packer (PCYU); one female, N of Taltal, *S 25.231°, W 70.437°, 20m*, 20.xi.2002, J. Grixti & A. Zayed, blue pan (PCYU); one male and one female, S of Paposo, Ruta 1, km 44, S 25.2433°, W 70.4336°, 42m, 5.xi.2013, S. Monckton (PCYU); two males and eight females, N of Taltal, *S 25.231°, W 70.437°, 20m*, 20.xi.2002, J. Grixti & A. Zayed, yellow pan (PCYU); two males and three females, same data, white pan (PCYU); two females, 13 km S of Paposo, S 25.1612°, W 70.4423°, 36m, 28.ix.2002, J. Grixti & A. Zayed, on *Oxalis bulbocastanum* (PCYU); three males and five females, S of Paposo, Ruta 1, km 85.5, S 25.1519°, W 70.4552°, 5m, 5.xi.2013, S. Monckton (PCYU); seven males and sixteen females, ~13km S of Paposo, *S 25.111°, W 70.484°, 21m*, 29.ix-4.x.2002, J Grixti & A Zayed, yellow pan (PCYU); four females, same data, 28.ix-29.ix.2002 (PCYU); six males and one female, Paposo, *S 25.003°, W 70.463°, 39m*, 1.x.1983, R. Aldunate (PUCV); one male and one female, same locality and date, F. Rodriguez (BBSL); two males and one female, 29 km N of Paposo, S 24.7830°, W 70.5402°, 59m, 29.x.2014, J. Postlethwaite, pan traps (PCYU); one male, same data, vane trap (PCYU); one male and two females, Cuesta Paposo, S 25.0186°, W 70.4271°, 676m, 24.x.2013, S. Monckton, PCYU 0021578, PCYU 0021645, CCDB-22791 E02 // PCYU 0021646 (PCYU); one male and three females, same locality and collector, 5.xi.2013, CCDB-19989 A01 // PCYU 0021663, CCDB-19989 A02 // PCYU 0021664, PCYU 0021579, PCYU 0021580 (PCYU).

###### Distribution.

Northern coastal extension of Intermediate Desert, from Taltal north to 29 km N of Paposo and inland SE of Taltal (Region II); 5–676m a.s.l.

###### Ecology.

Collected on *Balbisia peduncularis* (Lindl.) D.Don (1 record), *Cristaria* (1 record), *Nolana* (1 record), *Oxalis* (1 record), and *Oxalis bulbocastanum* Phil. (2 records). Recorded September to December.

###### Comments.

One female from Ruta 5, km 1006 (C.neff.072) has the metasomal colouration of *Chilicola packeri*, but is identified as *Chilicola neffi* based on metabasitarsus dimensions and collecting locality, which is most consistent with the observed range of this species.

##### Chilicola (Heteroediscelis) packeri

Taxon classificationAnimaliaHymenopteraColletidae

Monckton sp. n.

http://zoobank.org/C042F9C0-96B6-448C-B960-73568FD30193

**Figure 44.**
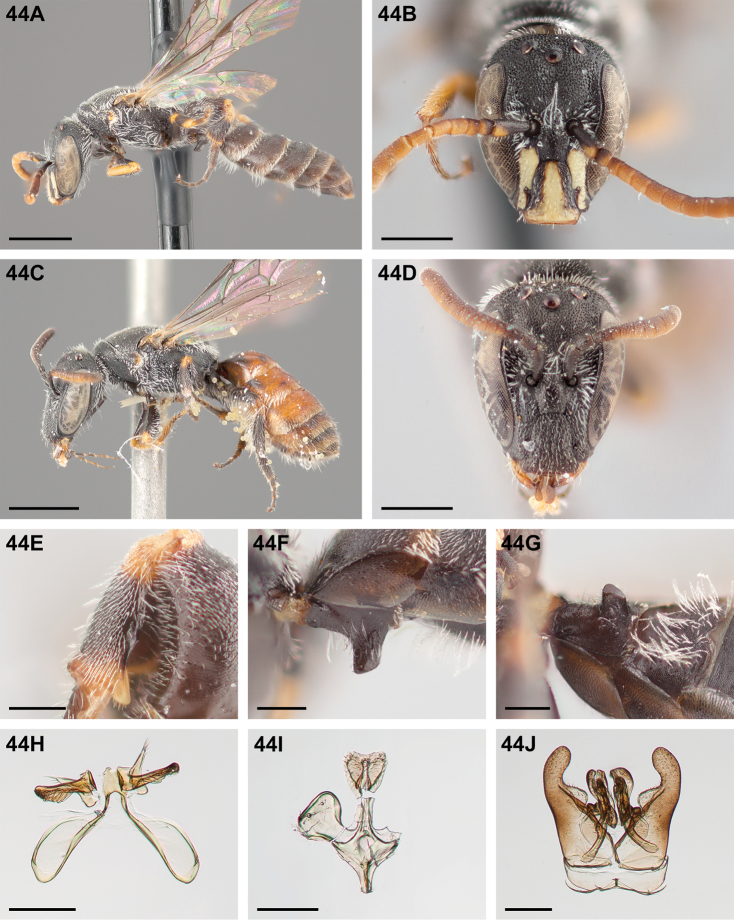
*Chilicola packeri*: **A** male habitus, lateral view, scale bar 1 mm **B** male head, frontal view, scale bar 0.5 mm **C** female habitus, lateral view, scale bar 1 mm **D** female head, frontal view, scale bar 0.5 mm **E** male metatibia, posterior view, scale bar 0.25 mm **F** male S1 process, lateral view, scale bar 0.25 mm **G** male S1 process, apical view, scale bar 0.25 mm **H** male S7 (C.pckr.013), scale bar 0.25 mm **I** male S8 (C.pckr.013), scale bar 0.25 mm **J** male genital capsule (C.pckr.013), scale bar 0.25 mm.

Figs 4C–D ; 26A–B ; 44A–J ; 49

###### Diagnosis.

Males and females of *Chilicola packeri* are differentiated from all other species of the subgenus except for *Chilicola neffi* by the longitudinal depressions on the paraocular area. Males are distinguished from the latter by the narrower metabasitarsus, ≥4× as long as maximum depth (≤3.9× in *Chilicola neffi*). Females are distinguished by the first three metasomal segments orange-brown, with the premarginal line of T1-T3 entirely dark or with paired lateral dark spots, and disc always entirely orange-brown (in *Chilicola neffi* T1-T3 are dark brown, or at most orange-brown only in marginal zone and pregradular area, with disc always dark brown in apical half).

###### Description.

**Male (Holotype).** Length 5.3mm, forewing length 3.0mm, head width 1.0mm, thorax width 1.1mm, median ocellar diameter (OD) 0.10mm.

*Colouration*: Black-brown, following parts yellow: labrum; inverted T shape on clypeus, apex dark laterad; lower paraocular area extending ~1OD above transverse epistomal suture, not reaching ventral tangent of antennal socket; apicoventral surface of scape and pedicel; ventral surface of antenna from F1 to terminal flagellomere, narrowing basally on F1, remaining flagellomeres suffused with brown basally; protrochanter apical rim posteriorly; apex of profemur broadly on anterior surface, narrowly on posterior surface; dorsal surface of protibia, including dorsal half of anterior surface; anterior surface of probasitarsus; apical half of prodistitarsus; apicodorsal rim of mesofemur, wider anteriorly; wide basal and apical rings on mesotibia, wider on apical anterior surface; basal spot on anterior surface of mesobasitarsus; apicoventral rim of metatrochanter; apicodorsal rim of metafemur; basal quarter of metatibia, and apical ring narrow on anterior surface, extending to summit of ventral convexity and widened to apical quarter on posterior surface; basal quarter of metabasitarsus ventrally; anterior spot on tegula. Apicoventral margin of metacoxa translucent yellow. Posterior metatibial carina black. Apex of T7 yellow-brown. Metasoma dark brown, T1-T6 marginal zones yellow-brown to translucent yellow at margins.

*Pubescence*: White, hairs generally short (0.5OD) and sparse, not especially plumose; on lower paraocular area and around antennal base moderately long and moderately dense (1OD); genal beard long and moderately dense (0.5–2.5OD) longest at midlength; discs of mesoscutum, scutellum, and metanotum mostly bare, few short hairs (0.5OD), denser and as long or longer toward lateral margin as follows: mesoscutum (0.5OD), scutellum (1–2OD) also on posterior margin, longest posteromedially, metanotum (≤2OD); propodeal hairs moderately long and dense dorsolaterally (0.5–1.5OD); T1-T3 apicolateral patches of tomentum (0.5–1OD); S2 hairs long and dense (1.5OD basolaterad, 1OD distilaterad, shorter mesad).

*Surface sculpture*: Microsculpture imbricate, integument generally dull; punctures generally small and deep. Clypeus moderately densely punctate (i=1-2d) very sparsely punctate medially (i≥3d); supraclypeal area irregularly punctate (i=1-3d) densely punctate toward lateral margin (i≤d); lower paraocular area densely punctate (i=d); frontal area very densely punctate (i≤0.5d); ocellocular space densely punctate adjacent to compound eye (i≤d) becoming impunctate adjacent to lateral ocellus; vertexal area very densely punctate (i≤0.5d); scape shallowly and irregularly punctate (i=1-3d); genal area microstriate and densely punctate (i≤d); hypostomal area weakly imbricate to glossy apically and sparsely punctate (i≥2d); pronotum densely punctate (0.5-1d), lateral surface coarsely imbricate; mesoscutum densely punctate (i≤d); scutellum densely punctate (i=0.5-1d) punctures crowded around median (i≤d); mesepisternum irregularly punctate, moderately dense to sparse below scrobe and anterior of episternal groove (i=1-2d), irregularly spaced on hypoepimeral area (i≤2d); metanotum densely punctate (i=0.5-1d); metepisternum densely punctate (i≤d); metapostnotum coarsely rugose; propodeum coarsely imbricate, rugulose posteriorly, moderately densely punctate throughout (i=1-2d); T1-T5 coarsely imbricate and moderately densely punctate (i≤2d); T6 imbricate and sparsely punctate (i=3d) to coarsely imbricate and densely punctate (i=0.5d) posteriorly; T7 moderately densely punctate (i=1-3d); marginal zones of terga weakly imbricate and shiny, minutely punctate to impunctate.

*Structure*: Labrum ~2.6× wider than long (21:8); malar space ~0.5× as long as clypeal lateral (3:6.5); LOT below anterior tentorial pits; clypeus ~1.2× as long as maximum width in frontal view (30:26) extending for approximately one third of its length beyond LOT; median longitudinal groove on clypeus weakly present in dorsal half, absent ventrad; length of subantennal sutures subequal to the shortest distance between them (13:12.5); IAD ~1.6× AOD (11.5:7); scape ~2.4× as long as maximum width (20:8.5); pedicel shorter than wide (7:8); F1 shorter than wide (6.5:7.5); F2 ~1.2× as long as F1 and shorter than F3 (F2:F3 - 8:9); UOD ~1.4× LOD, IOD ~1.05× UOD (UOD:IOD:LOD - 42:45:31); frontal line carinate in lower third, flat above; MOC longer than width of head (74:71); OOC ~0.6× IOC (10:17); genal area ~0.6× as wide as compound eye in lateral view (12:21); ratio of lengths of mesoscutum: scutellum: metanotum: metapostnotum - 50:18:7:11; probasitarsus 4× as long as maximum depth (20:5); length of prodistitarsus ~0.7× that of preceding three tarsomeres combined (from II to V - 7:5.5:4:12); metafemur ~1.9× as long as maximum depth (55:29); summit of metatibial ventral convexity at approximately two-thirds tibial length; metatibia ~2.2× as long as maximum depth (47:21); in apical view apical lamina of metatibia short and thick, length ~0.85× OD (6:7); ventral metatibial carina greatly reduced and originating in apical half of posterior carina, the latter sharply inflected near base; metabasitarsus 4× as long as maximum depth (30:7.5); S1 process apical surface weakly convex, in lateral view anterior and posterior margins subparallel apically. S7 apodemal arm sclerotized margin entire, encircling membranous portion of arm; ventral lobe ~0.5× as broad as basal attached length; dorsal lobe somewhat anteriorly curved and digitiform, apex widened relative to midlength, with row of broad setae mesobasally to apex; apex of disc disinctly emarginate. S8 lateral process ~0.9× as wide as long, anteromedial sclerotized margin wide and following marginal contour. Gonobase apicoventral truncate process biconvex and distinctly notched by ~2× width of one convexity.

**Female (Allotype).** Length 4.8mm, forewing length 2.8mm, head width 1.0mm, thorax width 1.1mm, median ocellar diameter (OD) 0.10mm.

*Colouration*: Black to black-brown except as follows: mandible with apex black, and pale yellow at base to translucent yellow apically. Following parts yellow-brown: ventral surface of antenna from F3 to terminal flagellomere, flagellomeres suffused with brown basally, terminal flagellomere dark apically. Following parts yellow: dorsal surface of protibia; anterior surface of probasitarsus; apices of protarsomeres, including apical two-thirds of prodistitarsus; wide basal ring on mesotibia; anterior spot on tegula. Following parts brown: apicoventral spot on F1 and F2; narrow apical ring on mesotibia; apical half of mesodistitarsus; apicodorsal rim of metafemur; wide basal ring on metatibia. Metasoma mostly orange-brown, T1-T5 marginal zones translucent orange to translucent yellow at margins; T1 pregradular area black, T1-T2 lateral recurved portion of gradulus black, T1-T3 with lateral dark spots on premarginal line, T4-T6 black; sterna orange-brown, suffused with dark brown on disc and premarginal line.

*Pubescence*: As in male except as follows: clypeal hairs short and sparse (0.5OD); genal beard sparse (0.5–1.5OD); discs of mesoscutum, scutellum, and metanotum with sparse short hairs (≤0.5OD), longer and denser toward lateral margin as follows: mesoscutum (0.5OD), scutellum (1–1.5OD) also on posterior margin, longest posteromedially, metanotum (≤1.5OD); propodeal hairs moderately long and dense dorsolaterally (0.5–1OD); scopae on metafemur and metatibia (1–1.5OD); T1-T2 apicolateral patches of tomentum (0.5–1OD), T3 apicolateral hair band sparse, not tomentose; S1 hairs long and moderately dense (≤2.5OD); S2 scopal hairs (1–2.5OD).

*Surface sculpture*: As in male except as follows: clypeus moderately densely punctate throughout (i=1-2d); supraclypeal area irregularly punctate (i=1-2d) densely punctate toward lateral margin (i≤d); frontal area densely punctate (i≤d); ocellocular space moderately densely punctate (i=1-2d); vertexal area densely punctate (i≤d); genal area coarsely imbricate and densely punctate (i=d); mesoscutum and scutellum densely punctate (i=0.5-1d); mesepisternum irregularly punctate, moderately dense below scrobe (i=0.5-2d), sparser anterior of episternal groove (i=1-3d), irregularly spaced on hypoepimeral area (i≤2d); metanotum moderately densely punctate (i=0.5-2d); metapostnotum coarsly rugose; propodeum coarsely imbricate and moderately densely punctate (i=1-2d); T1-T5 punctures small and sparse (i≥2d; but i=1-3d on T3); T6 moderately densely punctate (i=1-2d).

*Structure*: Labrum ~2.5× wider than long (23:9); malar space approximately one third as long as clypeal lateral (2.5:8); LOT below anterior tentorial pits; length of clypeus subequal to maximum width in frontal view (34:29) extending for less than half of its length beyond LOT; median longitudinal groove weak on clypeus; subantennal sutures ~1.1 longer than shortest distance between them (12.5:11); IAD 1.5× AOD (12:8); scape 3.5× as long as maximum width (21:6); pedicel longer than wide (8:6.5); F1 slightly longer than wide (6:5.5); F2 half as long as F1 (3) and 0.75× as long as F3 (4); UOD ~1.3× LOD, IOD ~1.05× UOD (UOD:IOD:LOD - 42:44:32); frontal line carinate in lower half, flat above; MOC longer than width of head (75:69); OOC ~0.6× IOC (10:17); genal area ~0.75× as wide as eye in lateral view (17:23); ratio of lengths of mesoscutum: scutellum: metanotum: metapostnotum – 51:19:9:13.5; probasitarsus ~3.5× as long as maximum depth (19:5.5); length of prodistitarsus ~0.9× that of preceding three tarsomeres combined (from II to V - 5:4.5:4:12); metafemur 3× as long as maximum depth (39:13); metatibia ~4.33× as long as maximum depth (52:12); metabasitarsus 4× as long as maximum depth (32:8)

###### Type material

(42 males & 96 females). **Holotype** (male): Region III, N of Vallenar, S 28.4142°, W 70.7213°, 658m, 15-17.ix.2010, L. Packer, pan trap (PCYU); **Allotype** (female): Region III, E of Llanos de Challe, S 28.1500°, W 70.9610°, 189m, 14.x.2013, S. Monckton, PCYU 0020860 (PCYU); **Paratypes: Region III**: three males and four females, same data as allotype, PCYU 0020858, PCYU 0020859, PCYU 0020862, PCYU 0020863, CCDB-19989 A03 // PCYU 0021665, PCYU 0020864, CCDB-19989 A04 // PCYU 0021666 (PCYU); two females, same data as allotype (PUCV); one female, same data as allotype (AMNH); four females, Parque Nacional Llanos de Challe, *S 28.196°, W 71.044°, 230m*, 22.x.2000, L. Packer (PCYU); one male, Panamericana km 683, N of Vallenar, *S 28.395°, W 70.717°, 632m*, 17.x.2000, L. Packer (PCYU); ten females, N of Vallenar, FIT Southern, *S 28.414°, W 70.721°, 658m*, x.2000, L. Packer (PCYU); six males, N of Vallenar, *S 28.414°, W 70.721°, 658m*, 11-12.x.2000, L. Packer (PCYU); two males and one female, Huasco Prov., 10 km N of Vallenar, S 28.4861°, W 70.7314°, 610m, 28.x.2000, J.G. Rozen (AMNH); one female, 10 km N of Vallenar, *S 28.488°, W 70.733°, 579m*, 18.x.2000, L. Packer (PCYU); one female, 6 km N of Huasco, S 28.4361°, W 71.1846°, 87m, 14.x.2013, S. Monckton (PCYU); one male and two females, N of Huasco, km 11, S 28.3816°, W 71.1625°, 86m, 7.xi.2013, S. Monckton (PCYU); one female, Tres Playitas, 10 km N of Huasco Bajo, *S 28.381°, W 71.177°, 22m*, 13.x.2001, L. Packer (PCYU); one female, N of Huasco, km 18, S 28.3248°, W 71.1528°, 62m, 7.xi.2013, S. Monckton (PCYU); one male, Canto Agua, *S 28.15°, W 70.933°, 247m*, 9.x.1977, H. Toro (PUCV); one male, same locality and date, J. Magunacelaya (PUCV); two males and twelve females, N of Huasco, km 39, S 28.1472°, W 71.1562°, 55m, 7.xi.2013, S. Monckton (PCYU); two females, same data, CCDB-19989 A06 // PCYU 0021668, PCYU 0021594 (PCYU); one female, same data (AMNH); one female, N of Huasco, km 43, S 28.1131°, W 71.1542°, 55m, 7.xi.2013, S. Monckton (PCYU); two males and six females, N of Huasco, km 52, S 28.0438°, W 71.1288°, 65m, 7.xi.2013, S. Monckton (PCYU); three males and three females, N of Huasco, km 67.2, S 27.9272°, W 71.0851°, 169m, 7.xi.2013, S. Monckton (PCYU); five males and twelve females, N of Huasco, km 72, S 27.8896°, W 71.0824°, 216m, 7.xi.2013, S. Monckton (PCYU); one male and one female, same data (BBSL); one male and one female, same data (CTMI); one female, Caleta Totoral, S 27.8316°, W 71.0841°, 14m, 7.xi.2013, S. Monckton (PCYU); three males and twelve females, C-451, 2.3 km E of Ruta 5, S 28.0188°, W 70.5538°, 488m, 22-25.x.2010, L. Packer, pan traps (PCYU); one male, Castilla, *S 27.903°, W 70.679°, 278m*, 18.ix.1986, L. Ruz (PUCV); one female, Ruta 5, 768.8km, S 27.6728°, W 70.4746°, 653m, 1.x.2013, S. Monckton, CCDB-22788 F01 (PCYU); one male and two females, Ruta 5, Km 773, S 27.6421°, W 70.4634°, 600m, 25.x.1995, L. Ruz (PUCV); one female, Rd to Nantoco km 3, S 27.6161°, W 70.4279°, 678m, 6.xi.2013, S. Monckton, CCDB-19989 A05 // PCYU 0021667 (PCYU); one male, Travesía, *S 27.54°, W 70.443°, 713m*, 9.x.1972, H. Toro (AMNH); one male, same locality, 29.ix.1983, O. Martinez (PUCV); one female, Quebrada del Potrero, S 26.8569°, W 70.6639°, 348m, 14.x.2010, L. Packer & E. Almeida, PCYU 0004790 (PCYU); five females, Embalse Santa Juana, C-479, km 21.5, S 28.6747°, W 70.6432°, 645m, 15.x.2013, S. Monckton, CCDB-19989 A07 // PCYU 0021669, PCYU 0020912, PCYU 0020911, PCYU 0020913, PCYU 0020914 (PCYU); one female, >2km W of Domeyko, S 28.9648°, W 70.9158°, 764m, 15.x.2013, S. Monckton (PCYU); one male, W of Domeyko, S 28.9772°, W 71.0006°, 610m, 5.x.2010, L. Packer & G.S. Fraser, PCYU 0004777 (PCYU); one female, Cta. Chañaral de Aceituno, *S 29.079°, W 71.488°, 12m*, 11.x.1981, O. Martinez (PUCV); one female, same locality and date, J. Magunacelaya (PUCV); four males and two females, S of Cachiyuyo, S 29.1475°, W 70.9419°, 1247m, 9.x.2010, L. Packer & G.S. Fraser, PCYU 0004713, PCYU 0004711, PCYU 0004708, PCYU 0004712, PCYU 0004714, PCYU 0004710 (PCYU).

###### Variation.

Some females have F1-F3 more thoroughly dark, with only the apicoventral margin brown.

###### Distribution.

Intermediate Desert and one record from Coquimban Desert, distributed coastally from Quebrada del Potrero (Region III) south to P.N. Fray Jorge (Region IV) and inland east to Travesia (Region III); 12–1613m a.s.l.

###### Ecology.

Recorded September to November.

###### Etymology.

The specific epithet honours Laurence Packer, the author’s mentor and supervisor, and collector of the holotype specimen. It is formed in the genitive singular case.

###### Comments.

Specimens of *Chilicola packeri* had previously all been identified as *Chilicola neffi* due to the longitudinal depressions on the frontal area present in both species. The former was recognized following the observation that it has a distinct, non-overlapping geographic distribution relative to *Chilicola neffi*, as well as a unique DNA barcode sequence, following which morphological differences were also observed.

##### Chilicola (Heteroediscelis) randolphi

Taxon classificationAnimaliaHymenopteraColletidae

Monckton sp. n.

http://zoobank.org/9CBF62E8-8C53-4139-A85F-F0A248D49E71

**Figure 45.**
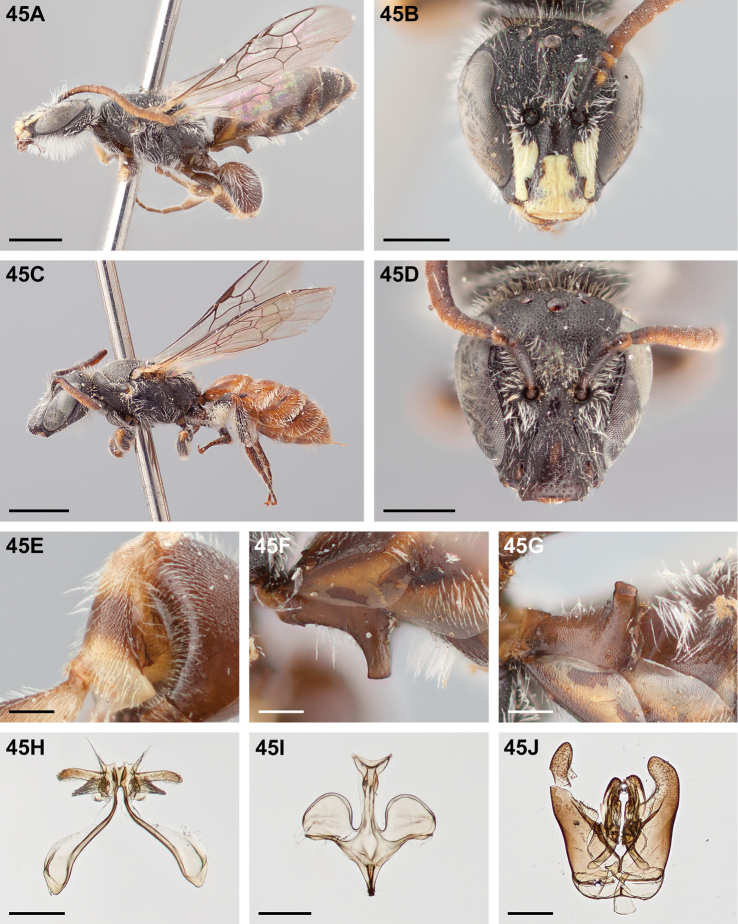
*Chilicola randolphi*: **A** male habitus, lateral view, scale bar 1 mm **B** male head, frontal view, scale bar 0.5 mm **C** female habitus, lateral view, scale bar 1 mm **D** female head, frontal view, scale bar 0.5 mm **E** male metatibia, posterior view, scale bar 0.25 mm **F** male S1 process, lateral view^†^, scale bar 0.25 mm **G** male S1 process, apical view, scale bar 0.25 mm **H** male S7 (C.rndi.003), scale bar 0.25 mm **I** male S8 (C.rndi.003), scale bar 0.25 mm **J** male genital capsule (C.rndi.003), scale bar 0.25 mm.

Figs 5B ; 11D ; 15A–C ; 17A ; 18A–C ; 45A–J ; 49

###### Diagnosis.

Males are readily diagnosed by the distinctly truncate S1 process with apex perpendicular to axis of process in lateral view, and metabasitarsus expanded basally (~0.33× as deep as long). Among consubgeners, only *Chilicola erithropoda* has the metabasitarsus expanded thus, but is readily distinguished from *Chilicola randolphi* by the entirely yellow-orange tibiae and S1 process in lateral view with anterior and posterior margins divergent apically. Females are diagnosable by the red-brown metasoma and protibia yellow only on basal half of dorsal surface. Females of *Chilicola packeri*, many females of *Chilicola curvapeligrosa*, and some females of *Chilicola neffi* also have the metasoma red-brown, but always have the protibia yellow on the entire dorsal surface.

###### Description.

**Male (Holotype).** Length 5.6mm, forewing length 3.6mm, head width 1.3mm, thorax width 1.4mm, median ocellar diameter (OD) 0.13mm.

*Colouration*: Black-brown, following parts yellow: labrum; clypeus except broadly dark along epistomal suture and apex brown laterad; lower paraocular area, extending dorsad to just below lower tangent of antennal socket laterally and about 1OD above transverse portion of epistomal suture medially; apicoventral spot on scape; protrochanter apical rim posteriorly; apex of profemur broadly on anterior surface, narrowly on posterior surface; protibia except dark medioventrally; basal half of dorsal surface of probasitarsus; wide apical ring on mesofemur; basal and apical quarters of mesotibia, dorsal surface suffused with yellow throughout; base of mesobasitarsus; apicodorsal rim of metafemur; ventral surface of metatibia, as well as dorsal surface in basal one-third and narrowly yellow apically except broad around apical lamina and posterior concavity; basal half of metabasitarsus ventrally; anterior spot on tegula; apex of T7. Following parts yellow-brown: ventral surface of antenna from apical half of pedicel to terminal flagellomere, narrowing basad on pedicel and F1; apical half of prodistitarsus; apicoventral rim of metacoxa and metatrochanter. Ventral and posterior metatibial carinae black. Metasoma dark brown, T1-T6 marginal zones amber to translucent yellow at margins.

*Pubescence*: White, hairs generally short (0.5OD) and sparse, not especially plumose; on lower paraocular area and around antennal base long and moderately dense (1.5OD); genal beard long and dense (0.5–3OD) longest at midlength; mesoscutal hairs moderately long and sparse, denser toward lateral margin (1–1.5OD); discs of scutellum and metanotum mostly bare, few short hairs (0.5OD), longer and denser toward lateral and posterior margin of scutellum (1–2OD) longest posteromedially, and toward lateral margin of metanotum (≤2.5OD); propodeal hairs moderately long and dense dorsolaterally (0.5–2.5OD); T1-T3 apicolateral patches of tomentum (0.5–1OD); S2 hairs long and dense (2OD basolaterad, 1.5OD distilaterad, shorter mesad).

*Surface sculpture*: Microsculpture imbricate, integument generally dull; punctures generally small and deep. Clypeus and lower paraocular area moderately densely punctate (i=1-2d) except clypeus sparsely punctate medially (i=2-3d); supraclypeal area irregularly punctate (i=1-4d) densely punctate toward lateral margin (i≤d); frontal area very densely punctate (i≤0.5d), densely punctate around ocelli (i≤d); ocellocular space moderately densely punctate adjacent to compound eye (i=1-2d) becoming impunctate adjacent to lateral ocellus; vertexal area rugose and densely punctate (i≤d); scape shallowly and moderately densely punctate (i=1-2d) densely punctate apicoventrally (i≤d); genal area microstriate and moderately densely punctate (i≤2d); hypostomal area densely punctate (i=d); pronotum densely punctate (i=0.5-1d), lateral surface coarsely imbricate; mesoscutum densely punctate (i=0.5-1d) more sparsely punctate toward lateral margin (i=0.5-2d) punctures crowded around median (i≤d); scutellum moderately densely punctate (i=1-2d) punctures crowded around median (i≤d); mesepisternum irregularly punctate, sparse below scrobe (i=1-3d), sparser anterior of episternal groove (i≥2d), denser on hypoepimeral area (i≤2d); metanotum moderately densely punctate (i≤2d) punctures crowded around median and anteriorly (i≤0.5d); metepisternum densely punctate (i≤d) and distinctly longitudinally striate anterodorsally; metapostnotum rugose; propodeum coarsely imbricate and moderately densely punctate (i=1-2d); T1 punctures small and moderately dense (i=1-3d); T2-T5 moderately densely punctate (i=1-3d); marginal zones of terga weakly imbricate and shiny, minutely punctate to impunctate.

*Structure*: Labrum ~2.2× wider than long (25:11.5); malar space slightly shorter than clypeal lateral (7:8); LOT at level of anterior tentorial pits; length of clypeus subequal to maximum width in frontal view (30:31) extending for less than half its length beyond LOT and convex above anterior tentorial pit, in lateral view bulging ~1OD above compound eye; median longitudinal groove present on clypeus except on apex; subantennal sutures shorter than the shortest distance between them (15:16); IAD nearly double AOD (15: 8); scape ~2.3× as long as maximum width (25:11); pedicel slightly shorter than wide (9:9.5); F1 as long as wide (9.5:9.5); F2 ~1.5× as long as F1 and same length as F3 (F2:F3 - 14.5:14.5); UOD ~1.3× LOD, IOD ~1.1× UOD (UOD:IOD:LOD - 55:60:42); frontal line carinate in lower half, flat above, interrupted at midlength; MOC shorter than width of head (82:93); OOC ~0.8× IOC (16:19); genal area 0.6× as wide as eye in lateral view (18:30); ratio of lengths of mesoscutum: scutellum: metanotum: metapostnotum – 65:25:11:20; probasitarsus ~2.6× as long as maximum depth (21:8); length of prodistitarsus subequal to that of preceding three tarsomeres combined (from II to V- 6:4:5:16); metafemur 1.85× as long as maximum depth (74:40); summit of metatibial ventral convexity near tibial apex; metatibia less than twice as long as maximum depth (53:32); in apical view apical lamina of metatibia broadly expanded with distinct, secondary anterior lobe, length ~1.33× OD (12:9); ventral metatibial carina sharply toothed near apex, posterior carina sublinear and weakly defined basally; metabasitarsus very deep in basal half, only ~3× as long as maximum depth (46:15); S1 process apical surface longitudinally concave, in lateral view anterior and posterior margins subparallel apically. S7 apodemal arm sclerotized margin terminating laterally at arm midlength; ventral lobe ~0.5× as broad as basal attached length; dorsal lobe strap-like, subparallel-sided and somewhat anteriorly curved, with basal tuft of long setae and a short apical row, midlength bare; apex of disc sinuate. S8 lateral process ~1.1× wider than long, anteromedial sclerotized margin following marginal contour. Gonobase apicoventral truncate process biconvex and distinctly notched by a width less than one convexity.

**Female (Allotype).** Length 5.6mm, forewing length 3.6mm, head width 1.1mm, thorax width 1.3mm, median ocellar diameter (OD) 0.10mm.

*Colouration*: Black to black-brown except as follows: labrum yellow-brown; mandible with yellow basal spot, translucent yellow in apical half except apex translucent brown. Following parts brown: spot below anterior tentorial pit; median spot on clypeus just above upper margin of anterior tentorial pits; basoventral spot on scape. Following parts yellow: dorsal surface of protibia in basal half; apical two-thirds of prodistitarsus; narrow basal ring on mesotibia; faint anterior spot on tegula. Following parts yellow-brown: apicoventral surfaces of pedicel and F1; ventral surface of antenna from F2 to terminal flagellomere, flagellomeres suffused with brown basally; apical ring on protibia; apical half of mesodistitarsus; apical ring on metafemur; basal ring on metatibia. Metasoma orange-brown, graduli of T2-T4 & S2-S4 dark and widened medially to basal half of disc on S2.

*Pubescence*: As in male except as follows: on lower paraocular area and around antennal base moderately long and moderately dense (1OD); genal beard moderately dense (0.5–2.5OD); discs of mesoscutum, scutellum, and metanotum with sparse short hairs (≤0.5OD), denser and as long or longer toward lateral margin as follows: mesoscutum (0.5OD), scutellum (1–2OD) also on posterior margin, longest posteromedially, metanotum (2OD); scopar on metafemur and metibia (1–1.5OD); T1-T3 apicolateral patches of tomentum (0.5–1OD); S1 hairs long and moderately dense (≤2OD); S2 scopal hairs (1–2OD).

*Surface sculpture*: As in male except as follows: lower paraocular area moderately densely punctate (i=1-2d) densely punctate adjacent to epistomal suture (i<d); frontal area densely punctate (i≤d), moderately densely punctate around ocelli (i=1-2d); vertexal area densely punctate (i≤d); scape shallowly and moderately densely punctate (i=1-2d); genal area weakly imbricate, weakly microstriate posteriorly, and moderately densely punctate throughout (i=1-2d); hypostomal area moderately densely punctate (i≤2d); pronotum densely punctate (i=d); mesoscutum densely punctate (i=d) moderately densely punctate toward lateral margin (i=1-2d); scutellum and metanotum moderately densely punctate (i=0.5-2d) except metanotal punctures crowded around median and anteriorly (i≤0.5d); metapostnotum rugose; T1-T5 punctures small and sparse (i=1-3d); T6 moderately densely punctate (i=1-2d).

*Structure*: Labrum ~2.3× wider than long (26:11.5); malar space ~0.8× as long as clypeal lateral (7:9); LOT at level of anterior tentorial pits; length of clypeus subequal to maximum width in frontal view (34:33) extending for less than half its length beyond LOT and convex above anterior tentorial pit as in male; median longitudinal groove absent from clypeus; subantennal sutures shorter than the shortest distance between them (12.5:14.5); IAD 1.6× AOD (16:10); scape ~3.3× as long as maximum width (26:8); pedicel ~1.3× as long as wide (9:7); F1 slightly longer than wide (8:7.5); F2 ~0.6× as long as F1 (4.5) and 0.75× as long as F3 (6); UOD ~1.3× LOD, IOD ~1.1× UOD (UOD:IOD:LOD - 55:60:42); frontal line carinate in lower half, flat above; MOC shorter than width of head (85:90); OOC ~0.7× IOC (13.5:20); genal area about two thirds as wide as eye in lateral view (18:28); ratio of lengths of mesoscutum: scutellum: metanotum: metapostnotum – 61:25:11.5:16; probasitarsus ~3.5× as long as maximum depth (25:8.5); prodistitarsus ~0.9× as long as preceding three tarsomeres combined (from II to V - 6:4:5:14); metafemur ~3.3× as long as maximum depth (53:16); metatibia ~4.7× as long as maximum depth (66:14); metabasitarsus almost 4× as long as maximum depth (43:11).

###### Type material

(6 males & 15 females): **Holotype** (male): Region VII, Laguna del Maule, S 36.0027°, W 70.4555°, 2227m, 4.i.2009, L. Packer, on *Phacelia*, CCDB-03755 D08, CHI09-6-5-004 (PCYU); **Allotype** (female): same data as holotype, CHI09-6-5-007 (PCYU); **Paratypes: Region VII**: one male and four females, same data as holotype, CHI09-6-5-008, CHI09-6-5-001, CHI09-6-5-002, CHI09-6-5-005, CHI09-6-6-001, (PCYU); two females, same data as holotype (PUCV); two females, same data as holotype (CTMI); one male and one female, E of Laguna del Maule, 160.120km, S 35.9928°, W 70.3991°, 2505m, 1-6.i.2013, L. Packer & R. Smith, white pan traps, CCDB-19991 D05, CCDB-19991 D02 (PCYU); one female, same locality, 1-20.iii.2013, S. Monckton & J. Postlethwaite, pan trap (PCYU); one female, W of Laguna del Maule, S 35.9901°, W 70.3977°, 2541m, 5.i.2009, L. Packer, on *Phacelia*, CCDB-03755 E07 // CHI09-6-5-056 (PCYU); one female, Río Maule, Hwy 115, km 122, S 35.9016°, W 70.6426°, 1359m, 1-20.iii.2013, S. Monckton & J. Postlethwaite, pan trap (PCYU); **Region VIII**: two males, Ñuble, Los Lleuques, 4km. E of Recinto, *S 36.854°, W 71.645°, 783m*, xii.1982, L. Peña (AMNH); one female, Ñuble, Las Trancas, Shangrila in Chillán area, SE of Recinto, *S 36.876°, W 71.468°, 1549m*, 14.xii.1983, L. Peña (AMNH); one female, Ñuble, Las Trancas, SE Recinto in Chillán area, *S 36.911°, W 71.48°, 1250m*, i.1987, Luis E. Peña (AMNH); one male, Las Cabras, mts. in Chillán area, S Chillán Volcano, Ñuble Prov., *S 36.918°, W 71.429°, 1500m*, 6–31.i.1963, L.E. Peña (AMNH).

###### Variation.

Some males have the ventral margin of the metatibia dark. Females often have the gradulus of T1 dark, and usually lack the brown spots on the clypeus and below the anterior tentorial pit.

###### Distribution.

Southern Andean Cordillera, from Laguna del Maule (Region VII) southwest to Valle Las Trancas (Region VIII); 783–2541m a.s.l.

###### Ecology.

Collected on *Phacelia* (12 records). Recorded December to January and March.

###### Etymology.

The specific epithet honours the author’s father, W.F. Randolph Monckton, who nurtured an interest in science and geography, and encouraged a desire for knowledge. It is formed in the genitive singular case.

##### Chilicola (Heteroediscelis) travesia

Taxon classificationAnimaliaHymenopteraColletidae

Toro & Moldenke, 1979

**Figure 46.**
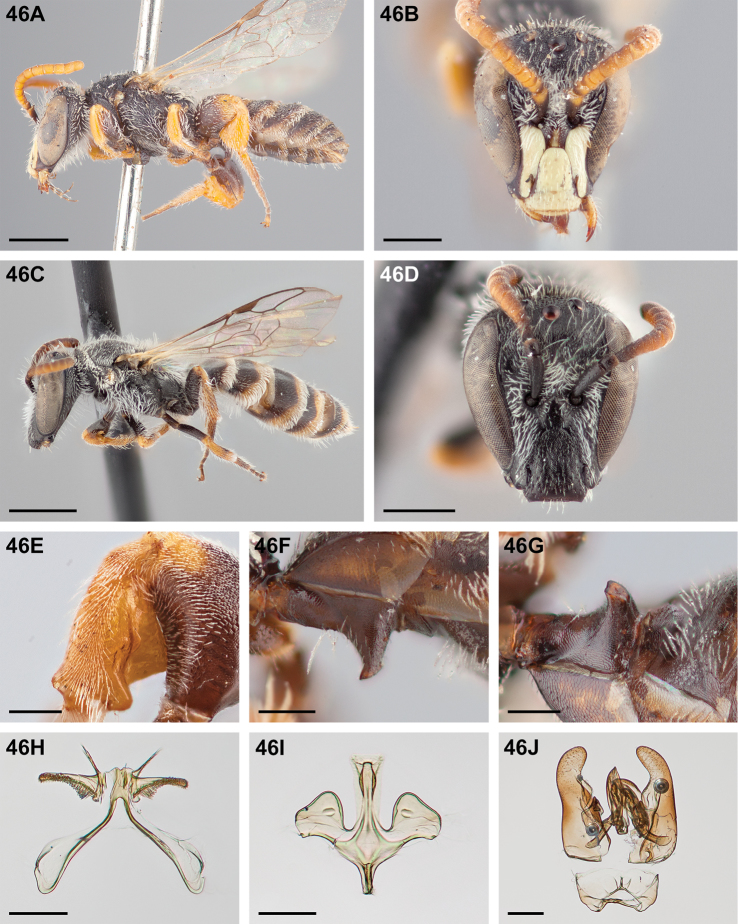
*Chilicola travesia*: **A** male habitus, lateral view, scale bar 1 mm^†^ **B** male head, frontal view, scale bar 0.5 mm **C** female habitus, lateral view, scale bar 1 mm **D** female head, frontal view, scale bar 0.5 mm **E** male metatibia, posterior view, scale bar 0.25 mm **F** male S1 process, lateral view^†^, scale bar 0.25 mm **G** male S1 process, apical view, scale bar 0.25 mm **H** male S7, scale bar 0.25 mm **I** male S8, scale bar 0.25 mm **J** male genital capsule, scale bar 0.25 mm.

Figs 1A ; 2A–B ; 29A–B ; 30A–B ; 46A–J ; 49

Chilicola travesia Toro & Moldenke, 1979: 125–127 (male holotype, female allotype, AMNH [examined]). Packer 2008: 77. Montalva and Ruz 2010: 29 (checklist). Moure et al. 2012 (catalogue). Ascher and Pickering 2015 (checklist).

###### Diagnosis.

Males are separated - along with *Chilicola erithropoda* - by the tibiae and tarsi mostly or entirely yellow-orange, and differentiated from the latter by MOC equal to or greater than width of the head, and apical surface of S1 process longitudinally concave. Females are separated from all other species of the subgenus except *Chilicola erithropoda* by the combination of malar space less than 2/3× as long as the clypeal lateral, metatibia expanded (>0.25× as deep as long), and T3 with apicolateral patches of tomentum as on T1-T2. Females of *Chilicola travesia* are differentiated from those of *Chilicola erithropoda* by the metatibia mostly or entirely yellow (basal one-third and apical quarter at minimum) and the probasitarsus entirely yellow (sometimes suffused with yellow-brown at midlength on posterior surface) – both are mostly dark in the latter species.

###### Description.

**Male.** Length 5.3–5.8mm, forewing length 3.2–3.5mm, head width 1.2–1.3mm, thorax width 1.3mm, median ocellar diameter (OD) 0.13mm.

*Colouration*: Black-brown, following parts yellow: labrum; clypeus except narrowly dark at mid-length along epistomal suture and apex often translucent brown laterad; lower paraocular area, extending more than 1OD above transverse portion of epistomal suture medially and reaching lower tangent of antennal socket laterally; apicoventral surface on scape; ventral surface of pedicel, narrowing basally; ventral surface of flagellum; protrochanter apical rim posteriorly; apex of profemur broadly on anterior surface, narrowly on posterior surface; protibia; protarsus; broad apical ring on mesofemur; mesotibia, sometimes suffused with brown medioventrally; mesotarsus; apicoventral rim of metacoxa and metatrochanter; broad apical ring on metafemur; metatibia, sometimes suffused with brown mediodorsally; metabasitarsus, at least ventrally; apical half of metadistitarsus; anterior spot on tegula; apex of T7. Ventral and posterior metatibial carinae brown. Metasoma dark brown, T1-T6 marginal zones yellow-orange to translucent yellow at margins.

*Pubescence*: White, hairs generally short (0.5OD) and sparse, not especially plumose; on lower paraocular area and around antennal base moderately long and moderately dense (1OD); genal beard long and moderately dense (0.5–2OD) longest at midlength; mesoscutal hairs moderately long and sparse (1OD) denser toward lateral margin (0.5–1OD); discs of scutellum and metanotum mostly bare, few short hairs (0.5 OD), longer and denser toward lateral and posterior margins of scutellum (1–1.5OD) longest posteromedially, and toward lateral margin of metanotum (≤2OD); propodeal hairs moderately long and dense dorsolaterally (0.5–1.5OD); T1-T3 apicolateral patches of tomentum (0.5–1OD); S2 hairs moderately long and moderately sparse (1OD basolaterad, 0.5OD distilaterad, bare mesad).

*Surface sculpture*: Microsculpture imbricate, integument generally dull; punctures generally small and deep. Clypeus sparsely and irregularly puncture (i=1-3d); supraclypeal area moderately densely punctate (i=1-2d) densely punctate toward lateral margin (i≤d); lower paraocular area densely punctate (i=d); frontal area very densely punctate (i≤0.5d); ocellocular space moderately densely punctate adjacent to compound eye (i=1-2d) becoming impunctate adjacent to lateral ocellus; vertexal area moderately densely punctate (i=1-2d); scape shallowly and moderately densely punctate (i=1-2d) densely punctate apicoventrally (i≤d); genal area microstriate and moderately densely punctate (i=1-2d); hypostomal area weakly imbricate and densely punctate (i=d); pronotum and mesoscutum densely punctate (i=0.5-1d); scutellum moderately densely punctate (i=1-2d) punctures crowded around median (i≤d); mesepisternum irregularly punctate, sparse below scrobe and anterior of episternal groove (i=1-2d), impunctate just dorsad of scrobe, otherwise irregularly spaced on hypoepimeral area (i≤2d); metanotum densely punctate (i=0.5-2d) punctures crowded around median and anteriorly (i≤0.5d); metepisternum densely punctate (i≤d); metapostnotum rugose, minutely rugose posteriorly; propodeum coarsely imbricate and moderately densely punctate (i=1-2d); T1-T5 coarsely imbricate; T1-T4 moderately densely punctate (i≤2d); T5 moderately punctate-puncticulate (i=1-3d); T6 sparsely punctate-puncticulate (i≥2d) and coarsely imbricate posteriorly; T7 sparsely punctate-puncticulate (i≥2d); marginal zones of terga weakly imbricate and shiny, minutely punctate.

*Structure*: Labrum ~2.8× wider than long (24:8.5); malar space ~0.6× as long as clypeal lateral (4:7); LOT below anterior tentorial pits; clypeus ~1.1× as long as maximum width in frontal view (35:31) extending for less than half of its length beyond LOT; clypeus median longitudinal groove absent or marked by a very weak depression; subantennal sutures ~1.3× as long as the shortest distance between them (14:11); IAD ~1.6× AOD (13:8); scape 2.4× as long as maximum width (24:10); pedicel slightly shorter than wide (8:8.5); F1 shorter than wide (7:9); F2 slightly longer than F1 and shorter than F3 (F2:F3 - 7.5:10); UOD ~1.4× LOD, IOD ~1.1× UOD (UOD:IOD:LOD - 50:54:36); frontal line carinate in lower half, flat just below median ocellus for a length of ~0.5OD, otherwise roughly defined; MOC subequal to width of head (89:87); OOC ~0.5× IOC (9:20); genal area ~0.6× as wide as compound eye in lateral view (18:33); ratio of lengths of mesoscutum: scutellum: metanotum: metapostnotum - 61:25:11:14; probasitarsus 4× as long as maximum depth (24:6); length of prodistitarsus ~0.7× that of preceding three tarsomeres combined (from II to V - 7:6:5:13); metafemur ~1.67× as long as maximum depth (60:36); summit of metatibial ventral convexity at approximately two-thirds tibial length; metatibia ~1.67× as long as maximum depth (55:33); in apical view length apical lamina of metatibia short and thick, length 0.8× OD (8:10); ventral metatibial carina bluntly toothed near midpoint and deeply concave apically, posterior carina absent basally and originating at level of tooth on ventral carina; metabasitarsus ~3.7× as long as maximum depth (41:11); S1 process apical surface longitudinally concave, in lateral view anterior and posterior margins divergent apically and apex appearing strongly anteriorly bent. S7 apodemal arm sclerotized margin terminating laterally at arm midlength; ventral lobe ~0.25× as broad as basal attached length; dorsal lobe strap-like, subparallel-sided and somewhat anteriorly curved, with row of setae long basally and shorter apically; apex of disc disinctly emarginate. S8 lateral process ~1.1× wider than long, apicolateral margin distinctly concave, anteromedial margin variable, having either a distinct convex incursion and appearing swollen, or a sclerotized spot disjunct from margin (as in Fig. 38). Gonobase apicoventral truncate process biconvex and distinctly notched by a width less than one convexity.

**Female.** Length 4.9–5.5mm, forewing length 3.1–3.2mm, head width 1.1–1.2mm, thorax width 1.1–1.3mm, median ocellar diameter (OD) 0.11–0.12mm.

*Colouration*: Black to black-brown except as follows: labrum black to translucent yellow; mandible with yellow basal spot, otherwise translucent yellow with apex translucent brown; antenna variable (scape with yellow-brown apicoventral spot to entirely dark; apicoventral surface of pedicel yellow to brown; ventral surface of flagellum yellow, either narrowing basally on F1, or with apicoventral surface of F1 brown and narrowing basally on F2). Following parts yellow: protibia, sometimes dark medioventrally; protarsus; mesotibia, at least in basal quarter ventrally, basal one-third dorsally, and apical quarter; mesotarsus, at least basitarsus ventrally and apical half of distitarsus; broad apical ring on metafemur; metatibia, at least in basal one-third and apical quarter; metabasitarsus ventrally; apical half of metadistitarsus; anterior spot on tegula. Metasoma black, T1-T5 marginal zones yellow to translucent yellow at margins.

*Pubescence*: As in male except as follows: clypeal hairs short and sparse (0.5OD); genal beard sparse (0.5–1.5OD); discs of mesoscutum, scutellum, and metanotum with sparse short hairs (≤0.5OD) denser and as long or longer toward lateral margin as follows: mesoscutum (≤0.5OD), scutellum (1–1.5OD) also on posterior margin, longest posteromedially, metanotum (≤1.5OD); propodeal hairs moderately long and dense dorsolaterally (0.5–1.5OD); scopae on metafemur and metatibia (1–2OD); T1-T3 apicolateral patches of tomentum (0.5–1OD); S1 hairs long and moderately dense (≤2.5OD); S2 scopal hairs (1–3OD).

*Surface sculpture*: As in male except as follows: supraclypeal area irregularly punctate (i=1-3d) densely punctate toward lateral margin (i≤d); frontal area densely punctate (i≤d), moderately densely punctate around ocelli (i=1-2d); vertexal area moderately densely punctate (i=1-2d); scape shallowly and moderately densely punctate (i=1-2d); genal area coarsely imbricate and moderately densely punctate (i=1-2d); hypostomal area weakly imbricate, shallowly and sparsely punctate (i≥2d); mesoscutum and scutellum densely punctate (i=0.5-1d); mesepisternum irregularly punctate, sparse below scrobe and anterior of episternal groove (i=1-3d), dense on hypoepimeral area (i≤d); metanotum moderately densely punctate (i=0.5-2d); metapostnotum rugose, minutely rugose posteriorly; propodeum moderately densely punctate (i=1-2d); T1 punctures small and sparse (i≥2d basally; i=1-3d apically); T2-T5 punctures small and sparse (i=1-3d on T2-T3; i≥2d on T4-T5); T6 moderately densely punctate (i=1-2d).

*Structure*: Labrum 2.4× wider than long (24:10); malar space two-thirds as long as clypeal lateral (6:9); LOT below anterior tentorial pits; clypeus ~1.2× as long as maximum width in frontal view (35:29) extending for less than half of its length beyond LOT; median longitudinal groove weak on clypeus; subantennal sutures shorter than the shortest distance between them (11.5:12.5); IAD 1.5× AOD (13.5:9); scape ~3.3× as long as maximum width (23:7); pedicel longer than wide (8.5:7); F1 shorter than wide (5.5:7); F2 shorter than F1 and shorter than F3 (F2:F3 - 4:5); UOD ~1.3× LOD, IOD ~1.1× as long as UOD (UOD:IOD:LOD - 48:51:38); frontal line carinate in lower half, flat above; MOC longer than width of head (83:79); OOC ~0.5× IOC (9.5:20.5); genal area ~0.6× as wide as eye in lateral view (16:28); ratio of lengths of mesoscutum: scutellum: metanotum: metapostnotum – 56:22:11:12; probasitarsus 3.5× as long as maximum depth (21:6); length of prodistitarsus ~0.7× that of preceding three tarsomeres combined (from II to V - 7:6:5:12.5); metafemur ~3× as long as maximum depth (46:15); metatibia 3.6× as long as maximum depth (54:15); metabasitarsus 4× as long as maximum depth (36:9).

###### Material studied

(29 males & 65 females): **Holotype** (male): Region III, Travesía, *S 27.540°, W 70.443°, 713m*, x.1972, Toro (AMNH); **Allotype** (female): same data as holotype (AMNH); **Region III**: one paratype female, same data as holotype (AMNH); two paratype females, same data as holotype, on *Heliotropium* (AMNH); three paratype males and two paratype females, same locality as holotype, x.1969, H. Toro (AMNH); one paratype male, same locality as holotype, 23.x.1969, H. Toro, PUCV-ENTO 19915 (PUCV); one paratype female, Via Panam N of Caldera, *S 27.058°, W 70.803°, 17m*, 2.x.1972, J.L. Neff, on *Cristaria* (AMNH); one male, same locality as holotype, ix.1986, De la Hoz (PUCV); one female, same locality as holotype, ix.1986, De la Hoz Jr. (PUCV); one male and one female, Hwy 5, km 848, S 27.3448°, W 70.6931°, 193m, 16.x.2009, J. Gibbs (PCYU); one male, same data, Bee-BOL BOWGF1087-10 B04743D06-CHL (PCYU); one male, 1 km N of Caldera, S 27.0575°, W 70.8028°, 17m, 18.x.2009, J. Gibbs, Bee-BOL BOWGF1094-10 B04743E01-CHL (PCYU); three females, Pesquera Bahía Caldera, S 27.0541°, W 70.8043°, 15m, 6.xi.2013, S. Monckton (PCYU); one male and one female, Pesquera Bahía Caldera, S 27.0541°, W 70.8043°, 0m, 6-15.xi.2013, S. Monckton & J. Postlethwaite (CTMI); one female, S of Chañaral, Ruta 5, km 930, S 26.6650°, W 70.7172°, 21m, 7.iv.2013, S. Monckton (PCYU); two males and two females, Balneario Flamenco, S of Chanaral, *S 26.573°, W 70.682°, 15m*, 22.iii.2000, L. Packer (PCYU); one male and one female, Rd to Nantoco km 0, S 27.6043°, W 70.4495°, 580m, 1.x.2013, S. Monckton, CCDB-19989 B05 // PCYU 0021679, CCDB-19989 B04 // PCYU 0021678 (PCYU); one male, Ruta 5, Km 773, S 27.6421°, W 70.4634°, 600m, 25.x.1995, L. Ruz (PUCV); three males, same locality and date, S. Rodriguez (BBSL); one male and one female, Atacama Prov., 50-60km S Copiapó, *S 27.798°, W 70.501°, 500m*, 24.viii.1966, M.E. Irwin & E.I. Schlinger (BBSL); two females, C-451, 2.3 km E of Ruta 5, S 28.0188°, W 70.5538°, 488m, 22-25.x.2010, L. Packer, pan traps (PCYU); one female, Cta Totoral, S 27.8316°, W 71.0841°, 14m, 7.xi.2013, S. Monckton, pan trap, CCDB-19989 C02 // PCYU 0021688 (PCYU); one male, Castilla, *S 27.856°, W 70.597°, 327m*, 18.ix.1986, L. Ruz (PUCV); one male, same locality, ix.1986, H. Toro (AMNH); one male, N of Huasco km 72, S 27.8896°, W 71.0824°, 216m, 7.xi.2013, S. Monckton, pan trap, CCDB-19989 B07 // PCYU 0021681 (PCYU); one female, N of Huasco, km 67.2, S 27.9272°, W 71.0851°, 169m, 7.xi.2013, S. Monckton, pan trap, CCDB-19989 C01 // PCYU 0021687 (PCYU); one female, N of Huasco, km 43, S 28.1131°, W 71.1542°, 55m, 7.xi.2013, S. Monckton (PCYU); one male and two females, Parque Nacional Llanos de Challe, *S 28.196°, W 71.044°, 230m*, 13.x.2000, L. Packer (PCYU); one female, N of Huasco, km 18, S 28.3248°, W 71.1442°, 37m, 7.xi.2013, S. Monckton (PCYU); one male and one female, Tres Playitas, 10 km N of Huasco Bajo, *S 28.397°, W 71.185°, 32m*, 13.x.2001, L. Packer (PCYU); four males, 4.4km N of Huasco Bajo, *S 28.43°, W 71.192°, 16m*, 14.ix.2010, L. Packer (PCYU); one male and fifteen females, 6 km N of Huasco, S 28.4361°, W 71.1846°, 87m, 14.x.2013, S. Monckton (PCYU); eighteen females, N of Huasco, S 28.4402°, W 71.1880°, 26m, 14.x.2013, S. Monckton (PCYU); three females, same data, pan traps, CCDB-19989 C03 // PCYU 0021689, CCDB-19989 D04 // PCYU 0021697, CCDB-19989 D03 // PCYU 0021696 (PCYU); one female, same locality and collector, 7.xi.2013, pan trap, CCDB-19989 C04 // PCYU 0021690 (PCYU); one male, 2.4 km N of Huasco Bajo, S 28.4554°, W 71.1848°, 112m, 19.x.2009, J. Gibbs, PCYU-JG09-199 (PCYU); one female, 2.4 km N of Huasco Bajo, S 28.4554°, W 71.1849°, 19m, 19.x.2009, J. Gibbs, Bee-BOL BOWGF1091-10 B04743D10-CHL (PCYU).

###### Distribution.

Intermediate Desert, distributed coastally from Balneario Flamenco south to Huasco Bajo and inland to Travesía (Region III); 0–713m a.s.l.

###### Ecology.

Collected on *Cristaria* (1 record) and *Heliotropium* L. (2 records). Recorded August to November and March to April.

##### Chilicola (Heteroediscelis) vicugna

Taxon classificationAnimaliaHymenopteraColletidae

Toro & Moldenke, 1979

**Figure 47.**
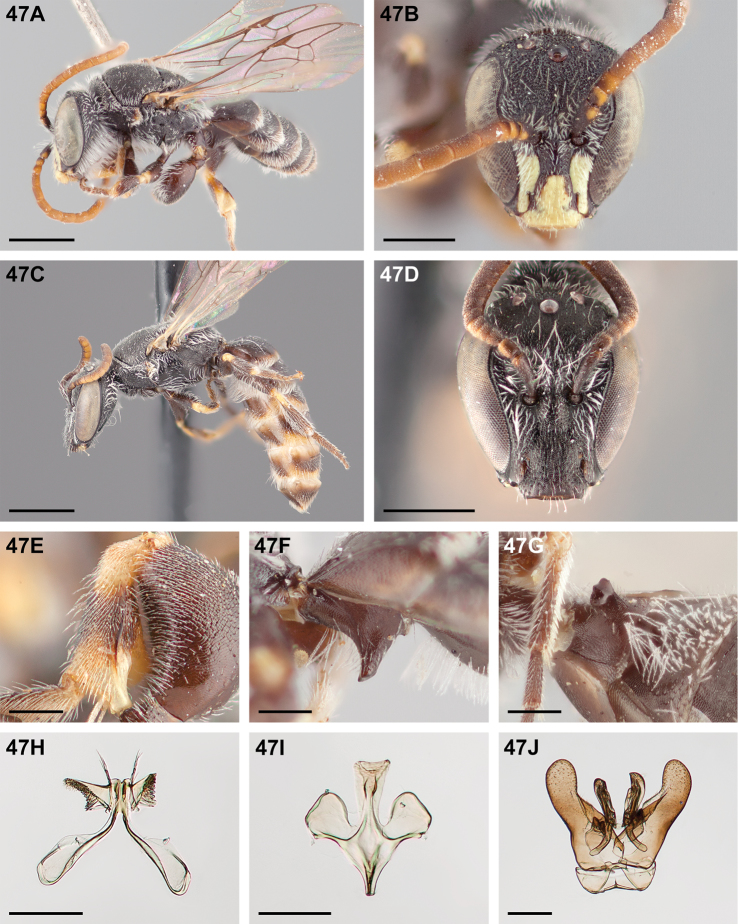
*Chilicola vicugna*: **A** male habitus, lateral view, scale bar 1 mm **B** male head, frontal view, scale bar 0.5 mm **C** female habitus, lateral view, scale bar 1 mm **D** female head, frontal view, scale bar 0.5 mm **E** male metatibia, posterior view, scale bar 0.25 mm **F** male S1 process, lateral view, scale bar 0.25 mm **G** male S1 process, apical view, scale bar 0.25 mm **H** male S7, scale bar 0.25 mm **I** male S8, scale bar 0.25 mm **J** male genital capsule, scale bar 0.25 mm.

Figs 3B ; 6D ; 8A ; 27A–B ; 28C–D ; 47A–J ; 49

Chilicola vicugna Toro & Moldenke, 1979: 120–121 (male holotype, female allotype, AMNH [examined]). Packer 2008: 77. Almeida et al. 2008: 76, 79 (phylogeny). Montalva and Ruz 2010: 29 (checklist). Almeida et al. 2011: 532 (phylogeny). Moure et al. 2012 (catalogue). Monckton 2014: 234 (male neotype, AMNH [examined]). Ascher and Pickering 2015 (checklist).

###### Diagnosis.

Males are diagnosable by the combination of malar space shorter than (~0.5×) clypeal lateral, S1 process apical surface with a distinct longitudinal ridge, and in lateral view having a distinctly acute anterior apical angle, sloping strongly toward sternum posteriorly. Females are diagnosable by the combination of malar space less than half as long as clypeal lateral, clypeus sparsely punctate medially (i≥2d), and absence of longitudinal depressions on the paraocular area.

###### Description.

**Male (Neotype).** Length 5.1mm, forewing length 3.1mm, head width 1.1mm, thorax width 1.1mm, median ocellar diameter (OD) 0.12mm.

*Colouration*: Black-brown, following parts yellow: labrum; clypeus except broadly dark along epistomal suture, sometimes apex brown altered; lower paraocular area, extending >1OD above transverse portion of epistomal suture and not reaching lower tangent of antennal socket; apicoventral spot on scape; apicoventral surface of pedicel; protrochanter apical rim posteriorly; apex of profemur broadly on anterior surface, narrowly on posterior surface; dorsal surface of protibia, as well anterior surface and basal and apical rings narrow ventrally; probasitarsus dorsally, suffused with brown ventrally; apex of mesofemur broadly on anterior surface, narrowly on posterior surface; narrow basal and apical ring on mesotibia, the latter widened to apical one-third anteriorly; anterior surface of metatibia in basal quarter and narrowly on apical margin to summit of ventral convexity, posterior surface in basal and apical one-third and along ventral carina; metabasitarsus ventrally, darker apically; anterior spot on tegula. Following parts yellow-brown: ventral surface of flagellum, flagellomeres suffused with brown basally; apical one-third of prodistitarsus; apicoventral rim of metatrochanter; apex of T7. Posterior metatibial carina black. Metasoma black, T1-T6 marginal zones translucent yellow.

*Pubescence*: White, hairs generally short (0.5OD) and sparse, not especially plumose; on lower paraocular area and around antennal base moderately long and moderately dense (1OD); genal beard long and dense (0.5–3OD) longest at midlength; mesoscutum, scutellum, and metanotum mostly bare, few short hairs (0.5OD), longer and denser toward lateral margin as follows: mesoscutum (0.5–1OD), scutellum (1–2OD) also on posterior margin, longest posteromedially, metanotum (≤2OD); propodeal hairs moderately long and dense dorsolaterally (0.5–1.5OD); T1-T3 apicolateral patches of tomentum (0.5–1OD); S2 hairs long and dense (1.5OD basolaterad, 0.5OD distilaterad, shorter mesad).

*Surface sculpture*: Microsculpture imbricate, integument generally dull; punctures generally small and deep. Clypeus and supraclypeal area moderately densely punctate (i=1-2d) except clypeus sparsely punctate medially (i=2-3d), supraclypeal area densely punctate toward lateral margin (i≤d); lower paraocular area densely punctate (i=d); frontal area very densely punctate (i≤0.5d), densely punctate around ocelli (i≤d); ocellocular space moderately densely punctate adjacent to compound eye (i=1-2d) becoming impunctate adjacent to lateral ocellus; vertexal area densely punctate (i≤d); scape shallowly and moderately densely punctate (i=1-2d) densely punctate apicoventrally (i≤d); genal area microstriate and densely punctate (i≤d); hypostomal area weakly imbricate and densely punctate (i=d); pronotum densely punctate (i=0.5-1d), lateral surface coarsely imbricate; mesoscutum and scutellum densely punctate (i=0.5-1d) punctures crowded around median (i≤d); mesepisternum irregularly punctate, sparse below scrobe and anterior of episternal groove (i=1-3d), impunctate just dorsad of scrobe, otherwise moderately dense on hypoepimeral area (i=1-2d); metanotum moderately densely punctate (i=0.5-2d) punctures crowded anteriorly (i≤0.5d); metepisternum densely punctate (i≤d) distinctly longitudinally striate anterodorsally; metapostnotum rugose; propodeum coarsely imbricate and moderately densely punctate (i=1-2d); T1-T5 coarsely imbricate; T1 sparsely punctate basally (i≥2d) more densely punctate apically (i≤2d); T2-T5 densely punctate (i≤d); T6 moderately densely punctate (i=1-2d) and coarsely imbricate posteriorly; T7 moderately densely punctate (i=1-2d); marginal zones of terga weakly imbricate and shiny, minutely punctate.

*Structure*: Labrum 2.5× wider than long (20:8); malar space ~0.5× as long as clypeal lateral (4:7); LOT below anterior tentorial pits; length of clypeus subequal to maximum width in frontal view (24:26) extending for approximately one third of its length beyond LOT; median longitudinal groove on clypeus weakly present in dorsal half, absent ventrad; length of subantennal sutures subequal the shortest distance between them (14:13.5); IAD ~2.2× AOD (13:6); scape 3× as long as maximum width (24:8); pedicel slightly shorter than wide (8:8.5); F1 slightly shorter than wide (8.5:9); F2 ~1.5× as long as F1 and ~0.9× length of F3 (F2:F3 - 13:15); UOD ~1.5× LOD, IOD ~1.05× UOD (UOD:IOD:LOD - 49:52:32); frontal line carinate between antennal bases for a length less than OD, flat just below median ocellus for less than 1OD and otherwise roughly defined; MOC shorter than width of head (76:83); OOC ~0.7× IOC (12:17); genal area ~0.67× as wide as compound eye in lateral view (19:28); ratio of lengths of mesoscutum: scutellum: metanotum: metapostnotum - 52:20:10:14; probasitarsus ~3.33× as long as maximum depth (20:6); prodistitarsus 0.8× as long as preceding three tarsomeres combined (from II to V - 6:5:4:12); metafemur ~1.75× as long as maximum depth (54:31); summit of metatibial ventral convexity at approximately two-thirds tibial length; metatibia 2.2× as long as maximum depth (44:20); in apical view apical lamina of metatibia short and thick, length ~0.8× OD (7:9); ventral metatibial carina bowed ventrad and absent basally, originating at midpoint of weakly sigmoid posterior carina; metabasitarsus ~4.2× as long as maximum depth (36:8.5); S1 process apical surface with a longitudinal median ridge, in lateral view anterior and posterior margins convergent apically. S7 apodemal arm sclerotized margin terminating laterally at arm midlength; ventral lobe narrow, almost linear; dorsal lobe roughly triangular, wide at base and narrow apically; row of setae long basally and shorter apically; apex of disc disinctly emarginate. S8 lateral process ~0.9× as wide as long, anteromedial sclerotized margin following marginal contour. Gonobase apicoventral truncate process biconvex and distinctly notched by ~1.5× width of one convexity.

**Female.** Length 4.3–5.3mm, forewing length 2.8–3.0mm, head width 1.0–1.1mm, thorax width 1.1–1.2mm, median ocellar diameter (OD) 0.11–0.12mm.

*Colouration*: Black to black-brown except as follows: mandible yellow except apex translucent brown, sometimes trasluscent yellow in apical half; antenna variable (pedicel and F1-F2 apicoventral surface narrowly brown to broadly yellow-brown; ventral surface of F3 to penultimate flagellomere yellow, often suffused with brown basally; terminal flagellomere yellow basally, brown apically). Following parts yellow: dorsal surface of protibia; apical one-third of prodistitarsus; narrow basal and apical rings on mesotibia; wide basal ring on metatibia, narrow ventrally, as well as apical rim posteriorly; anterior spot on tegula. Following parts yellow-brown: anterior surface of probasitarsus; apicodorsal rim of metafemur. Apical one-third of mesodistitarsus brown. Metasoma black, T1-T5 yellow-brown beyond premarginal line to translucent yellow at margins.

*Pubescence*: As in male except as follows: clypeal hairs short and sparse (0.5OD); genal beard sparse (0.5–1.5OD); discs of mesoscutum and scutellum with sparse short hairs (≤0.5OD) denser and short toward lateral margin of mesoscutum (0.5OD), longer and denser toward lateral and posterior margins of scutellum (1–1.5OD) longest posteromedially; scopae on metafemur and metatibia (1–1.5OD); T1-T2 apicolateral patches of tomentum (0.5–1OD), T3 apicolateral hair band sparse, not tomentose; S1 hairs long and moderately dense (≤2OD); S2 scopal hairs (1–2OD).

*Surface sculpture*: As in male except as follows: frontal area densely punctate (i≤d), moderately densely punctate around ocelli (i=1-2d); vertexal area moderately densely punctate (i=1-2d); scape shallowly and moderately densely punctate (i=1-2d); genal area moderately densely punctate (i=1-2d); hypostomal area weakly imbricate to glossy apically and sparsely punctate (i≥2d); mesoscutum and scutellum densely punctate (i=0.5-1d); mesepisternum irregularly punctate, sparse below scrobe (i=1-3d), sparser anterior of episternal groove (i≥3d), impunctate just dorsad of scrobe, otherwise irregularly spaced on hypoepimeral area (i≤2d); metanotum moderately densely punctate (i=0.5-2d); metapostnotum rugose, more weakly microsculptured posteriorly; propodeum moderately densely punctate (i=1-2d); T1-T5 punctures small and sparse (i=1-3d on T1-T3; i≥2d on T4-T5); T6 moderately densely punctate (i=1-2d).

*Structure*: Labrum ~2.2× wider than long (18.5:8.5); length of malar space ~0.4× as long as clypeal lateral (2.5:6); LOT below anterior tentorial pits; clypeus subequal in length to maximum width in frontal view (24:23) extending for approximately one third of its length beyond LOT; median longitudinal groove weak on clypeus; subantennal sutures shorter than the shortest distance between them (9.5:11); IAD ~1.6× AOD (11.5:7); scape ~3.33× as long as maximum width (20:6); pedicel longer than wide (7:6); F1 shorter than wide (4.5:6); F2 slightly shorter than F1 and slightly shorter than F3 (F2:F3 - 4:4.5); UOD ~1.4× LOD, IOD ~1.05× as long as UOD (UOD:IOD:LOD - 44:46:31); frontal line carinate in lower half, flat above; MOC subequal to width of head (66:67.5); OOC ~0.67× IOC (11:17); genal area ~0.6× as wide as eye in lateral view (14:22); ratio of lengths of mesoscutum: scutellum: metanotum: metapostnotum – 47:17:9.5:11; probasitarsus 3.8× as long as maximum depth (19:5); length of prodistitarsus ~0.8× that of preceding three tarsomeres combined (from II to V - 6:4:3.5:11); metafemur ~2.7× as long as maximum depth (38:14); metatibia ~4.4× as long as maximum depth (48:11); metabasitarsus ~4.1× longer tshan maximum depth (29:7).

###### Material studied

(84 males & 201 females). **Neotype** (male): Region IV, Elqui Prov., 26 km S of Vicuña, *S 30.190°, W 70.661°, 1691m*, 5.x.1994, Rozen, Quinter, Ascher (AMNH) - see comments; **Holotype** (male): Region IV, El Pangue, *S 30.168°, W 70.663°, 1711m*, x.1972, Toro (AMNH); **Allotype** (female): same locality and date as holotype, Cabezas (AMNH); **Region IV**: one paratype male, same data as holotype, on *Pleurophora pusilla* (AMNH); one female, same locality as holotype, 13.x.1977, De la Hoz (PUCV); one female, same locality as holotype, 13.x.1977, L. Ruz (PUCV); two females, Elqui Prov., Pangue, S 30.1539°, W 70.6639°, 1686m, 11-30.ix.2004, A. Ugarte (PCYU); one female, Elqui Prov., El Pangue 24 km S of Vicuña, *S 30.168°, W 70.663°, 1711m*, 31.x.1992, Snyder & Sharkov (AMNH); four females, Elqui Prov., 7 km S of Pisco Elqui, *S 30.177°, W 70.486°, 1449m*, 8.x.1994, Rozen, Quinter, Ascher (AMNH); four females, Elqui Prov., 26 km S of Vicuña, *S 30.19°, W 70.661°, 1691m*, 1.xi.1992, Rozen, Sharkov, Snyder (AMNH); two females, Elqui Prov., 26 km S of Vicuña, *S 30.19°, W 70.661°, 1691m*, 5.x.1994, Rozen, Quinter, Ascher (AMNH); two males and two females, Elqui Prov., 19 km S of Pisco Elqui, *S 30.265°, W 70.499°, 1991m*, 6.x.1994, Rozen, Quinter, Ascher (AMNH); one female, S of Vicuña km 97, S 30.1415°, W 70.6901°, 1304m, 3.x.2013, S. Monckton, CCDB-19989 B06 // PCYU 0021680 (PCYU); one female, Elqui Prov., 6 km S of Vicuña, *S 30.085°, W 70.725°, 889m*, 23-24.x.1992, Rozen, Sharkov, Snyder (AMNH); one female, Vicuña, *S 30.032°, W 70.708°, 625m*, 12.ix.1968, H. Toro (AMNH); one male, same locality, 26.x.1972, H. Toro (AMNH); one female, Vicuña, *S 30.032°, W 70.708°, 1157m*, 26.x.1972, H. Toro (PUCV); four females, E of Rivadavia, 41-CH, km 83, S 29.9619°, W 70.5411°, 855m, 5.xi.2013, S. Monckton (PCYU); one male and two females, Elqui Prov. bet. Chapilca & Guanta, *S 29.844°, W 70.461°, 1107m*, 7.x.1994, Rozen, Quinter, Ascher (AMNH); one female, E of Guanta, km 114.420, S 29.8852°, W 70.3214°, 1349m, 4.x.2012, L. Packer (PCYU); eight males and nineteen females, E of Vicuña, km 114, S 29.9123°, W 70.3013°, 1429m, ix.2010, L. Packer, white pans (PCYU); three males and thirteen females, same data, blue pans (PCYU); four males and sixteen females, same data, fluor. yellow pans (PCYU); five males and one female, E of Guanta, km 127.840, S 29.8852°, W 70.2602°, 1613m, 12.ix.2010, L. Packer (PCYU); one female, Puente Las Terneras, S 29.9888°, W 70.2522°, 1644m, 4.v.2010, Packer & Fraser, B09866-A08 Bees of Chile197 (PCYU); three females, same locality, 4.x.2010, L. Packer, CCDB-19989 C11 // PCYU 0021693, PCYU 0021700, PCYU 0021699 (PCYU); thirty-six males and eighty-four females, E of Vicuña, 127.8 km, S 29.9777°, W 70.2293°, 1711m, 12-19.ix.2010, L. Packer (PCYU); three males and three females, same data (BBSL); three males and three females, same data (CTMI); one male, Hwy 41-CH, km 133, S 29.9657°, W 70.2015°, 1716m, 17.x-8.xi.2013, S. Monckton (PCYU); one male and one female, 41-CH km 137.8, S 29.9670°, W 70.1936°, 1800m, 8.xi.2013, S. Monckton, pan traps, CCDB-19989 B11 // PCYU 0021685; CCDB-19989 B12 // PCYU 0021686 (PCYU); one female, Hwy 41-CH, km 132, S 29.0541°, W 70.1936°, 800m, 8.xi.2013, S. Monckton (PCYU); one female, El Tofo, *S 29.442°, W 71.252°, 631m*, 27.x.1972, H. Toro (AMNH); two females, Incahuasi, *S 29.228°, W 71.014°, 779m*, 1.x.1982, O. Martinez (PUCV); two females, same locality and date, De La Hoz (PUCV); two males and one female, Chañares, *S 30.295°, W 70.629°, 1389m*, 12.ix.1984, H. Toro (PUCV); two males and two females, same data (AMNH); one male, same locality and date, De La Hoz (PUCV); one male, same locality and date, De La Hoz, PUCV-ENTO 21886 (PUCV); two males and one female, same locality and date, De La Hoz (AMNH); one female, same locality and date, P. Guerrero (PUCV); one male and two females, Chañar-Los Lavadores, S 30.2963°, W 70.6273°, 1396m, 10.ix.2010, L. Packer, B09866-F09 Bees of Chile258, B09866-F07 Bees of Chile256, B09866-F08 Bees of Chile257 (PCYU); two males and two females, Las Breas, *S 30.369°, W 70.613°, 1622m*, 11-19.ix.2010, L. Packer (PCYU); one male, Elqui, 4–5 km S of Pisco, *S 30.383°, W 70.347°, 1340m*, 27.x.1992, Andrey Sharkov (AMNH); seven females, Limarí Prov., Embalse Recoleta, 21 km NE of Ovalle, *S 30.495°, W 71.086°, 406m*, 11-12.x.1994, Rozen, Quinter, Ascher (AMNH); one female, Limarí Prov., Las Mollacas, E Monte Patria, *S 30.753°, W 70.657°, 1142m*, 13.x.1994, Rozen, Quinter, Ascher (AMNH); one female, same data, on *Salix chilensis* (AMNH); **Region III**: one female, E of Vallenar, S 28.7716°, W 70.4486°, 830m, 1.x.2013, L. Packer (PCYU); one male and three females, Embalse Santa Juana, C-479, km 21.5, S 28.6747°, W 70.6433°, 645m, 15.x.2013, S. Monckton, CCDB-19989 A08 // PCYU 0021670, CCDB-19989 A09 // PCYU 0021671, PCYU 0020916, PCYU 0020915 (PCYU).

###### Variation.

Some females have a yellow-brown or yellow spot on the lower paraocular area below the anterior tentorial pit.

###### Distribution.

Central Andean Cordillera and Coquimban & Intermediate Desert, from Embalse Sta. Juana (Region III) south to Las Mollacas (Region IV), west to El Tofo, and east to E of Vicuña, Hwy. 41-CH km 137.8 (Region IV); 406–1991m a.s.l.

###### Ecology.

Collected on *Pleurophora pusilla* Hook. & Arn. (1 record) and *Salix chilensis* Molina (1 record). Recorded September to November and May.

###### Comments.

Designation of the neotype is justified because of damaged type material; identification of *Chilicola vicugna* relies on characters on the head, without which it is not readily differentiated from *Chilicola mavida*. However, the male holotype and single male paratype have lost their heads since the time of original description, and the taxonomic identity of *Chilicola vicugna* cannot, therefore, be strictly verified based on existing type material; this renders *Chilicola vicugna* a *nomen dubium*. The neotype described herein is so designated in the interest of taxonomic stability, and was the subject of a proposal to the International Commission on Zoological Nomenclature (ICZN) to set aside the name-bearing status of the holotype and to officially designate the neotype in its place (Monckton 2014).

##### Chilicola (Heteroediscelis) vina

Taxon classificationAnimaliaHymenopteraColletidae

Toro & Moldenke, 1979

**Figure 48.**
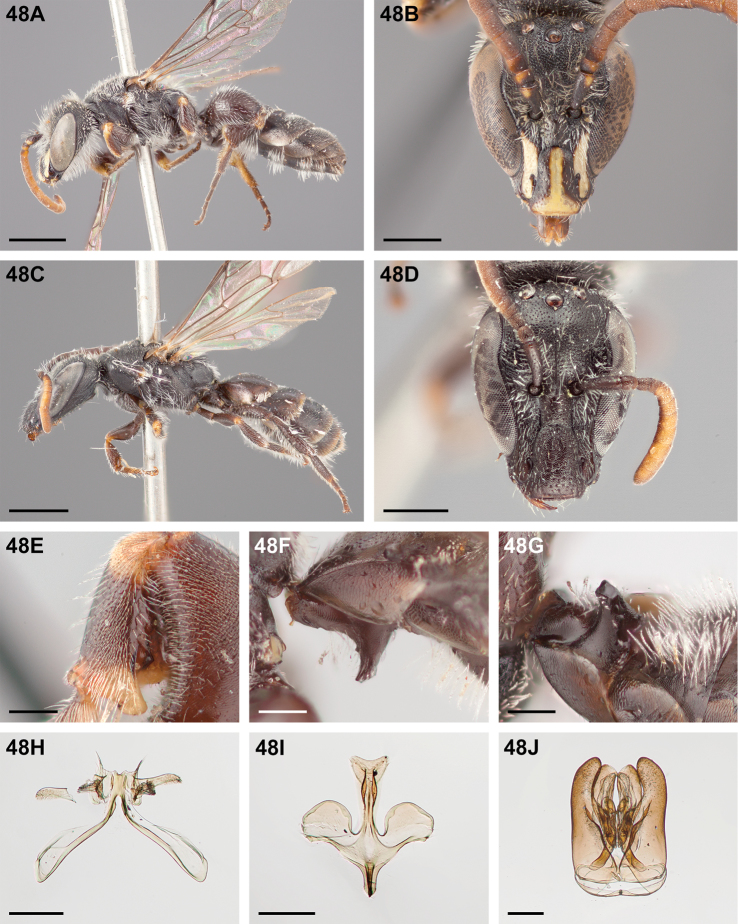
*Chilicola vina*: **A** male habitus, lateral view, scale bar 1 mm **B** male head, frontal view, scale bar 0.5 mm **C** female habitus, lateral view, scale bar 1 mm **D** female head, frontal view, scale bar 0.5 mm **E** male metatibia, posterior view, scale bar 0.25 mm **F** male S1 process, lateral view^†^, scale bar 0.25 mm **G** male S1 process, apical view, scale bar 0.25 mm **H** male S7, scale bar 0.25 mm **I** male S8, scale bar 0.25 mm **J** male genital capsule, scale bar 0.25 mm.

**Figure 49.**
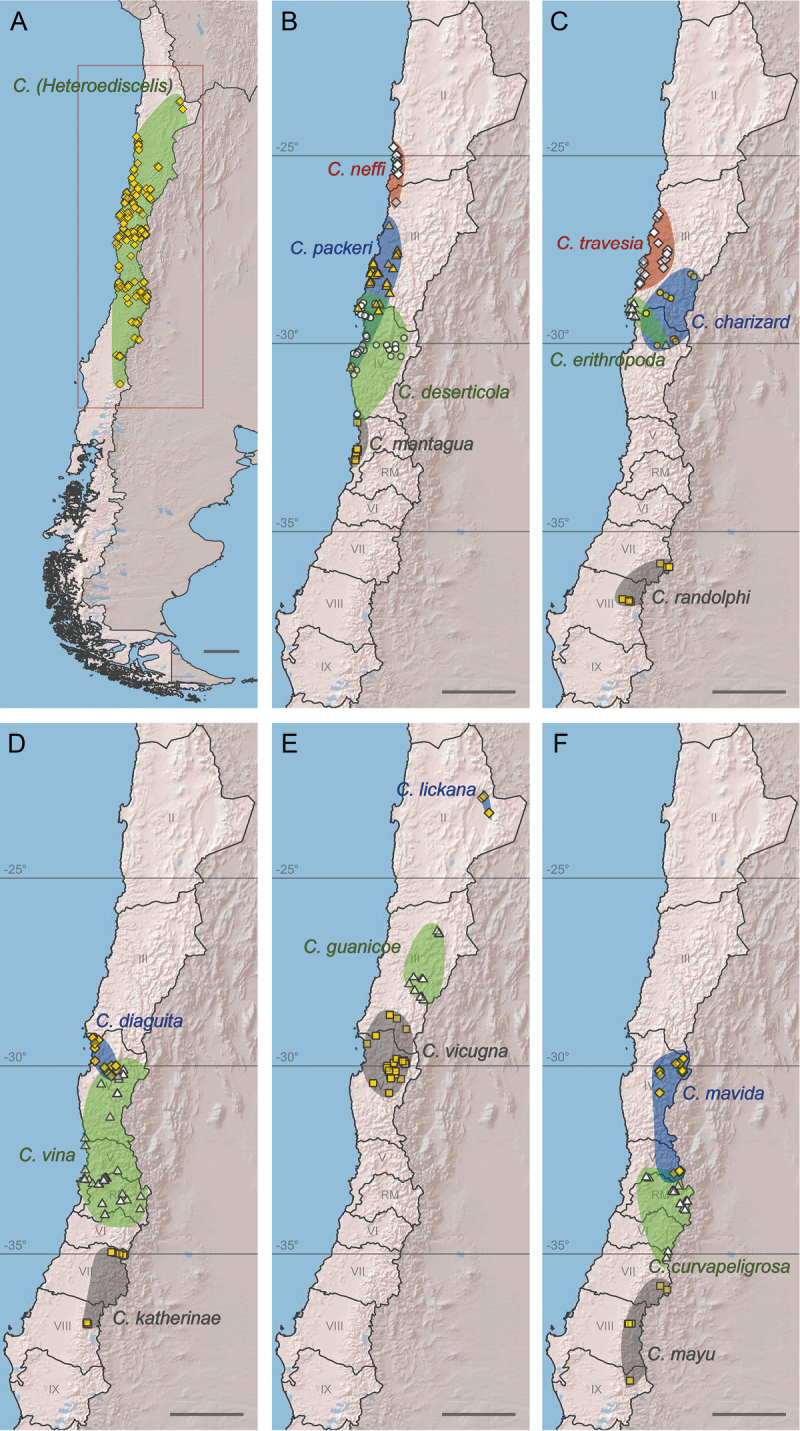
Distribution of Chilicola (Heteroediscelis) by species. Original and geo-referenced records are plotted as diamonds, circles, triangles, and squares in alternating yellow and white; speculative ranges are plotted in contrasting colours, and are smoothed, slightly enlarged convex hulls. Scale bars approximately 200 km, background imagery provided by ESRI.

Figs 13B–C ; 14C–D ; 18E–F ; 19B ; 20B ; 48A–J ; 49

Chilicola vina Toro & Moldenke, 1979: 124–125 (male holotype, female allotype, AMNH [examined]). Packer 2008: 77. Montalva and Ruz 2010: 29 (checklist). Moure et al. 2012 (catalogue). Ascher and Pickering 2015 (checklist).

###### Diagnosis.

Males are diagnosable by the combination of malar space 1.2–1.4× as long as the clypeal lateral, apical surface of the S1 process weakly longitudinally concave, and the LOT passing through the anterior tentorial pits. Females are diagnosable by the combination of protibia yellow on basal half of dorsal surface only, malar space 1.2–1.3× as long as clypeal lateral, and LOT passing through the anterior tentorial pits. Longer-faced individuals of both sexes may be confused with *Chilicola diaguita* and are best distinguished by the position of the LOT, which in *Chilicola diaguita* passes above or just tangent to the upper margin of the anterior tentorial pits. Additionally, males of *Chilicola vina* have the S8 anteromedial sclerotized margin swollen, forming a convex incursion into the membranous interior (sclerotized edge subparallel to margin throughout in *Chilicola diaguita*).

###### Description.

**Male.** Length 6.0–6.5mm, forewing length 3.5–3.7mm, head width 1.3mm, thorax width 1.3–1.4mm, median ocellar diameter (OD) 0.13mm.

*Colouration*: Black-brown, following parts yellow: labrum except apical margin; inverted T shape on clypeus, apex dark laterad; lower paraocular area at most up to level of transverse portion of epistomal suture medially, extending further laterally but not reaching lower tangent of antennal socket; apicoventral spot on scape; apicoventral surface of pedicel; protrochanter apical rim posteriorly; apex of profemur broadly on anterior surface, narrowly on posterior surface; dorsal surface of protibia, including dorsal half of anterior surface; narrow apical ring on mesofemur; narrow basal ring and apicodorsal rim of mesotibia; wide basal ring on metatibia, narrow apical ring wider ventrally; basal half to two-thirds of metabasitarsus ventrally; anterior spot on tegula. Ventral surface of antennal flagellum yellow-brown, narrowing basally on F1, F11 brown. Apical half of prodistitarsus variably yellow to yellow-brown. Ventral metatibial carina black. Metasoma black, T1-T7 marginal zones translucent yellow.

*Pubescence*: White hairs generally short (0.5OD) and sparse, not especially plumose; on lower paraocular area and around antennal base long and moderately dense (1.5-2OD); genal beard long and moderately dense (0.5-3OD) longest at midlength; mesoscutum, scutellum, and metanotum mostly bare, few short hairs (0.5OD), longer and denser toward lateral margin as follows: mesoscutum (0.5-1OD), scutellum (1-2OD) also on posterior margin, longest posteromedially, metanotum (≤2.5OD); propodeal hairs moderately long and dense dorsolaterally (0.5-2OD); T1-T3 apicolateral patches of tomentum (0.5-1OD); S2 hairs long and dense (1.5OD basolaterad, 0.5OD distilaterad, shorter mesad).

*Surface sculpture*: Microsculpture imbricate, integument generally dull; punctures generally small and deep. Clypeus and lower paraocular areas moderately densely punctate (i=1-2d) except clypeus sparsely punctate medially (i=2-3d); supraclypeal area moderately densely and irregularly punctate (i=1-4d) densely punctate toward lateral margin (i<d); frontal area very densely punctate (i≤0.5d), densely punctate around ocelli (i≤d); ocellocular space moderately densely punctate adjacent to compound eye (i=1-2d) becoming impunctate adjacent to lateral ocellus; vertexal area rugose and densely punctate (i≤d); scape shallowly and moderately densely punctate (i=1-2d) densely punctate apicoventrally (i≤d); genal area microstriate and moderately densely punctate (i=1-2d); hypostomal area weakly imbricate and moderately densely punctate (i=1-2d); pronotum densely punctate (i=0.5-1d), lateral surface coarsely imbricate; mesoscutum and scutellum densely punctate (i=0.5-1d) punctures crowded around median (i≤d), mesoscutum more sparsely punctate laterally (i=1-2d); mesepisternum irregularly punctate, sparse below scrobe (i=1-3d), sparser anterior of episternal groove (i≥3d), irregularly spaced on hypoepimeral area (i≤2d); metanotum moderately densely punctate (i=1-2d) punctures crowded around median and anteriorly (i≤0.5d); metepisternum densely punctate (i≤d) distinctly longitudinally striate anterodorsally; metapostnotum rugose, fewer transverse lineations posteriorly; propodeum coarsely imbricate, rugulose posteriorly, moderately densely punctate throughout (i=1-2d); T1-T5 coarsely imbricate; T1 sparsely punctate basally (i≥2d) moderately densely punctate apically (i≤2d); T2-T5 moderately densely punctate (i=1-2d) basal depressed area more coarsely microsculptured but punctures distinct; T6 coarsely imbricate posteriorly; T6-T7 moderately sparsely punctate (i=1-3d); marginal zones of terga weakly imbricate and shiny, minutely punctate.

*Structure*: Labrum 2.1× wider than long (21:10); malar space ~1.4× as long as clypeal lateral (11:8); LOT at level of anterior tentorial pits; clypeus ~1.25× as long as maximum width in frontal view (36:29) extending for approximately half its length beyond LOT; clypeus median longitudinal groove present but weak apically; subantennal sutures ~1.2× as long as the shortest distance between them (17:14); IAD ~1.6× AOD (11:7); scape 2.5× as long as maximum width (25:10); pedicel as long as wide (9:9); F1 shorter than wide (8.5:9.5); F2 ~1.6× as long as F1 and shorter than F3 (F2:F3 - 13.5:16); UOD 1.5× LOD, IOD ~1.1× UOD (UOD:IOD:LOD - 51:56:34); frontal line carinate in lower two-fifths, flat just below median ocellus for a length of ~1OD, otherwise roughly defined; MOC subequal to width of head (93:90); OOC ~0.67× IOC (12:18); genal area ~0.6× as wide as compound eye in lateral view (17:31); ratio of lengths of mesoscutum: scutellum: metanotum: metapostnotum - 62:23:14.5:19; probasitarsus 4× as long as maximum depth (24:6); length of prodistitarsus subequal to that of preceding three tarsomeres combined (from II to V - 7:6:4:16); metafemur ~2× as long as maximum depth (71:36); summit of metatibial ventral convexity broadly rounded and near tibial apex; metatibia ~1.9× as long as maximum depth (56:30); in apical view apical lamina of metatibia broad and thick, length subequal to OD (10:10); ventral metatibial carina toothed near apex, posterior carina sublinear and weakly defined basally; metabasitarsus ~4.67× as long as maximum depth (42:9); S1 process apical surface longitudinally concave at least posteriorly, in lateral view anterior and posterior margins divergent apically, apex appearing anteriorly bent. S7 apodemal arm sclerotized margin terminating laterally at arm midlength; ventral lobe ~0.33× as broad as basal attached length; dorsal lobe strap-like, subparallel-sided and somewhat anteriorly curved, with basal tuft of long setae and a short apical row, midlength bare. S8 lateral process ~1.2× wider than long, anteromedial sclerotized margin having distinct convex incursion, appearing swollen. Gonobase apicoventral truncate process biconvex and distinctly notched by a width less than one convexity.

**Female.** Length 4.9–6.1mm, forewing length 3.2–3.5mm, head width 1.1–1.2mm, thorax width 1.1–1.3mm, median ocellar diameter (OD) 0.12mm.

*Colouration*: Black to black-brown except as follows: mandible with yellow basal spot, translucent yellow in apical half except apex translucent brown. Following parts variably brown to black: pedicel, F1 and F2, on apicoventral margins; apicodorsal rim of metafemur; wide basal ring on metatibia. Following parts variably yellow to yellow-brown: ventral surface of F3-F10, suffused with brown basally; dorsal surface of protibia in basal half; apical half of prodistitarsus; basidorsal spot on mesotibia. Anterior spot on tegula absent or faintly translucent yellow. Metasoma black, T1-T5 marginal zones translucent yellow.

*Pubescence*: As in male except as follows: on lower paraocular area and around antennal base moderately long and moderately dense (1OD); genal beard sparse (0.5–2OD); discs of mesoscutum, scutellum, and metanotum with spase short hairs (≤0.5OD), denser and as long or longer toward lateral margin as follows: mesoscutum (≤0.5OD), scutellum (1–1.5OD) also on posterior margin, longest posteromediallym metanotum (≤2OD); propodeal hairs moderately long and dense dorsolaterally (0.5–1.5OD); scopae on metafemur and metatibia (1–2OD); T1-T2 apicolateral patches of tomentum (0.5–1OD), T3 apicolateral hair band sparse, not tomentose; S1 hairs long and moderately dense (≤2.5OD); S2 scopal hairs (1–3OD).

*Surface sculpture*: As in male except as follows: clypeus moderately densely punctate throughout (i=1-2d); supraclypeal, vertexal, and genal areas moderately densely punctate (i=1-2d) except supraclypeal area more densely punctate toward lateral margin (i≤d); frontal area densely punctate (i≤d); scape shallowly and moderately densely punctate (i=1-2d); hypostomal area weakly imbricate to glossy apically, sparsely punctate (i≥2d); mesoscutum and scutellum densely punctate (i=0.5-1d); mesepisternum irregularly punctate, sparse below scrobe (i=2-4d), sparser anterior of episternal groove (i≥4d), impunctate just dorsad of scrobe, otherwise irregular and moderately dense on hypoepimeral area (i≤2d); metanotum moderately densely punctate (i=0.5-2d); metapostnotum rugose, fewer transverse lineations posteriorly; propodeum moderately densely punctate (i=1-2d); T1-T5 punctures small and sparse (i=1-3d); T6 moderately densely punctate (i=1-2d).

*Structure*: Labrum 2.3× wider than long (23:10); malar space ~1.3× as long as clypeal lateral (9.5:7.5); LOT at level of anterior tentorial pits; clypeus ~1.1× as long as maximum width in frontal view (33:29) extending for approximately half its length beyond LOT; median longitudinal groove weak on clypeus; subantennal sutures subequal in length to the shortest distance between them (13.5:13); IAD ~1.33× AOD (12:9); scape ~3.6× as long as maximum width (25:7); pedicel as long as wide (6.5:6.5); F1 as long as wide (8:8); F2 shorter than F1 and shorter than F3 (F2:F3 - 4.5:6); UOD ~1.4× LOD, IOD ~1.1× as long as UOD (UOD:IOD:LOD - 49:53:36); frontal line carinate in lower third, flat above; MOC ~1.1× as long as width of head (86:79); OOC ~0.65× IOC (12:19); genal area ~0.65× as wide as eye in lateral view (17:26); ratio of lengths of mesoscutum: scutellum: metanotum: metapostnotum – 58:21:10:14; probasitarsus 4× as long as maximum depth (24:6); length of prodistitarsus ~0.9× that of preceding three tarsomeres combined (from II to V - 6:5:5:14); metafemur ~3.1× as long as maximum depth (50:16); metatibia ~4.2× as long as maximum depth (59:14); metabasitarsus ~4.4× as long as maximum depth (40:9).

###### Material studied

(58 males & 71 females): **Holotype** (male): Region V, Cuesta la Dormida, *S 33.067°, W 71.019°, 1197m*, Moldenke (AMNH); **Allotype** (female): same data as holotype (AMNH); **RM Santiago**: one paratype female, Pr. Valparaíso & Santiago, Cuesta La Dormida, *S 33.068°, W 70.998°, 1078m*, 1970-72, A.R. Moldenke, INT BIOL PROGRAM (AMNH); one female, same locality, 20.x.1971, A.R. Moldenke, on *Loasa*, INT BIOL PROGRAM 31210 (AMNH); one female, same locality, 20.x.1971, A.R. Moldenke, on *Loasa*, INT BIOL PROGRAM 31209 (AMNH); one female, same locality, 20.x.1971, A.R. Moldenke, on *Stachys grandidentata*, INT BIOL PROGRAM 31245 (AMNH); two females, same locality, 9.x.1971, A.R. Moldenke, INT BIOL PROGRAM 31104, INT BIOL PROGRAM 31108 (AMNH); two males and two females, Chacabuco: Cuesta la Dormida, *S 33.068°, W 70.998°, 1078m*, 15.xi.1992, Rozen, Sharkov, Snyder (AMNH); two females, Caleu, *S 32.998°, W 70.977°, 1054m*, 3.x.2000, L. Packer (PCYU); one male, nr. Caleu, S 33.0089°, W 70.9966°, 1230m, 26.x.2010, L. Packer, B09857-C02 Bees of Chile310 (PCYU); one male and seven females, Espinalillo, S 33.0050°, W 70.9406°, 877m, 5.v.2013, L. Packer (PCYU); one male and one female, same data (CTMI); two males, El Paico, *S 33.689°, W 71.055°, 252m*, i.1975, Luis E. Peña (AMNH); three males and two females, Pte. Alto, *S 33.617°, W 70.567°, 705m*, 5.xii.1999, F. Vivallo, on *Aloysium citriodora* (UFRJ); one male and one female, same locality and collector, 10.i.2000, on *Aloysium citriodora* (UFRJ); eleven males, same locality and collector, 4.ii.2001 (UFRJ); eight males, same locality and collector, 4.ii.2001, on *Aloysium citriodora* (UFRJ); three males, same locality and collector, 4.iii.2001, on *Aloysium citriodora* (UFRJ); two males, same locality and collector, 25.xii.2001, F. Vivallo (UFRJ); five males, same locality and collector, 25.xii.2001, F. Vivalo, on *Aloysium citriodora* (UFRJ); one male, Quebrada de San Ramón, *S 33.433°, W 70.516°, 853m*, xii.1974, L.E. Peña (AMNH); one female, Río Maipo, El Manzano, *S 33.587°, W 70.395°, 898m*, 18.i.1975, M. Pasten (AMNH); one male, E of El Volcán, S 33.4971°, W 70.0263°, 1953m, 7.i.2009, L. Packer, CCDB-03755 A09 // PCYU CHI09-9-3-016 (PCYU); one male, E of El Volcán, S 33.8282°, W 70.0437°, 1963m, 31.xii-7.i.2013, L. Packer & R. Smith, pan trap, CCDB-22789 D09 (PCYU); **Region IV**: one female, Los Vilos path of Lobos, *S 31.938°, W 71.515°, 15m*, 11.v.2010, Packer & Fraser, B09866-D01 Bees of Chile226 (PCYU); fourteen females, Limarí Prov., Las Mollacas, E of Monte Patria, *S 30.753°, W 70.657°, 1142m*, 13.x.1994, Rozen, Quinter, Ascher, on *Oxalis* (AMNH); one male and three females, Los Molles, S 30.7496°, W 70.6486°, 1147m, 12.x.2013, S. Monckton, CCDB-19989 B01 // PCYU 0021675, CCDB-19989 B02 // PCYU 0021676, PCYU 0020825, PCYU 0020826 (PCYU); one female, Limarí Prov., Embalse Recoleta, 21 km NE Ovalle, *S 30.495°, W 71.086°, 406m*, 11-12.x.1994, Rozen, Quinter, Ascher (AMNH); one female, Las Breas, *S 30.369°, W 70.613°, 1622m*, 23.ix.1980, H. Toro (AMNH); one male, Chañares, *S 30.295°, W 70.629°, 1389m*, 12.ix.1984, H. Toro (PUCV); one male and two females, Limarí Prov., Chañar, S 30.2804°, W 70.6402°, 1520m, 11-30.ix.2004, A. Ugarte (PCYU); four females, Limarí Prov., Chañar, S 30.2804°, W 70.6402°, 1564m, 4.ix.2004, L. Packer (PCYU); four males, Elqui: 16 km south Pisco, *S 30.248°, W 70.495°, 1811m*, 26.x.1992, A. Sharkov (AMNH); one female, Elqui Prov., 14 km S of Pisco Elqui, *S 30.23°, W 70.495°, 1733m*, 6.x.1994, Rozen, Quinter, Ascher (AMNH); two females, Elqui Prov., 26 km S of Vicuña, *S 30.19°, W 70.661°, 1691m*, 5.x.1994, Rozen, Quinter, Ascher (AMNH); one male, El Pangue, *S 30.168°, W 70.663°, 1711m*, x.1972, H. Toro (PUCV); four females, Elqui Prov., Pangue, S 30.1539°, W 70.6639°, 1686m, 11-30.ix.2004, A. Ugarte (PCYU); five females, Elqui Prov., Pisco Elqui, *S 30.122°, W 70.493°, 1247m*, 6.x.1994, Rozen, Quinter, Ascher (AMNH); one male, El Tofo, *S 29.442°, W 71.252°, 631m*, x.1972, L. Ruz, on *Flourensia* (PUCV); **Region V**: one female, Pr. Aconcagua, Papudo/Zapallar, i.1973, A.R. Moldenke, 45330 INT BIOL Program (AMNH); one female, Paihuén, *S 31.384°, W 70.848°, 501m*, 4.xii.1983, M. Rojas (PUCV); one female, Choapa, Pichidangui, *S 32.138°, W 71.507°, 15m*, 10.ii.2003, F.D. Parker, pan trap, CHL-14610-84 // USDA Native Bee Survey BBSL695284 (BBSL); one female, Loncura, *S 32.786°, W 71.508°, 38m*, 5.x.2000, L. Packer (PCYU); one male, Aconcagua: Los Andes, *S 32.809°, W 70.645°, 767m*, xii.1989, Luis E. Peña (AMNH); one female, Quinta Vergara, S 33.0321°, W 71.5526°, 71m, 18.xi.2000, F. Vivallo (UFRJ); one male, El Salto, *S 33.041°, W 71.522°, 31m*, 6.xi.1966, L. Ruz (PUCV); one female, Belloto, *S 33.048°, W 71.408°, 127m*, 22.x.1967, collector unknown (PUCV); one male, Cuesta la Dormida, *S 33.067°, W 71.019°, 1197m*, 26.x.2010, L. Packer, PCYU 0004674 (PCYU); one male, Valparaíso Prov., Río Marga Marga, Los Perales, *S 33.15°, W 71.317°, 330m*, 13.x.1966, M. Irwin & E.I. Schlinger (BBSL); one male, Colliguay, *S 33.171°, W 71.164°, 491m*, 17.ix.1976, O. Martinez (PUCV); two females, Colliguay, Las Compuertas, *S 33.169°, W 71.134°, 515m*, 4.x.2000, L. Packer, CCDB-03755 C03, CCDB-19989 C08 (PCYU); two females, Alto de Cantillana, Alhue, *S 33.979°, W 70.987°, 1398m*, 25.xi.2000, L. Packer (PCYU).

###### Distribution.

Coquimban Desert, Central Coastal Cordillera, Central Valley, and Southern Andean Cordillera, from Viña del Mar (Region V) north to Pisco Elqui (Region IV), south to Alto de Cantillana (RM Santiago), and east to E of El Volcán (RM Santiago); 15–1963m a.s.l.

###### Ecology.

Collected on *Aloysia citriodora* Palau (19 records), *Flourensia* DC. (1 record), *Loasa* Adans. (2 records), *Oxalis* (14 records), and *Stachys grandidentata* Lindl. (1 record). Recorded September to March and May.

### Phylogenetic analysis

**Figure 50.**
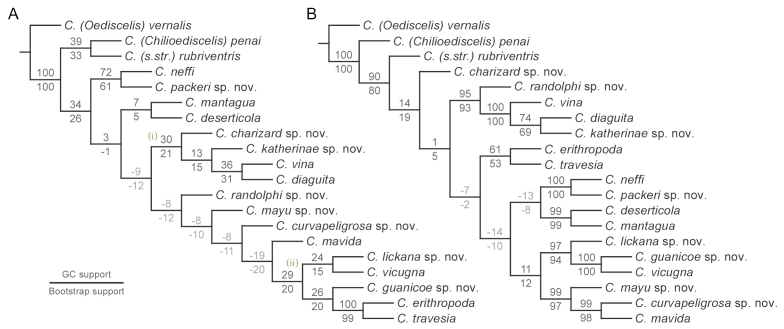
Results of maximum parsimony analysis of: **A** morphological data only **B** DNA barcode data only. GC support values are indicated above branches, bootstrap support values are below. Groups (i) and (ii) are discussed in text.

Analysis of 74 morphological characters (discrete and continuous) produced one most parsimonious tree with a fit of 13.3, CI = 37.4, RI = 49.7 (Fig. 50A). Most groups were found to have negative support from symmetric resampling and bootstrap analysis, meaning that these groups were more often contradicted than supported in resampling routines; groups receiving stronger support (i-ii) are discussed below.

Analysis of 658 base pairs from CO1 sequence data produced one most parsimonious tree with a fit of 42.3, CI = 51.9, RI = 61.4 (Fig. 50B). On the molecular-based tree only, *Chilicola charizard* was recovered as sister to the remaining members of the subgenus, but with near-zero support; moreover, in this arrangement, its sister group is not defined by any discernible morphological character states. The remaining groups have either negative support and are not recovered in other analyses or have low, moderate, or high support and are also recovered in the subsequent analysis (discussed below). This tree agrees with the morphology-based tree with respect to the species pairs [*Chilicola neffi* + *Chilicola packeri*], [*Chilicola erithropoda* + *Chilicola travesia*], and [*Chilicola deserticola + Chilicola mantagua*]. Both trees also support the grouping together of *Chilicola vina*, *Chilicola diaguita* & *Chilicola katherinae* and that of *Chilicola lickana*, *Chilicola guanicoe* & *Chilicola vicugna*, albeit in different arrangements within the groups.

**Figure 51.**
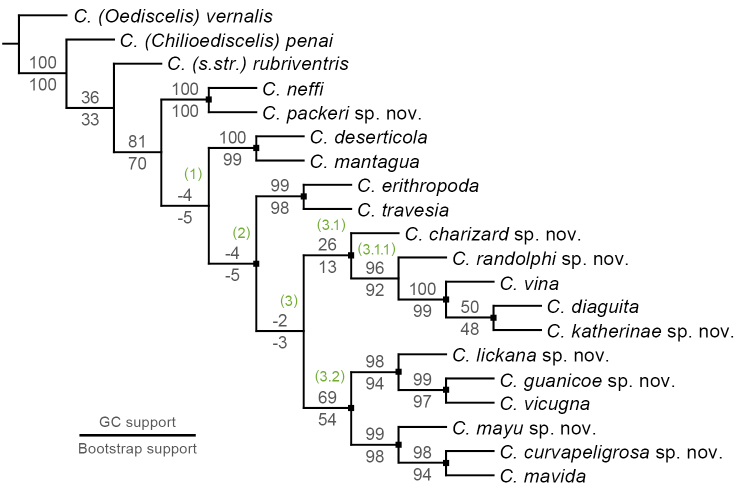
Single most parsimonious tree resulting from maximum parsimony analysis of combined morphological and molecular dataset. GC support values are indicated above branches, bootstrap support values are below. Nodes with distributional disjunctions are indicated by a black square. Numbered groups are discussed in text.

Combined analysis of morphological and molecular data produced a single most parsimonious tree with a fit of 28.8, CI = 45.9, RI = 55.0 (Fig. 51). This phylogeny has higher support overall than either morphological or molecular data alone, and monophly is moderately or strongly supported by resampling for a majority of the groups found. Hereafter, larger clades within this phylogeny are referred to by number for ease of reference (Fig. 51). Groups 1-3 appear at first to be problematic due to the lack of resampling support, but each is defined by at least two synapomorphies, and well supported by additional morphological characters, which are summarized below (see Suppl. material 1 for character-annotated tree, Fig. S1). Groups 3.1.1 and 3.2 (Fig. 51) and all species pairs were recovered in both the combined and molecular-only analyses, while species pairs [*Chilicola neffi* + *Chilicola packeri*], [*Chilicola erithropoda* + *Chilicola travesia*], and [*Chilicola deserticola* + *Chilicola mantagua*] were consistently recovered in all three analyses. The strongest contradictions to the combined analysis phylogeny come from groups (i) and (ii) in the morphology-only result. First, group (i) excludes *Chilicola randolphi* relative to the combined analysis tree, and is supported by a handful of homoplasies, particularly by the S1 process with apically divergent margins in lateral view and the malar space subequal to or longer than clypeal lateral; including *Chilicola randolphi* requires a reversion in both characters. Support for this grouping is otherwise low, however, while the combined analysis recovers *Chilicola randolphi* as sister to [*Chilicola vina* + (*Chilicola diaguita* + *Chilicola katherinae*)] with very high support (group 3.1.1). Second, group (ii) notably has [*Chilicola erithropoda* + *Chilicola travesia*] nested deep within the subgenus sister to *Chilicola guanicoe*, an arrangement supported by several morphological characters; however, this species pair has many homoplasic character state reversals in this placement, and occupies a much more parsimonious position in the combined analysis tree. Thus, the combined phylogeny is presented as the preferred result.

Monophyly of the subgenus is supported by a combination of the yellow posterior margin of the female pronotal lobe, the truncate S1 process in males, the female frontal area without longitudinal striae, the male basal vein before Cu half as long as Rs, the male S2 pubescence ≥1.5OD long basally and 1.5 longer basally than apically, and the male S7 dorsal lobe with main body transverse. The species pair [*Chilicola neffi* + *Chilicola packeri*] is sister to the rest of the subgenus (group 1) and is defined by two synapomorphies: the longitudinal depressions on the frontal area and the broad mesoventral lobe of the male gonoforceps. The pair additionally share a male S7 with a broad ventral lobe and apodemal arm with membranous portion completely enclosed by the sclerotized margin. Only these two species have a weakly and uniformly convex apical surface of the male S1 process, but whether this character state is synapomorphic or symplesiomorphic is ambiguous, since outgroup taxa lack a truncate S1 process with a defined apical surface. Either a convex apical surface of the S1 process is the ancestral state in *Heteroediscelis*, or it is derived from a longitudinally concave apical surface; both arrangements require the same number of steps, although the latter requires one fewer homoplasy.

Group 1 is defined by two synapomorphies: male clypeal apex yellow along its entire width, and a strap-shaped male S7 dorsal lobe. Members of this clade also have a male ventral metatibial carina which spans half the length or more of the longitudinal concavity, and the posterior carina lacks a sharp inflection at its base (this is regained in two species). Within group 1, and sister to group 2, the species pair [*Chilicola deserticola* + *Chilicola mantagua*] lacks any synapomorphies, and is instead grouped by a combination of several male homoplasies: the clypeus is moderately densely punctate throughout, the S1 process has a longitudinally concave apical surface, the S2 pubescence is relatively long, the apical margin of T7 is brown or black, the S7 discal apex is sinuate, and the dorsal lobe of S7 has a long basal tuft and short apical row of setae with midlength bare.

Group 2 is defined by two synapomorphies: male S1 process in lateral view with margins subparallel or divergent apically and male S7 dorsal lobe with a continuous row of setae long basally and shorter apically. Within this group, the pair [*Chilicola erithropoda* + *Chilicola travesia*] is defined by several synapomorphies, namely by the extensively pale leg colouration in both sexes and the unique shape of both the enlarged male metatibia and male S8 lateral process.

Group 3 is defined by three synapomorphies: the malar space is subequal to or longer than the clypeal lateral, the prodistitarsus is brown or black in males, and the male mesepisternum is more sparsely punctate in front of the episternal groove than behind. Additionally, the clypeus projects for at least half its length beyond the lower ocular tangent in all but three species, a character state present also in *Chilicola penai* but synapomorphic within the subgenus (an elongate clypeus and/or face has numerous independent instances throughout the Xeromelissinae). This group is subdivided into groups 3.1 and 3.2.

Group 3.1 lacks synapomorphies, but is characterized by a combination of three homoplasic states, invariant in all members: the male clypeal apex is brown laterally, the male S7 dorsal lobe has a long basal tuft and short apical row of setae with midlength bare, and the mesoventral lobe of the male gonoforceps has anterior and posterior margins concave. The group is additionally supported by the sinuate male S7 discal apex. Nested within, group 3.1.1 is defined by the structure of the male posterior metatibial carina, which is sublinear and weakly defined basally, and by the colouration of the female protibia, which is yellow only in the basal half of the dorsal surface. All members of this subgroup also have a male S1 process with longitudinally concave apical surface. The group [*Chilicola vina* + (*Chilicola diaguita* + *Chilicola katherinae*)] is united by six invariant homoplasies: the male clypeal maculation forms an inverted T shape, the male lower paraocular maculation extends no more than 1OD above the transverse epistomal suture, the genal beard is relatively sparse, the male S8 lateral process is ≥1.2 wider than long, the apicolateral hair patches of the female terga are tomentose on T1-T2 only, and the female legs are extensively dark. The pair [*Chilicola diaguita* + *Chilicola katherinae*] is not defined by any readily identified morphological characters; support for this pairing comes from molecular evidence, as the two share a synapomorphic substitution of C at site 595 of CO1 (relative to *Apis mellifera* L.) and homoplasic substitution of A at site 531 (although two divergent sequences from *Chilicola vina* have these same substitutions).

Group 3.2 is defined by three synapomomorphies: the male ventral metatibial carina is absent basally, the posterior carina extends the full length of the longitudinal concavity and is weakly sigmoid, and the apical surface of the male S1 process has a distinct longitudinal median ridge in all species. As well, the male S7 dorsal lobe is triangular, a convergent character state present in *Chilicola penai* but synapomorphic within *Heteroediscelis*. [*Chilicola lickana* + (*Chilicola guanicoe* + *Chilicola vicugna*)] has synapomorphic female leg colouration, and is further supported by the male S1 process in lateral view with margins convergent apically and by the relatively long male S2 pubescence. The pair [*Chilicola guanicoe* + *Chilicola vicugna*] is united by the lower paraocular maculation reaching the antennal socket (converged upon in other species, but unique in group 3.2). [*Chilicola mayu* + (*Chilicola curvapeligrosa* + *Chilicola mavida*)] is supported by synapomorphic male hindleg colouration, and the pair [*Chilicola curvapeligrosa* + *Chilicola mavida*] share a male third flagellomere subequal in length to the second (unique in group 3.2).

## Biogeography

**Figure 52.**
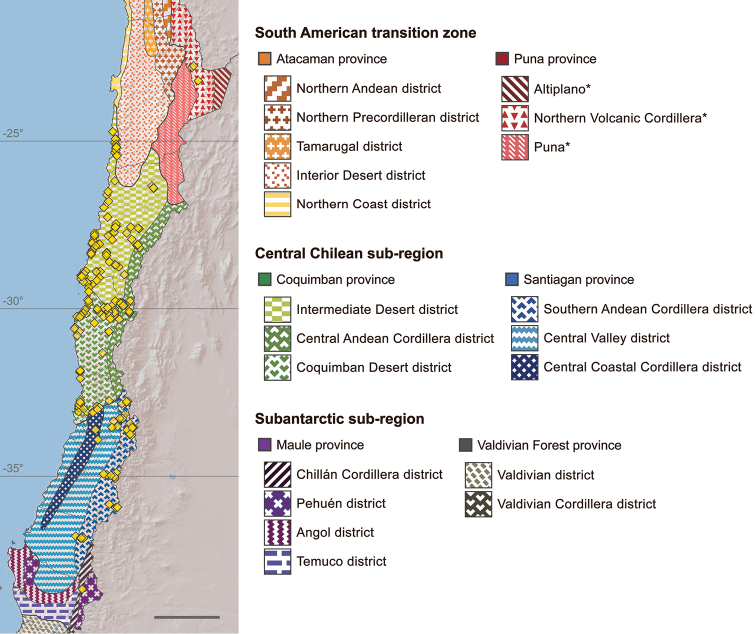
Chilean biogeographic areas overlaid with distribution of Chilicola (Heteroediscelis). Provinces are reproduced from Morrone’s regionalisation (2015) and districts are those proposed therein, with boundaries reproduced from their respective authors (Peña 1966, O’Brien 1971, Artigas 1975). Areas marked with an asterisk (*) represent undescribed portions of the Puna province, and are given placeholder names for ease of reference. Scale bar approximately 200 km, background imagery provided by ESRI.

Morrone’s (2015) biogeographic areas are outlined in Fig. 52, and species distributions are depicted in Fig. 49. Analysis with VIP identified 12 disjunct sister pairs in the consensus reconstruction, with disjunctions present at 12 out of 16 internal nodes; these are indicated on Fig. 51. Each pair of sister species exhibits a north-south disjunction – i.e. along a latitudinal gradient – while divergence along an elevational gradient is evident at two nodes: first, the disjunction between group 3 and [*Chilicola travesia* + *Chilicola erithropoda*] lies longitudinally between the Andes and lower interior to the east, and along the boundary between the Intermediate and Coquimban Deserts to the south, thus separating dry, northern, coastal and interior areas inhabited by [*Chilicola travesia* + *Chilicola erithropoda*], from more humid, southern, and Andean areas inhabited by group 3. The second is the disjunction between *Chilicola charizard* and group 3.1.1, which corresponds well to the boundary between the Intermediate and Coquimban Deserts at higher elevations, and at lower elevations runs north-by-northwest along coastal mountains. A third disjunction, between *Chilicola vina* and [*Chilicola diaguita* + *Chilicola katherinae*], also lies in part along an elevational gradient, but is discussed below.

Of the latitudinal disjunctions, seven correspond to previously identified boundaries between biogeographic areas, and three cut across biogeographic areas, seeming to lie along divisions between river systems. Of the latter three, two are associated with the Río Huasco: the disjunction between *Chilicola vicugna* and *Chilicola guanicoe* crosses the Intermediate Desert somewhere between the Huasco and Copiapó river systems, while the disjunction between *Chilicola travesia* and *Chilicola erithropoda* lies along or just to the south of the Río Huasco. The third, a disjunction between *Chilicola mayu* and [*Chilicola curvapeligrosa* + *Chilicola mavida*], extends west across the Santiagan province (at ~35°S) somewhere between the Mantaquito-Teno-Lontué and Maule river systems.

Out of the seven remaining disjunctions mentioned above, at least three correspond to the boundary between the Coquimban and Santiagan provinces (~32.5–33°S), giving it particularly good support; these are the disjunctions between *Chilicola katherinae* and *Chilicola diaguita*, between *Chilicola mavida* and *Chilicola curvapeligrosa*, and between the two sister clades of group 3.2. This last disjunction precedes the one between *Chilicola mavida* and *Chilicola curvapeligrosa*, and actually extends north along the Andes to accommodate the range of *Chilicola mavida*; this can be interpreted as a disjunction at 32.5–33°S between the ranges of the northern clade [*Chilicola lickana* + (*Chilicola guanicoe* + *Chilicola vicugna*)] and the southern clade [*Chilicola lickana* + (*Chilicola guanicoe* + *Chilicola vicugna*)], followed by later allopatric dispersal or vicariance of *Chilicola mavida* along the Andes to the north. The Coquimban–Santiagan boundary separates semi-arid scrubland to the north from more humid sclerophyll and spiny (*espinal*) woodland to the south, though most of this woodland has been destroyed by heavy cultivation (Chester 2008, Peña 1966, Morrone 2015). A similar disjunction is present between *Chilicola mantagua* and *Chilicola deserticola*, with the distribution of *Chilicola mantagua* extending a little further north along the coast than the other Santiagan groups, to south of the Río Choapa.

The disjunction between *Chilicola neffi* and *Chilicola packeri* suggests a barrier somewhat north of Copiapó (~26.5°S) near the boundary between Peña’s (1966) Northern Coast and Intermediate Desert regions. The southern limit of the Northern Coast is pushed north in Morrone’s regionalisation (2015), but a boundary near ~26.5°S can be defined based on moisture: to the north the coastal zone is very dry and receives nearly all of its moisture from fog, while to the south rainfall is more common (Peña 1966). Moreover, just inland, this boundary lies along the southern extent of the Interior Desert district, sometimes known as the “Absolute Desert” (Chester 2008). The most pronounced disjunction with regard to moisture is that between *Chilicola lickana* and [*Chilicola guanicoe + Chilicola vicugna*], lying somewhere between the Coquimban province of the Central Chilean sub-region and the Puna province of the South American transition zone. *Chilicola lickana*’s distribution is the most arid of the subgeneric range, and this species may well have diverged as a result of one or more episodes of hyperaridity of the Atacama Desert (Amundson et al. 2012, Jordan et al. 2014).

The disjunction between *Chilicola vina* and [*Chilicola diaguita* + *Chilicola katherinae*] consists of two disjunctions: one in the north which crosses the Río Elqui near Vicuña, separating higher-elevation-inhabiting *Chilicola vina* from *Chilicola diaguita*, and one in the south across the Andes at around 34°S, south of which *Chilicola katherinae* occurs. The southern disjunction corresponds well to Peña’s boundary between the Central and Southern Andean Cordillera regions near the Río Tinguiririca, which also forms the northern boundary of Peña’s precordilleran Northern Valdivian Forest region (both were shifted in Morrone’s regionalisation, to the north and south, respectively). To the south of this boundary, an increase in humidity is marked by the appearance of *Nothofagus* forests (Peña 1966, Chester 2008). Depending on which of the phylogenetic hypotheses is considered, there are at least two possible interpretations for this apparent two-fold disjunction. Under the preferred phylogeny, one interpretation is that these three species diverged from one another in a relatively short time, which could help explain the discrepancy between molecular and morphological evidence as to whether *Chilicola vina* or *Chilicola katherinae* diverged first. Alternatively, in the morphology-only phylogeny, *Chilicola katherinae* is sister to the other two species (Fig. 50A); if this is taken as the true relationship between these three species, it would suggest two separate disjunctions, with the southern one preceding the northern one.

## Discussion

### Taxonomy

This revision increases the size of the subgenus *Heteroediscelis*; with the description of eight new species and the redescription of nine previously-described ones, the total is increased to seventeen (previously ten). Also included are three minor, but notable alterations to the taxonomic record: the synonymy of *Chilicola mantagua* with *Chilicola valparaiso*, the designation of a neotype specimen for *Chilicola vicugna*, and a re-interpretation of the type localities of *Chilicola erithropoda* and *Chilicola deserticola*. Details regarding the latter two are included under comments on the relevant species descriptions. The first was motivated by insufficient distinction between *Chilicola mantagua* and *Chilicola valparaiso*. The diagnosis for *Chilicola valparaiso* refers to two characters (Toro and Moldenke 1979): malar space subequal to minimum width of scape, and apical lamina of metatibia narrowing distally in apical view. In fact, the holotypes of *Chilicola mantagua* and *Chilicola valparaiso* both have the malar space approximately half as long as the clypeal lateral (i.e. of equal relative length), and the apical lamina of the metatibia is not differentiable between them. Moreover, characters of the male terminalia do not support any division between these two supposed species, and I have identified many specimens from the same broad geographic area as the *Chilicola valparaiso* holotype as either *Chilicola mantagua* or *Chilicola vina*. Indeed, the type locality of *Chilicola valparaiso* is just to the south of the originally-documented range of *Chilicola mantagua*. Finally, CO1 barcode sequences from specimens tentatively identified as *Chilicola valparaiso* are indistinguishable from those of *Chilicola mantagua*. Therefore, in absence of any evidence to maintain the distinction of *Chilicola valparaiso* as a species, I have treated it as a synonym of *Chilicola mantagua* – the latter is the type species of the subgenus and has page priority in the original publication (Toro and Moldenke 1979).

### DNA Barcodes

DNA barcoding was useful for the association of females to males of several species, particularly among those whose females are difficult to tell apart (e.g. *Chilicola deserticola*, *Chilicola erithropoda*, *Chilicola mantagua*, and *Chilicola vicugna*). As an exploratory tool, barcoding initially helped reveal the presence of two new species from localities new to the subgenus: *Chilicola randolphi* from Laguna del Maule and *Chilicola curvapeligrosa* from Farellones. Barcode sequences also served as supporting evidence for the description of morphologically distinct species, such as *Chilicola charizard*, *Chilicola lickana*, and *Chilicola mayu*. In three cases, however, DNA barcodes of distinct species clustered into a single BIN. These are: *Chilicola mavida* & *Chilicola curvapeligrosa*, *Chilicola vicugna* & *Chilicola guanicoe*, and *Chilicola vina*, *Chilicola diaguita* & *Chilicola katherinae* (Table 1). All seven of these species are supported by the morphological characters provided in the keys and descriptions, but are reduced to three when using BINs as a proxy for species (i.e. molecular operational taxonomic units – MOTUs; Floyd et al. 2002). On the other hand, there are three species which are represented by seven total BINs: *Chilicola mantagua* (two BINs), *Chilicola neffi* (three BINs) and *Chilicola packeri* (two BINs); as a result, BIN analysis overestimates the number of MOTUs found at low elevations and underestimates the number of MOTUs found at high elevations. DNA barcoding is indeed a rapid, efficient means to estimate diversity (e.g. Smith et al. 2005, Gibson et al. 2014) and can be invaluable as an aid in the discovery of new species (e.g. Gibbs 2009a, 2009b, Packer et al. 2009, Butcher et al. 2012), but in this specific case, at least, using BINs to evaluate patterns of diversity seems not to produce accurate results.

**Table 1.** Diagnostic or invariant DNA barcode nucleotide substitutions, and Barcode Index Numbers (BINs) for each species of Chilicola (Heteroediscelis). Nucleotide position is given relative to the CO1 sequence of *Apis mellifera*. Brackets indicate subtitutions which must co-occur in order to be diagnostic. Nucleotides in other species are listed in order of prevalence. BINs which are not unique to one species are italicized.

Species	Number of sequences	Diagnostic nucleotides	Most common nucleotide in other species	Barcode Index Number (BIN)
*Chilicola mantagua*	5	115 - C 640 - C	A A	BOLD:ACE7694 or BOLD:AAN4855
*Chilicola charizard*	5	19 - A 22 - A 44 - A 232 - C 266 - C 284 - T 290 - C 359 - C 361 - T 517 - T 604 - C	T,C T T A,T T A T T A A T	BOLD:AAO3429
*Chilicola curvapeligrosa*	5	232 - A 370 - C	A,T A	*BOLD:AAD5081*
*Chilicola deserticola*	5	241 - T	A	BOLD:ABZ0969
*Chilicola diaguita* & *Chilicola vina*	3 7	-	-	*BOLD:AAD5082*
*Chilicola erithropoda*	6	223 - C 359 - A 484 - C 544 - G	T,A T A A	BOLD:AAO1435
*Chilicola guanicoe* & *Chilicola vicugna*	6 9	337 - C	T	*BOLD:AAO3440* ^†^
*Chilicola katherinae*	3	133 - A 412 - T 595 - C	A A,T A,T	*BOLD:AAD5082*
*Chilicola lickana*	1 (+1)^*^	125 - A 244 - G 280 - C “356 - A” “380 - A” “622 - C”	G A T C G A	BOLD:ACL6898
*Chilicola mavida*	4	232 - T 553 - C	A,T A	*BOLD:AAD5081*
*Chilicola mayu*	2	364 - C 479 - C	T T	BOLD:ACL7424
*Chilicola neffi*	2	124 - A	T	BOLD:AAO4632, BOLD:ACO4269, or BOLD:ACP7825
*Chilicola packeri*	4	292 - G or 163 - C 202 - C 218 - T 253 - G 289 - G 325 - C	A T T G A A,T A	BOLD:ACL7140 or BOLD:ACL7007
*Chilicola randolphi*	4	5 - C 81 - T 290 - A 313 - C 382 - C 596 - C	T C T A,T T T	BOLD:AAD5080
*Chilicola travesia*	11	124 - C 526 - C 548 - C	T T,A T	BOLD:AAI4251

† this BIN includes one sequence from *Chilicola vina*, from which *Chilicola guanicoe* and *Chilicola vicugna* can be separated by the indicated substitution.

* this BIN contains only one sequence from *Chilicola lickana* and as such the substitutions in quotation marks may not be diagnostic; a second, partial (284 bp) sequence from *Chilicola lickana* shares the first three substitutions, but does not span the remaining three bases.

As a partial correction, a higher resolution of molecular species identification is possible by specifying diagnostic, single-nucleotide substitutions within barcode sequences (Fisher and Smith 2008, Gibbs 2009a, 2009b). I have summarized these for *Heteroediscelis* in Table 1. Of the seventeen species in the subgenus, thirteen have diagnostic nucleotide subsitutions (or diagnostic combinations of them) in their barcode sequences. Provided a specimen belongs to the subgenus, the presence of any one of these substitutions should result in succesful identification of the corresponding species. In particular, this approach allows molecular differentiation of *Chilicola mavida* and *Chilicola curvapeligrosa*, as well as separation of *Chilicola katherinae* from *Chilicola diaguita* & *Chilicola vina*; it also allows multiple BINs associated with a single species to be collapsed into a single MOTU. Given that diagnostic nucleotide subsitutions are very easily determined, the practice of identifying them in taxonomic revisions should be encouraged (Fisher and Smith 2008, Gibbs 2009a, 2009b).

### Phylogeny and biogeography

The phylogeny in Fig. 51 represents the most strongly supported evolutionary hypothesis for the subgenus, drawing on a total evidence approach to analysis (Eernisse and Kluge 1993, Huelsenbeck et al. 1996). A single tree was recovered with good GC/BS support for most of its branches, and with ample morphological support for all of them.

Aside from the morphological evidence supporting the various clades, this phylogeny includes groupings which have some biogeographic basis. Disjunctions in distributions of sister clades correspond in many cases to the biogeographic areas described by Morrone (2015) and Peña (1966), and in a few cases to boundaries identified by others. For example, both the southern disjunction between *Chilicola vina* and [*Chilicola diaguita* + *Chilicola katherinae*] and the disjunction between *Chilicola mayu* and [*Chilicola curvapeligrosa* + *Chilicola mavida*] correspond to a vicariance event identified by Dominguez et al. (2016) in tenebrionid beetles and phasmids, and indeed Peña’s boundary between the Central and Southern Andean Cordillera falls in the same general area, representing the northern limit of moderate-elevation *Nothofagus* woodlands (Peña 1966, Chester 2008). As well, the disjunction between *Chilicola lickana* and [*Chilicola guanicoe* + *Chilicola vicugna*] unambiguously places these taxa on either side of an occasionally-cited summer/winter rainfall divide across northern Chile, which arose 2–2.2 Mya (Amundson et al. 2012), marking the boundary between the tropical and mid-latitude climate zones (Arroyo et al. 1988, Messerli et al. 1993, Houston 2006).

While a few of the earlier divergences have associated disjunctions across an elevational gradient, most of the recent divergences exhibit disjunctions across the latitudinal moisture gradient which extends from the arid north to the temperate south. These disjunctions may be the result of varying degrees of aridification in northern Chile, given that the Atacama Desert reached its present hyperarid state sometime after the origin of *Chilicola* (Almeida et al. 2011, Dunai et al. 2005, Rech et al. 2010), and perhaps around the same time as the origin of *Heteroediscelis* (Jordan et al. 2014). In any case, divergence along this gradient is the primary pattern for more closely related taxa, and in many cases coincides with well-documented shifts in vegetation, such as the shift from semi-arid scrubland to sclerophyll and spiny woodland across the Coquimban-Santiagan boundary, and the appearance of *Nothofagus* woodlands south of ~34°S noted above (Peña 1966, Chester 2008).

Although ancestral distributions cannot be inferred from the analysis used in the present study, one can speculate: the grade comprising [*Chilicola neffi* + *Chilicola packeri*], [*Chilicola deserticola* + *Chilicola mantagua*], and [*Chilicola erithropoda* + *Chilicola travesia*] is collectively distributed along the primarily coastal and low-elevation portions of the Coquimban & Intermediate Deserts, which could suggest an ancestral distribution along the semi-arid mid-northern coast and interior (commonly called the *Norte Chico*; Sánchez and Morales 1990). This would be consistent with the designation of *Heteroediscelis* as endemic to Chile, with Andean (i.e. high-elevation) and latitudinally more extreme distributions potentially representing derived ranges.

With the completion of this phylogenetic analysis, in addition to the work done by Gibbs and Packer (2006) on *Chilicola* *s. str.* and by Willis and Packer (2007) on *Chilioediscelis*, revisionary work on the clade [*Heteroediscelis* + (*Chilicola* *s. str.* + *Chilioediscelis*)] found in Packer’s subgeneric phylogeny (2008) is fit for synthesis. Combined, these three revisions provide an exhaustive set of data well-suited to a thorough analysis of the clade, which could incorporate discrete morphological, continuous morphological, and molecular evidence. In light of this – along with the eight species newly described herein, plus additional new discoveries in *Chilicola* *s. str.* and *Chilioediscelis* (L. Packer, personal communication) – a combined phylogenetic analysis of these three subgenera together with *Oediscelis* is recommended in order to re-assess subgeneric boundaries and relationships within *Chilicola*, and to investigate biogeographic patterns throughout its broad neotropical distribution.

## Supplementary Material

XML Treatment for Chilicola (Heteroediscelis)

XML Treatment for Chilicola (Heteroediscelis) mantagua

XML Treatment for Chilicola (Heteroediscelis) charizard

XML Treatment for Chilicola (Heteroediscelis) curvapeligrosa

XML Treatment for Chilicola (Heteroediscelis) deserticola

XML Treatment for Chilicola (Heteroediscelis) diaguita

XML Treatment for Chilicola (Heteroediscelis) erithropoda

XML Treatment for Chilicola (Heteroediscelis) guanicoe

XML Treatment for Chilicola (Heteroediscelis) katherinae

XML Treatment for Chilicola (Heteroediscelis) lickana

XML Treatment for Chilicola (Heteroediscelis) mavida

XML Treatment for Chilicola (Heteroediscelis) mayu

XML Treatment for Chilicola (Heteroediscelis) neffi

XML Treatment for Chilicola (Heteroediscelis) packeri

XML Treatment for Chilicola (Heteroediscelis) randolphi

XML Treatment for Chilicola (Heteroediscelis) travesia

XML Treatment for Chilicola (Heteroediscelis) vicugna

XML Treatment for Chilicola (Heteroediscelis) vina

## Appendix 1

### List of characters

Characters 1–54 are represented by discrete states; characters 55–74 are continuous and based upon ratios.

1. Male, colour of clypeal apex: (0) yellow full width (Fig. 10B, 11D); (1) brown laterad (Figs 10D, 11A).
2. Male, clypeus colour: (0) entirely yellow except along epistomal suture (Fig. 41B); (1) yellow inverted T shape (Fig. 48B).
3. Male, clypeal median depression: (0) absent or marked by a very weak/faint depression (Fig. 7B); (1) present and strong for less than half of clypeal length, absent or weak in ventral half (Fig. 41B); (2) present and strong throughout entire clypeal length (Fig. 7A).
4. Male, clypeal sculpture: (0) moderately densely punctate (i=1-2d) throughout; (1) sparsely punctate (i=2-3d) medially.
5. Male, F1: (0) at most as long as wide (Fig. 10A); (1) longer than wide (Fig. 10C).
6. Male, ratio of length of F2:F1: (0) subequal, <1.1x (Fig. 16E); (1) ≥1.2x (Fig. 16B).
7. Male, ratio of length of F3:F2: (0) subequal, <1.05x; (1) ≥1.1x.
8. Male, frontal line: (0) flat/indistinct, or carinate only between antennal bases; (1) carinate for 1/2 to 3/4 distance from supraclypeal area to median ocellus.
9. Male, lower paraocular maculation: (0) extending <1OD above transverse portion of epistomal suture (Fig. 36B); (1) extending ~1OD above transverse portion of epistomal suture (Fig. 32B); (2) reaching/nearly reaching lower tangent of antennal socket (Fig. 38B).
10. Female, colour of F1 and F2 ventrally: (0) mostly brown or black (Fig. 36D); (1) at least F2 mostly yellow or yellow-brown (Fig. 45D).
11. Female, clypeal sculpture: (0) moderately densely punctate throughout, i=1-2d (Fig. 27D); (1) sparsely punctate medially, i≥2d (Fig. 27B).
12. Female, ratio of length of F3:F2: (0) subequal, ≤1.1×; (1) ≥1.2×.
13. Female, longitudinal striae on frontal area: (0) absent (Gibbs and Packer 2006: Fig. 13C); (1) present (Gibbs and Packer 2006: Fig. 13D).
14. Genal beard: (0) moderately dense in male and sparse in female (Fig. 43A, 43C); (1) dense in male and sparse in female (Fig. 47A, C); (2) dense in male and moderately dense in female (Fig. 36A, C).
15. Projection of clypeus: (0) projecting for less than half its length beyond LOT (Fig. 10C); (1) projecting for half its length or more beyond LOT (Fig. 10A).
16. Clypeus above anterior tentorial pit: (0) unmodified, projecting ≤1OD above compound eye in lateral view (Figs 28C, 16F); (1) convex, bulging ~1.5OD above compound eye in lateral view (Figs 20A, 10B); (2) strongly convex, bulging ~2OD above compound eye in lateral view (Fig. 28A, 10D).
17. Frontal area: (0) without longitudinal depressions (Figs 3B, 25B); (1) with longitudinal depressions which partially accommodate the scape (Figs 3A, 25A).
18. Malar space: (0) <0.9× clypeal lateral (Fig. 8A); (1) ~1× clypeal lateral (Fig. 6C); (2) 1.2-1.7× clypeal lateral (Fig. 6A); (3) ≥2× clypeal lateral (Fig. 12A).
19. Male, metatibia shape: (0) 2.0-2.2× as long as maximum depth, summit of ventral convexity at two-thirds to three-quarters tibial length (Fig. 15E); (1) ~1.67× as long as maximum depth, summit of ventral convexity at two-thirds to three-quarters tibial length (Fig. 37A); (2) <2.0× as long as maximum depth, summit of ventral convexity at 0.85× tibial length (Fig. 15B).
20. Male, ventral metatibial carina: (0) greatly reduced, originating in apical half of posterior carina (Fig. 43E); (1) absent basally, originating at midpoint of posterior carina (Fig. 34E); (2) extending full length of longitudinal concavity and angled ventrad, rounded inflection near midpoint (Fig. 35E); (3) extending full length of longitudinal concavity and toothed (Fig. 36E).
21. Male, posterior metatibial carina: (0) absent basally, originating in apical half of ventral carina (Fig. 39E); (1) sublinear and weakly defined basally (Fig. 36E); (2) extending full length of longitudinal concavity and weakly sigmoid (Fig. 47E); (3) extending full length of longitudinal concavity and sharply inflected near base (Fig. 38E).
22. Male, midleg colour: NRLC axis 1 (See Appendix 3).
23. Male, midleg colour: NRLC axis 2 (See Appendix 3).
24. Male, hindleg colour: NRLC axis 1 (See Appendix 3).
25. Male, hindleg colour: NRLC axis 2 (See Appendix 3).
26. Male, mesepisternum: (0) punctures anterior of episternal groove same density as those below scrobe; (1) punctures anterior of episternal groove sparser than those below scrobe.
27. Male, prodistitarsus: (0) entirely brown or black; (1) apical one-third/half yellow-brown or yellow.
28. Male, basal vein length before Cu: (0) at most half as long as Rs (Willis and Packer 2007: Fig. 12D); (1) more than half as long as Rs (Willis and Packer 2007: Fig. 12E).
29. Female, pronotal lobe colour: (0) dark; (1) with yellow posterior margin.
30. Female, mesepisternum: (0) punctures anterior of episternal groove same density as those below scrobe; (1) punctures anterior of episternal groove sparser than those below scrobe.
31. Female, prodistitarsus: (0) entirely brown or black; (1) apical one-third/half yellow-brown or yellow.
32. Female, protibia dorsal surface: (0) yellow for entire length (Fig. 17B–C); (1) yellow in basal half only (Fig. 17A).
33. Female, mid-/hindleg colour: NRLC axis 1 (Appendix 3).
34. Female, mid-/hindleg colour: NRLC axis 2 (Appendix 3).
35. Metatibial spurs: (0) unsclerotised and straight (Gibbs and Packer 2006: Fig. 15E); (1) sclerotised and strongly curved (Gibbs and Packer 2006: Fig. 15F).
36. Metepisternum: (0) without longitudinal striae anterodorsally; (1) distinctly longitudinally striate anterodorsally.
37. Male, S1: (0) slightly swollen apicoventrally (Packer 2007: Fig. 4U); (1) with long pointed process (Packer 2007: Fig. 4S); (2) with long truncate process (Packer 2007: Fig. 4T).
38. Male, S1 process apical surface: (0) longitudinally concave (Fig. 5B); (1) concave posteriorly, convex anteromedially (Fig. 2C, 5D); (2) weakly convex (Fig. 5C); (3) distinct longitudinal median ridge and otherwise flat/weakly concave (Fig. 5A).
39. Male, S1 process anterior and posterior margins in profile: (0) convergent apically (Fig. 32F); (1) subparallel (Fig. 45F); (2) divergent apically (Fig. 28F).
40. Male, S2 pubescence: (0) ≥1.5OD long basally and 3× longer basally than apically; (1) ≥1.5OD long basally and 1.5× longer basally than apically; (2) reduced, no longer than 1OD basally.
41. Male, T7 apical margin: (0) brown or black; (1) yellow.
42. Male, S7 ventral lobe: (0) narrow, almost linear (Fig. 34H); (1) ~0.25× as broad as basal attached length (Fig. 37H); (2) ~0.33× as broad as basal attached length (Fig. 39H); (3) ~0.5× as broad as basal attached length (Fig. 32H); (4) much broader than basal attached length (Gibbs and Packer 2006: Fig. 16B).
43. Male, S7 dorsal lobe orientation: (0) main body transverse, basal portion recurved anterolaterally (Willis and Packer 2007: Fig. 16A; in *Heteroediscelis* basal portion is present as an anteroventral bulge); (1) curved posteriorly (Willis and Packer 2007: Fig. 16B); (2) transverse & almost parallel-sided, neither curved posteriorly nor with basal recurved portion (Gibbs and Packer 2006: Fig. 1E).
44. Male, S7 dorsal lobe shape: (0) roughly triangular, wide at base narrowing to apex (Fig. 47H); (1) strap-like, subparallel-sided and somewhat anteriorly curved (Fig. 32H); (2) digitiform, apex widened relative to midlength, somewhat anteriorly curved (Fig. 44H).
45. Male, S7 dorsal lobe setation: (0) row of setae long basally and shorter apically (Fig. 47H); (1) row of broad setae mesobasally to apex (Fig. 44H); (2) basal tuft of long setae and a short apical row, midlength bare (Fig. 32H); (3) without appreciable setation.
46. Male, S7 apex of disc: (0) sinuate (32H); (1) distinctly emarginated (Fig. 39H); (2) nearly linear (Fig. 42H).
47. Male, S7 apodemal arm: (0) sclerotized margin entire, enclosing membranous portion of arm (Fig. 44H); (1) sclerotized margin terminating laterally at arm midlength (Fig. 48H); (2) sclerotized margin recurved mesad, dividing membranous portion of arm (Fig. 33H).
48. Male, S8 lateral process: (0) anteromedial sclerotized margin following marginal contour (Fig. 32I); (1) anteromedial sclerotized margin swollen forming convex incursion (Fig. 43I); (2) with anteromedial sclerotized spot disjunct from margin (Fig. 39I).
49. Male, S8 lateral process: (0) ratio of width:length ~1-1.1×, apicolateral margin evenly rounded or weakly concave (Fig. 33I); (1) ratio of width:length ≤0.9×, apicolateral margin evenly rounded or weakly concave (Fig. 34I); (2) ratio of width:length ≥1.2×, apicolateral margin evenly rounded or weakly concave (Fig. 48I); (3) ratio of width:length ~1.1×, apicolateral margin distinctly concave (Fig. 37I).
50. Male, S8 spiculum: (0) short (Gibbs and Packer 2006: Fig. 16C); (1) long (Gibbs and Packer 2006: Fig. 16D).
51. Male, gonobase, apicoventral truncate process: (0) biconvex and roundly emarginated (Fig. 40J); (1) biconvex and distinctly notched by a width equal to or less than one convexity (Fig. 33J); (2) biconvex and distinctly notched by a width ~1.5-2× one convexity (Fig. 38J).
52. Male, gonoforceps, mesoventral lobe: (0) evenly rounded (Fig. 35J); (1) anterior and posterior margins concave (Fig. 40J); (2) broad, width of lobe approximately equal minimum width of gonoforceps (Fig. 44J).
53. Female, metasoma: (0) mostly black-brown (Fig. 18C); (1) mostly orange-brown (Fig. 18F).
54. Female, terga: (0) T1-T3 with apicolateral patches of tomentum (Figs 18C, 27C); (1) T1-T2 with apicolateral patches of tomentum tomentose, but apicolateral hair band sparse on T3 (Figs 18F, 27A).
55. Male, ratio of clypeus width:minimum distance between subantennal sutures.
56. Female, ratio of clypeus width:minimum distance between subantennal sutures.
57. Male, ratio of subantennal suture length:minimum distance between them.
58. Female, ratio of subantennal suture length:minimum distance between them.
59. Male, ratio of IAD:AOD.
60. Female, ratio of IAD:AOD.
61. Male, ratio of scape length:maximum width.
62. Male, ratio of UOD:LOD.
63. Male, ratio of OOC:IOC.
64. Female, ratio of OOC:IOC.
65. Male, ratio of probasitarsus length:depth.
66. Female, ratio of probasitarsus length:depth.
67. Male, ratio of prodistitarsus length:combined length of tarsomeres 2-4.
68. Female, ratio of prodistitarsus length:combined length of tarsomeres 2-4.
69. Male, ratio of metafemur length:depth.
70. Female, ratio of metafemur length:depth.
71. Female, ratio of metatibia length:depth.
72. Male, ratio of metabasitarsus length:depth.
73. Male, ratio of metapostnotum length:mesoscutum length.
74. Female, ratio of metapostnotum length:mesoscutum length.

## Appendix 2

### Data matrix

Morphological data used in the phylogenetic analysis is summarized in the tables below. For DNA barcode data used in the phylogenetic analysis and for the specification of diagnostic nucleotide substitutions, see Suppl. material 3: DNA Barcode Data.

**Table A1.** Discrete morphological data used in phylogenetic analysis.

Character #	1	2	3	4	5	6	7	8	9	10	11	12	13	14	15	16	17	18	19	20	21	22	23	24	25	26	27
Chilicola (Oediscelis) vernalis	1	0	0	1	1	1	0	0	1	0	1	0	0	0	0	0	0	0	2	?	3	1	2	0	2	0	1
Chilicola (Chilioediscelis) penai	1	0	2	1	0	1	1	0	0	0	1	0	1	1	1	2	0	0	-	-	-	3	3	3	2	0	1
*Chilicola* (*s. str.*) *rubriventris*	1	1	1	1	0	1	0	0	0	0	1	1	1	2	0	0	0	0	2	0	?	1	2	1	0	0	1
Chilicola (Heteroediscelis) charizard	1	0	0	1	0	1	1	1	1	1	1	1	0	1	1	0	0	3	0	2	0	0	2	0	2	1	0
Chilicola (Heteroediscelis) curvapeligrosa	0	0	1	1	0	1	0	0	1	0	1	1	0	1	1	1	0	1	2	1	2	2	2	2	2	1	0
Chilicola (Heteroediscelis) deserticola	0	0	0	0	0	1	0	1	1	0	0	1	0	1	0	1	0	0	0	2	0	3	3	1	3	0	1
Chilicola (Heteroediscelis) diaguita	1	1	1	1	0	1	1	0	0	0	1	1	0	0	1	0	0	2	2	3	1	0	2	0	2	1	0
Chilicola (Heteroediscelis) erithropoda	0	0	0	1	0	0	1	1	2	0	0	0	0	1	0	0	0	0	1	3	0	5	2	5	2	0	1
Chilicola (Heteroediscelis) guanicoe	0	0	1	1	0	1	1	0	2	1	0	1	0	1	1	0	0	2	0	1	3	3	2	4	2	1	0
Chilicola (Heteroediscelis) katherinae	1	1	2	0	0	1	1	1	0	0	1	1	0	0	1	1	0	1	0	2	0	0	2	0	2	1	1
Chilicola (Heteroediscelis) lickana	0	0	2	1	0	1	1	0	1	1	1	1	0	1	1	0	0	3	0	1	3	3	1	1	1	0	0
Chilicola (Heteroediscelis) mantagua	0	0	0	0	0	0	1	1	1	0	0	0	0	1	0	0	0	0	0	2	0	1	2	1	2	0	1
Chilicola (Heteroediscelis) mavida	0	0	1	0	1	1	0	1	1	0	1	1	0	1	1	1	0	1	2	1	2	3	2	3	2	1	0
Chilicola (Heteroediscelis) mayu	1	1	2	1	1	1	1	0	0	0	1	1	0	2	0	2	0	0	2	1	2	3	1	2	2	1	0
Chilicola (Heteroediscelis) neffi	1	1	1	1	0	1	0	0	0	0	0	1	0	0	0	0	1	0	0	0	3	0	2	1	2	0	1
Chilicola (Heteroediscelis) packeri	1	1	1	1	0	1	1	1	1	0	0	1	0	0	0	0	1	0	0	0	3	3	0	1	2	0	1
Chilicola (Heteroediscelis) randolphi	1	0	2	1	0	1	0	1	2	1	1	1	0	2	0	1	0	0	2	3	1	4	2	3	2	1	0
Chilicola (Heteroediscelis) travesia	0	0	0	1	0	0	1	1	2	0	0	1	0	0	0	0	0	0	1	3	0	5	2	5	2	0	1
Chilicola (Heteroediscelis) vicugna	0	0	1	1	0	1	1	0	2	0	1	0	0	1	0	0	0	0	0	1	2	3	0	1	0	1	1
Chilicola (Heteroediscelis) vina	1	1	1	1	0	1	1	1	0	0	0	1	0	0	1	1	0	2	2	3	1	0	2	0	2	1	0
Character #	28	29	30	31	32	33	34	35	36	37	38	39	40	41	42	43	44	45	46	47	48	49	50	51	52	53	54
Chilicola (Oediscelis) vernalis	1	0	1	1	1	0	0	0	0	0	-	-	2	0	1	0	2	3	0	1	0	2	0	1	0	0	1
Chilicola (Chilioediscelis) penai	0	0	0	0	0	1	0	1	1	0	-	-	2	1	2	2	0	2	0	0	0	2	1	2	0	0	0
*Chilicola* (*s. str.*) *rubriventris*	0	0	0	1	1	0	0	1	1	1	-	0	2	1	4	1	2	1	2	1	1	0	1	0	0	1	0
Chilicola (Heteroediscelis) charizard	1	1	1	0	0	1	0	0	0	2	1	2	1	0	0	0	1	2	0	2	0	0	1	1	1	0	0
Chilicola (Heteroediscelis) curvapeligrosa	1	1	1	0	0	1	0	0	1	2	3	1	1	1	0	0	0	0	1	1	0	1	1	1	0	0	1
Chilicola (Heteroediscelis) deserticola	1	1	1	0	0	3	0	0	1	2	0	0	0	0	0	0	1	2	0	1	0	1	1	2	0	0	0
Chilicola (Heteroediscelis) diaguita	1	1	1	0	1	0	0	0	0	2	0	2	1	0	0	0	1	2	0	1	0	2	1	1	1	0	1
Chilicola (Heteroediscelis) erithropoda	1	1	0	1	0	5	0	0	0	2	1	2	2	1	1	0	1	0	1	0	0	3	1	2	1	0	0
Chilicola (Heteroediscelis) guanicoe	1	1	1	1	0	4	0	0	0	2	3	0	0	1	0	0	0	0	1	1	0	1	1	2	0	0	0
Chilicola (Heteroediscelis) katherinae	1	1	1	1	1	0	0	0	1	2	0	2	1	0	1	0	1	2	1	2	2	2	1	2	1	0	1
Chilicola (Heteroediscelis) lickana	1	1	0	1	0	4	0	0	1	2	3	0	0	1	0	0	0	0	1	1	1	1	1	0	1	0	1
Chilicola (Heteroediscelis) mantagua	1	1	0	0	0	1	1	0	1	2	0	0	0	0	3	0	1	2	0	0	0	0	1	2	0	0	1
Chilicola (Heteroediscelis) mavida	1	1	1	0	0	1	2	0	1	2	3	0	0	1	0	0	0	0	1	1	0	1	1	2	0	0	0
Chilicola (Heteroediscelis) mayu	1	1	1	0	0	1	0	0	1	2	3	1	1	1	0	0	0	0	2	2	0	2	1	0	1	0	1
Chilicola (Heteroediscelis) neffi	1	1	1	1	0	2	0	0	1	2	2	0	1	0	3	0	2	1	1	0	1	1	1	2	2	0	1
Chilicola (Heteroediscelis) packeri	1	1	1	0	0	2	0	0	0	2	2	0	1	1	3	0	2	1	1	0	0	1	1	2	2	1	1
Chilicola (Heteroediscelis) randolphi	1	1	1	0	1	1	1	0	1	2	0	1	1	1	3	0	1	2	0	1	0	0	1	1	1	1	0
Chilicola (Heteroediscelis) travesia	1	1	0	1	0	6	0	0	0	2	0	2	2	1	1	0	1	0	1	1	2	3	1	1	0	0	0
Chilicola (Heteroediscelis) vicugna	1	1	1	0	0	4	0	0	1	2	3	0	0	0	0	0	0	0	1	1	0	1	1	2	0	0	1
Chilicola (Heteroediscelis) vina	1	1	1	0	1	0	0	0	1	2	0	2	0	0	2	0	1	2	0	1	1	2	1	1	1	0	1

**Table A2.** Continuous morphological data used in phylogenetic analysis.

Character #	55	56	57	58	59	60	61	62	63	64
Chilicola (Oediscelis) vernalis	0.523	0.317	0.492	0.459	0.220	0.000	0.804	1.000	1.000	1.000
Chilicola (Chilioediscelis) penai	0.176	0.063	0.056	0.000	0.000	0.361	1.000	0.000	0.573	0.788
*Chilicola* (*s. str.*) *rubriventris*	0.294	0.133	0.000	0.170	0.047	0.265	0.727	0.528	0.569	0.604
Chilicola (Heteroediscelis) charizard	0.075	0.744	0.761	1.000	0.805	0.638	0.359	0.822	0.205	0.139
Chilicola (Heteroediscelis) curvapeligrosa	0.150	0.194	0.340	0.477	0.715	0.587	0.283	0.585	0.296	0.357
Chilicola (Heteroediscelis) deserticola	0.411	0.333	0.391	0.419	0.337	0.806	0.172	0.700	0.296	0.540
Chilicola (Heteroediscelis) diaguita	0.411	0.151	0.726	0.541	0.285	0.451	0.144	0.621	0.231	0.185
Chilicola (Heteroediscelis) erithropoda	0.929	0.641	0.590	0.442	0.166	0.587	0.364	0.238	0.098	0.099
Chilicola (Heteroediscelis) guanicoe	0.689	0.556	0.596	0.649	0.805	0.491	0.127	0.700	0.296	0.185
Chilicola (Heteroediscelis) katherinae	0.541	0.052	0.449	0.435	0.185	0.357	0.227	0.443	0.320	0.250
Chilicola (Heteroediscelis) lickana	1.000	1.000	1.000	0.911	0.439	0.331	0.144	0.310	0.296	0.117
Chilicola (Heteroediscelis) mantagua	0.495	0.296	0.391	0.326	0.220	0.683	0.283	0.506	0.350	0.409
Chilicola (Heteroediscelis) mavida	0.579	0.333	0.619	0.387	1.000	0.491	0.127	0.630	0.220	0.359
Chilicola (Heteroediscelis) mayu	0.144	0.342	0.333	0.360	0.461	0.477	0.327	0.581	0.410	0.439
Chilicola (Heteroediscelis) neffi	0.882	0.744	0.695	0.567	0.303	1.000	0.394	0.519	0.325	0.326
Chilicola (Heteroediscelis) packeri	0.156	0.444	0.275	0.485	0.387	0.587	0.080	0.432	0.189	0.200
Chilicola (Heteroediscelis) randolphi	0.012	0.226	0.181	0.287	0.659	0.740	0.000	0.310	0.536	0.339
Chilicola (Heteroediscelis) travesia	0.901	0.280	0.488	0.329	0.366	0.587	0.127	0.524	0.000	0.000
Chilicola (Heteroediscelis) vicugna	0.000	0.000	0.272	0.288	1.000	0.806	0.727	0.906	0.350	0.295
Chilicola (Heteroediscelis) vina	0.147	0.171	0.434	0.414	0.303	0.331	0.227	0.822	0.296	0.270
Character #	65	66	67	68	69	70	71	72	73	74
Chilicola (Oediscelis) vernalis	1.000	0.972	0.952	0.228	0.463	0.929	1.000	1.000	0.000	0.000
Chilicola (Chilioediscelis) penai	0.219	0.341	0.674	0.528	1.000	0.000	0.000	?	0.471	0.990
*Chilicola* (*s. str.*) *rubriventris*	0.054	0.000	0.806	0.528	0.665	0.240	0.443	0.419	0.559	0.516
Chilicola (Heteroediscelis) charizard	0.470	1.000	0.000	0.041	0.859	0.844	0.512	0.527	0.658	0.761
Chilicola (Heteroediscelis) curvapeligrosa	0.235	0.273	0.525	0.817	0.350	0.532	0.548	0.313	0.919	0.993
Chilicola (Heteroediscelis) deserticola	0.425	0.451	0.226	0.272	0.457	0.562	0.656	0.185	0.680	0.813
Chilicola (Heteroediscelis) diaguita	0.387	0.574	0.348	0.325	0.658	0.740	0.561	0.548	0.698	0.711
Chilicola (Heteroediscelis) erithropoda	0.381	0.304	0.195	0.281	0.000	0.395	0.377	0.000	0.680	0.300
Chilicola (Heteroediscelis) guanicoe	0.378	0.439	0.613	0.249	0.855	0.740	0.634	0.536	0.587	0.757
Chilicola (Heteroediscelis) katherinae	0.473	0.295	0.392	0.427	0.866	0.694	0.532	0.916	0.722	0.717
Chilicola (Heteroediscelis) lickana	0.357	0.709	0.645	0.528	0.482	0.565	0.781	0.512	0.813	0.662
Chilicola (Heteroediscelis) mantagua	0.219	0.273	0.419	0.469	0.857	0.428	0.548	0.185	0.503	0.501
Chilicola (Heteroediscelis) mavida	0.193	0.345	0.636	1.000	0.708	0.629	0.689	0.263	0.613	0.751
Chilicola (Heteroediscelis) mayu	0.098	0.169	0.525	0.240	0.280	0.642	0.392	0.244	0.839	0.923
Chilicola (Heteroediscelis) neffi	0.271	0.473	0.613	0.672	0.399	0.680	0.522	0.341	0.758	0.489
Chilicola (Heteroediscelis) packeri	0.425	0.451	0.015	0.336	0.717	0.740	0.586	0.441	0.513	1.000
Chilicola (Heteroediscelis) randolphi	0.000	0.439	1.000	0.413	0.625	1.000	0.728	0.042	1.000	0.969
Chilicola (Heteroediscelis) travesia	0.425	0.473	0.000	0.000	0.263	0.795	0.313	0.325	0.566	0.359
Chilicola (Heteroediscelis) vicugna	0.219	0.615	0.226	0.208	0.412	0.502	0.598	0.542	0.786	0.610
Chilicola (Heteroediscelis) vina	0.425	0.709	0.636	0.312	0.866	0.844	0.542	0.726	0.993	0.703

## Appendix 3

### Non-redundant linear coding

Three character state trees were constructed for non-redundant linear coding of potentially confounded leg colour characters (O’Grady and Deets 1987). Each sequence of numbers refers to a combination of colour character states for the mid- or hindleg; roughly speaking, these are scored as black/brown (0), narrowly/partially yellow (1), broadly/mostly yellow (2), and entirely yellow (3). Given that some combinations are not observed, it is probable that changes in colour of different leg regions occur in concert; thus, these character state combinations are shown as linked by single evolutionary changes. Bolded numbers indicate character states derived from these trees, and the resultant NRLC characters (22–25, 33–34) are coded as additive in the analysis.

## Supplementary materials

Supplementary material 1 Character-Annotated Phylogeny Data type: Phylogeny Explanation note: Figure S1. Phylogeny with evolutionary character changes mapped onto branches. Character numbers are above branches with corresponding states below. Solid circles indicate unique changes, open circles indicate homoplasies. Character optimization is colour-coded as black (unambiguous), red (fast), or blue (slow). This dataset is made available under the Open Database License (http://opendatacommons.org/licenses/odbl/1.0/). The Open Database License (ODbL) is a license agreement intended to allow users to freely share, modify, and use this Dataset while maintaining this same freedom for others, provided that the original source and author(s) are credited.Spencer K. Monckton

Supplementary material 2 Material Studied Data type: Occurrence & collection data Explanation note: Specimen data for 1316 specimens, including localities, geographic coordinates, and depositories. This dataset is made available under the Open Database License (http://opendatacommons.org/licenses/odbl/1.0/). The Open Database License (ODbL) is a license agreement intended to allow users to freely share, modify, and use this Dataset while maintaining this same freedom for others, provided that the original source and author(s) are credited.Spencer K. Monckton

Supplementary material 3
DNA Barcode Data Data type: Sequence data Explanation note: CO1 sequence data used in phylogenetic and diagnostic nucleotide analyses, including BOLD sequence IDs and GenBank accession numbers. This dataset is made available under the Open Database License (http://opendatacommons.org/licenses/odbl/1.0/). The Open Database License (ODbL) is a license agreement intended to allow users to freely share, modify, and use this Dataset while maintaining this same freedom for others, provided that the original source and author(s) are credited.Spencer K. Monckton
