# Supplementary figures and images for: A revision of Chilicola (Heteroediscelis), a subgenus of xeromelissine bees (Hymenoptera, Colletidae) endemic to Chile: taxonomy, phylogeny, and biogeography, with descriptions of eight new species
Source: Zookeys. 2016 May 19;(591):1–144. doi: 10.3897/zookeys.591.7731 (PMC4926649; doi:10.3897/zookeys.591.7731)

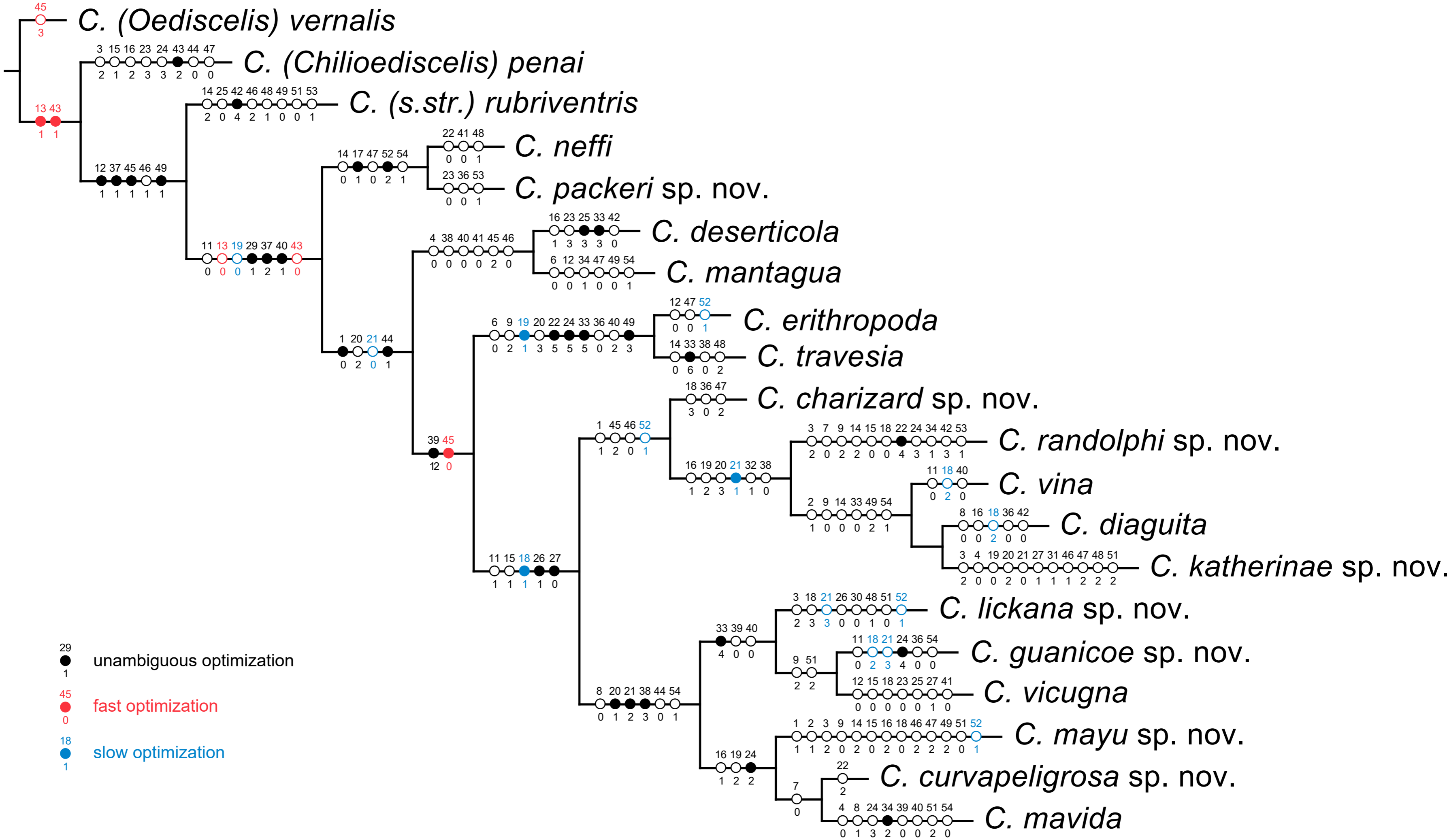

Supplement: Supplementary material 1 — Character-Annotated Phylogeny [file zookeys-591-001-s001.pdf]
